# Stroke Prevalence in a Coastal Town on the Black Sea Coast in Turkey: Community Based Study

**DOI:** 10.1155/2018/8246123

**Published:** 2018-07-02

**Authors:** Cansu Köseoğlu Toksoy, Cem Bölük, Ülkü Türk Börü, Seydahmet Akın, Abdullah Yasir Yılmaz, Sanem Coşkun Duman, Mustafa Taşdemir

**Affiliations:** ^1^Gaziosmanpaşa Taksim Training and Research Hospital, Department of Neurology, Istanbul, Turkey; ^2^University of Health Sciences, Dr. Lütfi Kırdar Kartal Training and Research Hospital, Department of Neurology, Istanbul, Turkey; ^3^University of Health Sciences, Dr. Lütfi Kırdar Kartal Training and Research Hospital, Department of Internal Medicine, Istanbul, Turkey; ^4^Ankara University, Department of Neurology, Ankara, Turkey; ^5^Istanbul Medeniyet University, Department of Public Health, Istanbul, Turkey

## Abstract

**Background:**

This study aims to determine the stroke prevalence in Akçakoca which is a rural area in Turkey.

**Methods:**

The study was designed as a cross-sectional, door-to-door survey. The stroke questionnaire was completed by a trained team in the presence of the participants according to their answers. Based on the screenings, patients who had been diagnosed with stroke previously were reexamined by a neurologist and determined the prevalence values, risk factors, and stroke types.

**Results:**

A total of 3750 people over 44 years old were reached in the screenings. It was determined that 83 people had previously suffered a stroke. The prevalence rate of stroke in those above 44 years was found to be 2.2 (98% confidence level and ± 2% margin of error). 70 (84.3%) patients had suffered an ischemic stroke while 12 (14.5%) had suffered a hemorrhagic stroke. Male/female ratio was 1.1.

**Conclusion:**

The results of this study give the prevalence rate of stroke among the Turkish population living in a rural area. Due to a lack of other similar studies, it is impossible to make any data comparison. However, the results of this study help shed light on the stroke prevalence rate.

## 1. Introduction

Stroke is the third most common cause of death in developed countries after coronary heart disease and cancer [1, 2]. The prevalence rate of stroke is increasing and changing all over the world, and so is its economic burden [3]. It has been estimated that, by the year 2030, around 80% of all stroke cases will be seen in low and middle income countries of the world [4].

As far as is known, the prevalence is changing between 0.3% and 2.1% in low and middle income countries [5, 6].

Turkey, as a developing middle income country, has currently no active nationwide registry for stroke and the prevalence remains unclear. Accurate data on stroke is very limited. Unfortunately, there are only two published studies on the prevalence rate of stroke in Turkey. The prevalence in the ≥45 age group is between 0.9% and 4.1% [7, 8].

The study aimed to shed more light on the prevalence rate of stroke in Turkey by investigating another rural coastal town named Akçakoca.

## 2. Materials and Methods

We conducted descriptive, cross-sectional, door-to-door surveys in a joint effort with neurologists from Dr. Lütfi Kırdar Kartal Training and Research Hospital and public health specialists from Istanbul Medeniyet University. We followed the methods of Şensöz et al. [8]. This study was conducted after another study that aimed to estimate the prevalence rate of multiple sclerosis.

### 2.1. Study Area

Akçakoca is a little coastal town linked to Düzce province, located in the western Black Sea region at latitude 41.05°N and longitude 31.07°E, with a 35-km-long coastline along the Black Sea and covering an area of 462 km^2^. Almost half of its surface is covered in nut trees. During the winter, it is rainy and in the summer it is mild, and annual rainfall is approximately 1 meter. The population consists of homogeneous Turkish, the migration rate is low. Most locals grow nut plants and others are fishermen. The mean GDP per capita in was US$ 6,500 in 2017. People usually consume meat (beef) and seafood. The mean duration of education is 6.4 years. A few tourists visit Akçakoca in the summer for a holiday. There are no official reports of air pollution causing health problems. According to the 2016 data, 24,161 people live and there are a total of 7 neighborhoods in Akçakoca [9].

### 2.2. Sample Size Calculation

The population of ≥45 age group was 14,332. Sample size was calculated as 3,383 persons with a 98% confidence level and a ± 2% margin of error. Since we did not know the exact prevalence, we took it as 0,5 to find out maximum sample size. We added a reserve of more than 500 and we aimed to screen 3920 individuals. Proportional samples were determined from the 7 neighborhoods with simple random sampling. Home visits were performed at each neighborhood until specified participant numbers were reached.

### 2.3. Data Collection

We used the research protocol developed by WHO for developing countries as in our previous studies [10]. The questionnaire which was prepared and validated (sensitivity 85.7%, specify 99%) in our previous PhD study (Şile) was used again. The questionnaire consisted of the following questions: participants were asked whether they had been diagnosed with cerebrovascular disorders by a doctor; whether or not they needed help with day to day activities; whether or not they had had a stroke in the last six months. Stroke was defined as neurological symptoms lasted ≥24 hours. Risk factors were asked in the second section: the presence of a high blood pressure diagnosis by a doctor, the presence of a diabetes mellitus diagnosis by a doctor, the presence of coronary heart disease or other heart diseases diagnosed by a doctor, the presence of dyslipidemia diagnosis by a doctor, whether or not they consume alcohol more than once a week, and whether or not they are currently a smoker.

The study was completed according to the answers given by the participants. Prevalence values, risk factors, and stroke types were determined. 15 surveyors making up 3 groups, consisting of a neurology assistant, a local nurse, and 3 surveyors, were put together. On the first day, the groups were trained by a health specialist and neurologist. The survey was a door-to-door and face-to-face one. Participants who claimed to have had a stroke diagnosed by a doctor were reexamined on site by a neurology assistant. All medical records and documents were reviewed and recorded. In the event the participants were unable to express themselves, a relative answer in their place. If nobody was home, the same home was revisited the following day. The survey was carried out between 1 March and 30 May 2017.

### 2.4. Statistical Analysis

Statistical analysis was performed using PASW Statistics 18.0 software. Frequency distribution, percentage, and mean and standard deviation were calculated where appropriate.

## 3. Results

In total, 3920 persons were reached. 3750 persons agreed to participate in the survey. Participation rate was 95%. Of the 3750 persons screened, the mean age was 56.4 ± 10.2. 1914 (51%) were male; 1836 (49%) were female. Demographic characteristics of participants are shown in Table 1.

83 persons had suffered a stroke. The crude prevalence rate was found to be 2.2%. Their mean age was 66.3±10.8. 43 (51.8%) were male; 40 (48.2%) were female. Male/female ratio was 1.1. Of the 83 stroke patients, 70 (84.3%) were ischemic stroke, 12 (14.5%) were hemorrhagic, and 1 (1.2%) was an unknown type. Demographic and clinical characteristics of stroke patients are shown in Table 2. The crude prevalence rate of stroke in Akçakoca according to age distribution is shown in Table 3.

## 4. Risk Factors

Among the population screened 1353 (36.1%) had hypertension, 645 (17.2%) had diabetes mellitus, 429 (11.4%) had heart disease, 507 (13.5%) had dyslipidemia, 583 (15.5%) were current smokers, and 147 (3.9%) were current alcohol consumers. Risk factors of participants are summarized in Table 4.

Among stroke patients, 71 (85.5%) had hypertension, 28 (33.7%) had diabetes mellitus, 40 (48.2%) had dyslipidemia, 39 (47%) had heart disease, 22 (26.5%) were current smokers, and 2 (2.4%) were current alcohol consumers. Risk factors of patients are summarized in Table 5. Proportion of stroke patients with multiple risk factors is shown in Table 6.

## 5. Discussion

The results of our study showed a 2.2% prevalence rate of stroke of in the Turkish population living in a rural area.

The previous two studies from Turkey were carried out in urban areas. One of them was conducted in 2011 in Denizli city (Aegean region) and the other in 2014 in Karabük (Black Sea region). Only one unpublished stroke prevalence study was conducted in a rural area in Şile at the same (Black Sea) region. The study indicated that the stroke prevalence rate among those ≥45 years was 2.9%. Both areas are located at the same latitude and in the Black Sea region. The racial, geographic, and socioeconomic characteristics are similar.

The prevalence of stroke in Denizli was 0.9% while the prevalence of stroke in Karabük was found to be 4.1% [7, 8]. When compared to these results our prevalence rate falls in the middle.

Denizli prevalence rate is significantly lower than our results. The two previous studies were conducted with same methods at the same age group (≥45) and on the Turkish population. However, these results recorded very different figures. Diet, lifestyle, and our unknown factors may be responsible for these remarkable differences. It is well known that ischemic stroke had a statistically significant inverse association with the Mediterranean diet [11]. Also, there are six years between the two studies.

Our previous study recorded a prevalence rate of 4.1% in Karabük. Dietary habits of two cities are similar. Both cities mainly consume meat and seafood. A possible reason for this high figure may be due to air pollution produced by the iron-and-steel factory located next to the city. The factory has been reported to produce air pollution including particulate matters for a long time. These pollutants are 4-11 time higher above the WHO threshold [12]. In addition, a study recorded very high level of trace elements in local lichens in Karabük district when compared to control area [13]. Akçakoca is officially reported not to have any air pollution by mobile stations of National Air Pollution Monitoring Network System (pollutant levels; PM_10_ < 20 *μ*g/m^3^, SO_2_ < 20 *μ*g/m^3^, NO_2_ < 40 *μ*g/m^3^); thus there is no permanent observation station in Akçakoca.

Some studies have already shown the link between environmental factors and cerebrovascular disorders. In one particular study, the correlation between high PM2.5 levels and an increased in stroke risk was seen to be direct. In addition, a strong relationship between exposure to black carbon and NO2, markers of traffic pollution, and stroke was recorded to be significant. These results are in agreement with past studies suggesting that traffic pollution may possibly cause ischemic strokes [14–17].

It is impossible to compare our results with those of others in Turkey and our neighboring countries because data is very limited.

Male-female ratio in our studies was found to be almost identical. However, especially in western countries, the male rate is generally higher than the female [18–20]. Our previous study also recorded a similar result [8]. The equality in the male-female ratio may be down to genetic and other unknown factors.

We found a high prevalence rate of hypertension as a risk factor. This was also reported in our previous study in Karabük [8].

## 6. Strengths and Limitations of the Study

One of the strengths of this study is that it was carried out door-to-door under the observation of a neurology assistant and a local nurse. A further strength is the high participation rate. The diagnosis of patients was confirmed by examination carried out by an assistant neurologist on site and laboratory findings and medical records were reevaluated. A possible limitation of this study is that some participants may have held back important information regarding their diagnosis.

## 7. Conclusion

The study is the first rural area based study from Turkey indicating the stroke prevalence rate among ≥45 years. Further studies are required to understand the real prevalence rates of the Turkish population.

## Figures and Tables

**Table 1:** Demographic characteristics of participants.

**Participant number**	3750
**Refused to participate**	170
**Participation rate**	95%
**Mean age, SD**	56.4 ± 10.2
**Male/Female**	1914/1836

**Table 2:** Demographic and clinical characteristics of stroke patients.

**Stroke cases**	83
**Crude prevalence rate**	2.2%
**Mean age of patients, SD**	66.3±10.8
**Male/female ratio**	1.1
**Ischemic stroke**	84.3%
**Hemorrhagic stroke**	14.5%

**Table 3:** Prevalence rate of stroke according age.

	**Population**	**Case**	**PR**
45-54	1899	11	0.6
55-64	1069	24	2.2
65-74	485	28	5.8
75-84	250	16	6.4
≥85	47	4	8.5
**All age**	**3750**	**83**	**2.2**

PR; prevalence rate %

**Table 4:** Risk factors of participants.

**Hypertension**	1353 (36.1%)
**Diabetes mellitus**	429 (11.4%)
**Dyslipidemia**	507 (13.5%)
**Current smokers**	583 (15.5%)
**Current alcohol consumers**	147 (3.9%)

**Table 5:** Risk factors of stroke patients.

**Hypertension**	71 (85.5%)
**Diabetes mellitus**	28 (33.7%)
**Dyslipidemia**	40 (48.2%)
**Heart Disease**	39 (47%)
**Current smokers**	22 (26.5%)
**Current alcohol consumers**	2 (2.4%)

**Table 6:** Proportion of stroke patients with multiple risk factors.

**Number of risk factors**	**0**	**1**	**2**	**3**	**4**	**5**	**6**
Number of patients	3	21	23	16	13	7	0

## Data Availability

The database used to support the findings of this study are included within the supplementary information file.

## Supplementary Materials

Supplementary Materials The data provided in the supplementary material file is the PDF format of SPSS database including all participants' flow.
