# Supplementary material for: Stroke Prevalence in a Coastal Town on the Black Sea Coast in Turkey: Community Based Study
Source: Neurol Res Int. 2018 Jul 2;2018:8246123. doi: 10.1155/2018/8246123 (PMC6051058; doi:10.1155/2018/8246123)
Supplement: Supplementary Materials — The data provided in the supplementary material file is the PDF format of SPSS database including all participants' flow. [file 8246123.f1.pdf]

yas	cinsiyet	tansiyon	diabet	kalp	kolesterol	sigara	sigmiktar	birakyıl
63,00	2,00	1,00	2,00	2,00	1,00	2,00	#NULL!	#NULL!
66,00	1,00	2,00	2,00	2,00	2,00	2,00	#NULL!	#NULL!
58,00	1,00	2,00	2,00	1,00	1,00	1,00	47,00	#NULL!
56,00	2,00	2,00	2,00	2,00	1,00	2,00	#NULL!	#NULL!
46,00	2,00	2,00	2,00	2,00	2,00	2,00	#NULL!	#NULL!
52,00	2,00	2,00	2,00	1,00	1,00	2,00	#NULL!	#NULL!
57,00	2,00	1,00	2,00	2,00	2,00	2,00	#NULL!	#NULL!
62,00	1,00	2,00	2,00	1,00	2,00	1,00	42,00	#NULL!
48,00	1,00	2,00	2,00	2,00	2,00	2,00	#NULL!	#NULL!
65,00	1,00	1,00	1,00	2,00	1,00	2,00	#NULL!	#NULL!
71,00	2,00	1,00	2,00	1,00	1,00	2,00	#NULL!	#NULL!
49,00	2,00	2,00	2,00	2,00	2,00	2,00	#NULL!	#NULL!
51,00	2,00	1,00	1,00	1,00	2,00	1,00	15,00	#NULL!
66,00	2,00	1,00	1,00	2,00	2,00	1,00	15,00	#NULL!
65,00	2,00	2,00	2,00	2,00	1,00	2,00	#NULL!	#NULL!
65,00	2,00	1,00	1,00	2,00	2,00	2,00	#NULL!	#NULL!
46,00	2,00	2,00	2,00	2,00	2,00	2,00	#NULL!	#NULL!
45,00	2,00	2,00	2,00	2,00	2,00	1,00	7,00	#NULL!
56,00	2,00	2,00	1,00	2,00	1,00	2,00	#NULL!	#NULL!
59,00	1,00	2,00	2,00	2,00	2,00	2,00	#NULL!	#NULL!
74,00	2,00	1,00	1,00	2,00	2,00	2,00	#NULL!	#NULL!
49,00	2,00	2,00	2,00	2,00	2,00	1,00	10,00	#NULL!
56,00	2,00	1,00	1,00	2,00	1,00	2,00	2,00	#NULL!
75,00	1,00	1,00	1,00	2,00	1,00	2,00	#NULL!	#NULL!
51,00	2,00	1,00	2,00	2,00	2,00	1,00	30,00	#NULL!
52,00	1,00	1,00	2,00	2,00	2,00	1,00	30,00	#NULL!
49,00	2,00	2,00	2,00	2,00	2,00	2,00	#NULL!	#NULL!
50,00	1,00	1,00	2,00	2,00	2,00	1,00	25,00	#NULL!
53,00	2,00	2,00	2,00	2,00	2,00	1,00	10,00	#NULL!
54,00	2,00	2,00	2,00	2,00	2,00	2,00	#NULL!	#NULL!
50,00	2,00	1,00	1,00	1,00	2,00	2,00	#NULL!	#NULL!
50,00	2,00	2,00	2,00	2,00	2,00	2,00	#NULL!	#NULL!
46,00	2,00	1,00	1,00	2,00	1,00	2,00	#NULL!	#NULL!
48,00	1,00	2,00	2,00	2,00	2,00	2,00	#NULL!	#NULL!
47,00	1,00	2,00	2,00	2,00	2,00	2,00	#NULL!	#NULL!
52,00	1,00	2,00	2,00	2,00	2,00	2,00	#NULL!	#NULL!
69,00	1,00	1,00	1,00	2,00	2,00	2,00	#NULL!	#NULL!
60,00	2,00	1,00	2,00	2,00	2,00	2,00	#NULL!	#NULL!
69,00	2,00	1,00	2,00	1,00	1,00	2,00	#NULL!	#NULL!
55,00	1,00	2,00	2,00	2,00	1,00	2,00	#NULL!	#NULL!
60,00	2,00	1,00	1,00	2,00	1,00	2,00	#NULL!	#NULL!
63,00	1,00	1,00	2,00	2,00	2,00	1,00	20,00	#NULL!
48,00	2,00	1,00	1,00	2,00	1,00	2,00	#NULL!	#NULL!
84,00	2,00	1,00	1,00	1,00	2,00	2,00	#NULL!	#NULL!
68,00	2,00	1,00	2,00	2,00	2,00	2,00	#NULL!	#NULL!
68,00	2,00	2,00	2,00	2,00	1,00	2,00	#NULL!	#NULL!
70,00	2,00	1,00	1,00	1,00	1,00	2,00	#NULL!	#NULL!
65,00	2,00	1,00	2,00	2,00	1,00	2,00	#NULL!	#NULL!
64,00	1,00	1,00	2,00	2,00	2,00	1,00	30,00	#NULL!

61,00	2,00	2,00	2,00	2,00	2,00	2,00	#NULL!	#NULL!
56,00	1,00	1,00	2,00	2,00	2,00	2,00	#NULL!	#NULL!
53,00	1,00	2,00	2,00	2,00	2,00	2,00	#NULL!	#NULL!
53,00	2,00	1,00	2,00	2,00	2,00	2,00	#NULL!	#NULL!
54,00	1,00	2,00	2,00	2,00	2,00	2,00	#NULL!	#NULL!
63,00	2,00	1,00	2,00	2,00	2,00	2,00	#NULL!	#NULL!
54,00	2,00	2,00	2,00	2,00	2,00	2,00	#NULL!	#NULL!
75,00	1,00	2,00	2,00	2,00	1,00	1,00	50,00	#NULL!
50,00	2,00	1,00	2,00	2,00	2,00	2,00	#NULL!	#NULL!
75,00	2,00	1,00	1,00	1,00	1,00	2,00	#NULL!	#NULL!
53,00	1,00	1,00	1,00	2,00	1,00	2,00	#NULL!	#NULL!
54,00	1,00	1,00	1,00	2,00	2,00	2,00	#NULL!	#NULL!
63,00	1,00	1,00	1,00	2,00	2,00	2,00	#NULL!	#NULL!
53,00	2,00	1,00	1,00	2,00	1,00	2,00	#NULL!	#NULL!
59,00	1,00	1,00	1,00	2,00	1,00	2,00	#NULL!	#NULL!
59,00	1,00	1,00	2,00	2,00	2,00	2,00	#NULL!	#NULL!
67,00	1,00	1,00	2,00	2,00	1,00	1,00	40,00	#NULL!
68,00	1,00	1,00	1,00	2,00	2,00	2,00	#NULL!	#NULL!
46,00	1,00	2,00	2,00	2,00	2,00	1,00	15,00	#NULL!
53,00	1,00	1,00	2,00	2,00	2,00	1,00	18,00	#NULL!
47,00	1,00	2,00	2,00	2,00	2,00	2,00	#NULL!	#NULL!
48,00	2,00	2,00	2,00	2,00	2,00	2,00	#NULL!	#NULL!
52,00	2,00	1,00	2,00	2,00	2,00	1,00	40,00	#NULL!
60,00	2,00	1,00	2,00	2,00	2,00	2,00	#NULL!	#NULL!
48,00	1,00	1,00	2,00	2,00	1,00	1,00	20,00	#NULL!
61,00	1,00	1,00	2,00	1,00	1,00	2,00	#NULL!	#NULL!
56,00	1,00	2,00	2,00	2,00	2,00	1,00	32,00	#NULL!
65,00	1,00	2,00	1,00	2,00	2,00	2,00	#NULL!	#NULL!
60,00	1,00	2,00	2,00	2,00	2,00	2,00	#NULL!	#NULL!
76,00	1,00	1,00	2,00	2,00	2,00	2,00	#NULL!	#NULL!
67,00	2,00	1,00	1,00	2,00	1,00	2,00	#NULL!	#NULL!
57,00	2,00	1,00	1,00	1,00	1,00	2,00	#NULL!	#NULL!
53,00	1,00	2,00	2,00	2,00	2,00	1,00	26,00	#NULL!
48,00	2,00	2,00	2,00	2,00	2,00	2,00	#NULL!	#NULL!
58,00	1,00	1,00	1,00	2,00	2,00	2,00	#NULL!	#NULL!
56,00	2,00	1,00	2,00	2,00	2,00	2,00	#NULL!	#NULL!
52,00	1,00	1,00	1,00	2,00	2,00	2,00	#NULL!	#NULL!
46,00	1,00	2,00	2,00	2,00	2,00	2,00	#NULL!	#NULL!
67,00	1,00	1,00	1,00	1,00	1,00	2,00	#NULL!	#NULL!
68,00	1,00	1,00	2,00	2,00	1,00	2,00	#NULL!	#NULL!
46,00	1,00	1,00	2,00	2,00	2,00	2,00	#NULL!	#NULL!
77,00	1,00	2,00	1,00	2,00	2,00	2,00	#NULL!	#NULL!
58,00	1,00	1,00	2,00	2,00	2,00	2,00	#NULL!	#NULL!
48,00	1,00	2,00	2,00	2,00	2,00	2,00	#NULL!	#NULL!
53,00	2,00	2,00	2,00	2,00	2,00	1,00	10,00	#NULL!
51,00	1,00	2,00	2,00	2,00	2,00	1,00	25,00	#NULL!
70,00	1,00	1,00	1,00	1,00	1,00	2,00	#NULL!	#NULL!
67,00	2,00	2,00	1,00	2,00	2,00	2,00	#NULL!	#NULL!
57,00	2,00	1,00	2,00	2,00	2,00	2,00	#NULL!	#NULL!
65,00	2,00	1,00	2,00	2,00	1,00	2,00	#NULL!	#NULL!

51,00	2,00	1,00	2,00	2,00	1,00	2,00	#NULL!	#NULL!
52,00	2,00	1,00	2,00	2,00	1,00	2,00	#NULL!	#NULL!
60,00	2,00	2,00	2,00	2,00	2,00	2,00	#NULL!	#NULL!
61,00	2,00	2,00	1,00	2,00	2,00	2,00	#NULL!	#NULL!
78,00	2,00	1,00	2,00	2,00	2,00	2,00	#NULL!	#NULL!
50,00	2,00	2,00	2,00	2,00	2,00	2,00	#NULL!	#NULL!
58,00	1,00	1,00	2,00	2,00	2,00	1,00	30,00	#NULL!
58,00	1,00	2,00	1,00	2,00	2,00	1,00	20,00	#NULL!
56,00	2,00	1,00	1,00	1,00	2,00	1,00	10,00	#NULL!
65,00	1,00	1,00	2,00	1,00	1,00	2,00	#NULL!	#NULL!
63,00	1,00	1,00	2,00	2,00	2,00	2,00	#NULL!	#NULL!
65,00	1,00	1,00	1,00	2,00	2,00	1,00	40,00	#NULL!
50,00	1,00	1,00	1,00	2,00	2,00	2,00	#NULL!	#NULL!
73,00	2,00	2,00	2,00	2,00	2,00	2,00	#NULL!	#NULL!
80,00	1,00	2,00	2,00	2,00	2,00	2,00	#NULL!	#NULL!
75,00	2,00	1,00	2,00	1,00	1,00	2,00	#NULL!	#NULL!
62,00	1,00	1,00	2,00	2,00	2,00	2,00	#NULL!	#NULL!
54,00	2,00	2,00	2,00	2,00	2,00	1,00	20,00	#NULL!
53,00	1,00	2,00	2,00	2,00	1,00	2,00	#NULL!	#NULL!
59,00	2,00	1,00	1,00	2,00	2,00	2,00	#NULL!	#NULL!
70,00	1,00	1,00	1,00	2,00	2,00	2,00	#NULL!	#NULL!
56,00	1,00	2,00	2,00	2,00	2,00	2,00	#NULL!	#NULL!
49,00	2,00	1,00	2,00	2,00	2,00	2,00	#NULL!	#NULL!
68,00	2,00	1,00	1,00	2,00	2,00	2,00	#NULL!	#NULL!
76,00	1,00	1,00	1,00	1,00	2,00	2,00	#NULL!	#NULL!
55,00	2,00	2,00	2,00	2,00	2,00	2,00	#NULL!	#NULL!
54,00	1,00	2,00	2,00	2,00	2,00	2,00	#NULL!	#NULL!
65,00	1,00	1,00	2,00	2,00	2,00	1,00	40,00	#NULL!
85,00	2,00	2,00	2,00	2,00	2,00	2,00	#NULL!	#NULL!
45,00	2,00	2,00	2,00	2,00	2,00	1,00	13,00	#NULL!
49,00	1,00	2,00	2,00	2,00	2,00	1,00	20,00	#NULL!
47,00	2,00	2,00	2,00	2,00	2,00	1,00	10,00	#NULL!
52,00	1,00	1,00	2,00	2,00	1,00	2,00	#NULL!	#NULL!
66,00	1,00	1,00	2,00	1,00	2,00	1,00	25,00	#NULL!
61,00	2,00	1,00	2,00	2,00	2,00	2,00	#NULL!	#NULL!
52,00	2,00	1,00	2,00	2,00	1,00	2,00	#NULL!	#NULL!
52,00	1,00	2,00	2,00	2,00	2,00	2,00	#NULL!	#NULL!
48,00	2,00	2,00	2,00	2,00	2,00	2,00	#NULL!	#NULL!
54,00	1,00	2,00	2,00	2,00	2,00	2,00	#NULL!	#NULL!
58,00	2,00	2,00	2,00	2,00	2,00	2,00	#NULL!	#NULL!
59,00	2,00	2,00	2,00	2,00	2,00	2,00	#NULL!	#NULL!
82,00	1,00	1,00	2,00	2,00	2,00	2,00	#NULL!	#NULL!
69,00	1,00	2,00	1,00	1,00	2,00	2,00	#NULL!	#NULL!
52,00	1,00	2,00	2,00	2,00	2,00	2,00	#NULL!	#NULL!
57,00	2,00	2,00	2,00	2,00	2,00	2,00	#NULL!	#NULL!
62,00	1,00	1,00	2,00	2,00	2,00	2,00	#NULL!	#NULL!
50,00	2,00	1,00	2,00	2,00	2,00	2,00	#NULL!	#NULL!
55,00	1,00	2,00	2,00	2,00	2,00	2,00	#NULL!	#NULL!
57,00	1,00	1,00	1,00	2,00	2,00	2,00	#NULL!	#NULL!
55,00	2,00	2,00	2,00	2,00	2,00	2,00	#NULL!	#NULL!

77,00	2,00	1,00	2,00	2,00	2,00	1,00	67,00	#NULL!
61,00	1,00	2,00	2,00	2,00	1,00	2,00	#NULL!	#NULL!
47,00	1,00	2,00	2,00	2,00	2,00	2,00	#NULL!	#NULL!
50,00	2,00	2,00	2,00	2,00	1,00	2,00	#NULL!	#NULL!
62,00	1,00	2,00	2,00	1,00	1,00	2,00	#NULL!	#NULL!
84,00	2,00	1,00	2,00	1,00	1,00	2,00	#NULL!	#NULL!
52,00	2,00	2,00	2,00	2,00	2,00	2,00	#NULL!	#NULL!
45,00	2,00	1,00	1,00	2,00	2,00	2,00	#NULL!	#NULL!
48,00	2,00	2,00	2,00	2,00	2,00	2,00	#NULL!	#NULL!
59,00	2,00	1,00	2,00	1,00	2,00	2,00	#NULL!	#NULL!
52,00	1,00	1,00	2,00	2,00	2,00	2,00	#NULL!	#NULL!
48,00	2,00	2,00	2,00	2,00	2,00	2,00	#NULL!	#NULL!
64,00	1,00	1,00	2,00	1,00	2,00	2,00	#NULL!	#NULL!
48,00	1,00	2,00	2,00	2,00	2,00	2,00	#NULL!	#NULL!
66,00	1,00	2,00	2,00	2,00	2,00	2,00	29,00	10,00
56,00	2,00	1,00	2,00	2,00	2,00	2,00	#NULL!	#NULL!
60,00	1,00	1,00	1,00	2,00	2,00	2,00	#NULL!	#NULL!
60,00	2,00	1,00	2,00	2,00	2,00	2,00	#NULL!	#NULL!
50,00	2,00	2,00	2,00	2,00	2,00	1,00	10,00	#NULL!
52,00	1,00	2,00	2,00	2,00	2,00	1,00	15,00	#NULL!
75,00	1,00	2,00	1,00	1,00	2,00	2,00	#NULL!	#NULL!
57,00	2,00	2,00	2,00	2,00	2,00	2,00	#NULL!	#NULL!
80,00	2,00	1,00	2,00	2,00	2,00	2,00	#NULL!	#NULL!
90,00	1,00	1,00	1,00	1,00	1,00	2,00	#NULL!	#NULL!
40,00	2,00	2,00	2,00	2,00	2,00	2,00	#NULL!	#NULL!
54,00	1,00	1,00	2,00	2,00	2,00	1,00	24,00	#NULL!
81,00	1,00	2,00	2,00	2,00	2,00	2,00	25,00	21,00
80,00	2,00	2,00	2,00	2,00	2,00	2,00	#NULL!	#NULL!
64,00	1,00	2,00	2,00	1,00	2,00	1,00	34,00	#NULL!
67,00	1,00	2,00	2,00	1,00	1,00	2,00	#NULL!	#NULL!
53,00	1,00	1,00	1,00	2,00	2,00	1,00	35,00	#NULL!
84,00	2,00	1,00	1,00	2,00	2,00	2,00	#NULL!	#NULL!
58,00	2,00	1,00	1,00	2,00	2,00	1,00	#NULL!	#NULL!
53,00	2,00	1,00	2,00	1,00	2,00	1,00	20,00	#NULL!
60,00	1,00	1,00	2,00	1,00	2,00	1,00	40,00	#NULL!
61,00	1,00	2,00	2,00	2,00	2,00	1,00	15,00	#NULL!
48,00	2,00	1,00	2,00	2,00	2,00	2,00	#NULL!	#NULL!
61,00	1,00	1,00	1,00	2,00	1,00	1,00	47,00	#NULL!
45,00	1,00	2,00	2,00	2,00	2,00	2,00	#NULL!	#NULL!
56,00	2,00	1,00	1,00	1,00	1,00	2,00	#NULL!	#NULL!
65,00	2,00	2,00	1,00	2,00	2,00	2,00	#NULL!	#NULL!
67,00	1,00	2,00	1,00	1,00	1,00	2,00	#NULL!	#NULL!
78,00	2,00	2,00	2,00	2,00	2,00	2,00	#NULL!	#NULL!
51,00	1,00	2,00	2,00	2,00	2,00	2,00	#NULL!	#NULL!
53,00	2,00	2,00	2,00	2,00	2,00	2,00	#NULL!	#NULL!
46,00	1,00	2,00	2,00	2,00	2,00	2,00	#NULL!	#NULL!
46,00	2,00	2,00	2,00	2,00	2,00	2,00	#NULL!	#NULL!
51,00	2,00	2,00	2,00	2,00	2,00	1,00	10,00	#NULL!
56,00	1,00	2,00	2,00	2,00	2,00	2,00	#NULL!	#NULL!
47,00	2,00	2,00	2,00	2,00	2,00	2,00	#NULL!	#NULL!

48,00	2,00	2,00	2,00	2,00	2,00	2,00	#NULL!	#NULL!
56,00	2,00	1,00	1,00	1,00	2,00	2,00	#NULL!	#NULL!
66,00	2,00	1,00	2,00	1,00	2,00	2,00	#NULL!	#NULL!
67,00	2,00	1,00	1,00	2,00	1,00	2,00	#NULL!	#NULL!
75,00	1,00	2,00	2,00	2,00	2,00	2,00	#NULL!	#NULL!
90,00	2,00	1,00	2,00	2,00	2,00	2,00	#NULL!	#NULL!
60,00	2,00	2,00	2,00	2,00	2,00	2,00	#NULL!	#NULL!
46,00	2,00	1,00	1,00	1,00	2,00	2,00	#NULL!	#NULL!
49,00	1,00	2,00	2,00	2,00	2,00	1,00	30,00	#NULL!
56,00	2,00	1,00	1,00	2,00	1,00	1,00	10,00	#NULL!
61,00	1,00	2,00	1,00	2,00	2,00	1,00	50,00	#NULL!
54,00	2,00	2,00	2,00	2,00	2,00	2,00	#NULL!	#NULL!
63,00	1,00	2,00	2,00	2,00	2,00	1,00	50,00	#NULL!
55,00	2,00	1,00	2,00	2,00	1,00	1,00	10,00	#NULL!
55,00	1,00	1,00	1,00	2,00	1,00	2,00	#NULL!	#NULL!
66,00	2,00	1,00	2,00	2,00	2,00	1,00	15,00	#NULL!
46,00	2,00	2,00	2,00	2,00	2,00	2,00	#NULL!	#NULL!
83,00	1,00	1,00	2,00	1,00	1,00	1,00	180,00	#NULL!
58,00	1,00	1,00	2,00	2,00	2,00	2,00	2,00	#NULL!
47,00	1,00	1,00	2,00	2,00	1,00	2,00	#NULL!	#NULL!
52,00	1,00	2,00	1,00	2,00	1,00	2,00	#NULL!	#NULL!
53,00	2,00	2,00	2,00	2,00	2,00	2,00	#NULL!	#NULL!
73,00	1,00	2,00	2,00	1,00	2,00	2,00	#NULL!	#NULL!
61,00	1,00	1,00	2,00	2,00	2,00	2,00	#NULL!	#NULL!
50,00	1,00	2,00	2,00	2,00	2,00	1,00	15,00	#NULL!
52,00	1,00	2,00	2,00	2,00	2,00	2,00	#NULL!	#NULL!
46,00	1,00	2,00	2,00	2,00	2,00	2,00	#NULL!	#NULL!
59,00	1,00	2,00	2,00	1,00	2,00	1,00	30,00	#NULL!
52,00	1,00	2,00	2,00	2,00	2,00	2,00	#NULL!	#NULL!
47,00	1,00	2,00	2,00	2,00	2,00	2,00	#NULL!	#NULL!
51,00	1,00	2,00	2,00	2,00	2,00	2,00	#NULL!	#NULL!
51,00	2,00	1,00	2,00	1,00	2,00	1,00	66,00	#NULL!
60,00	1,00	1,00	1,00	1,00	1,00	2,00	#NULL!	#NULL!
45,00	2,00	2,00	2,00	2,00	2,00	2,00	#NULL!	#NULL!
45,00	1,00	2,00	2,00	2,00	2,00	2,00	#NULL!	#NULL!
52,00	1,00	2,00	2,00	2,00	2,00	2,00	#NULL!	#NULL!
48,00	1,00	2,00	2,00	2,00	2,00	2,00	#NULL!	#NULL!
62,00	2,00	2,00	2,00	2,00	2,00	2,00	#NULL!	#NULL!
48,00	1,00	2,00	2,00	2,00	2,00	2,00	#NULL!	#NULL!
46,00	1,00	2,00	2,00	2,00	2,00	2,00	#NULL!	#NULL!
47,00	1,00	2,00	2,00	2,00	2,00	2,00	#NULL!	#NULL!
50,00	2,00	2,00	2,00	2,00	2,00	2,00	#NULL!	#NULL!
73,00	1,00	2,00	2,00	1,00	2,00	2,00	#NULL!	#NULL!
70,00	2,00	2,00	2,00	2,00	1,00	2,00	#NULL!	#NULL!
70,00	2,00	2,00	2,00	2,00	2,00	2,00	#NULL!	#NULL!
70,00	1,00	1,00	1,00	1,00	1,00	2,00	#NULL!	#NULL!
70,00	1,00	2,00	2,00	1,00	1,00	2,00	#NULL!	#NULL!
74,00	1,00	1,00	1,00	2,00	2,00	2,00	#NULL!	#NULL!
48,00	1,00	2,00	1,00	2,00	2,00	1,00	20,00	#NULL!
72,00	1,00	2,00	2,00	2,00	2,00	2,00	#NULL!	#NULL!

49,00	1,00	2,00	2,00	2,00	2,00	2,00	#NULL!	#NULL!
58,00	2,00	1,00	1,00	2,00	1,00	2,00	#NULL!	#NULL!
62,00	2,00	1,00	1,00	1,00	1,00	2,00	#NULL!	#NULL!
55,00	1,00	2,00	2,00	2,00	2,00	2,00	#NULL!	#NULL!
48,00	2,00	2,00	2,00	2,00	2,00	2,00	#NULL!	#NULL!
52,00	2,00	1,00	2,00	2,00	2,00	2,00	#NULL!	#NULL!
60,00	2,00	2,00	2,00	2,00	2,00	2,00	#NULL!	#NULL!
67,00	2,00	1,00	2,00	2,00	2,00	2,00	#NULL!	#NULL!
45,00	1,00	1,00	2,00	2,00	2,00	2,00	#NULL!	#NULL!
48,00	1,00	2,00	2,00	2,00	2,00	2,00	10,00	15,00
70,00	2,00	2,00	2,00	2,00	2,00	2,00	#NULL!	#NULL!
70,00	1,00	1,00	2,00	2,00	2,00	2,00	#NULL!	#NULL!
75,00	1,00	2,00	2,00	2,00	2,00	2,00	#NULL!	#NULL!
65,00	2,00	1,00	2,00	2,00	2,00	2,00	#NULL!	#NULL!
56,00	2,00	1,00	1,00	2,00	2,00	2,00	#NULL!	#NULL!
45,00	2,00	2,00	2,00	2,00	2,00	2,00	#NULL!	#NULL!
47,00	2,00	2,00	2,00	2,00	2,00	2,00	#NULL!	#NULL!
46,00	1,00	2,00	2,00	2,00	2,00	2,00	#NULL!	#NULL!
45,00	1,00	2,00	2,00	2,00	2,00	2,00	#NULL!	#NULL!
48,00	1,00	2,00	2,00	2,00	2,00	2,00	#NULL!	#NULL!
46,00	2,00	2,00	2,00	2,00	2,00	2,00	#NULL!	#NULL!
49,00	1,00	2,00	2,00	2,00	2,00	1,00	22,00	#NULL!
46,00	1,00	2,00	2,00	2,00	2,00	1,00	18,00	#NULL!
45,00	2,00	2,00	2,00	2,00	2,00	1,00	8,00	#NULL!
71,00	2,00	2,00	2,00	2,00	2,00	2,00	#NULL!	#NULL!
78,00	2,00	1,00	2,00	2,00	2,00	2,00	#NULL!	#NULL!
80,00	1,00	2,00	2,00	2,00	2,00	2,00	#NULL!	#NULL!
60,00	1,00	2,00	2,00	2,00	2,00	2,00	#NULL!	#NULL!
78,00	1,00	2,00	2,00	2,00	2,00	2,00	#NULL!	#NULL!
55,00	1,00	2,00	2,00	2,00	2,00	2,00	#NULL!	#NULL!
49,00	2,00	2,00	2,00	2,00	2,00	2,00	#NULL!	#NULL!
68,00	2,00	1,00	2,00	2,00	2,00	1,00	40,00	#NULL!
50,00	1,00	2,00	2,00	2,00	2,00	1,00	20,00	#NULL!
53,00	1,00	2,00	2,00	1,00	1,00	1,00	30,00	#NULL!
83,00	1,00	1,00	1,00	1,00	1,00	2,00	#NULL!	#NULL!
65,00	2,00	1,00	1,00	2,00	2,00	2,00	#NULL!	#NULL!
61,00	2,00	2,00	2,00	2,00	2,00	2,00	#NULL!	#NULL!
64,00	2,00	1,00	1,00	2,00	1,00	2,00	#NULL!	#NULL!
61,00	1,00	1,00	2,00	2,00	1,00	1,00	43,00	#NULL!
50,00	2,00	1,00	1,00	2,00	1,00	2,00	#NULL!	#NULL!
91,00	2,00	1,00	2,00	2,00	2,00	2,00	#NULL!	#NULL!
53,00	2,00	2,00	2,00	2,00	2,00	2,00	#NULL!	#NULL!
60,00	1,00	1,00	2,00	2,00	1,00	2,00	#NULL!	#NULL!
53,00	2,00	2,00	2,00	2,00	2,00	1,00	25,00	#NULL!
46,00	2,00	2,00	2,00	2,00	1,00	2,00	#NULL!	#NULL!
65,00	1,00	2,00	1,00	2,00	2,00	2,00	#NULL!	#NULL!
69,00	1,00	1,00	1,00	2,00	2,00	2,00	#NULL!	#NULL!
58,00	1,00	2,00	2,00	2,00	2,00	1,00	26,00	#NULL!
48,00	2,00	2,00	1,00	2,00	2,00	2,00	#NULL!	#NULL!
69,00	2,00	1,00	2,00	2,00	2,00	2,00	#NULL!	#NULL!

48,00	2,00	2,00	2,00	2,00	2,00	2,00	#NULL!	#NULL!
81,00	2,00	1,00	2,00	2,00	1,00	2,00	#NULL!	#NULL!
82,00	1,00	2,00	1,00	2,00	2,00	2,00	#NULL!	#NULL!
45,00	1,00	2,00	2,00	2,00	2,00	1,00	15,00	#NULL!
60,00	1,00	1,00	2,00	2,00	2,00	1,00	30,00	#NULL!
48,00	1,00	1,00	2,00	2,00	2,00	1,00	18,00	#NULL!
57,00	1,00	2,00	2,00	2,00	2,00	1,00	22,00	#NULL!
65,00	1,00	2,00	2,00	2,00	2,00	2,00	#NULL!	#NULL!
55,00	2,00	1,00	2,00	2,00	1,00	2,00	#NULL!	#NULL!
45,00	2,00	2,00	2,00	2,00	2,00	2,00	#NULL!	#NULL!
47,00	1,00	2,00	2,00	2,00	2,00	2,00	#NULL!	#NULL!
67,00	2,00	2,00	2,00	2,00	2,00	2,00	#NULL!	#NULL!
71,00	1,00	2,00	1,00	1,00	2,00	1,00	40,00	2,00
73,00	1,00	1,00	2,00	2,00	2,00	2,00	#NULL!	#NULL!
55,00	1,00	2,00	2,00	2,00	2,00	1,00	10,00	#NULL!
48,00	2,00	1,00	2,00	2,00	2,00	2,00	#NULL!	#NULL!
51,00	1,00	2,00	1,00	2,00	2,00	2,00	#NULL!	#NULL!
61,00	1,00	2,00	2,00	2,00	2,00	1,00	10,00	#NULL!
70,00	2,00	1,00	2,00	2,00	1,00	2,00	#NULL!	#NULL!
77,00	1,00	1,00	2,00	1,00	1,00	2,00	#NULL!	#NULL!
55,00	2,00	2,00	2,00	2,00	2,00	1,00	20,00	#NULL!
52,00	2,00	2,00	2,00	2,00	2,00	1,00	19,00	#NULL!
70,00	2,00	2,00	2,00	2,00	2,00	2,00	#NULL!	#NULL!
52,00	2,00	1,00	1,00	2,00	2,00	2,00	#NULL!	#NULL!
78,00	1,00	2,00	2,00	2,00	1,00	2,00	#NULL!	#NULL!
50,00	1,00	1,00	2,00	2,00	2,00	2,00	#NULL!	#NULL!
45,00	2,00	2,00	2,00	2,00	2,00	1,00	5,00	#NULL!
47,00	2,00	1,00	2,00	2,00	2,00	2,00	#NULL!	#NULL!
52,00	1,00	2,00	1,00	2,00	2,00	2,00	#NULL!	#NULL!
54,00	2,00	2,00	2,00	2,00	2,00	2,00	#NULL!	#NULL!
56,00	1,00	2,00	2,00	2,00	2,00	2,00	#NULL!	#NULL!
67,00	2,00	1,00	2,00	2,00	1,00	2,00	#NULL!	#NULL!
75,00	1,00	2,00	2,00	2,00	2,00	2,00	#NULL!	#NULL!
45,00	1,00	2,00	2,00	2,00	2,00	2,00	#NULL!	#NULL!
47,00	1,00	2,00	2,00	2,00	2,00	2,00	#NULL!	#NULL!
48,00	1,00	2,00	2,00	2,00	1,00	1,00	10,00	#NULL!
50,00	1,00	2,00	2,00	2,00	2,00	1,00	15,00	#NULL!
56,00	1,00	2,00	2,00	2,00	2,00	1,00	20,00	#NULL!
66,00	2,00	2,00	1,00	2,00	2,00	2,00	#NULL!	#NULL!
69,00	1,00	1,00	2,00	2,00	2,00	2,00	#NULL!	#NULL!
65,00	2,00	2,00	1,00	2,00	2,00	2,00	#NULL!	#NULL!
46,00	1,00	2,00	2,00	2,00	2,00	1,00	20,00	#NULL!
49,00	1,00	2,00	2,00	2,00	2,00	1,00	30,00	#NULL!
45,00	1,00	2,00	2,00	2,00	2,00	1,00	15,00	#NULL!
48,00	1,00	2,00	2,00	2,00	2,00	1,00	5,00	#NULL!
52,00	2,00	2,00	2,00	2,00	2,00	2,00	#NULL!	#NULL!
45,00	1,00	1,00	1,00	2,00	1,00	1,00	10,00	#NULL!
51,00	1,00	1,00	2,00	2,00	2,00	1,00	30,00	#NULL!
45,00	2,00	2,00	2,00	2,00	2,00	2,00	#NULL!	#NULL!
59,00	1,00	2,00	2,00	2,00	2,00	2,00	#NULL!	#NULL!

65,00	2,00	1,00	1,00	2,00	2,00	2,00	#NULL!	#NULL!
60,00	2,00	2,00	2,00	2,00	2,00	2,00	#NULL!	#NULL!
54,00	1,00	2,00	2,00	2,00	2,00	1,00	30,00	#NULL!
52,00	1,00	1,00	1,00	2,00	1,00	2,00	#NULL!	#NULL!
50,00	2,00	2,00	2,00	2,00	2,00	2,00	#NULL!	#NULL!
45,00	1,00	2,00	2,00	2,00	2,00	1,00	40,00	#NULL!
46,00	1,00	2,00	2,00	1,00	1,00	2,00	#NULL!	#NULL!
47,00	1,00	1,00	1,00	2,00	2,00	1,00	28,00	#NULL!
45,00	2,00	1,00	1,00	2,00	2,00	2,00	#NULL!	#NULL!
51,00	1,00	1,00	1,00	2,00	2,00	2,00	#NULL!	#NULL!
51,00	2,00	1,00	1,00	2,00	2,00	2,00	#NULL!	#NULL!
54,00	1,00	2,00	2,00	2,00	2,00	2,00	#NULL!	#NULL!
56,00	2,00	2,00	2,00	2,00	2,00	2,00	#NULL!	#NULL!
45,00	1,00	2,00	2,00	2,00	2,00	2,00	#NULL!	#NULL!
45,00	2,00	2,00	2,00	2,00	2,00	2,00	#NULL!	#NULL!
52,00	2,00	2,00	2,00	2,00	2,00	2,00	#NULL!	#NULL!
52,00	1,00	2,00	2,00	2,00	2,00	2,00	#NULL!	#NULL!
55,00	1,00	1,00	1,00	2,00	2,00	2,00	#NULL!	#NULL!
53,00	2,00	2,00	2,00	2,00	2,00	2,00	#NULL!	#NULL!
53,00	1,00	2,00	2,00	2,00	2,00	1,00	15,00	#NULL!
50,00	1,00	2,00	2,00	2,00	2,00	2,00	#NULL!	#NULL!
55,00	1,00	1,00	1,00	1,00	2,00	1,00	10,00	#NULL!
50,00	2,00	2,00	2,00	2,00	2,00	2,00	#NULL!	#NULL!
53,00	1,00	1,00	1,00	2,00	2,00	2,00	#NULL!	#NULL!
50,00	2,00	1,00	1,00	2,00	2,00	2,00	#NULL!	#NULL!
50,00	2,00	1,00	1,00	2,00	2,00	1,00	30,00	#NULL!
58,00	1,00	2,00	2,00	2,00	2,00	2,00	#NULL!	#NULL!
55,00	2,00	2,00	2,00	2,00	2,00	2,00	#NULL!	#NULL!
59,00	1,00	2,00	2,00	2,00	2,00	2,00	#NULL!	#NULL!
55,00	1,00	2,00	2,00	2,00	2,00	2,00	#NULL!	#NULL!
60,00	2,00	2,00	2,00	2,00	2,00	2,00	#NULL!	#NULL!
56,00	1,00	1,00	1,00	2,00	2,00	1,00	10,00	#NULL!
54,00	2,00	1,00	1,00	2,00	2,00	1,00	10,00	#NULL!
55,00	2,00	2,00	2,00	2,00	2,00	2,00	#NULL!	#NULL!
56,00	1,00	2,00	2,00	2,00	2,00	2,00	#NULL!	#NULL!
50,00	1,00	1,00	1,00	2,00	2,00	2,00	#NULL!	#NULL!
47,00	2,00	1,00	1,00	2,00	2,00	2,00	#NULL!	#NULL!
50,00	1,00	2,00	2,00	2,00	2,00	1,00	10,00	#NULL!
49,00	2,00	2,00	2,00	2,00	2,00	2,00	#NULL!	#NULL!
50,00	2,00	1,00	1,00	2,00	2,00	1,00	20,00	#NULL!
55,00	1,00	1,00	1,00	2,00	2,00	1,00	20,00	#NULL!
50,00	2,00	1,00	1,00	2,00	2,00	2,00	#NULL!	#NULL!
49,00	1,00	1,00	1,00	2,00	1,00	1,00	12,00	#NULL!
45,00	2,00	2,00	2,00	2,00	2,00	2,00	#NULL!	#NULL!
48,00	1,00	2,00	2,00	2,00	2,00	2,00	#NULL!	#NULL!
48,00	2,00	1,00	2,00	2,00	2,00	2,00	#NULL!	#NULL!
49,00	1,00	2,00	2,00	2,00	2,00	2,00	#NULL!	#NULL!
75,00	2,00	2,00	2,00	2,00	2,00	2,00	#NULL!	#NULL!
78,00	1,00	1,00	2,00	2,00	2,00	2,00	#NULL!	#NULL!
49,00	2,00	2,00	2,00	2,00	2,00	2,00	#NULL!	#NULL!

69,00	1,00	2,00	2,00	2,00	2,00	2,00	#NULL!	#NULL!
45,00	1,00	2,00	2,00	2,00	2,00	2,00	#NULL!	#NULL!
56,00	1,00	1,00	2,00	2,00	2,00	1,00	9,00	#NULL!
49,00	1,00	2,00	2,00	2,00	2,00	1,00	30,00	#NULL!
55,00	1,00	2,00	2,00	2,00	2,00	2,00	#NULL!	#NULL!
52,00	1,00	2,00	2,00	2,00	2,00	2,00	#NULL!	#NULL!
45,00	2,00	2,00	2,00	2,00	2,00	2,00	#NULL!	#NULL!
70,00	2,00	2,00	2,00	2,00	2,00	2,00	#NULL!	#NULL!
60,00	2,00	1,00	2,00	2,00	2,00	2,00	#NULL!	#NULL!
68,00	1,00	2,00	2,00	2,00	2,00	2,00	#NULL!	#NULL!
60,00	2,00	2,00	2,00	2,00	2,00	2,00	#NULL!	#NULL!
47,00	2,00	2,00	2,00	2,00	2,00	2,00	#NULL!	#NULL!
48,00	1,00	2,00	2,00	2,00	2,00	2,00	#NULL!	#NULL!
49,00	1,00	1,00	1,00	2,00	2,00	1,00	30,00	#NULL!
48,00	2,00	1,00	1,00	2,00	2,00	1,00	26,00	#NULL!
50,00	1,00	1,00	1,00	2,00	2,00	2,00	#NULL!	#NULL!
45,00	2,00	1,00	1,00	2,00	2,00	2,00	#NULL!	#NULL!
55,00	2,00	2,00	2,00	2,00	2,00	2,00	#NULL!	#NULL!
53,00	1,00	2,00	2,00	2,00	2,00	2,00	#NULL!	#NULL!
50,00	2,00	1,00	1,00	2,00	2,00	2,00	#NULL!	#NULL!
49,00	1,00	1,00	1,00	2,00	2,00	2,00	#NULL!	#NULL!
82,00	2,00	1,00	2,00	2,00	2,00	2,00	#NULL!	#NULL!
67,00	2,00	2,00	2,00	2,00	2,00	2,00	#NULL!	#NULL!
46,00	1,00	2,00	2,00	2,00	2,00	2,00	#NULL!	#NULL!
74,00	2,00	2,00	2,00	2,00	2,00	2,00	#NULL!	#NULL!
48,00	1,00	2,00	2,00	2,00	2,00	2,00	#NULL!	#NULL!
49,00	2,00	2,00	2,00	2,00	2,00	2,00	#NULL!	#NULL!
46,00	2,00	2,00	2,00	2,00	2,00	1,00	40,00	#NULL!
45,00	2,00	2,00	2,00	2,00	2,00	1,00	30,00	#NULL!
49,00	1,00	2,00	2,00	2,00	2,00	2,00	#NULL!	#NULL!
45,00	2,00	2,00	2,00	2,00	2,00	2,00	#NULL!	#NULL!
45,00	1,00	2,00	2,00	2,00	2,00	2,00	#NULL!	#NULL!
48,00	1,00	2,00	2,00	2,00	2,00	2,00	#NULL!	#NULL!
45,00	2,00	2,00	2,00	2,00	2,00	2,00	#NULL!	#NULL!
47,00	1,00	2,00	2,00	2,00	2,00	2,00	#NULL!	#NULL!
45,00	2,00	2,00	2,00	2,00	2,00	1,00	20,00	#NULL!
46,00	1,00	2,00	2,00	2,00	2,00	2,00	#NULL!	#NULL!
75,00	2,00	2,00	2,00	2,00	2,00	2,00	#NULL!	#NULL!
48,00	2,00	2,00	2,00	2,00	2,00	2,00	#NULL!	#NULL!
75,00	1,00	2,00	2,00	1,00	2,00	2,00	#NULL!	#NULL!
75,00	1,00	1,00	1,00	2,00	1,00	2,00	#NULL!	#NULL!
45,00	2,00	2,00	2,00	2,00	2,00	2,00	#NULL!	#NULL!
46,00	1,00	2,00	2,00	2,00	2,00	2,00	#NULL!	#NULL!
55,00	1,00	2,00	2,00	2,00	2,00	2,00	#NULL!	#NULL!
45,00	2,00	2,00	2,00	2,00	2,00	2,00	#NULL!	#NULL!
82,00	1,00	1,00	1,00	2,00	2,00	2,00	#NULL!	#NULL!
61,00	2,00	1,00	2,00	2,00	2,00	2,00	#NULL!	#NULL!
65,00	1,00	1,00	2,00	2,00	2,00	2,00	#NULL!	#NULL!
62,00	1,00	1,00	2,00	2,00	2,00	2,00	#NULL!	#NULL!
46,00	1,00	2,00	2,00	2,00	2,00	2,00	#NULL!	#NULL!

45,00	2,00	2,00	2,00	2,00	2,00	2,00	#NULL!	#NULL!
45,00	1,00	2,00	2,00	2,00	2,00	2,00	#NULL!	#NULL!
45,00	2,00	2,00	2,00	2,00	2,00	2,00	#NULL!	#NULL!
46,00	1,00	2,00	2,00	2,00	2,00	1,00	20,00	#NULL!
45,00	2,00	2,00	2,00	2,00	2,00	2,00	#NULL!	#NULL!
53,00	2,00	2,00	2,00	2,00	2,00	2,00	#NULL!	#NULL!
45,00	1,00	2,00	2,00	2,00	2,00	2,00	#NULL!	#NULL!
52,00	1,00	2,00	2,00	2,00	1,00	2,00	#NULL!	#NULL!
46,00	1,00	2,00	2,00	2,00	2,00	1,00	18,00	#NULL!
55,00	1,00	2,00	2,00	2,00	2,00	2,00	#NULL!	#NULL!
53,00	1,00	2,00	2,00	2,00	2,00	2,00	#NULL!	#NULL!
45,00	1,00	2,00	2,00	2,00	2,00	2,00	#NULL!	#NULL!
51,00	2,00	2,00	2,00	2,00	2,00	2,00	#NULL!	#NULL!
45,00	2,00	2,00	2,00	2,00	2,00	1,00	5,00	#NULL!
45,00	1,00	2,00	2,00	2,00	2,00	2,00	#NULL!	#NULL!
52,00	2,00	2,00	2,00	2,00	2,00	2,00	#NULL!	#NULL!
52,00	2,00	2,00	2,00	2,00	2,00	1,00	13,00	#NULL!
55,00	1,00	2,00	2,00	2,00	2,00	2,00	#NULL!	#NULL!
50,00	1,00	2,00	1,00	2,00	2,00	2,00	#NULL!	#NULL!
55,00	1,00	2,00	1,00	2,00	2,00	2,00	#NULL!	#NULL!
50,00	1,00	2,00	2,00	2,00	2,00	2,00	#NULL!	#NULL!
55,00	1,00	2,00	2,00	2,00	2,00	2,00	#NULL!	#NULL!
53,00	1,00	2,00	2,00	2,00	2,00	2,00	#NULL!	#NULL!
48,00	1,00	2,00	2,00	2,00	2,00	2,00	#NULL!	#NULL!
52,00	1,00	2,00	2,00	2,00	2,00	2,00	#NULL!	#NULL!
50,00	1,00	2,00	2,00	2,00	2,00	2,00	#NULL!	#NULL!
45,00	2,00	2,00	2,00	2,00	2,00	2,00	#NULL!	#NULL!
45,00	1,00	2,00	2,00	2,00	1,00	2,00	#NULL!	#NULL!
50,00	1,00	2,00	1,00	2,00	2,00	1,00	30,00	#NULL!
45,00	2,00	2,00	2,00	2,00	2,00	2,00	#NULL!	#NULL!
45,00	2,00	2,00	2,00	2,00	2,00	2,00	#NULL!	#NULL!
55,00	1,00	2,00	2,00	2,00	2,00	2,00	#NULL!	#NULL!
50,00	1,00	2,00	2,00	2,00	2,00	2,00	#NULL!	#NULL!
50,00	2,00	2,00	2,00	2,00	2,00	2,00	#NULL!	#NULL!
55,00	2,00	2,00	2,00	2,00	2,00	2,00	#NULL!	#NULL!
45,00	2,00	2,00	2,00	2,00	2,00	2,00	#NULL!	#NULL!
45,00	2,00	1,00	2,00	2,00	2,00	1,00	10,00	#NULL!
50,00	1,00	2,00	2,00	2,00	2,00	2,00	#NULL!	#NULL!
45,00	2,00	2,00	2,00	2,00	2,00	2,00	#NULL!	#NULL!
45,00	2,00	2,00	2,00	2,00	2,00	2,00	#NULL!	#NULL!
50,00	1,00	2,00	2,00	2,00	2,00	2,00	#NULL!	#NULL!
50,00	1,00	2,00	2,00	2,00	2,00	2,00	#NULL!	#NULL!
45,00	1,00	2,00	2,00	2,00	2,00	2,00	#NULL!	#NULL!
45,00	2,00	2,00	2,00	2,00	2,00	2,00	#NULL!	#NULL!
53,00	1,00	2,00	2,00	2,00	2,00	2,00	#NULL!	#NULL!
55,00	1,00	2,00	2,00	2,00	2,00	2,00	#NULL!	#NULL!
50,00	2,00	2,00	2,00	2,00	2,00	2,00	#NULL!	#NULL!
71,00	1,00	2,00	2,00	2,00	2,00	2,00	#NULL!	#NULL!
60,00	2,00	2,00	2,00	2,00	2,00	2,00	#NULL!	#NULL!
53,00	1,00	2,00	2,00	2,00	2,00	2,00	#NULL!	#NULL!

45,00	2,00	2,00	2,00	2,00	2,00	2,00	#NULL!	#NULL!
45,00	2,00	2,00	2,00	2,00	2,00	1,00	20,00	#NULL!
55,00	2,00	2,00	2,00	2,00	2,00	1,00	#NULL!	#NULL!
53,00	1,00	2,00	2,00	2,00	2,00	1,00	40,00	#NULL!
45,00	2,00	1,00	2,00	2,00	2,00	2,00	#NULL!	#NULL!
50,00	1,00	2,00	2,00	2,00	2,00	2,00	#NULL!	#NULL!
45,00	1,00	2,00	2,00	2,00	2,00	1,00	15,00	#NULL!
45,00	2,00	2,00	2,00	2,00	2,00	2,00	#NULL!	#NULL!
55,00	1,00	1,00	2,00	2,00	2,00	2,00	#NULL!	#NULL!
50,00	1,00	2,00	2,00	2,00	2,00	2,00	#NULL!	#NULL!
55,00	2,00	2,00	1,00	2,00	1,00	2,00	#NULL!	#NULL!
50,00	2,00	2,00	2,00	2,00	2,00	2,00	#NULL!	#NULL!
55,00	1,00	2,00	1,00	2,00	2,00	2,00	#NULL!	#NULL!
65,00	1,00	2,00	2,00	2,00	1,00	2,00	#NULL!	#NULL!
50,00	2,00	2,00	2,00	2,00	2,00	1,00	10,00	#NULL!
55,00	1,00	1,00	2,00	2,00	2,00	2,00	#NULL!	#NULL!
55,00	2,00	2,00	1,00	2,00	2,00	2,00	#NULL!	#NULL!
66,00	2,00	1,00	1,00	2,00	2,00	2,00	#NULL!	#NULL!
45,00	1,00	2,00	2,00	2,00	2,00	2,00	#NULL!	#NULL!
66,00	2,00	2,00	2,00	2,00	2,00	2,00	#NULL!	#NULL!
45,00	2,00	2,00	2,00	2,00	2,00	2,00	#NULL!	#NULL!
50,00	2,00	2,00	2,00	2,00	2,00	2,00	#NULL!	#NULL!
45,00	1,00	2,00	2,00	2,00	2,00	2,00	#NULL!	#NULL!
78,00	2,00	1,00	2,00	2,00	2,00	2,00	#NULL!	#NULL!
58,00	1,00	1,00	2,00	2,00	2,00	2,00	#NULL!	#NULL!
43,00	2,00	2,00	2,00	2,00	2,00	1,00	30,00	#NULL!
65,00	1,00	1,00	2,00	2,00	2,00	2,00	#NULL!	#NULL!
49,00	1,00	1,00	2,00	2,00	2,00	2,00	#NULL!	#NULL!
60,00	1,00	2,00	2,00	2,00	2,00	2,00	#NULL!	#NULL!
49,00	2,00	2,00	2,00	2,00	2,00	2,00	#NULL!	#NULL!
49,00	1,00	2,00	2,00	2,00	2,00	2,00	#NULL!	#NULL!
69,00	2,00	1,00	1,00	1,00	2,00	2,00	#NULL!	#NULL!
48,00	2,00	2,00	2,00	2,00	2,00	2,00	#NULL!	#NULL!
49,00	1,00	1,00	2,00	2,00	2,00	2,00	#NULL!	#NULL!
49,00	1,00	2,00	2,00	2,00	2,00	2,00	#NULL!	#NULL!
48,00	2,00	2,00	2,00	2,00	2,00	2,00	#NULL!	#NULL!
46,00	2,00	2,00	2,00	2,00	2,00	2,00	#NULL!	#NULL!
52,00	1,00	2,00	2,00	2,00	2,00	1,00	15,00	#NULL!
51,00	1,00	2,00	2,00	2,00	2,00	2,00	#NULL!	#NULL!
56,00	2,00	2,00	2,00	2,00	2,00	1,00	20,00	#NULL!
50,00	1,00	2,00	2,00	2,00	2,00	2,00	#NULL!	#NULL!
57,00	2,00	2,00	2,00	2,00	2,00	1,00	15,00	#NULL!
56,00	1,00	2,00	2,00	2,00	2,00	2,00	#NULL!	#NULL!
50,00	2,00	2,00	2,00	2,00	2,00	2,00	#NULL!	#NULL!
56,00	2,00	2,00	2,00	2,00	2,00	2,00	#NULL!	#NULL!
51,00	2,00	2,00	2,00	2,00	2,00	2,00	#NULL!	#NULL!
54,00	1,00	2,00	2,00	2,00	2,00	2,00	#NULL!	#NULL!
46,00	2,00	2,00	2,00	2,00	2,00	2,00	#NULL!	#NULL!
52,00	1,00	2,00	2,00	2,00	2,00	2,00	#NULL!	#NULL!
46,00	1,00	1,00	1,00	2,00	2,00	1,00	18,00	#NULL!

49,00	1,00	1,00	2,00	2,00	1,00	2,00	#NULL!	#NULL!
45,00	1,00	2,00	2,00	2,00	2,00	2,00	#NULL!	#NULL!
46,00	2,00	2,00	2,00	2,00	2,00	2,00	#NULL!	#NULL!
46,00	1,00	2,00	2,00	2,00	1,00	2,00	#NULL!	#NULL!
56,00	2,00	2,00	2,00	2,00	2,00	2,00	#NULL!	#NULL!
46,00	1,00	2,00	2,00	2,00	2,00	2,00	#NULL!	#NULL!
55,00	1,00	2,00	2,00	2,00	2,00	1,00	20,00	#NULL!
46,00	1,00	2,00	2,00	2,00	2,00	2,00	#NULL!	#NULL!
48,00	2,00	2,00	2,00	2,00	2,00	2,00	#NULL!	#NULL!
52,00	1,00	2,00	1,00	2,00	2,00	2,00	#NULL!	#NULL!
45,00	2,00	2,00	2,00	2,00	2,00	2,00	#NULL!	#NULL!
46,00	1,00	2,00	2,00	2,00	2,00	2,00	#NULL!	#NULL!
48,00	1,00	2,00	1,00	2,00	2,00	2,00	#NULL!	#NULL!
50,00	1,00	2,00	2,00	2,00	2,00	2,00	#NULL!	#NULL!
45,00	1,00	2,00	2,00	2,00	2,00	2,00	#NULL!	#NULL!
45,00	1,00	2,00	2,00	2,00	2,00	2,00	#NULL!	#NULL!
46,00	1,00	2,00	2,00	2,00	2,00	2,00	#NULL!	#NULL!
48,00	1,00	2,00	2,00	2,00	2,00	2,00	#NULL!	#NULL!
45,00	1,00	2,00	2,00	2,00	2,00	2,00	#NULL!	#NULL!
46,00	1,00	2,00	2,00	1,00	2,00	2,00	#NULL!	#NULL!
45,00	2,00	2,00	2,00	2,00	2,00	2,00	#NULL!	#NULL!
49,00	1,00	2,00	2,00	2,00	2,00	1,00	10,00	#NULL!
48,00	2,00	2,00	2,00	2,00	2,00	2,00	#NULL!	#NULL!
49,00	2,00	2,00	2,00	2,00	2,00	2,00	#NULL!	#NULL!
59,00	2,00	1,00	2,00	2,00	2,00	2,00	#NULL!	#NULL!
71,00	2,00	1,00	1,00	1,00	2,00	2,00	#NULL!	#NULL!
45,00	1,00	2,00	2,00	2,00	2,00	2,00	#NULL!	#NULL!
45,00	2,00	2,00	1,00	2,00	2,00	2,00	#NULL!	#NULL!
50,00	1,00	2,00	2,00	2,00	2,00	2,00	#NULL!	#NULL!
45,00	2,00	2,00	2,00	2,00	2,00	2,00	#NULL!	#NULL!
46,00	1,00	2,00	2,00	2,00	2,00	2,00	#NULL!	#NULL!
48,00	1,00	1,00	2,00	1,00	2,00	2,00	#NULL!	#NULL!
52,00	1,00	1,00	1,00	1,00	1,00	2,00	#NULL!	#NULL!
51,00	2,00	1,00	2,00	2,00	2,00	2,00	#NULL!	#NULL!
52,00	1,00	1,00	1,00	2,00	2,00	2,00	#NULL!	#NULL!
48,00	2,00	2,00	2,00	2,00	2,00	2,00	#NULL!	#NULL!
49,00	1,00	1,00	2,00	2,00	2,00	1,00	25,00	#NULL!
49,00	2,00	2,00	2,00	2,00	2,00	2,00	#NULL!	#NULL!
51,00	1,00	1,00	1,00	2,00	2,00	2,00	#NULL!	#NULL!
49,00	2,00	1,00	2,00	2,00	2,00	2,00	#NULL!	#NULL!
50,00	1,00	1,00	2,00	2,00	2,00	2,00	#NULL!	#NULL!
47,00	2,00	2,00	2,00	2,00	2,00	2,00	#NULL!	#NULL!
67,00	2,00	2,00	2,00	2,00	2,00	2,00	#NULL!	#NULL!
78,00	2,00	2,00	2,00	2,00	2,00	2,00	#NULL!	#NULL!
49,00	2,00	1,00	2,00	2,00	2,00	2,00	#NULL!	#NULL!
48,00	1,00	2,00	1,00	2,00	2,00	1,00	10,00	#NULL!
50,00	1,00	2,00	2,00	2,00	2,00	1,00	20,00	#NULL!
45,00	2,00	2,00	2,00	2,00	2,00	1,00	12,00	#NULL!
50,00	1,00	2,00	2,00	2,00	2,00	2,00	#NULL!	#NULL!
48,00	2,00	1,00	2,00	2,00	2,00	1,00	8,00	#NULL!

49,00	1,00	2,00	2,00	2,00	2,00	2,00	#NULL!	#NULL!
48,00	2,00	1,00	2,00	2,00	2,00	2,00	#NULL!	#NULL!
50,00	1,00	2,00	2,00	2,00	2,00	2,00	#NULL!	#NULL!
50,00	1,00	1,00	1,00	2,00	2,00	1,00	20,00	#NULL!
53,00	2,00	1,00	1,00	2,00	2,00	1,00	20,00	#NULL!
52,00	1,00	1,00	1,00	2,00	2,00	1,00	24,00	#NULL!
54,00	2,00	1,00	1,00	2,00	2,00	1,00	20,00	#NULL!
52,00	1,00	2,00	2,00	2,00	2,00	2,00	#NULL!	#NULL!
48,00	2,00	2,00	2,00	2,00	2,00	2,00	#NULL!	#NULL!
56,00	1,00	1,00	1,00	2,00	2,00	2,00	#NULL!	#NULL!
55,00	2,00	1,00	1,00	2,00	2,00	2,00	#NULL!	#NULL!
50,00	1,00	2,00	2,00	2,00	2,00	1,00	25,00	#NULL!
60,00	1,00	2,00	2,00	2,00	2,00	2,00	#NULL!	#NULL!
60,00	1,00	2,00	2,00	2,00	2,00	2,00	#NULL!	#NULL!
45,00	1,00	2,00	2,00	2,00	2,00	2,00	#NULL!	#NULL!
47,00	1,00	1,00	1,00	2,00	2,00	2,00	#NULL!	#NULL!
47,00	2,00	1,00	1,00	2,00	2,00	2,00	#NULL!	#NULL!
45,00	1,00	1,00	1,00	2,00	2,00	2,00	#NULL!	#NULL!
45,00	2,00	1,00	1,00	2,00	2,00	2,00	#NULL!	#NULL!
57,00	1,00	1,00	1,00	2,00	2,00	1,00	40,00	#NULL!
51,00	2,00	2,00	2,00	2,00	2,00	2,00	#NULL!	#NULL!
53,00	2,00	2,00	2,00	2,00	2,00	2,00	#NULL!	#NULL!
55,00	1,00	2,00	2,00	2,00	2,00	2,00	#NULL!	#NULL!
52,00	2,00	2,00	2,00	2,00	2,00	1,00	5,00	#NULL!
62,00	2,00	2,00	2,00	2,00	2,00	1,00	30,00	#NULL!
61,00	1,00	1,00	2,00	2,00	2,00	2,00	#NULL!	#NULL!
50,00	2,00	2,00	1,00	2,00	2,00	1,00	20,00	#NULL!
61,00	1,00	1,00	2,00	2,00	2,00	2,00	#NULL!	#NULL!
54,00	2,00	2,00	2,00	2,00	2,00	2,00	#NULL!	#NULL!
45,00	1,00	2,00	2,00	2,00	2,00	2,00	#NULL!	#NULL!
46,00	2,00	2,00	2,00	2,00	2,00	2,00	#NULL!	#NULL!
62,00	1,00	1,00	2,00	2,00	2,00	2,00	#NULL!	#NULL!
58,00	1,00	2,00	2,00	2,00	2,00	2,00	#NULL!	#NULL!
63,00	1,00	1,00	2,00	2,00	2,00	1,00	40,00	#NULL!
64,00	1,00	1,00	2,00	2,00	2,00	1,00	42,00	#NULL!
51,00	1,00	1,00	2,00	2,00	2,00	1,00	20,00	#NULL!
48,00	2,00	1,00	2,00	2,00	2,00	2,00	#NULL!	#NULL!
46,00	1,00	2,00	2,00	2,00	2,00	2,00	#NULL!	#NULL!
45,00	2,00	2,00	2,00	2,00	2,00	2,00	#NULL!	#NULL!
81,00	2,00	1,00	2,00	2,00	2,00	2,00	#NULL!	#NULL!
88,00	1,00	1,00	1,00	2,00	2,00	2,00	#NULL!	#NULL!
56,00	2,00	2,00	2,00	2,00	2,00	2,00	#NULL!	#NULL!
66,00	1,00	2,00	2,00	2,00	2,00	2,00	#NULL!	#NULL!
49,00	2,00	2,00	2,00	2,00	2,00	2,00	#NULL!	#NULL!
50,00	1,00	1,00	2,00	2,00	2,00	1,00	20,00	#NULL!
69,00	1,00	1,00	1,00	2,00	2,00	2,00	#NULL!	#NULL!
62,00	1,00	1,00	1,00	2,00	1,00	2,00	#NULL!	#NULL!
59,00	1,00	1,00	2,00	2,00	1,00	2,00	#NULL!	#NULL!
58,00	1,00	2,00	2,00	2,00	2,00	2,00	#NULL!	#NULL!
55,00	1,00	1,00	1,00	2,00	1,00	1,00	20,00	#NULL!

49,00	1,00	2,00	2,00	2,00	2,00	2,00	#NULL!	#NULL!
62,00	1,00	1,00	1,00	1,00	1,00	1,00	40,00	#NULL!
55,00	1,00	2,00	1,00	2,00	2,00	2,00	#NULL!	#NULL!
50,00	1,00	2,00	2,00	2,00	2,00	1,00	30,00	#NULL!
60,00	1,00	2,00	2,00	2,00	2,00	1,00	20,00	#NULL!
53,00	1,00	2,00	2,00	2,00	2,00	1,00	15,00	#NULL!
50,00	1,00	2,00	2,00	2,00	2,00	1,00	22,00	#NULL!
62,00	1,00	1,00	2,00	2,00	2,00	1,00	5,00	#NULL!
59,00	2,00	1,00	1,00	2,00	2,00	2,00	#NULL!	#NULL!
50,00	2,00	2,00	1,00	2,00	2,00	2,00	#NULL!	#NULL!
54,00	1,00	1,00	1,00	2,00	2,00	2,00	#NULL!	#NULL!
45,00	2,00	2,00	2,00	2,00	2,00	1,00	18,00	#NULL!
60,00	2,00	1,00	2,00	2,00	2,00	2,00	#NULL!	#NULL!
58,00	1,00	2,00	2,00	2,00	1,00	2,00	#NULL!	#NULL!
57,00	1,00	2,00	2,00	2,00	2,00	2,00	#NULL!	#NULL!
65,00	2,00	1,00	1,00	2,00	2,00	1,00	40,00	#NULL!
59,00	1,00	1,00	2,00	2,00	2,00	1,00	50,00	#NULL!
60,00	2,00	2,00	2,00	2,00	2,00	2,00	#NULL!	#NULL!
62,00	2,00	2,00	2,00	2,00	2,00	1,00	20,00	#NULL!
70,00	1,00	1,00	2,00	2,00	2,00	1,00	60,00	#NULL!
65,00	2,00	1,00	1,00	2,00	2,00	2,00	#NULL!	#NULL!
49,00	1,00	2,00	2,00	2,00	2,00	1,00	10,00	#NULL!
45,00	2,00	2,00	2,00	2,00	2,00	1,00	8,00	#NULL!
80,00	1,00	1,00	1,00	2,00	2,00	2,00	#NULL!	#NULL!
74,00	2,00	1,00	1,00	2,00	2,00	2,00	#NULL!	#NULL!
54,00	1,00	2,00	2,00	1,00	2,00	2,00	#NULL!	#NULL!
55,00	1,00	2,00	2,00	2,00	2,00	2,00	#NULL!	#NULL!
58,00	2,00	2,00	2,00	2,00	2,00	2,00	#NULL!	#NULL!
64,00	2,00	1,00	2,00	1,00	2,00	2,00	#NULL!	#NULL!
70,00	1,00	1,00	2,00	2,00	2,00	2,00	#NULL!	#NULL!
60,00	2,00	1,00	2,00	2,00	2,00	2,00	#NULL!	#NULL!
65,00	1,00	2,00	2,00	2,00	2,00	1,00	80,00	#NULL!
57,00	2,00	2,00	2,00	2,00	2,00	1,00	15,00	#NULL!
51,00	2,00	2,00	1,00	2,00	2,00	2,00	#NULL!	#NULL!
55,00	1,00	2,00	2,00	2,00	2,00	1,00	70,00	#NULL!
45,00	2,00	2,00	2,00	2,00	2,00	2,00	#NULL!	#NULL!
50,00	2,00	2,00	1,00	2,00	2,00	2,00	#NULL!	#NULL!
47,00	2,00	2,00	2,00	2,00	1,00	2,00	#NULL!	#NULL!
51,00	1,00	2,00	2,00	2,00	2,00	1,00	30,00	#NULL!
45,00	1,00	2,00	2,00	2,00	2,00	2,00	#NULL!	#NULL!
55,00	1,00	2,00	2,00	2,00	2,00	1,00	10,00	#NULL!
59,00	1,00	2,00	2,00	2,00	2,00	2,00	#NULL!	#NULL!
55,00	2,00	2,00	2,00	2,00	2,00	2,00	#NULL!	#NULL!
46,00	1,00	2,00	2,00	2,00	2,00	2,00	#NULL!	#NULL!
57,00	1,00	2,00	1,00	2,00	2,00	2,00	#NULL!	#NULL!
51,00	1,00	2,00	2,00	2,00	2,00	2,00	#NULL!	#NULL!
51,00	2,00	2,00	2,00	2,00	2,00	2,00	#NULL!	#NULL!
53,00	2,00	2,00	2,00	2,00	2,00	2,00	#NULL!	#NULL!
56,00	1,00	2,00	2,00	2,00	2,00	2,00	#NULL!	#NULL!
60,00	1,00	2,00	2,00	2,00	2,00	2,00	#NULL!	#NULL!

45,00	2,00	2,00	2,00	2,00	2,00	2,00	#NULL!	#NULL!
46,00	1,00	2,00	2,00	2,00	2,00	2,00	#NULL!	#NULL!
45,00	2,00	2,00	2,00	2,00	2,00	2,00	#NULL!	#NULL!
59,00	1,00	2,00	2,00	2,00	2,00	2,00	#NULL!	#NULL!
60,00	1,00	1,00	2,00	2,00	2,00	2,00	#NULL!	#NULL!
47,00	2,00	2,00	2,00	2,00	2,00	2,00	#NULL!	#NULL!
45,00	2,00	2,00	2,00	2,00	2,00	2,00	#NULL!	#NULL!
48,00	2,00	2,00	2,00	2,00	2,00	1,00	15,00	#NULL!
49,00	1,00	2,00	2,00	2,00	2,00	1,00	#NULL!	#NULL!
49,00	1,00	2,00	2,00	2,00	2,00	2,00	#NULL!	#NULL!
48,00	2,00	2,00	2,00	2,00	2,00	2,00	#NULL!	#NULL!
45,00	2,00	2,00	2,00	2,00	2,00	2,00	#NULL!	#NULL!
45,00	1,00	2,00	2,00	2,00	2,00	2,00	#NULL!	#NULL!
45,00	2,00	2,00	2,00	2,00	2,00	2,00	#NULL!	#NULL!
46,00	1,00	2,00	2,00	2,00	2,00	2,00	#NULL!	#NULL!
46,00	1,00	2,00	2,00	2,00	2,00	2,00	#NULL!	#NULL!
45,00	2,00	2,00	2,00	2,00	2,00	2,00	#NULL!	#NULL!
45,00	2,00	2,00	2,00	2,00	2,00	2,00	#NULL!	#NULL!
46,00	1,00	2,00	2,00	2,00	2,00	2,00	#NULL!	#NULL!
45,00	2,00	2,00	2,00	2,00	2,00	2,00	#NULL!	#NULL!
48,00	1,00	2,00	2,00	2,00	2,00	2,00	#NULL!	#NULL!
50,00	1,00	2,00	2,00	2,00	2,00	1,00	10,00	#NULL!
47,00	2,00	2,00	2,00	2,00	2,00	2,00	#NULL!	#NULL!
48,00	2,00	2,00	2,00	2,00	2,00	2,00	#NULL!	#NULL!
46,00	2,00	2,00	2,00	2,00	2,00	2,00	#NULL!	#NULL!
49,00	2,00	2,00	2,00	2,00	2,00	2,00	#NULL!	#NULL!
48,00	1,00	2,00	2,00	2,00	2,00	2,00	#NULL!	#NULL!
45,00	1,00	2,00	2,00	2,00	2,00	2,00	#NULL!	#NULL!
64,00	2,00	2,00	2,00	2,00	2,00	2,00	#NULL!	#NULL!
47,00	1,00	2,00	2,00	2,00	2,00	2,00	#NULL!	#NULL!
45,00	2,00	2,00	2,00	2,00	2,00	2,00	#NULL!	#NULL!
48,00	1,00	2,00	2,00	2,00	2,00	2,00	#NULL!	#NULL!
47,00	2,00	2,00	2,00	2,00	2,00	2,00	#NULL!	#NULL!
47,00	2,00	2,00	2,00	2,00	2,00	2,00	#NULL!	#NULL!
50,00	1,00	2,00	2,00	2,00	2,00	2,00	#NULL!	#NULL!
45,00	2,00	2,00	2,00	2,00	2,00	2,00	#NULL!	#NULL!
48,00	1,00	2,00	2,00	2,00	2,00	2,00	#NULL!	#NULL!
77,00	2,00	1,00	1,00	2,00	1,00	2,00	#NULL!	#NULL!
58,00	1,00	2,00	2,00	2,00	2,00	2,00	#NULL!	#NULL!
60,00	1,00	1,00	1,00	2,00	1,00	2,00	#NULL!	#NULL!
56,00	2,00	1,00	1,00	2,00	2,00	2,00	#NULL!	#NULL!
52,00	2,00	1,00	1,00	2,00	2,00	2,00	#NULL!	#NULL!
46,00	2,00	2,00	2,00	2,00	2,00	2,00	#NULL!	#NULL!
47,00	2,00	1,00	2,00	2,00	2,00	2,00	#NULL!	#NULL!
58,00	1,00	1,00	2,00	2,00	2,00	2,00	#NULL!	#NULL!
45,00	1,00	2,00	2,00	2,00	2,00	2,00	#NULL!	#NULL!
66,00	2,00	1,00	1,00	2,00	2,00	2,00	#NULL!	#NULL!
55,00	1,00	2,00	2,00	2,00	2,00	2,00	#NULL!	#NULL!
45,00	2,00	2,00	2,00	2,00	2,00	2,00	#NULL!	#NULL!
48,00	1,00	2,00	2,00	2,00	2,00	2,00	#NULL!	#NULL!

45,00	2,00	2,00	2,00	2,00	2,00	2,00	#NULL!	#NULL!
46,00	1,00	2,00	2,00	2,00	2,00	2,00	#NULL!	#NULL!
45,00	2,00	2,00	2,00	2,00	2,00	2,00	#NULL!	#NULL!
45,00	2,00	2,00	2,00	2,00	2,00	2,00	#NULL!	#NULL!
45,00	1,00	2,00	2,00	2,00	2,00	2,00	#NULL!	#NULL!
50,00	1,00	2,00	2,00	2,00	2,00	2,00	#NULL!	#NULL!
48,00	2,00	2,00	2,00	2,00	2,00	2,00	#NULL!	#NULL!
52,00	2,00	2,00	2,00	2,00	2,00	2,00	#NULL!	#NULL!
47,00	2,00	2,00	2,00	2,00	2,00	2,00	#NULL!	#NULL!
62,00	1,00	1,00	1,00	2,00	2,00	2,00	#NULL!	#NULL!
61,00	1,00	1,00	1,00	2,00	2,00	2,00	#NULL!	#NULL!
45,00	1,00	2,00	2,00	2,00	2,00	2,00	#NULL!	#NULL!
62,00	2,00	2,00	2,00	2,00	2,00	2,00	#NULL!	#NULL!
46,00	2,00	2,00	2,00	2,00	2,00	1,00	20,00	#NULL!
69,00	1,00	2,00	2,00	2,00	2,00	1,00	45,00	#NULL!
45,00	2,00	2,00	2,00	2,00	2,00	2,00	#NULL!	#NULL!
45,00	1,00	2,00	2,00	2,00	2,00	2,00	#NULL!	#NULL!
52,00	1,00	2,00	2,00	2,00	2,00	2,00	#NULL!	#NULL!
46,00	2,00	2,00	2,00	2,00	2,00	2,00	#NULL!	#NULL!
47,00	2,00	2,00	2,00	2,00	2,00	2,00	#NULL!	#NULL!
53,00	1,00	2,00	2,00	2,00	2,00	2,00	#NULL!	#NULL!
47,00	2,00	2,00	2,00	2,00	2,00	2,00	#NULL!	#NULL!
52,00	1,00	2,00	2,00	2,00	2,00	2,00	#NULL!	#NULL!
60,00	2,00	2,00	2,00	2,00	2,00	2,00	#NULL!	#NULL!
45,00	2,00	2,00	2,00	2,00	2,00	2,00	#NULL!	#NULL!
50,00	2,00	2,00	2,00	2,00	2,00	2,00	#NULL!	#NULL!
52,00	1,00	2,00	2,00	2,00	2,00	2,00	#NULL!	#NULL!
60,00	1,00	2,00	2,00	2,00	2,00	2,00	#NULL!	#NULL!
55,00	2,00	2,00	2,00	2,00	2,00	2,00	#NULL!	#NULL!
60,00	1,00	2,00	2,00	2,00	2,00	2,00	#NULL!	#NULL!
67,00	2,00	2,00	2,00	2,00	2,00	2,00	#NULL!	#NULL!
54,00	1,00	2,00	2,00	2,00	2,00	2,00	#NULL!	#NULL!
58,00	2,00	2,00	2,00	2,00	2,00	2,00	#NULL!	#NULL!
50,00	1,00	2,00	2,00	2,00	2,00	2,00	#NULL!	#NULL!
46,00	2,00	2,00	2,00	2,00	2,00	2,00	#NULL!	#NULL!
45,00	2,00	2,00	2,00	2,00	2,00	2,00	#NULL!	#NULL!
47,00	1,00	2,00	2,00	2,00	2,00	2,00	#NULL!	#NULL!
45,00	1,00	2,00	2,00	2,00	2,00	1,00	20,00	#NULL!
60,00	1,00	2,00	2,00	2,00	2,00	2,00	#NULL!	#NULL!
52,00	2,00	2,00	1,00	2,00	2,00	2,00	#NULL!	#NULL!
60,00	1,00	2,00	2,00	2,00	2,00	2,00	#NULL!	#NULL!
65,00	2,00	2,00	2,00	2,00	2,00	2,00	#NULL!	#NULL!
47,00	1,00	2,00	2,00	2,00	2,00	2,00	#NULL!	#NULL!
45,00	2,00	2,00	2,00	2,00	2,00	2,00	#NULL!	#NULL!
56,00	1,00	2,00	2,00	2,00	2,00	2,00	#NULL!	#NULL!
49,00	2,00	2,00	2,00	2,00	2,00	1,00	10,00	#NULL!
55,00	1,00	2,00	2,00	2,00	2,00	2,00	#NULL!	#NULL!
60,00	1,00	1,00	2,00	2,00	2,00	2,00	#NULL!	#NULL!
73,00	1,00	1,00	2,00	2,00	1,00	1,00	45,00	#NULL!
72,00	1,00	2,00	2,00	2,00	2,00	1,00	25,00	#NULL!

60,00	1,00	1,00	2,00	2,00	2,00	2,00	#NULL!	#NULL!
72,00	1,00	1,00	2,00	2,00	2,00	2,00	#NULL!	#NULL!
68,00	2,00	1,00	2,00	2,00	2,00	2,00	#NULL!	#NULL!
58,00	1,00	2,00	2,00	2,00	2,00	2,00	#NULL!	#NULL!
58,00	2,00	1,00	2,00	2,00	1,00	2,00	#NULL!	#NULL!
72,00	1,00	1,00	2,00	1,00	2,00	2,00	#NULL!	#NULL!
50,00	1,00	2,00	2,00	2,00	1,00	2,00	#NULL!	#NULL!
52,00	1,00	2,00	2,00	2,00	2,00	1,00	15,00	#NULL!
50,00	1,00	2,00	2,00	2,00	2,00	2,00	#NULL!	#NULL!
63,00	1,00	1,00	2,00	1,00	2,00	2,00	#NULL!	#NULL!
80,00	1,00	1,00	2,00	2,00	1,00	2,00	#NULL!	#NULL!
80,00	1,00	1,00	2,00	2,00	2,00	2,00	#NULL!	#NULL!
45,00	1,00	2,00	2,00	2,00	2,00	2,00	#NULL!	#NULL!
60,00	2,00	1,00	2,00	2,00	2,00	2,00	#NULL!	#NULL!
54,00	1,00	2,00	2,00	2,00	2,00	2,00	#NULL!	#NULL!
52,00	2,00	1,00	2,00	2,00	2,00	2,00	#NULL!	#NULL!
51,00	1,00	2,00	2,00	2,00	2,00	2,00	#NULL!	#NULL!
49,00	2,00	2,00	2,00	2,00	2,00	2,00	#NULL!	#NULL!
51,00	1,00	2,00	2,00	2,00	2,00	2,00	#NULL!	#NULL!
43,00	1,00	2,00	2,00	2,00	2,00	1,00	40,00	#NULL!
80,00	1,00	1,00	2,00	2,00	2,00	2,00	#NULL!	#NULL!
54,00	1,00	2,00	2,00	2,00	2,00	1,00	15,00	#NULL!
65,00	1,00	1,00	1,00	2,00	2,00	1,00	45,00	#NULL!
60,00	2,00	1,00	2,00	2,00	2,00	2,00	#NULL!	#NULL!
75,00	1,00	1,00	2,00	1,00	2,00	1,00	50,00	#NULL!
65,00	1,00	1,00	2,00	1,00	2,00	1,00	45,00	#NULL!
62,00	1,00	2,00	2,00	2,00	2,00	1,00	20,00	#NULL!
56,00	1,00	2,00	1,00	2,00	2,00	2,00	#NULL!	#NULL!
55,00	1,00	2,00	2,00	2,00	2,00	2,00	#NULL!	#NULL!
49,00	2,00	2,00	2,00	2,00	2,00	2,00	#NULL!	#NULL!
50,00	1,00	2,00	2,00	2,00	2,00	1,00	25,00	#NULL!
55,00	2,00	1,00	2,00	2,00	2,00	2,00	#NULL!	#NULL!
72,00	1,00	2,00	2,00	2,00	2,00	2,00	#NULL!	#NULL!
75,00	1,00	2,00	2,00	1,00	2,00	2,00	#NULL!	#NULL!
58,00	1,00	2,00	2,00	2,00	2,00	1,00	30,00	#NULL!
62,00	2,00	1,00	1,00	2,00	2,00	2,00	#NULL!	#NULL!
51,00	2,00	2,00	2,00	2,00	2,00	2,00	#NULL!	#NULL!
56,00	2,00	2,00	2,00	2,00	2,00	2,00	#NULL!	#NULL!
50,00	2,00	1,00	2,00	1,00	1,00	2,00	#NULL!	#NULL!
55,00	1,00	2,00	2,00	2,00	2,00	1,00	90,00	#NULL!
49,00	1,00	1,00	2,00	2,00	2,00	1,00	25,00	#NULL!
65,00	1,00	1,00	1,00	2,00	2,00	2,00	#NULL!	#NULL!
55,00	1,00	2,00	1,00	2,00	2,00	1,00	40,00	#NULL!
72,00	2,00	1,00	2,00	2,00	2,00	2,00	#NULL!	#NULL!
54,00	1,00	1,00	2,00	2,00	2,00	1,00	60,00	#NULL!
51,00	2,00	1,00	1,00	1,00	1,00	1,00	30,00	#NULL!
54,00	2,00	2,00	2,00	2,00	1,00	1,00	30,00	#NULL!
56,00	2,00	1,00	2,00	2,00	2,00	2,00	#NULL!	#NULL!
60,00	1,00	2,00	2,00	2,00	2,00	1,00	60,00	#NULL!
51,00	2,00	2,00	2,00	2,00	1,00	2,00	#NULL!	#NULL!

86,00	2,00	1,00	2,00	2,00	2,00	2,00	#NULL!	#NULL!
56,00	2,00	2,00	2,00	2,00	2,00	2,00	#NULL!	#NULL!
49,00	1,00	2,00	2,00	2,00	2,00	2,00	#NULL!	#NULL!
60,00	1,00	2,00	2,00	2,00	2,00	1,00	40,00	#NULL!
61,00	1,00	2,00	1,00	2,00	2,00	1,00	46,00	#NULL!
65,00	2,00	2,00	1,00	2,00	2,00	2,00	#NULL!	#NULL!
48,00	2,00	2,00	2,00	2,00	2,00	1,00	14,00	#NULL!
45,00	2,00	2,00	2,00	2,00	2,00	2,00	#NULL!	#NULL!
70,00	2,00	1,00	2,00	2,00	1,00	2,00	#NULL!	#NULL!
74,00	1,00	2,00	1,00	1,00	2,00	2,00	#NULL!	#NULL!
58,00	2,00	1,00	1,00	2,00	1,00	2,00	#NULL!	#NULL!
47,00	2,00	1,00	1,00	1,00	2,00	2,00	#NULL!	#NULL!
48,00	1,00	2,00	1,00	2,00	1,00	2,00	#NULL!	#NULL!
47,00	2,00	2,00	2,00	2,00	1,00	2,00	#NULL!	#NULL!
62,00	1,00	2,00	2,00	1,00	2,00	2,00	#NULL!	#NULL!
62,00	2,00	2,00	2,00	1,00	2,00	2,00	#NULL!	#NULL!
48,00	1,00	2,00	1,00	2,00	1,00	2,00	#NULL!	#NULL!
47,00	2,00	2,00	2,00	2,00	1,00	2,00	#NULL!	#NULL!
66,00	1,00	1,00	2,00	1,00	1,00	2,00	30,00	1,00
65,00	1,00	2,00	2,00	2,00	2,00	2,00	#NULL!	#NULL!
54,00	1,00	2,00	2,00	2,00	2,00	2,00	#NULL!	#NULL!
45,00	2,00	2,00	2,00	2,00	2,00	2,00	#NULL!	#NULL!
55,00	1,00	2,00	2,00	2,00	2,00	2,00	#NULL!	#NULL!
72,00	1,00	2,00	2,00	2,00	2,00	2,00	#NULL!	#NULL!
50,00	1,00	2,00	1,00	2,00	2,00	2,00	#NULL!	#NULL!
62,00	1,00	1,00	1,00	2,00	2,00	2,00	#NULL!	#NULL!
50,00	1,00	2,00	2,00	2,00	2,00	2,00	#NULL!	#NULL!
56,00	1,00	1,00	2,00	2,00	2,00	2,00	#NULL!	#NULL!
56,00	1,00	2,00	2,00	1,00	1,00	2,00	#NULL!	#NULL!
56,00	1,00	2,00	2,00	2,00	2,00	2,00	#NULL!	#NULL!
50,00	1,00	2,00	1,00	2,00	1,00	2,00	#NULL!	#NULL!
50,00	2,00	2,00	2,00	2,00	2,00	2,00	#NULL!	#NULL!
46,00	2,00	2,00	2,00	2,00	2,00	2,00	#NULL!	#NULL!
56,00	1,00	1,00	2,00	2,00	2,00	2,00	#NULL!	#NULL!
50,00	1,00	2,00	2,00	2,00	2,00	2,00	#NULL!	#NULL!
56,00	1,00	1,00	2,00	2,00	2,00	2,00	#NULL!	#NULL!
45,00	2,00	2,00	2,00	2,00	2,00	2,00	#NULL!	#NULL!
50,00	2,00	1,00	2,00	2,00	2,00	2,00	#NULL!	#NULL!
54,00	1,00	2,00	2,00	1,00	2,00	2,00	10,00	20,00
56,00	1,00	2,00	2,00	2,00	2,00	2,00	#NULL!	#NULL!
49,00	2,00	2,00	2,00	2,00	2,00	2,00	#NULL!	#NULL!
52,00	2,00	1,00	2,00	2,00	2,00	2,00	#NULL!	#NULL!
63,00	1,00	1,00	2,00	2,00	2,00	2,00	#NULL!	#NULL!
60,00	2,00	1,00	2,00	2,00	2,00	2,00	#NULL!	#NULL!
45,00	2,00	2,00	2,00	2,00	2,00	2,00	#NULL!	#NULL!
50,00	2,00	1,00	2,00	2,00	2,00	2,00	#NULL!	#NULL!
50,00	1,00	2,00	2,00	2,00	2,00	1,00	10,00	#NULL!
55,00	1,00	1,00	2,00	1,00	2,00	2,00	#NULL!	#NULL!
49,00	1,00	2,00	2,00	2,00	2,00	1,00	2,00	#NULL!
49,00	2,00	2,00	2,00	2,00	2,00	2,00	#NULL!	#NULL!

59,00	2,00	2,00	2,00	2,00	2,00	2,00	#NULL!	#NULL!
49,00	2,00	2,00	2,00	2,00	2,00	2,00	#NULL!	#NULL!
70,00	2,00	1,00	2,00	2,00	2,00	2,00	#NULL!	#NULL!
65,00	2,00	2,00	2,00	2,00	2,00	2,00	#NULL!	#NULL!
52,00	2,00	1,00	1,00	1,00	1,00	2,00	#NULL!	#NULL!
46,00	2,00	2,00	2,00	2,00	2,00	2,00	#NULL!	#NULL!
56,00	1,00	2,00	2,00	1,00	1,00	2,00	#NULL!	#NULL!
51,00	1,00	2,00	2,00	2,00	2,00	2,00	#NULL!	#NULL!
55,00	1,00	2,00	2,00	2,00	2,00	2,00	#NULL!	#NULL!
50,00	2,00	2,00	2,00	2,00	2,00	2,00	#NULL!	#NULL!
52,00	1,00	2,00	2,00	2,00	2,00	2,00	#NULL!	#NULL!
50,00	2,00	2,00	1,00	2,00	2,00	2,00	#NULL!	#NULL!
49,00	2,00	1,00	2,00	2,00	2,00	2,00	#NULL!	#NULL!
50,00	1,00	2,00	2,00	2,00	2,00	2,00	#NULL!	#NULL!
50,00	1,00	2,00	2,00	2,00	2,00	2,00	#NULL!	#NULL!
46,00	1,00	2,00	2,00	2,00	2,00	2,00	#NULL!	#NULL!
55,00	1,00	1,00	2,00	1,00	2,00	2,00	#NULL!	#NULL!
45,00	2,00	2,00	2,00	2,00	2,00	2,00	#NULL!	#NULL!
58,00	1,00	1,00	2,00	2,00	1,00	2,00	#NULL!	#NULL!
55,00	2,00	2,00	2,00	2,00	2,00	2,00	#NULL!	#NULL!
50,00	1,00	2,00	2,00	2,00	2,00	1,00	60,00	#NULL!
45,00	2,00	2,00	2,00	2,00	2,00	2,00	#NULL!	#NULL!
49,00	2,00	2,00	2,00	2,00	2,00	2,00	#NULL!	#NULL!
52,00	1,00	2,00	2,00	2,00	2,00	2,00	#NULL!	#NULL!
49,00	2,00	2,00	2,00	2,00	2,00	2,00	#NULL!	#NULL!
46,00	2,00	2,00	2,00	2,00	2,00	2,00	#NULL!	#NULL!
59,00	1,00	2,00	2,00	2,00	2,00	2,00	#NULL!	#NULL!
50,00	2,00	2,00	2,00	2,00	2,00	2,00	#NULL!	#NULL!
47,00	2,00	1,00	2,00	2,00	2,00	2,00	#NULL!	#NULL!
49,00	2,00	2,00	2,00	2,00	2,00	2,00	#NULL!	#NULL!
51,00	2,00	2,00	2,00	2,00	2,00	2,00	#NULL!	#NULL!
45,00	2,00	2,00	2,00	2,00	2,00	2,00	#NULL!	#NULL!
51,00	1,00	1,00	2,00	2,00	2,00	2,00	#NULL!	#NULL!
53,00	1,00	2,00	2,00	2,00	2,00	2,00	#NULL!	#NULL!
46,00	1,00	2,00	2,00	2,00	2,00	1,00	10,00	#NULL!
59,00	1,00	2,00	1,00	2,00	2,00	2,00	#NULL!	#NULL!
66,00	1,00	2,00	2,00	2,00	2,00	2,00	#NULL!	#NULL!
49,00	1,00	1,00	2,00	2,00	2,00	1,00	3,00	#NULL!
55,00	2,00	1,00	1,00	2,00	2,00	2,00	#NULL!	#NULL!
48,00	2,00	1,00	2,00	2,00	2,00	2,00	#NULL!	#NULL!
50,00	1,00	1,00	2,00	2,00	2,00	1,00	25,00	#NULL!
65,00	1,00	1,00	2,00	2,00	2,00	2,00	#NULL!	#NULL!
63,00	2,00	2,00	1,00	2,00	2,00	2,00	#NULL!	#NULL!
65,00	2,00	1,00	2,00	2,00	2,00	2,00	#NULL!	#NULL!
66,00	1,00	1,00	1,00	2,00	2,00	2,00	#NULL!	#NULL!
49,00	2,00	2,00	2,00	2,00	2,00	2,00	#NULL!	#NULL!
47,00	1,00	2,00	2,00	2,00	2,00	1,00	20,00	#NULL!
49,00	2,00	2,00	2,00	2,00	2,00	1,00	30,00	#NULL!
50,00	1,00	1,00	2,00	2,00	2,00	2,00	#NULL!	#NULL!
48,00	2,00	2,00	2,00	2,00	2,00	2,00	#NULL!	#NULL!

49,00	1,00	2,00	2,00	2,00	2,00	2,00	#NULL!	#NULL!
53,00	1,00	1,00	2,00	2,00	2,00	2,00	#NULL!	#NULL!
54,00	2,00	1,00	2,00	2,00	2,00	2,00	#NULL!	#NULL!
66,00	2,00	1,00	2,00	2,00	2,00	2,00	#NULL!	#NULL!
67,00	1,00	1,00	1,00	2,00	2,00	2,00	#NULL!	#NULL!
48,00	2,00	2,00	2,00	2,00	2,00	2,00	#NULL!	#NULL!
48,00	1,00	2,00	2,00	2,00	2,00	2,00	#NULL!	#NULL!
75,00	2,00	1,00	2,00	2,00	2,00	2,00	#NULL!	#NULL!
77,00	1,00	2,00	2,00	2,00	2,00	2,00	#NULL!	#NULL!
45,00	1,00	2,00	2,00	2,00	2,00	2,00	#NULL!	#NULL!
76,00	1,00	1,00	2,00	2,00	2,00	2,00	#NULL!	#NULL!
73,00	2,00	1,00	2,00	2,00	1,00	1,00	20,00	#NULL!
65,00	2,00	2,00	2,00	2,00	1,00	1,00	40,00	#NULL!
62,00	1,00	2,00	2,00	2,00	2,00	2,00	#NULL!	#NULL!
51,00	1,00	2,00	2,00	2,00	2,00	2,00	#NULL!	#NULL!
60,00	1,00	1,00	2,00	1,00	1,00	1,00	80,00	#NULL!
59,00	1,00	2,00	2,00	2,00	2,00	1,00	30,00	#NULL!
50,00	1,00	2,00	2,00	2,00	2,00	2,00	#NULL!	#NULL!
45,00	1,00	2,00	2,00	2,00	2,00	2,00	#NULL!	#NULL!
75,00	1,00	1,00	1,00	2,00	1,00	2,00	#NULL!	#NULL!
74,00	1,00	2,00	1,00	2,00	2,00	2,00	#NULL!	#NULL!
81,00	1,00	1,00	2,00	2,00	2,00	2,00	#NULL!	#NULL!
61,00	1,00	1,00	1,00	1,00	1,00	2,00	#NULL!	#NULL!
72,00	1,00	1,00	2,00	1,00	1,00	2,00	#NULL!	#NULL!
61,00	1,00	2,00	2,00	2,00	2,00	2,00	#NULL!	#NULL!
50,00	1,00	2,00	2,00	2,00	2,00	2,00	#NULL!	#NULL!
55,00	2,00	2,00	2,00	1,00	2,00	2,00	#NULL!	#NULL!
67,00	1,00	2,00	2,00	2,00	2,00	1,00	20,00	#NULL!
46,00	2,00	2,00	2,00	2,00	1,00	2,00	#NULL!	#NULL!
45,00	2,00	2,00	2,00	2,00	2,00	1,00	30,00	#NULL!
68,00	2,00	2,00	2,00	2,00	2,00	2,00	#NULL!	#NULL!
46,00	2,00	2,00	2,00	2,00	2,00	1,00	7,00	#NULL!
45,00	1,00	2,00	2,00	2,00	2,00	2,00	#NULL!	#NULL!
69,00	2,00	1,00	2,00	1,00	2,00	2,00	#NULL!	#NULL!
79,00	1,00	2,00	2,00	1,00	2,00	2,00	#NULL!	#NULL!
50,00	1,00	2,00	1,00	2,00	2,00	2,00	#NULL!	#NULL!
50,00	2,00	2,00	2,00	2,00	2,00	2,00	#NULL!	#NULL!
68,00	2,00	2,00	2,00	2,00	2,00	2,00	#NULL!	#NULL!
54,00	2,00	2,00	2,00	2,00	2,00	1,00	20,00	#NULL!
78,00	2,00	1,00	2,00	1,00	1,00	2,00	#NULL!	#NULL!
76,00	2,00	2,00	2,00	2,00	2,00	2,00	#NULL!	#NULL!
80,00	1,00	2,00	1,00	2,00	2,00	2,00	#NULL!	#NULL!
55,00	2,00	1,00	2,00	2,00	2,00	2,00	#NULL!	#NULL!
63,00	1,00	2,00	2,00	2,00	2,00	2,00	#NULL!	#NULL!
48,00	2,00	1,00	1,00	2,00	2,00	1,00	15,00	#NULL!
55,00	2,00	2,00	2,00	2,00	2,00	2,00	#NULL!	#NULL!
45,00	2,00	2,00	2,00	2,00	2,00	1,00	25,00	#NULL!
48,00	1,00	2,00	2,00	2,00	2,00	1,00	30,00	#NULL!
46,00	2,00	2,00	2,00	2,00	2,00	1,00	15,00	#NULL!
66,00	1,00	1,00	2,00	1,00	2,00	2,00	#NULL!	#NULL!

60,00	2,00	1,00	1,00	2,00	2,00	2,00	#NULL!	#NULL!
60,00	1,00	1,00	2,00	2,00	1,00	2,00	#NULL!	#NULL!
65,00	1,00	2,00	2,00	2,00	2,00	2,00	#NULL!	#NULL!
54,00	1,00	2,00	2,00	2,00	1,00	1,00	40,00	#NULL!
47,00	2,00	2,00	1,00	2,00	2,00	1,00	30,00	#NULL!
49,00	2,00	1,00	2,00	2,00	2,00	1,00	15,00	#NULL!
52,00	1,00	2,00	2,00	2,00	2,00	2,00	#NULL!	#NULL!
55,00	1,00	1,00	2,00	2,00	1,00	2,00	#NULL!	#NULL!
74,00	2,00	2,00	2,00	2,00	2,00	2,00	#NULL!	#NULL!
74,00	2,00	1,00	2,00	2,00	2,00	2,00	#NULL!	#NULL!
57,00	1,00	2,00	2,00	1,00	2,00	2,00	#NULL!	#NULL!
59,00	1,00	1,00	1,00	2,00	1,00	2,00	#NULL!	#NULL!
69,00	1,00	1,00	2,00	1,00	1,00	1,00	100,00	#NULL!
64,00	1,00	1,00	1,00	1,00	1,00	2,00	75,00	9,00
53,00	2,00	1,00	2,00	2,00	1,00	2,00	#NULL!	#NULL!
64,00	1,00	1,00	2,00	1,00	1,00	1,00	135,00	#NULL!
54,00	2,00	2,00	2,00	2,00	2,00	2,00	#NULL!	#NULL!
49,00	1,00	2,00	2,00	2,00	2,00	2,00	#NULL!	#NULL!
56,00	1,00	1,00	2,00	1,00	2,00	1,00	40,00	#NULL!
54,00	1,00	1,00	2,00	2,00	2,00	1,00	30,00	#NULL!
58,00	1,00	1,00	2,00	2,00	2,00	1,00	34,00	#NULL!
48,00	2,00	2,00	1,00	2,00	1,00	2,00	#NULL!	#NULL!
50,00	2,00	1,00	1,00	2,00	1,00	2,00	#NULL!	#NULL!
54,00	2,00	1,00	2,00	2,00	1,00	2,00	#NULL!	#NULL!
62,00	1,00	1,00	1,00	1,00	1,00	2,00	90,00	20,00
53,00	2,00	2,00	2,00	2,00	2,00	2,00	#NULL!	#NULL!
61,00	1,00	1,00	2,00	1,00	2,00	2,00	#NULL!	#NULL!
72,00	2,00	1,00	2,00	1,00	1,00	1,00	40,00	#NULL!
76,00	1,00	1,00	2,00	1,00	1,00	1,00	100,00	#NULL!
49,00	2,00	2,00	2,00	2,00	2,00	2,00	#NULL!	#NULL!
59,00	1,00	1,00	2,00	1,00	2,00	1,00	40,00	#NULL!
49,00	1,00	2,00	2,00	2,00	2,00	2,00	#NULL!	#NULL!
48,00	2,00	2,00	2,00	2,00	2,00	2,00	#NULL!	#NULL!
60,00	1,00	1,00	2,00	1,00	2,00	1,00	25,00	#NULL!
57,00	2,00	1,00	1,00	2,00	1,00	2,00	#NULL!	#NULL!
60,00	2,00	1,00	1,00	1,00	1,00	2,00	#NULL!	#NULL!
66,00	1,00	1,00	2,00	2,00	2,00	1,00	40,00	#NULL!
54,00	2,00	2,00	2,00	2,00	2,00	2,00	#NULL!	#NULL!
68,00	1,00	1,00	2,00	2,00	2,00	2,00	#NULL!	#NULL!
56,00	2,00	2,00	2,00	2,00	2,00	2,00	#NULL!	#NULL!
62,00	1,00	1,00	2,00	2,00	2,00	2,00	#NULL!	#NULL!
63,00	1,00	2,00	1,00	2,00	2,00	2,00	#NULL!	#NULL!
55,00	2,00	2,00	2,00	2,00	2,00	1,00	20,00	#NULL!
49,00	1,00	2,00	2,00	2,00	2,00	2,00	#NULL!	#NULL!
48,00	2,00	2,00	2,00	2,00	2,00	2,00	#NULL!	#NULL!
52,00	2,00	2,00	2,00	2,00	2,00	2,00	#NULL!	#NULL!
56,00	1,00	2,00	2,00	2,00	2,00	2,00	#NULL!	#NULL!
46,00	1,00	2,00	2,00	2,00	2,00	2,00	#NULL!	#NULL!
47,00	2,00	2,00	2,00	2,00	2,00	2,00	#NULL!	#NULL!
52,00	2,00	1,00	2,00	2,00	1,00	2,00	#NULL!	#NULL!

55,00	1,00	1,00	2,00	2,00	2,00	2,00	#NULL!	#NULL!
70,00	1,00	1,00	1,00	1,00	1,00	2,00	#NULL!	#NULL!
74,00	1,00	1,00	1,00	1,00	1,00	2,00	#NULL!	#NULL!
45,00	1,00	2,00	2,00	2,00	2,00	2,00	#NULL!	#NULL!
75,00	2,00	2,00	1,00	2,00	2,00	2,00	#NULL!	#NULL!
52,00	1,00	1,00	2,00	2,00	2,00	2,00	#NULL!	#NULL!
51,00	2,00	1,00	2,00	2,00	2,00	2,00	#NULL!	#NULL!
49,00	2,00	2,00	2,00	2,00	2,00	2,00	#NULL!	#NULL!
50,00	1,00	1,00	2,00	2,00	2,00	2,00	#NULL!	#NULL!
49,00	1,00	1,00	1,00	2,00	2,00	2,00	#NULL!	#NULL!
48,00	2,00	1,00	1,00	2,00	2,00	2,00	#NULL!	#NULL!
49,00	2,00	1,00	1,00	2,00	2,00	2,00	#NULL!	#NULL!
50,00	1,00	1,00	1,00	2,00	2,00	2,00	#NULL!	#NULL!
50,00	2,00	2,00	1,00	2,00	2,00	2,00	#NULL!	#NULL!
51,00	1,00	2,00	2,00	2,00	2,00	2,00	#NULL!	#NULL!
49,00	2,00	1,00	1,00	2,00	2,00	2,00	#NULL!	#NULL!
50,00	1,00	2,00	1,00	2,00	2,00	2,00	#NULL!	#NULL!
48,00	2,00	1,00	2,00	2,00	2,00	2,00	#NULL!	#NULL!
49,00	1,00	2,00	1,00	2,00	2,00	2,00	#NULL!	#NULL!
49,00	2,00	1,00	1,00	2,00	2,00	2,00	#NULL!	#NULL!
50,00	1,00	1,00	1,00	2,00	2,00	2,00	#NULL!	#NULL!
70,00	2,00	2,00	1,00	2,00	2,00	2,00	#NULL!	#NULL!
76,00	1,00	1,00	2,00	2,00	2,00	2,00	#NULL!	#NULL!
50,00	2,00	2,00	2,00	2,00	2,00	2,00	#NULL!	#NULL!
48,00	2,00	2,00	2,00	2,00	2,00	2,00	#NULL!	#NULL!
50,00	1,00	2,00	2,00	2,00	2,00	2,00	#NULL!	#NULL!
57,00	1,00	2,00	2,00	2,00	2,00	1,00	40,00	#NULL!
46,00	2,00	2,00	2,00	2,00	2,00	2,00	#NULL!	#NULL!
49,00	2,00	1,00	2,00	2,00	2,00	2,00	#NULL!	#NULL!
67,00	2,00	1,00	1,00	2,00	1,00	2,00	#NULL!	#NULL!
48,00	1,00	1,00	2,00	2,00	2,00	2,00	#NULL!	#NULL!
60,00	1,00	2,00	1,00	2,00	2,00	2,00	#NULL!	#NULL!
52,00	2,00	1,00	2,00	2,00	2,00	2,00	#NULL!	#NULL!
62,00	2,00	1,00	1,00	2,00	2,00	2,00	#NULL!	#NULL!
63,00	1,00	1,00	1,00	2,00	2,00	1,00	45,00	#NULL!
63,00	1,00	1,00	1,00	2,00	1,00	1,00	47,00	#NULL!
62,00	1,00	2,00	2,00	2,00	1,00	1,00	30,00	#NULL!
73,00	2,00	1,00	2,00	2,00	2,00	2,00	#NULL!	#NULL!
77,00	2,00	1,00	2,00	1,00	1,00	2,00	#NULL!	#NULL!
59,00	2,00	2,00	2,00	2,00	1,00	2,00	#NULL!	#NULL!
85,00	2,00	1,00	1,00	1,00	2,00	2,00	#NULL!	#NULL!
49,00	1,00	2,00	2,00	2,00	2,00	1,00	20,00	#NULL!
50,00	2,00	1,00	2,00	2,00	2,00	1,00	30,00	#NULL!
45,00	1,00	2,00	2,00	2,00	2,00	2,00	#NULL!	#NULL!
46,00	1,00	2,00	2,00	2,00	2,00	2,00	#NULL!	#NULL!
49,00	1,00	1,00	2,00	2,00	1,00	1,00	20,00	#NULL!
50,00	2,00	1,00	1,00	2,00	2,00	2,00	#NULL!	#NULL!
48,00	1,00	1,00	1,00	2,00	2,00	2,00	#NULL!	#NULL!
49,00	2,00	1,00	2,00	2,00	1,00	2,00	#NULL!	#NULL!
48,00	1,00	2,00	2,00	2,00	2,00	1,00	20,00	#NULL!

50,00	2,00	1,00	2,00	2,00	2,00	1,00	16,00	#NULL!
47,00	1,00	2,00	2,00	2,00	2,00	2,00	#NULL!	#NULL!
46,00	2,00	1,00	2,00	2,00	2,00	2,00	#NULL!	#NULL!
48,00	2,00	2,00	2,00	2,00	2,00	2,00	#NULL!	#NULL!
49,00	1,00	2,00	2,00	2,00	2,00	2,00	#NULL!	#NULL!
64,00	1,00	2,00	2,00	2,00	2,00	2,00	#NULL!	#NULL!
46,00	2,00	2,00	2,00	2,00	2,00	1,00	20,00	#NULL!
49,00	2,00	1,00	2,00	2,00	2,00	2,00	#NULL!	#NULL!
50,00	1,00	1,00	2,00	2,00	2,00	2,00	#NULL!	#NULL!
49,00	2,00	1,00	2,00	2,00	1,00	2,00	#NULL!	#NULL!
48,00	1,00	1,00	1,00	2,00	2,00	2,00	#NULL!	#NULL!
78,00	2,00	1,00	2,00	2,00	2,00	2,00	#NULL!	#NULL!
78,00	1,00	1,00	2,00	2,00	2,00	2,00	#NULL!	#NULL!
46,00	1,00	2,00	2,00	2,00	2,00	2,00	#NULL!	#NULL!
51,00	1,00	2,00	2,00	2,00	2,00	2,00	#NULL!	#NULL!
65,00	2,00	2,00	2,00	2,00	2,00	2,00	#NULL!	#NULL!
80,00	1,00	2,00	2,00	1,00	1,00	1,00	65,00	#NULL!
49,00	2,00	2,00	1,00	2,00	2,00	2,00	#NULL!	#NULL!
53,00	1,00	2,00	2,00	1,00	1,00	2,00	#NULL!	#NULL!
50,00	2,00	1,00	2,00	2,00	2,00	2,00	#NULL!	#NULL!
46,00	2,00	2,00	2,00	2,00	2,00	2,00	#NULL!	#NULL!
50,00	2,00	2,00	2,00	2,00	2,00	1,00	10,00	#NULL!
53,00	1,00	2,00	2,00	2,00	2,00	2,00	#NULL!	#NULL!
45,00	2,00	2,00	2,00	2,00	2,00	2,00	#NULL!	#NULL!
50,00	1,00	2,00	2,00	2,00	2,00	2,00	#NULL!	#NULL!
55,00	1,00	2,00	1,00	2,00	2,00	2,00	#NULL!	#NULL!
50,00	2,00	2,00	2,00	2,00	2,00	2,00	#NULL!	#NULL!
45,00	2,00	2,00	2,00	2,00	2,00	2,00	#NULL!	#NULL!
50,00	2,00	2,00	2,00	2,00	2,00	2,00	#NULL!	#NULL!
50,00	2,00	2,00	2,00	2,00	1,00	1,00	30,00	#NULL!
55,00	1,00	2,00	2,00	2,00	2,00	2,00	#NULL!	#NULL!
50,00	1,00	2,00	2,00	2,00	2,00	1,00	15,00	#NULL!
45,00	2,00	2,00	2,00	2,00	2,00	2,00	#NULL!	#NULL!
45,00	2,00	2,00	2,00	2,00	2,00	2,00	#NULL!	#NULL!
55,00	1,00	2,00	2,00	2,00	2,00	2,00	#NULL!	#NULL!
48,00	2,00	2,00	2,00	2,00	2,00	2,00	#NULL!	#NULL!
45,00	1,00	2,00	2,00	2,00	2,00	2,00	#NULL!	#NULL!
50,00	1,00	2,00	2,00	2,00	2,00	2,00	#NULL!	#NULL!
48,00	2,00	2,00	2,00	2,00	2,00	2,00	#NULL!	#NULL!
45,00	2,00	1,00	2,00	2,00	2,00	1,00	10,00	#NULL!
48,00	1,00	2,00	2,00	2,00	2,00	2,00	#NULL!	#NULL!
50,00	2,00	2,00	2,00	2,00	2,00	2,00	#NULL!	#NULL!
58,00	1,00	2,00	2,00	2,00	2,00	2,00	#NULL!	#NULL!
51,00	1,00	2,00	2,00	2,00	2,00	2,00	#NULL!	#NULL!
55,00	1,00	2,00	1,00	2,00	2,00	2,00	#NULL!	#NULL!
55,00	1,00	2,00	2,00	2,00	2,00	2,00	#NULL!	#NULL!
50,00	2,00	2,00	2,00	2,00	2,00	2,00	#NULL!	#NULL!
55,00	2,00	2,00	2,00	2,00	1,00	2,00	#NULL!	#NULL!
82,00	1,00	1,00	2,00	1,00	2,00	2,00	#NULL!	#NULL!
75,00	2,00	1,00	1,00	1,00	1,00	2,00	#NULL!	#NULL!

70,00	2,00	1,00	2,00	2,00	1,00	2,00	#NULL!	#NULL!
85,00	2,00	1,00	1,00	2,00	1,00	2,00	#NULL!	#NULL!
75,00	1,00	2,00	2,00	2,00	2,00	2,00	#NULL!	#NULL!
54,00	2,00	2,00	2,00	2,00	2,00	2,00	#NULL!	#NULL!
54,00	1,00	2,00	2,00	2,00	2,00	2,00	#NULL!	#NULL!
51,00	2,00	1,00	1,00	1,00	1,00	2,00	#NULL!	#NULL!
65,00	1,00	2,00	1,00	2,00	1,00	2,00	#NULL!	#NULL!
52,00	1,00	2,00	2,00	2,00	2,00	2,00	#NULL!	#NULL!
76,00	1,00	2,00	2,00	2,00	2,00	2,00	#NULL!	#NULL!
65,00	2,00	2,00	2,00	2,00	2,00	2,00	#NULL!	#NULL!
62,00	1,00	2,00	2,00	2,00	1,00	2,00	#NULL!	#NULL!
46,00	1,00	2,00	2,00	2,00	2,00	2,00	#NULL!	#NULL!
50,00	1,00	1,00	1,00	2,00	2,00	2,00	#NULL!	#NULL!
56,00	1,00	2,00	2,00	2,00	2,00	1,00	60,00	#NULL!
66,00	2,00	2,00	2,00	2,00	2,00	2,00	#NULL!	#NULL!
53,00	1,00	2,00	2,00	2,00	2,00	1,00	12,00	#NULL!
52,00	2,00	2,00	2,00	2,00	2,00	2,00	#NULL!	#NULL!
57,00	1,00	2,00	2,00	2,00	2,00	1,00	20,00	#NULL!
54,00	2,00	2,00	2,00	2,00	2,00	2,00	#NULL!	#NULL!
45,00	1,00	2,00	2,00	2,00	2,00	1,00	25,00	#NULL!
53,00	2,00	2,00	2,00	2,00	2,00	2,00	#NULL!	#NULL!
45,00	1,00	2,00	2,00	2,00	2,00	1,00	20,00	#NULL!
48,00	2,00	2,00	2,00	2,00	2,00	2,00	#NULL!	#NULL!
51,00	2,00	2,00	2,00	2,00	2,00	2,00	#NULL!	#NULL!
55,00	1,00	2,00	2,00	2,00	2,00	2,00	#NULL!	#NULL!
50,00	2,00	2,00	2,00	2,00	2,00	2,00	#NULL!	#NULL!
55,00	1,00	2,00	2,00	2,00	2,00	2,00	#NULL!	#NULL!
45,00	2,00	2,00	2,00	2,00	2,00	2,00	#NULL!	#NULL!
47,00	2,00	2,00	2,00	2,00	2,00	2,00	#NULL!	#NULL!
85,00	1,00	2,00	2,00	2,00	2,00	2,00	#NULL!	#NULL!
89,00	1,00	1,00	1,00	1,00	1,00	2,00	#NULL!	#NULL!
81,00	1,00	1,00	2,00	2,00	2,00	2,00	#NULL!	#NULL!
71,00	2,00	1,00	1,00	1,00	1,00	2,00	#NULL!	#NULL!
84,00	2,00	1,00	2,00	1,00	2,00	2,00	#NULL!	#NULL!
90,00	1,00	1,00	2,00	2,00	2,00	2,00	#NULL!	#NULL!
77,00	1,00	1,00	2,00	1,00	2,00	2,00	#NULL!	#NULL!
90,00	2,00	1,00	2,00	2,00	1,00	2,00	#NULL!	#NULL!
54,00	2,00	2,00	2,00	2,00	2,00	2,00	#NULL!	#NULL!
74,00	1,00	1,00	2,00	2,00	2,00	2,00	50,00	2,00
45,00	2,00	1,00	1,00	2,00	1,00	2,00	#NULL!	#NULL!
64,00	1,00	1,00	2,00	2,00	2,00	1,00	15,00	#NULL!
89,00	1,00	2,00	2,00	2,00	2,00	2,00	#NULL!	#NULL!
75,00	2,00	1,00	2,00	2,00	2,00	2,00	#NULL!	#NULL!
54,00	2,00	2,00	2,00	2,00	2,00	1,00	4,00	#NULL!
46,00	1,00	1,00	2,00	2,00	2,00	2,00	#NULL!	#NULL!
47,00	2,00	2,00	2,00	2,00	2,00	2,00	#NULL!	#NULL!
53,00	1,00	2,00	1,00	2,00	2,00	2,00	#NULL!	#NULL!
45,00	2,00	2,00	2,00	2,00	2,00	2,00	#NULL!	#NULL!
59,00	1,00	1,00	2,00	2,00	2,00	2,00	#NULL!	#NULL!
55,00	2,00	1,00	2,00	2,00	2,00	2,00	#NULL!	#NULL!

60,00	1,00	2,00	2,00	2,00	2,00	2,00	#NULL!	#NULL!
55,00	2,00	2,00	2,00	2,00	2,00	2,00	#NULL!	#NULL!
66,00	1,00	1,00	1,00	2,00	2,00	2,00	20,00	5,00
75,00	1,00	1,00	2,00	1,00	1,00	2,00	40,00	7,00
76,00	1,00	2,00	2,00	2,00	2,00	2,00	30,00	35,00
50,00	2,00	1,00	1,00	1,00	2,00	2,00	#NULL!	#NULL!
84,00	1,00	1,00	2,00	1,00	2,00	2,00	#NULL!	#NULL!
76,00	2,00	1,00	2,00	2,00	2,00	2,00	#NULL!	#NULL!
64,00	2,00	1,00	1,00	2,00	1,00	2,00	#NULL!	#NULL!
76,00	1,00	1,00	2,00	2,00	2,00	2,00	#NULL!	#NULL!
75,00	1,00	1,00	2,00	2,00	2,00	2,00	#NULL!	#NULL!
70,00	2,00	1,00	1,00	2,00	1,00	2,00	#NULL!	#NULL!
51,00	2,00	1,00	2,00	1,00	1,00	1,00	12,00	#NULL!
79,00	2,00	1,00	1,00	1,00	1,00	2,00	#NULL!	#NULL!
86,00	2,00	2,00	2,00	2,00	2,00	2,00	#NULL!	#NULL!
76,00	2,00	1,00	2,00	2,00	2,00	2,00	#NULL!	#NULL!
51,00	1,00	1,00	2,00	2,00	2,00	2,00	#NULL!	#NULL!
46,00	1,00	1,00	2,00	2,00	2,00	2,00	#NULL!	#NULL!
58,00	1,00	2,00	2,00	1,00	1,00	2,00	#NULL!	#NULL!
56,00	2,00	2,00	2,00	2,00	1,00	2,00	#NULL!	#NULL!
49,00	2,00	1,00	2,00	2,00	2,00	2,00	#NULL!	#NULL!
56,00	2,00	1,00	2,00	2,00	2,00	2,00	#NULL!	#NULL!
50,00	1,00	2,00	2,00	2,00	2,00	2,00	#NULL!	#NULL!
48,00	2,00	2,00	2,00	2,00	2,00	2,00	#NULL!	#NULL!
45,00	2,00	2,00	2,00	2,00	2,00	2,00	#NULL!	#NULL!
47,00	1,00	2,00	2,00	2,00	2,00	1,00	25,00	#NULL!
49,00	1,00	2,00	2,00	2,00	2,00	2,00	#NULL!	#NULL!
46,00	2,00	2,00	2,00	2,00	2,00	2,00	#NULL!	#NULL!
48,00	2,00	2,00	2,00	2,00	2,00	2,00	#NULL!	#NULL!
45,00	1,00	2,00	2,00	2,00	2,00	2,00	#NULL!	#NULL!
50,00	1,00	1,00	1,00	2,00	2,00	2,00	#NULL!	#NULL!
55,00	1,00	1,00	1,00	2,00	2,00	2,00	#NULL!	#NULL!
45,00	1,00	2,00	2,00	2,00	2,00	2,00	#NULL!	#NULL!
47,00	2,00	2,00	2,00	2,00	2,00	2,00	#NULL!	#NULL!
74,00	1,00	1,00	2,00	2,00	2,00	2,00	#NULL!	#NULL!
77,00	1,00	1,00	2,00	2,00	1,00	2,00	#NULL!	#NULL!
76,00	1,00	1,00	2,00	2,00	1,00	2,00	#NULL!	#NULL!
72,00	1,00	1,00	2,00	2,00	2,00	2,00	#NULL!	#NULL!
74,00	1,00	1,00	2,00	2,00	2,00	2,00	#NULL!	#NULL!
80,00	1,00	1,00	1,00	2,00	1,00	2,00	#NULL!	#NULL!
87,00	1,00	2,00	2,00	1,00	1,00	1,00	62,00	#NULL!
62,00	1,00	1,00	1,00	2,00	2,00	2,00	#NULL!	#NULL!
49,00	2,00	2,00	2,00	2,00	2,00	2,00	#NULL!	#NULL!
48,00	1,00	2,00	2,00	2,00	2,00	2,00	#NULL!	#NULL!
53,00	1,00	2,00	2,00	2,00	2,00	1,00	20,00	#NULL!
45,00	2,00	2,00	2,00	2,00	2,00	2,00	#NULL!	#NULL!
50,00	1,00	1,00	2,00	2,00	2,00	2,00	#NULL!	#NULL!
50,00	1,00	1,00	2,00	2,00	2,00	2,00	#NULL!	#NULL!
45,00	2,00	2,00	2,00	2,00	2,00	2,00	#NULL!	#NULL!
48,00	1,00	1,00	2,00	2,00	2,00	2,00	#NULL!	#NULL!

53,00	1,00	1,00	1,00	1,00	1,00	1,00	#NULL!	#NULL!
60,00	1,00	2,00	2,00	2,00	2,00	2,00	#NULL!	#NULL!
60,00	1,00	2,00	2,00	2,00	2,00	2,00	#NULL!	#NULL!
64,00	2,00	2,00	2,00	2,00	2,00	2,00	#NULL!	#NULL!
55,00	1,00	2,00	2,00	2,00	2,00	1,00	25,00	#NULL!
52,00	1,00	2,00	2,00	2,00	2,00	2,00	#NULL!	#NULL!
46,00	1,00	2,00	2,00	2,00	2,00	2,00	#NULL!	#NULL!
45,00	1,00	2,00	2,00	2,00	2,00	2,00	#NULL!	#NULL!
62,00	1,00	1,00	2,00	2,00	1,00	2,00	#NULL!	#NULL!
62,00	1,00	2,00	2,00	2,00	1,00	2,00	#NULL!	#NULL!
55,00	1,00	1,00	1,00	2,00	2,00	2,00	#NULL!	#NULL!
57,00	1,00	2,00	2,00	2,00	2,00	2,00	#NULL!	#NULL!
49,00	1,00	2,00	2,00	2,00	2,00	1,00	15,00	#NULL!
64,00	1,00	2,00	2,00	2,00	1,00	2,00	#NULL!	#NULL!
67,00	1,00	2,00	2,00	2,00	2,00	2,00	#NULL!	#NULL!
71,00	1,00	1,00	2,00	2,00	2,00	1,00	40,00	#NULL!
78,00	1,00	1,00	1,00	2,00	2,00	2,00	#NULL!	#NULL!
46,00	1,00	1,00	1,00	1,00	1,00	2,00	#NULL!	#NULL!
73,00	1,00	2,00	1,00	2,00	2,00	2,00	#NULL!	#NULL!
78,00	1,00	1,00	2,00	2,00	2,00	2,00	#NULL!	#NULL!
46,00	1,00	2,00	2,00	2,00	2,00	1,00	20,00	#NULL!
67,00	1,00	1,00	1,00	2,00	1,00	2,00	#NULL!	#NULL!
48,00	1,00	2,00	2,00	2,00	2,00	2,00	#NULL!	#NULL!
76,00	1,00	2,00	2,00	2,00	2,00	2,00	#NULL!	#NULL!
62,00	1,00	1,00	1,00	1,00	1,00	2,00	#NULL!	#NULL!
55,00	1,00	2,00	2,00	2,00	2,00	2,00	#NULL!	#NULL!
66,00	1,00	2,00	2,00	2,00	2,00	2,00	#NULL!	#NULL!
53,00	1,00	2,00	2,00	2,00	2,00	2,00	#NULL!	#NULL!
48,00	1,00	2,00	2,00	2,00	1,00	2,00	#NULL!	#NULL!
70,00	2,00	1,00	2,00	1,00	1,00	2,00	#NULL!	#NULL!
48,00	2,00	2,00	2,00	2,00	2,00	2,00	#NULL!	#NULL!
47,00	1,00	2,00	2,00	2,00	2,00	2,00	#NULL!	#NULL!
61,00	2,00	1,00	2,00	2,00	2,00	2,00	#NULL!	#NULL!
65,00	1,00	2,00	2,00	2,00	2,00	1,00	#NULL!	#NULL!
55,00	2,00	2,00	2,00	2,00	2,00	2,00	#NULL!	#NULL!
58,00	1,00	1,00	2,00	2,00	2,00	2,00	#NULL!	#NULL!
59,00	1,00	1,00	2,00	2,00	2,00	2,00	#NULL!	#NULL!
61,00	1,00	2,00	2,00	2,00	2,00	2,00	#NULL!	#NULL!
56,00	1,00	2,00	2,00	2,00	1,00	2,00	#NULL!	#NULL!
65,00	2,00	1,00	2,00	1,00	1,00	2,00	#NULL!	#NULL!
51,00	1,00	2,00	2,00	2,00	2,00	1,00	15,00	#NULL!
48,00	1,00	2,00	2,00	2,00	2,00	1,00	16,00	#NULL!
66,00	2,00	2,00	2,00	2,00	2,00	2,00	#NULL!	#NULL!
65,00	2,00	2,00	1,00	2,00	2,00	2,00	#NULL!	#NULL!
75,00	2,00	2,00	2,00	2,00	2,00	2,00	#NULL!	#NULL!
66,00	1,00	1,00	2,00	2,00	1,00	2,00	#NULL!	#NULL!
64,00	2,00	1,00	2,00	2,00	1,00	2,00	#NULL!	#NULL!
67,00	2,00	1,00	1,00	2,00	1,00	2,00	#NULL!	#NULL!
51,00	1,00	2,00	2,00	2,00	2,00	1,00	22,00	#NULL!
45,00	2,00	2,00	2,00	2,00	2,00	2,00	#NULL!	#NULL!

59,00	2,00	1,00	1,00	1,00	1,00	2,00	#NULL!	#NULL!
59,00	1,00	1,00	1,00	2,00	2,00	2,00	#NULL!	#NULL!
68,00	2,00	1,00	2,00	2,00	2,00	2,00	#NULL!	#NULL!
57,00	1,00	1,00	1,00	1,00	1,00	1,00	60,00	#NULL!
73,00	1,00	1,00	2,00	2,00	2,00	2,00	#NULL!	#NULL!
45,00	1,00	2,00	2,00	2,00	2,00	2,00	#NULL!	#NULL!
63,00	2,00	1,00	2,00	2,00	2,00	2,00	#NULL!	#NULL!
48,00	1,00	2,00	2,00	2,00	2,00	1,00	70,00	#NULL!
63,00	1,00	1,00	2,00	1,00	1,00	1,00	50,00	#NULL!
75,00	1,00	1,00	1,00	1,00	1,00	1,00	10,00	1,00
45,00	2,00	1,00	1,00	2,00	2,00	2,00	#NULL!	#NULL!
59,00	1,00	2,00	2,00	2,00	2,00	2,00	#NULL!	#NULL!
58,00	2,00	1,00	1,00	2,00	1,00	2,00	#NULL!	#NULL!
71,00	1,00	1,00	2,00	2,00	2,00	2,00	#NULL!	#NULL!
66,00	1,00	1,00	2,00	2,00	2,00	2,00	#NULL!	#NULL!
82,00	2,00	1,00	1,00	1,00	1,00	2,00	#NULL!	#NULL!
88,00	1,00	1,00	1,00	2,00	1,00	2,00	#NULL!	#NULL!
63,00	2,00	2,00	2,00	2,00	2,00	2,00	#NULL!	#NULL!
59,00	2,00	2,00	2,00	2,00	1,00	1,00	19,00	#NULL!
47,00	2,00	2,00	2,00	2,00	2,00	2,00	#NULL!	#NULL!
46,00	1,00	2,00	2,00	2,00	2,00	2,00	#NULL!	#NULL!
84,00	1,00	1,00	2,00	1,00	1,00	2,00	#NULL!	#NULL!
65,00	1,00	2,00	1,00	2,00	1,00	2,00	#NULL!	#NULL!
52,00	2,00	1,00	2,00	2,00	2,00	2,00	#NULL!	#NULL!
48,00	1,00	2,00	2,00	2,00	2,00	2,00	#NULL!	#NULL!
66,00	1,00	1,00	1,00	1,00	1,00	2,00	#NULL!	#NULL!
84,00	2,00	1,00	1,00	2,00	2,00	2,00	#NULL!	#NULL!
51,00	2,00	2,00	2,00	2,00	1,00	2,00	#NULL!	#NULL!
57,00	2,00	1,00	2,00	2,00	2,00	2,00	#NULL!	#NULL!
74,00	2,00	2,00	2,00	2,00	2,00	2,00	50,00	5,00
80,00	1,00	1,00	1,00	2,00	1,00	2,00	#NULL!	#NULL!
70,00	2,00	1,00	1,00	2,00	1,00	2,00	#NULL!	#NULL!
57,00	2,00	1,00	1,00	2,00	1,00	2,00	#NULL!	#NULL!
55,00	2,00	2,00	2,00	2,00	1,00	2,00	25,00	10,00
77,00	2,00	1,00	1,00	1,00	2,00	2,00	#NULL!	#NULL!
80,00	1,00	2,00	2,00	2,00	2,00	2,00	#NULL!	#NULL!
74,00	2,00	1,00	2,00	2,00	1,00	2,00	#NULL!	#NULL!
56,00	2,00	2,00	2,00	2,00	1,00	2,00	#NULL!	#NULL!
55,00	1,00	2,00	2,00	2,00	2,00	2,00	#NULL!	#NULL!
51,00	2,00	1,00	1,00	1,00	2,00	2,00	#NULL!	#NULL!
65,00	2,00	2,00	2,00	1,00	2,00	2,00	#NULL!	#NULL!
48,00	2,00	2,00	2,00	2,00	1,00	1,00	3,00	#NULL!
48,00	2,00	1,00	2,00	1,00	1,00	1,00	20,00	#NULL!
45,00	1,00	1,00	2,00	2,00	2,00	2,00	#NULL!	#NULL!
47,00	2,00	2,00	2,00	2,00	2,00	2,00	#NULL!	#NULL!
51,00	2,00	1,00	2,00	2,00	1,00	2,00	#NULL!	#NULL!
46,00	1,00	2,00	2,00	2,00	2,00	1,00	10,00	#NULL!
63,00	1,00	1,00	2,00	1,00	1,00	2,00	#NULL!	#NULL!
60,00	2,00	1,00	2,00	2,00	2,00	2,00	#NULL!	#NULL!
56,00	1,00	1,00	2,00	2,00	2,00	1,00	50,00	#NULL!

47,00	2,00	2,00	2,00	2,00	2,00	2,00	#NULL!	#NULL!
65,00	2,00	1,00	2,00	1,00	1,00	2,00	#NULL!	#NULL!
75,00	1,00	2,00	2,00	2,00	2,00	2,00	#NULL!	#NULL!
51,00	1,00	2,00	2,00	2,00	1,00	2,00	#NULL!	#NULL!
45,00	2,00	1,00	1,00	2,00	2,00	2,00	#NULL!	#NULL!
50,00	1,00	2,00	2,00	2,00	2,00	2,00	#NULL!	#NULL!
59,00	1,00	2,00	2,00	2,00	2,00	2,00	#NULL!	#NULL!
56,00	2,00	1,00	2,00	1,00	1,00	2,00	#NULL!	#NULL!
68,00	1,00	2,00	2,00	2,00	2,00	2,00	#NULL!	#NULL!
52,00	1,00	2,00	2,00	2,00	2,00	1,00	28,00	#NULL!
80,00	1,00	1,00	2,00	1,00	1,00	1,00	52,00	#NULL!
76,00	2,00	1,00	1,00	2,00	1,00	2,00	#NULL!	#NULL!
50,00	2,00	1,00	2,00	2,00	2,00	2,00	#NULL!	#NULL!
47,00	1,00	2,00	2,00	2,00	2,00	1,00	22,00	#NULL!
45,00	2,00	1,00	2,00	2,00	2,00	2,00	#NULL!	#NULL!
68,00	2,00	1,00	1,00	2,00	1,00	2,00	#NULL!	#NULL!
54,00	1,00	2,00	2,00	2,00	2,00	2,00	#NULL!	#NULL!
64,00	1,00	2,00	2,00	2,00	2,00	2,00	#NULL!	#NULL!
60,00	2,00	1,00	1,00	2,00	2,00	2,00	#NULL!	#NULL!
69,00	1,00	2,00	2,00	1,00	2,00	1,00	45,00	#NULL!
72,00	2,00	1,00	1,00	2,00	2,00	2,00	#NULL!	#NULL!
52,00	1,00	1,00	2,00	2,00	2,00	1,00	15,00	#NULL!
45,00	1,00	2,00	2,00	2,00	2,00	2,00	#NULL!	#NULL!
65,00	1,00	2,00	2,00	2,00	2,00	2,00	#NULL!	#NULL!
52,00	1,00	2,00	2,00	2,00	2,00	2,00	#NULL!	#NULL!
50,00	1,00	1,00	1,00	2,00	1,00	2,00	#NULL!	#NULL!
65,00	2,00	1,00	1,00	2,00	1,00	1,00	30,00	#NULL!
66,00	1,00	2,00	2,00	2,00	2,00	1,00	25,00	#NULL!
60,00	2,00	1,00	2,00	2,00	2,00	2,00	#NULL!	#NULL!
54,00	1,00	1,00	2,00	2,00	2,00	1,00	35,00	#NULL!
64,00	2,00	1,00	1,00	2,00	2,00	2,00	#NULL!	#NULL!
54,00	1,00	2,00	2,00	2,00	2,00	2,00	#NULL!	#NULL!
76,00	2,00	2,00	2,00	2,00	2,00	2,00	#NULL!	#NULL!
78,00	1,00	2,00	1,00	1,00	1,00	1,00	30,00	#NULL!
81,00	2,00	1,00	2,00	2,00	2,00	2,00	#NULL!	#NULL!
57,00	1,00	2,00	1,00	2,00	2,00	2,00	#NULL!	#NULL!
45,00	2,00	2,00	2,00	2,00	2,00	2,00	#NULL!	#NULL!
49,00	1,00	1,00	2,00	1,00	1,00	1,00	30,00	#NULL!
50,00	2,00	1,00	1,00	2,00	1,00	2,00	#NULL!	#NULL!
54,00	1,00	2,00	2,00	2,00	2,00	1,00	15,00	#NULL!
56,00	1,00	2,00	2,00	2,00	2,00	1,00	30,00	#NULL!
87,00	1,00	1,00	2,00	1,00	2,00	2,00	#NULL!	#NULL!
80,00	1,00	1,00	2,00	1,00	2,00	2,00	#NULL!	#NULL!
48,00	2,00	2,00	2,00	2,00	2,00	1,00	15,00	#NULL!
60,00	2,00	1,00	2,00	2,00	2,00	1,00	40,00	#NULL!
46,00	1,00	1,00	2,00	2,00	1,00	2,00	#NULL!	#NULL!
49,00	1,00	1,00	1,00	2,00	2,00	2,00	#NULL!	#NULL!
49,00	2,00	1,00	1,00	2,00	2,00	1,00	15,00	#NULL!
49,00	1,00	1,00	1,00	2,00	2,00	2,00	#NULL!	#NULL!
56,00	2,00	1,00	2,00	1,00	2,00	2,00	#NULL!	#NULL!

61,00	1,00	2,00	2,00	2,00	2,00	2,00	#NULL!	#NULL!
72,00	2,00	2,00	2,00	2,00	1,00	2,00	#NULL!	#NULL!
48,00	1,00	2,00	2,00	2,00	2,00	1,00	6,00	#NULL!
55,00	1,00	2,00	2,00	2,00	1,00	1,00	8,00	#NULL!
73,00	2,00	1,00	1,00	1,00	1,00	2,00	#NULL!	#NULL!
70,00	2,00	2,00	2,00	2,00	1,00	2,00	#NULL!	#NULL!
57,00	2,00	1,00	1,00	2,00	2,00	2,00	#NULL!	#NULL!
66,00	1,00	1,00	2,00	1,00	1,00	2,00	20,00	30,00
64,00	2,00	2,00	2,00	2,00	2,00	2,00	#NULL!	#NULL!
76,00	2,00	2,00	2,00	2,00	2,00	2,00	#NULL!	#NULL!
79,00	2,00	2,00	2,00	2,00	2,00	2,00	#NULL!	#NULL!
55,00	2,00	1,00	1,00	1,00	1,00	2,00	#NULL!	#NULL!
58,00	1,00	2,00	2,00	2,00	1,00	2,00	#NULL!	#NULL!
50,00	2,00	2,00	2,00	1,00	2,00	1,00	26,00	#NULL!
56,00	1,00	2,00	2,00	2,00	1,00	2,00	#NULL!	#NULL!
48,00	1,00	1,00	2,00	2,00	2,00	1,00	20,00	#NULL!
45,00	2,00	2,00	2,00	2,00	2,00	2,00	#NULL!	#NULL!
50,00	2,00	1,00	1,00	2,00	2,00	2,00	#NULL!	#NULL!
52,00	1,00	1,00	1,00	2,00	2,00	2,00	#NULL!	#NULL!
87,00	1,00	1,00	2,00	2,00	2,00	1,00	100,00	20,00
58,00	2,00	1,00	1,00	2,00	1,00	2,00	#NULL!	#NULL!
90,00	1,00	1,00	1,00	2,00	1,00	1,00	70,00	#NULL!
70,00	1,00	1,00	2,00	2,00	2,00	2,00	#NULL!	#NULL!
49,00	1,00	1,00	2,00	1,00	1,00	1,00	20,00	#NULL!
46,00	1,00	2,00	2,00	2,00	2,00	2,00	#NULL!	#NULL!
46,00	2,00	2,00	2,00	2,00	2,00	2,00	#NULL!	#NULL!
47,00	2,00	2,00	1,00	2,00	2,00	2,00	#NULL!	#NULL!
70,00	2,00	1,00	2,00	2,00	2,00	2,00	#NULL!	#NULL!
76,00	1,00	1,00	1,00	1,00	1,00	2,00	#NULL!	#NULL!
56,00	2,00	1,00	2,00	1,00	1,00	2,00	#NULL!	#NULL!
60,00	1,00	2,00	2,00	2,00	2,00	2,00	#NULL!	#NULL!
63,00	2,00	1,00	2,00	2,00	2,00	2,00	#NULL!	#NULL!
65,00	1,00	2,00	2,00	2,00	2,00	2,00	#NULL!	#NULL!
62,00	2,00	1,00	2,00	2,00	2,00	2,00	#NULL!	#NULL!
68,00	1,00	2,00	2,00	2,00	2,00	2,00	#NULL!	#NULL!
72,00	2,00	2,00	2,00	2,00	2,00	2,00	#NULL!	#NULL!
50,00	2,00	1,00	2,00	2,00	1,00	2,00	#NULL!	#NULL!
50,00	2,00	1,00	2,00	2,00	2,00	2,00	#NULL!	#NULL!
55,00	1,00	1,00	2,00	1,00	1,00	1,00	30,00	#NULL!
75,00	2,00	1,00	2,00	2,00	2,00	2,00	#NULL!	#NULL!
49,00	2,00	2,00	2,00	2,00	2,00	2,00	#NULL!	#NULL!
58,00	1,00	2,00	2,00	2,00	2,00	1,00	15,00	#NULL!
48,00	2,00	2,00	2,00	2,00	2,00	2,00	#NULL!	#NULL!
71,00	2,00	2,00	2,00	2,00	2,00	2,00	#NULL!	#NULL!
76,00	1,00	2,00	2,00	2,00	2,00	2,00	#NULL!	#NULL!
60,00	1,00	2,00	2,00	2,00	2,00	2,00	#NULL!	#NULL!
49,00	1,00	2,00	2,00	2,00	2,00	2,00	#NULL!	#NULL!
46,00	2,00	2,00	2,00	2,00	2,00	2,00	#NULL!	#NULL!
45,00	1,00	2,00	2,00	2,00	2,00	2,00	#NULL!	#NULL!
46,00	1,00	2,00	2,00	2,00	2,00	2,00	#NULL!	#NULL!

45,00	2,00	2,00	2,00	2,00	2,00	2,00	#NULL!	#NULL!
46,00	1,00	2,00	2,00	2,00	2,00	2,00	#NULL!	#NULL!
45,00	2,00	2,00	2,00	2,00	2,00	2,00	#NULL!	#NULL!
50,00	1,00	1,00	2,00	2,00	2,00	2,00	#NULL!	#NULL!
49,00	2,00	2,00	2,00	2,00	2,00	2,00	#NULL!	#NULL!
47,00	1,00	2,00	2,00	2,00	2,00	2,00	#NULL!	#NULL!
46,00	2,00	2,00	2,00	2,00	2,00	2,00	#NULL!	#NULL!
45,00	2,00	2,00	2,00	2,00	2,00	1,00	20,00	#NULL!
46,00	1,00	1,00	2,00	2,00	2,00	1,00	10,00	#NULL!
47,00	1,00	2,00	2,00	2,00	2,00	2,00	#NULL!	#NULL!
46,00	2,00	2,00	2,00	2,00	2,00	2,00	#NULL!	#NULL!
46,00	1,00	2,00	2,00	2,00	2,00	2,00	#NULL!	#NULL!
45,00	2,00	1,00	2,00	2,00	2,00	2,00	#NULL!	#NULL!
45,00	2,00	2,00	2,00	2,00	2,00	2,00	#NULL!	#NULL!
46,00	1,00	2,00	2,00	2,00	2,00	2,00	#NULL!	#NULL!
45,00	2,00	2,00	2,00	2,00	2,00	2,00	#NULL!	#NULL!
46,00	1,00	2,00	2,00	2,00	2,00	2,00	#NULL!	#NULL!
45,00	1,00	2,00	2,00	2,00	2,00	2,00	#NULL!	#NULL!
46,00	2,00	2,00	2,00	2,00	2,00	2,00	#NULL!	#NULL!
45,00	2,00	2,00	2,00	2,00	2,00	2,00	#NULL!	#NULL!
46,00	1,00	2,00	2,00	2,00	2,00	2,00	#NULL!	#NULL!
45,00	2,00	2,00	2,00	2,00	2,00	2,00	#NULL!	#NULL!
45,00	1,00	2,00	2,00	2,00	2,00	2,00	#NULL!	#NULL!
45,00	2,00	2,00	2,00	2,00	2,00	2,00	#NULL!	#NULL!
45,00	2,00	2,00	2,00	2,00	2,00	2,00	#NULL!	#NULL!
57,00	2,00	2,00	2,00	2,00	2,00	2,00	#NULL!	#NULL!
58,00	1,00	2,00	2,00	2,00	2,00	2,00	#NULL!	#NULL!
55,00	1,00	2,00	2,00	2,00	2,00	2,00	#NULL!	#NULL!
60,00	2,00	2,00	2,00	2,00	2,00	2,00	#NULL!	#NULL!
58,00	2,00	2,00	2,00	2,00	2,00	2,00	#NULL!	#NULL!
52,00	2,00	2,00	2,00	2,00	2,00	2,00	#NULL!	#NULL!
50,00	2,00	2,00	2,00	2,00	2,00	2,00	#NULL!	#NULL!
56,00	1,00	2,00	2,00	2,00	2,00	2,00	#NULL!	#NULL!
55,00	1,00	2,00	2,00	2,00	2,00	2,00	#NULL!	#NULL!
50,00	2,00	2,00	2,00	2,00	2,00	2,00	#NULL!	#NULL!
48,00	1,00	2,00	2,00	2,00	2,00	2,00	#NULL!	#NULL!
45,00	2,00	2,00	2,00	2,00	2,00	2,00	#NULL!	#NULL!
49,00	1,00	2,00	2,00	2,00	2,00	1,00	20,00	#NULL!
48,00	2,00	2,00	2,00	2,00	2,00	2,00	#NULL!	#NULL!
45,00	2,00	2,00	2,00	2,00	2,00	2,00	#NULL!	#NULL!
46,00	1,00	2,00	2,00	2,00	2,00	2,00	#NULL!	#NULL!
52,00	2,00	1,00	1,00	2,00	1,00	1,00	30,00	#NULL!
50,00	2,00	1,00	2,00	2,00	2,00	2,00	#NULL!	#NULL!
43,00	2,00	2,00	1,00	2,00	2,00	1,00	15,00	#NULL!
76,00	2,00	1,00	2,00	2,00	2,00	2,00	#NULL!	#NULL!
49,00	1,00	1,00	2,00	2,00	2,00	2,00	#NULL!	#NULL!
54,00	2,00	2,00	1,00	2,00	2,00	2,00	#NULL!	#NULL!
48,00	2,00	2,00	2,00	2,00	2,00	2,00	#NULL!	#NULL!
52,00	2,00	2,00	2,00	2,00	2,00	1,00	10,00	#NULL!
46,00	2,00	2,00	2,00	2,00	2,00	2,00	#NULL!	#NULL!

49,00	1,00	2,00	2,00	2,00	2,00	2,00	#NULL!	#NULL!
54,00	2,00	1,00	1,00	2,00	2,00	2,00	#NULL!	#NULL!
54,00	1,00	1,00	2,00	2,00	2,00	2,00	#NULL!	#NULL!
50,00	1,00	1,00	2,00	2,00	2,00	2,00	#NULL!	#NULL!
49,00	1,00	1,00	2,00	2,00	2,00	2,00	#NULL!	#NULL!
48,00	2,00	2,00	1,00	2,00	2,00	2,00	#NULL!	#NULL!
50,00	1,00	1,00	2,00	2,00	2,00	2,00	#NULL!	#NULL!
61,00	2,00	2,00	2,00	2,00	2,00	2,00	#NULL!	#NULL!
47,00	2,00	2,00	2,00	2,00	2,00	2,00	#NULL!	#NULL!
47,00	2,00	2,00	2,00	2,00	2,00	2,00	#NULL!	#NULL!
45,00	1,00	2,00	2,00	2,00	2,00	2,00	#NULL!	#NULL!
45,00	2,00	2,00	2,00	2,00	2,00	2,00	#NULL!	#NULL!
45,00	2,00	2,00	2,00	2,00	2,00	2,00	#NULL!	#NULL!
46,00	1,00	2,00	2,00	2,00	2,00	2,00	#NULL!	#NULL!
45,00	2,00	2,00	2,00	2,00	2,00	2,00	#NULL!	#NULL!
48,00	2,00	1,00	2,00	2,00	2,00	2,00	#NULL!	#NULL!
57,00	2,00	2,00	2,00	2,00	2,00	2,00	#NULL!	#NULL!
74,00	2,00	2,00	2,00	1,00	2,00	2,00	#NULL!	#NULL!
80,00	2,00	1,00	1,00	1,00	1,00	2,00	#NULL!	#NULL!
50,00	1,00	2,00	2,00	2,00	2,00	2,00	#NULL!	#NULL!
45,00	2,00	2,00	2,00	2,00	2,00	2,00	#NULL!	#NULL!
60,00	1,00	2,00	2,00	2,00	2,00	2,00	#NULL!	#NULL!
65,00	1,00	2,00	2,00	2,00	2,00	2,00	#NULL!	#NULL!
49,00	1,00	2,00	2,00	2,00	2,00	2,00	#NULL!	#NULL!
45,00	2,00	2,00	2,00	2,00	2,00	2,00	#NULL!	#NULL!
63,00	1,00	2,00	2,00	2,00	2,00	1,00	40,00	#NULL!
45,00	1,00	2,00	1,00	2,00	2,00	1,00	30,00	#NULL!
47,00	2,00	1,00	2,00	2,00	2,00	2,00	#NULL!	#NULL!
48,00	2,00	2,00	2,00	2,00	2,00	2,00	#NULL!	#NULL!
45,00	2,00	2,00	2,00	2,00	2,00	2,00	#NULL!	#NULL!
45,00	1,00	2,00	2,00	2,00	2,00	2,00	#NULL!	#NULL!
46,00	2,00	2,00	2,00	2,00	2,00	2,00	#NULL!	#NULL!
47,00	2,00	2,00	2,00	2,00	2,00	2,00	#NULL!	#NULL!
45,00	1,00	2,00	2,00	2,00	2,00	2,00	#NULL!	#NULL!
48,00	2,00	2,00	2,00	2,00	2,00	2,00	#NULL!	#NULL!
60,00	2,00	1,00	1,00	2,00	2,00	2,00	#NULL!	#NULL!
60,00	1,00	2,00	2,00	2,00	2,00	2,00	#NULL!	#NULL!
83,00	2,00	1,00	2,00	2,00	2,00	2,00	#NULL!	#NULL!
60,00	1,00	2,00	2,00	2,00	2,00	1,00	30,00	#NULL!
50,00	1,00	2,00	2,00	2,00	2,00	1,00	20,00	#NULL!
45,00	2,00	2,00	2,00	2,00	2,00	2,00	#NULL!	#NULL!
54,00	1,00	1,00	2,00	2,00	1,00	1,00	18,00	#NULL!
55,00	2,00	2,00	2,00	1,00	2,00	1,00	40,00	#NULL!
50,00	1,00	2,00	2,00	2,00	2,00	2,00	#NULL!	#NULL!
47,00	2,00	2,00	2,00	2,00	2,00	2,00	#NULL!	#NULL!
50,00	2,00	2,00	2,00	2,00	2,00	2,00	#NULL!	#NULL!
53,00	1,00	2,00	2,00	2,00	2,00	2,00	#NULL!	#NULL!
52,00	2,00	2,00	2,00	2,00	2,00	2,00	#NULL!	#NULL!
53,00	1,00	2,00	2,00	2,00	2,00	2,00	#NULL!	#NULL!
54,00	1,00	2,00	2,00	2,00	2,00	2,00	#NULL!	#NULL!

50,00	2,00	2,00	2,00	2,00	2,00	2,00	#NULL!	#NULL!
46,00	2,00	2,00	2,00	2,00	2,00	2,00	#NULL!	#NULL!
48,00	1,00	2,00	2,00	2,00	2,00	2,00	#NULL!	#NULL!
53,00	1,00	2,00	2,00	2,00	2,00	2,00	#NULL!	#NULL!
50,00	2,00	2,00	2,00	2,00	2,00	2,00	#NULL!	#NULL!
50,00	1,00	2,00	2,00	2,00	2,00	2,00	#NULL!	#NULL!
45,00	2,00	2,00	2,00	2,00	2,00	2,00	#NULL!	#NULL!
51,00	2,00	2,00	2,00	2,00	2,00	2,00	#NULL!	#NULL!
55,00	1,00	2,00	2,00	2,00	2,00	2,00	#NULL!	#NULL!
55,00	1,00	2,00	2,00	2,00	2,00	2,00	#NULL!	#NULL!
54,00	1,00	2,00	2,00	2,00	2,00	2,00	#NULL!	#NULL!
50,00	1,00	2,00	2,00	2,00	2,00	2,00	#NULL!	#NULL!
45,00	2,00	2,00	2,00	2,00	2,00	2,00	#NULL!	#NULL!
50,00	1,00	2,00	2,00	2,00	2,00	2,00	#NULL!	#NULL!
47,00	1,00	2,00	2,00	2,00	2,00	2,00	#NULL!	#NULL!
50,00	1,00	2,00	2,00	2,00	2,00	2,00	#NULL!	#NULL!
45,00	2,00	2,00	2,00	2,00	2,00	2,00	#NULL!	#NULL!
60,00	1,00	2,00	2,00	2,00	2,00	2,00	#NULL!	#NULL!
54,00	2,00	2,00	2,00	2,00	2,00	2,00	#NULL!	#NULL!
50,00	2,00	2,00	2,00	2,00	2,00	2,00	#NULL!	#NULL!
52,00	1,00	2,00	2,00	2,00	2,00	2,00	#NULL!	#NULL!
47,00	1,00	2,00	2,00	2,00	2,00	2,00	#NULL!	#NULL!
45,00	1,00	2,00	2,00	2,00	2,00	2,00	#NULL!	#NULL!
50,00	1,00	2,00	2,00	2,00	2,00	2,00	#NULL!	#NULL!
44,00	2,00	2,00	2,00	2,00	2,00	2,00	#NULL!	#NULL!
63,00	2,00	2,00	2,00	2,00	2,00	2,00	#NULL!	#NULL!
65,00	2,00	2,00	2,00	2,00	2,00	2,00	#NULL!	#NULL!
47,00	2,00	2,00	2,00	2,00	2,00	2,00	#NULL!	#NULL!
51,00	1,00	2,00	2,00	2,00	2,00	2,00	#NULL!	#NULL!
45,00	2,00	1,00	2,00	2,00	2,00	2,00	#NULL!	#NULL!
47,00	1,00	1,00	2,00	2,00	2,00	2,00	#NULL!	#NULL!
47,00	1,00	2,00	2,00	2,00	2,00	2,00	#NULL!	#NULL!
48,00	1,00	2,00	2,00	2,00	2,00	2,00	#NULL!	#NULL!
47,00	2,00	2,00	2,00	2,00	2,00	2,00	#NULL!	#NULL!
45,00	2,00	2,00	2,00	2,00	2,00	2,00	#NULL!	#NULL!
45,00	2,00	2,00	2,00	2,00	2,00	2,00	#NULL!	#NULL!
47,00	1,00	2,00	2,00	2,00	2,00	2,00	#NULL!	#NULL!
43,00	2,00	2,00	2,00	2,00	2,00	2,00	#NULL!	#NULL!
55,00	1,00	2,00	2,00	2,00	2,00	2,00	#NULL!	#NULL!
55,00	2,00	2,00	2,00	2,00	2,00	2,00	#NULL!	#NULL!
55,00	2,00	2,00	2,00	2,00	2,00	2,00	#NULL!	#NULL!
47,00	1,00	2,00	2,00	2,00	2,00	2,00	#NULL!	#NULL!
49,00	1,00	2,00	2,00	2,00	2,00	2,00	#NULL!	#NULL!
70,00	1,00	2,00	2,00	2,00	2,00	2,00	#NULL!	#NULL!
52,00	1,00	2,00	2,00	2,00	2,00	2,00	#NULL!	#NULL!
58,00	2,00	2,00	2,00	2,00	2,00	2,00	#NULL!	#NULL!
63,00	1,00	2,00	2,00	2,00	2,00	2,00	#NULL!	#NULL!
46,00	1,00	2,00	2,00	2,00	2,00	2,00	#NULL!	#NULL!
45,00	2,00	2,00	2,00	2,00	2,00	2,00	#NULL!	#NULL!
45,00	1,00	2,00	2,00	2,00	2,00	2,00	#NULL!	#NULL!

60,00	1,00	2,00	2,00	2,00	2,00	2,00	#NULL!	#NULL!
45,00	2,00	2,00	2,00	2,00	2,00	2,00	#NULL!	#NULL!
56,00	1,00	2,00	2,00	2,00	2,00	2,00	#NULL!	#NULL!
80,00	2,00	2,00	2,00	2,00	2,00	2,00	#NULL!	#NULL!
61,00	1,00	2,00	2,00	2,00	2,00	2,00	#NULL!	#NULL!
59,00	2,00	2,00	2,00	2,00	2,00	2,00	#NULL!	#NULL!
45,00	1,00	2,00	2,00	2,00	2,00	2,00	#NULL!	#NULL!
53,00	2,00	2,00	2,00	2,00	1,00	2,00	#NULL!	#NULL!
53,00	1,00	2,00	1,00	2,00	2,00	1,00	40,00	#NULL!
53,00	2,00	1,00	2,00	1,00	2,00	2,00	#NULL!	#NULL!
63,00	1,00	1,00	1,00	1,00	2,00	2,00	#NULL!	#NULL!
45,00	2,00	2,00	2,00	2,00	2,00	2,00	#NULL!	#NULL!
46,00	1,00	2,00	2,00	2,00	2,00	2,00	#NULL!	#NULL!
55,00	2,00	2,00	2,00	2,00	2,00	1,00	13,00	#NULL!
49,00	2,00	2,00	2,00	2,00	1,00	2,00	#NULL!	#NULL!
48,00	1,00	2,00	2,00	2,00	2,00	2,00	#NULL!	#NULL!
45,00	2,00	2,00	2,00	2,00	2,00	2,00	#NULL!	#NULL!
61,00	2,00	1,00	2,00	1,00	2,00	2,00	#NULL!	#NULL!
50,00	1,00	2,00	1,00	2,00	1,00	1,00	30,00	#NULL!
49,00	2,00	2,00	2,00	2,00	2,00	2,00	#NULL!	#NULL!
56,00	1,00	2,00	2,00	2,00	2,00	2,00	#NULL!	#NULL!
49,00	2,00	2,00	2,00	1,00	2,00	1,00	30,00	#NULL!
55,00	1,00	2,00	2,00	2,00	2,00	1,00	30,00	#NULL!
45,00	2,00	1,00	2,00	2,00	2,00	2,00	#NULL!	#NULL!
54,00	2,00	1,00	2,00	2,00	1,00	2,00	#NULL!	#NULL!
51,00	1,00	2,00	2,00	2,00	2,00	2,00	#NULL!	#NULL!
90,00	1,00	1,00	1,00	1,00	2,00	2,00	#NULL!	#NULL!
45,00	1,00	2,00	2,00	2,00	2,00	2,00	#NULL!	#NULL!
45,00	2,00	2,00	2,00	2,00	2,00	2,00	#NULL!	#NULL!
45,00	2,00	2,00	2,00	2,00	2,00	2,00	#NULL!	#NULL!
54,00	1,00	2,00	2,00	2,00	2,00	2,00	#NULL!	#NULL!
49,00	2,00	2,00	2,00	2,00	2,00	2,00	#NULL!	#NULL!
54,00	1,00	2,00	2,00	2,00	2,00	2,00	#NULL!	#NULL!
56,00	2,00	2,00	2,00	2,00	2,00	2,00	#NULL!	#NULL!
60,00	1,00	2,00	2,00	2,00	2,00	2,00	#NULL!	#NULL!
48,00	1,00	2,00	2,00	2,00	2,00	2,00	#NULL!	#NULL!
60,00	2,00	2,00	2,00	2,00	2,00	2,00	#NULL!	#NULL!
53,00	1,00	2,00	2,00	2,00	2,00	2,00	#NULL!	#NULL!
56,00	2,00	1,00	2,00	2,00	2,00	2,00	#NULL!	#NULL!
55,00	2,00	1,00	2,00	2,00	2,00	2,00	#NULL!	#NULL!
62,00	1,00	1,00	1,00	1,00	1,00	2,00	40,00	2,00
59,00	2,00	1,00	2,00	2,00	2,00	2,00	#NULL!	#NULL!
62,00	1,00	1,00	2,00	2,00	2,00	2,00	40,00	10,00
47,00	2,00	2,00	2,00	2,00	2,00	2,00	#NULL!	#NULL!
50,00	1,00	2,00	2,00	2,00	2,00	2,00	#NULL!	#NULL!
60,00	2,00	1,00	1,00	2,00	1,00	2,00	#NULL!	#NULL!
65,00	2,00	1,00	1,00	2,00	1,00	2,00	#NULL!	#NULL!
84,00	1,00	1,00	1,00	2,00	2,00	2,00	#NULL!	#NULL!
75,00	1,00	2,00	1,00	1,00	2,00	2,00	30,00	20,00
65,00	1,00	1,00	2,00	1,00	2,00	2,00	#NULL!	#NULL!

72,00	2,00	1,00	2,00	2,00	2,00	2,00	#NULL!	#NULL!
82,00	2,00	1,00	1,00	1,00	2,00	2,00	#NULL!	#NULL!
60,00	1,00	2,00	2,00	2,00	2,00	2,00	#NULL!	#NULL!
46,00	2,00	2,00	2,00	2,00	2,00	2,00	#NULL!	#NULL!
45,00	2,00	2,00	2,00	2,00	2,00	2,00	#NULL!	#NULL!
54,00	1,00	2,00	2,00	2,00	2,00	1,00	28,00	#NULL!
58,00	2,00	1,00	2,00	2,00	2,00	2,00	#NULL!	#NULL!
46,00	1,00	1,00	2,00	2,00	2,00	2,00	#NULL!	#NULL!
45,00	1,00	2,00	2,00	2,00	2,00	2,00	#NULL!	#NULL!
78,00	2,00	1,00	2,00	2,00	2,00	2,00	#NULL!	#NULL!
82,00	2,00	1,00	1,00	2,00	1,00	2,00	#NULL!	#NULL!
64,00	1,00	2,00	2,00	2,00	2,00	1,00	20,00	#NULL!
82,00	2,00	1,00	1,00	2,00	2,00	2,00	#NULL!	#NULL!
46,00	1,00	2,00	2,00	2,00	2,00	2,00	#NULL!	#NULL!
48,00	2,00	2,00	2,00	2,00	2,00	2,00	#NULL!	#NULL!
47,00	1,00	1,00	2,00	1,00	2,00	1,00	10,00	#NULL!
47,00	2,00	2,00	2,00	2,00	2,00	2,00	#NULL!	#NULL!
47,00	1,00	2,00	1,00	2,00	2,00	2,00	#NULL!	#NULL!
45,00	2,00	1,00	1,00	2,00	1,00	2,00	#NULL!	#NULL!
50,00	1,00	2,00	1,00	2,00	2,00	2,00	#NULL!	#NULL!
48,00	2,00	2,00	1,00	2,00	2,00	2,00	#NULL!	#NULL!
49,00	2,00	1,00	2,00	1,00	1,00	1,00	30,00	#NULL!
56,00	1,00	1,00	2,00	1,00	2,00	1,00	20,00	#NULL!
45,00	2,00	1,00	2,00	1,00	2,00	2,00	#NULL!	#NULL!
78,00	2,00	1,00	1,00	2,00	2,00	2,00	#NULL!	#NULL!
56,00	2,00	1,00	2,00	1,00	1,00	2,00	#NULL!	#NULL!
59,00	1,00	1,00	2,00	2,00	2,00	2,00	#NULL!	#NULL!
45,00	1,00	2,00	2,00	2,00	1,00	1,00	50,00	#NULL!
60,00	2,00	2,00	2,00	2,00	2,00	2,00	#NULL!	#NULL!
48,00	1,00	2,00	2,00	2,00	2,00	2,00	#NULL!	#NULL!
52,00	1,00	1,00	2,00	2,00	2,00	2,00	#NULL!	#NULL!
43,00	2,00	2,00	2,00	2,00	2,00	2,00	#NULL!	#NULL!
43,00	2,00	2,00	2,00	2,00	2,00	2,00	#NULL!	#NULL!
47,00	1,00	2,00	2,00	2,00	2,00	2,00	#NULL!	#NULL!
48,00	1,00	2,00	2,00	2,00	2,00	2,00	#NULL!	#NULL!
45,00	2,00	2,00	2,00	2,00	2,00	2,00	#NULL!	#NULL!
70,00	1,00	2,00	2,00	2,00	2,00	2,00	#NULL!	#NULL!
48,00	2,00	2,00	2,00	2,00	2,00	2,00	#NULL!	#NULL!
56,00	1,00	1,00	2,00	2,00	2,00	2,00	#NULL!	#NULL!
45,00	2,00	2,00	2,00	2,00	2,00	2,00	#NULL!	#NULL!
49,00	1,00	2,00	2,00	2,00	2,00	2,00	#NULL!	#NULL!
56,00	1,00	1,00	2,00	2,00	2,00	1,00	40,00	#NULL!
66,00	1,00	1,00	2,00	1,00	2,00	1,00	40,00	#NULL!
58,00	1,00	2,00	2,00	2,00	2,00	2,00	#NULL!	#NULL!
50,00	2,00	2,00	2,00	2,00	2,00	2,00	#NULL!	#NULL!
49,00	1,00	2,00	2,00	2,00	2,00	2,00	#NULL!	#NULL!
52,00	1,00	2,00	2,00	2,00	2,00	2,00	#NULL!	#NULL!
44,00	1,00	1,00	2,00	2,00	2,00	1,00	20,00	#NULL!
46,00	2,00	2,00	2,00	2,00	2,00	2,00	#NULL!	#NULL!
48,00	1,00	2,00	2,00	2,00	2,00	2,00	#NULL!	#NULL!

45,00	2,00	2,00	2,00	2,00	2,00	2,00	#NULL!	#NULL!
45,00	1,00	2,00	2,00	2,00	2,00	2,00	#NULL!	#NULL!
55,00	2,00	2,00	2,00	2,00	2,00	2,00	#NULL!	#NULL!
65,00	2,00	2,00	2,00	2,00	2,00	2,00	#NULL!	#NULL!
70,00	1,00	2,00	2,00	2,00	2,00	2,00	#NULL!	#NULL!
70,00	1,00	2,00	2,00	2,00	2,00	2,00	#NULL!	#NULL!
65,00	2,00	2,00	2,00	2,00	2,00	2,00	#NULL!	#NULL!
60,00	1,00	2,00	2,00	2,00	2,00	2,00	#NULL!	#NULL!
55,00	2,00	2,00	2,00	2,00	2,00	2,00	#NULL!	#NULL!
55,00	2,00	2,00	2,00	2,00	2,00	2,00	#NULL!	#NULL!
50,00	2,00	2,00	2,00	2,00	2,00	2,00	#NULL!	#NULL!
51,00	2,00	2,00	2,00	2,00	2,00	2,00	#NULL!	#NULL!
55,00	1,00	2,00	2,00	2,00	2,00	2,00	#NULL!	#NULL!
53,00	2,00	2,00	2,00	2,00	2,00	2,00	#NULL!	#NULL!
55,00	1,00	2,00	2,00	2,00	2,00	2,00	#NULL!	#NULL!
45,00	1,00	2,00	2,00	2,00	2,00	2,00	#NULL!	#NULL!
45,00	1,00	2,00	2,00	2,00	2,00	2,00	#NULL!	#NULL!
50,00	2,00	2,00	2,00	2,00	2,00	2,00	#NULL!	#NULL!
55,00	1,00	2,00	2,00	2,00	2,00	2,00	#NULL!	#NULL!
45,00	1,00	2,00	2,00	2,00	2,00	2,00	#NULL!	#NULL!
53,00	2,00	2,00	2,00	2,00	2,00	2,00	#NULL!	#NULL!
45,00	1,00	2,00	2,00	2,00	2,00	2,00	#NULL!	#NULL!
45,00	2,00	2,00	2,00	2,00	2,00	2,00	#NULL!	#NULL!
46,00	1,00	2,00	2,00	2,00	2,00	2,00	#NULL!	#NULL!
45,00	2,00	2,00	2,00	2,00	2,00	2,00	#NULL!	#NULL!
47,00	1,00	2,00	2,00	2,00	2,00	2,00	#NULL!	#NULL!
49,00	2,00	2,00	2,00	2,00	2,00	2,00	#NULL!	#NULL!
50,00	2,00	2,00	2,00	2,00	2,00	2,00	#NULL!	#NULL!
55,00	1,00	2,00	2,00	2,00	1,00	2,00	#NULL!	#NULL!
70,00	2,00	2,00	2,00	2,00	2,00	2,00	#NULL!	#NULL!
78,00	1,00	2,00	2,00	2,00	2,00	2,00	#NULL!	#NULL!
70,00	1,00	2,00	2,00	2,00	2,00	2,00	#NULL!	#NULL!
65,00	2,00	2,00	2,00	2,00	2,00	2,00	#NULL!	#NULL!
65,00	1,00	2,00	2,00	2,00	2,00	2,00	#NULL!	#NULL!
60,00	2,00	2,00	2,00	2,00	2,00	2,00	#NULL!	#NULL!
65,00	2,00	1,00	2,00	2,00	1,00	2,00	#NULL!	#NULL!
70,00	1,00	2,00	2,00	2,00	2,00	1,00	40,00	#NULL!
60,00	2,00	2,00	2,00	2,00	2,00	2,00	#NULL!	#NULL!
55,00	2,00	2,00	2,00	2,00	2,00	2,00	#NULL!	#NULL!
59,00	2,00	1,00	1,00	2,00	2,00	2,00	#NULL!	#NULL!
55,00	2,00	1,00	1,00	2,00	2,00	2,00	#NULL!	#NULL!
47,00	2,00	2,00	2,00	2,00	2,00	2,00	#NULL!	#NULL!
45,00	1,00	2,00	2,00	2,00	2,00	2,00	#NULL!	#NULL!
60,00	2,00	2,00	2,00	2,00	2,00	2,00	#NULL!	#NULL!
65,00	1,00	2,00	2,00	2,00	2,00	2,00	#NULL!	#NULL!
70,00	1,00	2,00	2,00	2,00	2,00	2,00	#NULL!	#NULL!
65,00	2,00	2,00	2,00	2,00	2,00	2,00	#NULL!	#NULL!
54,00	1,00	2,00	2,00	2,00	2,00	2,00	#NULL!	#NULL!
51,00	1,00	2,00	2,00	2,00	2,00	2,00	#NULL!	#NULL!
64,00	2,00	1,00	2,00	2,00	2,00	2,00	#NULL!	#NULL!

64,00	1,00	1,00	2,00	2,00	2,00	2,00	30,00	11,00
49,00	1,00	1,00	2,00	1,00	2,00	2,00	#NULL!	#NULL!
45,00	2,00	2,00	2,00	2,00	2,00	2,00	#NULL!	#NULL!
48,00	2,00	2,00	1,00	2,00	2,00	1,00	10,00	#NULL!
52,00	2,00	2,00	2,00	2,00	2,00	2,00	#NULL!	#NULL!
45,00	1,00	2,00	2,00	2,00	2,00	2,00	#NULL!	#NULL!
47,00	2,00	2,00	2,00	2,00	2,00	2,00	#NULL!	#NULL!
50,00	2,00	1,00	2,00	2,00	2,00	2,00	#NULL!	#NULL!
45,00	1,00	1,00	2,00	2,00	2,00	2,00	#NULL!	#NULL!
45,00	2,00	2,00	2,00	2,00	2,00	2,00	#NULL!	#NULL!
48,00	1,00	1,00	2,00	2,00	2,00	2,00	#NULL!	#NULL!
58,00	1,00	1,00	1,00	2,00	2,00	2,00	#NULL!	#NULL!
45,00	1,00	2,00	2,00	2,00	2,00	2,00	#NULL!	#NULL!
64,00	1,00	2,00	2,00	2,00	2,00	2,00	#NULL!	#NULL!
45,00	1,00	2,00	2,00	2,00	2,00	2,00	#NULL!	#NULL!
45,00	2,00	2,00	2,00	2,00	2,00	2,00	#NULL!	#NULL!
46,00	1,00	2,00	2,00	2,00	2,00	2,00	#NULL!	#NULL!
50,00	2,00	2,00	2,00	2,00	2,00	2,00	#NULL!	#NULL!
56,00	1,00	2,00	2,00	2,00	2,00	2,00	#NULL!	#NULL!
78,00	1,00	1,00	1,00	1,00	2,00	2,00	#NULL!	#NULL!
70,00	2,00	2,00	2,00	2,00	2,00	2,00	#NULL!	#NULL!
64,00	2,00	2,00	2,00	2,00	2,00	2,00	#NULL!	#NULL!
45,00	2,00	1,00	1,00	2,00	2,00	2,00	#NULL!	#NULL!
66,00	2,00	1,00	1,00	1,00	1,00	1,00	20,00	#NULL!
63,00	2,00	2,00	2,00	2,00	2,00	2,00	#NULL!	#NULL!
45,00	2,00	2,00	2,00	2,00	2,00	2,00	#NULL!	#NULL!
49,00	1,00	2,00	2,00	2,00	2,00	2,00	#NULL!	#NULL!
82,00	1,00	1,00	2,00	2,00	2,00	2,00	150,00	10,00
79,00	2,00	1,00	2,00	1,00	2,00	2,00	#NULL!	#NULL!
72,00	2,00	1,00	2,00	1,00	2,00	1,00	5,00	#NULL!
50,00	1,00	2,00	2,00	2,00	2,00	2,00	#NULL!	#NULL!
70,00	2,00	1,00	2,00	1,00	1,00	2,00	#NULL!	#NULL!
80,00	1,00	1,00	1,00	2,00	2,00	2,00	#NULL!	#NULL!
45,00	1,00	2,00	2,00	2,00	2,00	2,00	#NULL!	#NULL!
48,00	2,00	2,00	2,00	2,00	2,00	2,00	#NULL!	#NULL!
70,00	1,00	2,00	2,00	1,00	2,00	2,00	8,00	10,00
67,00	2,00	1,00	2,00	1,00	2,00	2,00	#NULL!	#NULL!
45,00	1,00	2,00	2,00	2,00	2,00	2,00	#NULL!	#NULL!
48,00	2,00	2,00	2,00	2,00	2,00	2,00	#NULL!	#NULL!
45,00	1,00	2,00	2,00	2,00	2,00	2,00	#NULL!	#NULL!
45,00	2,00	2,00	2,00	1,00	2,00	2,00	#NULL!	#NULL!
77,00	1,00	2,00	2,00	2,00	2,00	2,00	#NULL!	#NULL!
76,00	2,00	1,00	2,00	2,00	2,00	2,00	#NULL!	#NULL!
86,00	1,00	2,00	2,00	2,00	2,00	2,00	#NULL!	#NULL!
90,00	1,00	2,00	2,00	2,00	2,00	2,00	#NULL!	#NULL!
83,00	1,00	1,00	1,00	2,00	2,00	2,00	#NULL!	#NULL!
49,00	1,00	2,00	2,00	2,00	2,00	2,00	#NULL!	#NULL!
65,00	2,00	1,00	1,00	2,00	1,00	2,00	#NULL!	#NULL!
45,00	2,00	2,00	2,00	2,00	2,00	2,00	#NULL!	#NULL!
59,00	1,00	2,00	2,00	2,00	2,00	2,00	#NULL!	#NULL!

56,00	1,00	2,00	2,00	2,00	2,00	2,00	#NULL!	#NULL!
47,00	2,00	2,00	2,00	2,00	2,00	2,00	#NULL!	#NULL!
74,00	2,00	1,00	2,00	2,00	2,00	2,00	#NULL!	#NULL!
61,00	1,00	1,00	2,00	1,00	1,00	1,00	40,00	#NULL!
78,00	1,00	1,00	1,00	2,00	2,00	1,00	15,00	#NULL!
57,00	1,00	2,00	2,00	2,00	2,00	2,00	#NULL!	#NULL!
73,00	2,00	1,00	2,00	2,00	2,00	2,00	#NULL!	#NULL!
70,00	2,00	1,00	1,00	2,00	2,00	2,00	#NULL!	#NULL!
55,00	1,00	2,00	2,00	1,00	2,00	1,00	30,00	#NULL!
64,00	2,00	1,00	1,00	2,00	2,00	2,00	#NULL!	#NULL!
49,00	1,00	2,00	2,00	2,00	2,00	2,00	#NULL!	#NULL!
46,00	2,00	2,00	2,00	2,00	2,00	1,00	20,00	#NULL!
61,00	2,00	1,00	2,00	2,00	2,00	1,00	15,00	#NULL!
66,00	1,00	2,00	1,00	2,00	1,00	2,00	#NULL!	#NULL!
73,00	2,00	1,00	2,00	1,00	2,00	2,00	#NULL!	#NULL!
56,00	1,00	2,00	2,00	2,00	1,00	2,00	#NULL!	#NULL!
57,00	2,00	1,00	2,00	2,00	2,00	2,00	#NULL!	#NULL!
62,00	1,00	2,00	1,00	1,00	1,00	2,00	#NULL!	#NULL!
52,00	2,00	2,00	2,00	2,00	2,00	2,00	#NULL!	#NULL!
48,00	1,00	2,00	2,00	2,00	2,00	2,00	#NULL!	#NULL!
56,00	2,00	1,00	2,00	2,00	2,00	2,00	#NULL!	#NULL!
47,00	2,00	2,00	2,00	2,00	2,00	2,00	#NULL!	#NULL!
48,00	2,00	1,00	2,00	2,00	2,00	2,00	#NULL!	#NULL!
46,00	2,00	2,00	2,00	2,00	2,00	2,00	#NULL!	#NULL!
57,00	2,00	1,00	2,00	2,00	1,00	2,00	#NULL!	#NULL!
75,00	1,00	1,00	2,00	1,00	2,00	1,00	20,00	#NULL!
50,00	1,00	2,00	2,00	2,00	2,00	2,00	#NULL!	#NULL!
63,00	1,00	2,00	1,00	2,00	1,00	2,00	#NULL!	#NULL!
85,00	2,00	1,00	2,00	2,00	2,00	2,00	#NULL!	#NULL!
76,00	2,00	1,00	2,00	1,00	2,00	2,00	#NULL!	#NULL!
76,00	2,00	1,00	1,00	2,00	2,00	2,00	#NULL!	#NULL!
48,00	1,00	2,00	2,00	2,00	2,00	2,00	#NULL!	#NULL!
77,00	1,00	1,00	2,00	1,00	2,00	1,00	20,00	#NULL!
45,00	1,00	2,00	2,00	2,00	2,00	2,00	#NULL!	#NULL!
45,00	2,00	2,00	2,00	2,00	2,00	2,00	#NULL!	#NULL!
64,00	2,00	1,00	2,00	2,00	1,00	2,00	#NULL!	#NULL!
68,00	2,00	1,00	1,00	2,00	2,00	2,00	#NULL!	#NULL!
60,00	1,00	1,00	1,00	2,00	1,00	2,00	#NULL!	#NULL!
45,00	2,00	2,00	2,00	2,00	1,00	2,00	#NULL!	#NULL!
62,00	2,00	2,00	1,00	2,00	2,00	2,00	#NULL!	#NULL!
45,00	2,00	2,00	2,00	2,00	2,00	2,00	#NULL!	#NULL!
61,00	2,00	1,00	2,00	2,00	2,00	2,00	#NULL!	#NULL!
45,00	1,00	2,00	2,00	2,00	2,00	2,00	#NULL!	#NULL!
45,00	2,00	2,00	2,00	2,00	2,00	2,00	#NULL!	#NULL!
49,00	1,00	2,00	2,00	2,00	2,00	1,00	20,00	#NULL!
56,00	1,00	2,00	1,00	2,00	2,00	2,00	#NULL!	#NULL!
70,00	2,00	1,00	2,00	1,00	2,00	2,00	#NULL!	#NULL!
75,00	2,00	2,00	1,00	1,00	1,00	2,00	#NULL!	#NULL!
52,00	2,00	1,00	2,00	2,00	2,00	2,00	#NULL!	#NULL!
48,00	2,00	2,00	2,00	2,00	2,00	2,00	#NULL!	#NULL!

56,00	1,00	2,00	1,00	2,00	2,00	2,00	#NULL!	#NULL!
48,00	1,00	2,00	2,00	2,00	2,00	2,00	#NULL!	#NULL!
89,00	1,00	1,00	2,00	1,00	2,00	2,00	#NULL!	#NULL!
64,00	2,00	2,00	1,00	2,00	1,00	2,00	#NULL!	#NULL!
72,00	2,00	1,00	1,00	2,00	2,00	2,00	#NULL!	#NULL!
63,00	1,00	1,00	2,00	2,00	2,00	2,00	#NULL!	#NULL!
45,00	1,00	2,00	2,00	2,00	2,00	2,00	#NULL!	#NULL!
74,00	2,00	1,00	2,00	1,00	2,00	2,00	#NULL!	#NULL!
55,00	2,00	1,00	2,00	2,00	2,00	2,00	#NULL!	#NULL!
64,00	2,00	1,00	2,00	2,00	2,00	2,00	#NULL!	#NULL!
60,00	1,00	2,00	1,00	1,00	2,00	1,00	15,00	#NULL!
71,00	2,00	1,00	2,00	2,00	2,00	2,00	#NULL!	#NULL!
68,00	2,00	1,00	1,00	1,00	2,00	2,00	#NULL!	#NULL!
54,00	2,00	1,00	2,00	2,00	2,00	2,00	#NULL!	#NULL!
47,00	1,00	2,00	2,00	2,00	2,00	1,00	7,00	#NULL!
45,00	1,00	2,00	2,00	2,00	2,00	2,00	#NULL!	#NULL!
53,00	1,00	1,00	2,00	2,00	2,00	2,00	#NULL!	#NULL!
92,00	1,00	1,00	1,00	1,00	2,00	2,00	#NULL!	#NULL!
46,00	2,00	2,00	1,00	2,00	2,00	2,00	#NULL!	#NULL!
55,00	1,00	2,00	2,00	2,00	2,00	2,00	#NULL!	#NULL!
71,00	2,00	1,00	2,00	2,00	2,00	2,00	#NULL!	#NULL!
49,00	2,00	2,00	2,00	2,00	2,00	2,00	#NULL!	#NULL!
79,00	1,00	1,00	2,00	1,00	2,00	2,00	#NULL!	#NULL!
50,00	1,00	2,00	2,00	2,00	2,00	2,00	#NULL!	#NULL!
55,00	1,00	1,00	2,00	2,00	2,00	2,00	#NULL!	#NULL!
64,00	1,00	1,00	2,00	1,00	2,00	2,00	#NULL!	#NULL!
45,00	2,00	2,00	2,00	2,00	2,00	2,00	#NULL!	#NULL!
51,00	1,00	2,00	2,00	2,00	2,00	2,00	#NULL!	#NULL!
52,00	2,00	2,00	2,00	2,00	2,00	2,00	#NULL!	#NULL!
63,00	1,00	1,00	1,00	1,00	2,00	1,00	33,00	#NULL!
52,00	2,00	2,00	2,00	2,00	2,00	2,00	#NULL!	#NULL!
62,00	2,00	2,00	1,00	1,00	1,00	2,00	#NULL!	#NULL!
59,00	1,00	1,00	2,00	1,00	2,00	2,00	#NULL!	#NULL!
69,00	2,00	1,00	1,00	2,00	1,00	2,00	#NULL!	#NULL!
64,00	1,00	1,00	2,00	2,00	1,00	2,00	#NULL!	#NULL!
59,00	2,00	1,00	2,00	2,00	2,00	2,00	#NULL!	#NULL!
60,00	2,00	1,00	2,00	2,00	1,00	2,00	#NULL!	#NULL!
45,00	2,00	2,00	2,00	2,00	2,00	2,00	#NULL!	#NULL!
58,00	1,00	2,00	2,00	1,00	2,00	2,00	#NULL!	#NULL!
47,00	2,00	2,00	2,00	2,00	2,00	2,00	#NULL!	#NULL!
55,00	1,00	1,00	2,00	2,00	2,00	2,00	#NULL!	#NULL!
58,00	2,00	2,00	1,00	2,00	2,00	2,00	#NULL!	#NULL!
66,00	2,00	1,00	1,00	2,00	1,00	2,00	#NULL!	#NULL!
62,00	1,00	2,00	2,00	2,00	2,00	2,00	#NULL!	#NULL!
59,00	2,00	1,00	2,00	2,00	2,00	2,00	#NULL!	#NULL!
45,00	1,00	2,00	2,00	2,00	2,00	2,00	#NULL!	#NULL!
60,00	2,00	1,00	2,00	2,00	2,00	2,00	#NULL!	#NULL!
64,00	2,00	1,00	2,00	1,00	2,00	2,00	#NULL!	#NULL!
52,00	2,00	1,00	2,00	1,00	1,00	2,00	#NULL!	#NULL!
55,00	2,00	2,00	1,00	2,00	2,00	2,00	#NULL!	#NULL!

61,00	1,00	1,00	2,00	2,00	2,00	2,00	#NULL!	#NULL!
45,00	2,00	2,00	2,00	2,00	2,00	2,00	#NULL!	#NULL!
46,00	1,00	1,00	2,00	2,00	2,00	1,00	10,00	#NULL!
69,00	2,00	1,00	2,00	2,00	2,00	2,00	#NULL!	#NULL!
67,00	2,00	2,00	1,00	2,00	1,00	2,00	#NULL!	#NULL!
82,00	1,00	1,00	2,00	1,00	2,00	1,00	50,00	#NULL!
47,00	2,00	2,00	2,00	2,00	2,00	2,00	#NULL!	#NULL!
49,00	1,00	2,00	2,00	2,00	2,00	2,00	#NULL!	#NULL!
69,00	2,00	1,00	2,00	2,00	2,00	2,00	#NULL!	#NULL!
69,00	1,00	1,00	2,00	2,00	2,00	1,00	50,00	#NULL!
58,00	2,00	1,00	2,00	2,00	2,00	2,00	#NULL!	#NULL!
61,00	1,00	1,00	2,00	2,00	2,00	2,00	#NULL!	#NULL!
47,00	2,00	1,00	2,00	1,00	2,00	1,00	9,00	#NULL!
63,00	1,00	2,00	2,00	2,00	2,00	2,00	#NULL!	#NULL!
52,00	1,00	1,00	2,00	2,00	2,00	2,00	15,00	15,00
50,00	1,00	1,00	2,00	2,00	2,00	2,00	#NULL!	#NULL!
45,00	2,00	2,00	2,00	2,00	2,00	2,00	#NULL!	#NULL!
45,00	2,00	1,00	1,00	2,00	2,00	2,00	#NULL!	#NULL!
52,00	2,00	1,00	2,00	2,00	2,00	2,00	#NULL!	#NULL!
76,00	1,00	1,00	2,00	2,00	2,00	2,00	#NULL!	#NULL!
70,00	2,00	2,00	2,00	2,00	2,00	2,00	#NULL!	#NULL!
50,00	2,00	2,00	2,00	2,00	2,00	2,00	#NULL!	#NULL!
52,00	1,00	2,00	2,00	2,00	2,00	2,00	#NULL!	#NULL!
50,00	2,00	2,00	2,00	2,00	2,00	2,00	#NULL!	#NULL!
56,00	1,00	2,00	2,00	2,00	2,00	2,00	#NULL!	#NULL!
58,00	2,00	1,00	1,00	1,00	2,00	1,00	20,00	#NULL!
56,00	2,00	1,00	2,00	2,00	2,00	2,00	#NULL!	#NULL!
60,00	1,00	1,00	2,00	2,00	2,00	2,00	#NULL!	#NULL!
45,00	1,00	2,00	2,00	2,00	2,00	2,00	#NULL!	#NULL!
55,00	2,00	1,00	2,00	2,00	2,00	2,00	#NULL!	#NULL!
45,00	2,00	2,00	2,00	2,00	2,00	2,00	#NULL!	#NULL!
45,00	2,00	2,00	2,00	2,00	2,00	2,00	#NULL!	#NULL!
48,00	1,00	2,00	2,00	2,00	2,00	2,00	#NULL!	#NULL!
49,00	2,00	2,00	2,00	2,00	2,00	2,00	#NULL!	#NULL!
50,00	1,00	1,00	2,00	2,00	2,00	2,00	#NULL!	#NULL!
45,00	2,00	2,00	2,00	2,00	2,00	2,00	#NULL!	#NULL!
50,00	1,00	2,00	2,00	2,00	2,00	2,00	#NULL!	#NULL!
61,00	1,00	2,00	2,00	2,00	2,00	2,00	#NULL!	#NULL!
55,00	2,00	2,00	2,00	2,00	2,00	2,00	#NULL!	#NULL!
50,00	1,00	2,00	2,00	2,00	2,00	2,00	#NULL!	#NULL!
45,00	2,00	2,00	2,00	2,00	2,00	2,00	#NULL!	#NULL!
55,00	1,00	1,00	2,00	2,00	2,00	2,00	#NULL!	#NULL!
87,00	1,00	2,00	2,00	2,00	2,00	2,00	#NULL!	#NULL!
45,00	2,00	2,00	2,00	2,00	2,00	2,00	#NULL!	#NULL!
50,00	1,00	2,00	2,00	2,00	2,00	2,00	#NULL!	#NULL!
45,00	2,00	2,00	2,00	2,00	2,00	2,00	#NULL!	#NULL!
50,00	1,00	2,00	2,00	2,00	2,00	2,00	#NULL!	#NULL!
45,00	2,00	2,00	2,00	2,00	2,00	2,00	#NULL!	#NULL!
45,00	1,00	2,00	2,00	2,00	2,00	2,00	#NULL!	#NULL!
56,00	2,00	2,00	2,00	2,00	2,00	2,00	#NULL!	#NULL!

45,00	1,00	2,00	2,00	2,00	2,00	2,00	#NULL!	#NULL!
67,00	2,00	2,00	2,00	2,00	2,00	2,00	#NULL!	#NULL!
45,00	2,00	2,00	2,00	2,00	2,00	2,00	#NULL!	#NULL!
59,00	2,00	2,00	2,00	2,00	2,00	2,00	#NULL!	#NULL!
56,00	1,00	2,00	2,00	2,00	2,00	2,00	#NULL!	#NULL!
47,00	2,00	2,00	2,00	2,00	2,00	2,00	#NULL!	#NULL!
45,00	1,00	2,00	2,00	2,00	2,00	2,00	#NULL!	#NULL!
69,00	2,00	2,00	2,00	2,00	2,00	2,00	#NULL!	#NULL!
47,00	2,00	2,00	2,00	2,00	2,00	2,00	#NULL!	#NULL!
55,00	2,00	2,00	2,00	2,00	2,00	2,00	#NULL!	#NULL!
74,00	2,00	2,00	2,00	2,00	2,00	2,00	#NULL!	#NULL!
65,00	1,00	2,00	2,00	2,00	2,00	2,00	#NULL!	#NULL!
78,00	2,00	2,00	2,00	2,00	2,00	2,00	#NULL!	#NULL!
55,00	1,00	2,00	2,00	2,00	2,00	2,00	#NULL!	#NULL!
51,00	2,00	2,00	2,00	2,00	2,00	2,00	#NULL!	#NULL!
50,00	1,00	2,00	2,00	2,00	2,00	2,00	#NULL!	#NULL!
56,00	2,00	2,00	2,00	2,00	2,00	2,00	#NULL!	#NULL!
54,00	2,00	2,00	2,00	2,00	2,00	2,00	#NULL!	#NULL!
73,00	2,00	2,00	2,00	2,00	2,00	2,00	#NULL!	#NULL!
76,00	2,00	2,00	2,00	2,00	2,00	2,00	#NULL!	#NULL!
67,00	2,00	2,00	2,00	2,00	2,00	2,00	#NULL!	#NULL!
51,00	1,00	2,00	2,00	2,00	2,00	2,00	#NULL!	#NULL!
55,00	2,00	2,00	2,00	2,00	2,00	2,00	#NULL!	#NULL!
58,00	2,00	2,00	2,00	2,00	2,00	2,00	#NULL!	#NULL!
47,00	2,00	2,00	2,00	2,00	2,00	2,00	#NULL!	#NULL!
45,00	2,00	2,00	2,00	2,00	2,00	2,00	#NULL!	#NULL!
50,00	1,00	2,00	2,00	2,00	2,00	2,00	#NULL!	#NULL!
45,00	2,00	2,00	2,00	2,00	2,00	2,00	#NULL!	#NULL!
50,00	1,00	2,00	2,00	2,00	2,00	2,00	#NULL!	#NULL!
45,00	1,00	2,00	2,00	2,00	2,00	2,00	#NULL!	#NULL!
45,00	1,00	2,00	2,00	2,00	2,00	2,00	#NULL!	#NULL!
55,00	1,00	2,00	2,00	2,00	2,00	2,00	#NULL!	#NULL!
45,00	2,00	2,00	2,00	2,00	2,00	2,00	#NULL!	#NULL!
45,00	2,00	2,00	2,00	2,00	2,00	2,00	#NULL!	#NULL!
45,00	2,00	2,00	2,00	2,00	2,00	2,00	#NULL!	#NULL!
50,00	1,00	2,00	2,00	2,00	2,00	2,00	#NULL!	#NULL!
45,00	2,00	2,00	2,00	2,00	2,00	2,00	#NULL!	#NULL!
47,00	2,00	2,00	2,00	2,00	2,00	2,00	#NULL!	#NULL!
50,00	1,00	2,00	2,00	2,00	2,00	2,00	#NULL!	#NULL!
49,00	2,00	2,00	2,00	2,00	2,00	2,00	#NULL!	#NULL!
50,00	1,00	2,00	2,00	2,00	2,00	2,00	#NULL!	#NULL!
45,00	2,00	2,00	2,00	2,00	2,00	2,00	#NULL!	#NULL!
45,00	2,00	2,00	2,00	2,00	2,00	2,00	#NULL!	#NULL!
52,00	1,00	2,00	2,00	2,00	2,00	2,00	#NULL!	#NULL!
47,00	1,00	2,00	2,00	2,00	2,00	2,00	#NULL!	#NULL!
45,00	2,00	2,00	2,00	2,00	2,00	2,00	#NULL!	#NULL!
60,00	2,00	2,00	2,00	2,00	2,00	2,00	#NULL!	#NULL!
70,00	1,00	2,00	2,00	2,00	2,00	2,00	#NULL!	#NULL!
60,00	2,00	2,00	2,00	2,00	2,00	2,00	#NULL!	#NULL!
50,00	2,00	2,00	2,00	2,00	2,00	2,00	#NULL!	#NULL!

53,00	2,00	2,00	2,00	2,00	2,00	2,00	#NULL!	#NULL!
50,00	1,00	2,00	2,00	2,00	2,00	2,00	#NULL!	#NULL!
45,00	2,00	2,00	2,00	2,00	2,00	2,00	#NULL!	#NULL!
53,00	2,00	2,00	2,00	2,00	2,00	2,00	#NULL!	#NULL!
55,00	1,00	2,00	2,00	2,00	2,00	2,00	#NULL!	#NULL!
46,00	2,00	2,00	2,00	2,00	2,00	2,00	#NULL!	#NULL!
45,00	2,00	2,00	2,00	2,00	2,00	2,00	#NULL!	#NULL!
50,00	1,00	2,00	2,00	2,00	2,00	2,00	#NULL!	#NULL!
52,00	2,00	2,00	2,00	2,00	2,00	2,00	#NULL!	#NULL!
45,00	2,00	2,00	2,00	2,00	2,00	2,00	#NULL!	#NULL!
48,00	1,00	2,00	2,00	2,00	2,00	2,00	#NULL!	#NULL!
50,00	1,00	2,00	2,00	2,00	2,00	2,00	#NULL!	#NULL!
48,00	2,00	2,00	2,00	2,00	2,00	2,00	#NULL!	#NULL!
52,00	2,00	2,00	2,00	2,00	2,00	2,00	#NULL!	#NULL!
72,00	2,00	1,00	2,00	2,00	2,00	2,00	#NULL!	#NULL!
50,00	2,00	2,00	2,00	2,00	2,00	2,00	#NULL!	#NULL!
55,00	1,00	2,00	2,00	2,00	2,00	2,00	#NULL!	#NULL!
60,00	1,00	2,00	2,00	2,00	2,00	2,00	#NULL!	#NULL!
45,00	1,00	2,00	2,00	2,00	2,00	2,00	#NULL!	#NULL!
45,00	1,00	2,00	2,00	2,00	2,00	2,00	#NULL!	#NULL!
45,00	2,00	2,00	2,00	2,00	2,00	2,00	#NULL!	#NULL!
45,00	2,00	2,00	2,00	2,00	2,00	2,00	#NULL!	#NULL!
45,00	1,00	2,00	2,00	2,00	2,00	2,00	#NULL!	#NULL!
51,00	1,00	1,00	2,00	2,00	2,00	1,00	20,00	#NULL!
59,00	1,00	2,00	2,00	2,00	2,00	1,00	15,00	#NULL!
46,00	1,00	2,00	2,00	2,00	2,00	2,00	#NULL!	#NULL!
50,00	2,00	2,00	2,00	2,00	2,00	2,00	#NULL!	#NULL!
56,00	1,00	1,00	2,00	2,00	2,00	2,00	#NULL!	#NULL!
59,00	1,00	2,00	2,00	2,00	2,00	2,00	#NULL!	#NULL!
51,00	2,00	1,00	2,00	2,00	2,00	2,00	#NULL!	#NULL!
53,00	1,00	2,00	2,00	2,00	2,00	2,00	#NULL!	#NULL!
49,00	1,00	2,00	2,00	2,00	2,00	2,00	#NULL!	#NULL!
46,00	1,00	1,00	2,00	2,00	2,00	2,00	#NULL!	#NULL!
50,00	1,00	2,00	2,00	2,00	2,00	2,00	#NULL!	#NULL!
52,00	2,00	2,00	2,00	2,00	2,00	2,00	#NULL!	#NULL!
52,00	1,00	2,00	2,00	2,00	2,00	1,00	30,00	#NULL!
50,00	1,00	2,00	2,00	2,00	2,00	2,00	#NULL!	#NULL!
51,00	1,00	1,00	2,00	2,00	2,00	2,00	#NULL!	#NULL!
53,00	1,00	2,00	2,00	2,00	2,00	2,00	#NULL!	#NULL!
60,00	1,00	1,00	2,00	2,00	2,00	2,00	#NULL!	#NULL!
55,00	2,00	2,00	2,00	2,00	2,00	2,00	#NULL!	#NULL!
50,00	1,00	1,00	2,00	2,00	2,00	2,00	#NULL!	#NULL!
55,00	1,00	1,00	2,00	2,00	2,00	2,00	#NULL!	#NULL!
45,00	2,00	2,00	2,00	2,00	2,00	2,00	#NULL!	#NULL!
48,00	2,00	1,00	2,00	2,00	2,00	2,00	#NULL!	#NULL!
49,00	2,00	2,00	2,00	2,00	2,00	2,00	#NULL!	#NULL!
52,00	2,00	2,00	1,00	2,00	1,00	2,00	#NULL!	#NULL!
62,00	1,00	1,00	2,00	2,00	2,00	1,00	42,00	#NULL!
68,00	2,00	1,00	2,00	2,00	2,00	2,00	#NULL!	#NULL!
53,00	1,00	2,00	2,00	2,00	2,00	2,00	#NULL!	#NULL!

55,00	2,00	1,00	2,00	2,00	2,00	2,00	#NULL!	#NULL!
45,00	2,00	2,00	2,00	2,00	2,00	2,00	#NULL!	#NULL!
55,00	1,00	1,00	2,00	2,00	2,00	2,00	#NULL!	#NULL!
58,00	1,00	1,00	1,00	2,00	1,00	1,00	36,00	#NULL!
50,00	2,00	2,00	2,00	2,00	1,00	2,00	#NULL!	#NULL!
46,00	2,00	2,00	2,00	2,00	2,00	2,00	#NULL!	#NULL!
56,00	1,00	2,00	2,00	2,00	2,00	2,00	#NULL!	#NULL!
60,00	1,00	2,00	2,00	2,00	2,00	1,00	30,00	#NULL!
55,00	2,00	1,00	2,00	2,00	2,00	2,00	#NULL!	#NULL!
49,00	2,00	2,00	2,00	1,00	2,00	1,00	45,00	#NULL!
61,00	2,00	1,00	2,00	2,00	2,00	2,00	#NULL!	#NULL!
45,00	1,00	1,00	2,00	1,00	2,00	2,00	#NULL!	#NULL!
46,00	1,00	2,00	2,00	2,00	2,00	2,00	#NULL!	#NULL!
46,00	2,00	2,00	2,00	2,00	2,00	2,00	#NULL!	#NULL!
48,00	1,00	2,00	2,00	2,00	2,00	2,00	#NULL!	#NULL!
59,00	1,00	2,00	2,00	2,00	2,00	2,00	#NULL!	#NULL!
51,00	2,00	2,00	2,00	2,00	2,00	2,00	#NULL!	#NULL!
47,00	2,00	2,00	2,00	2,00	2,00	2,00	#NULL!	#NULL!
55,00	1,00	1,00	2,00	2,00	1,00	2,00	#NULL!	#NULL!
72,00	2,00	1,00	1,00	1,00	1,00	2,00	#NULL!	#NULL!
82,00	1,00	1,00	1,00	1,00	1,00	1,00	30,00	#NULL!
49,00	1,00	1,00	1,00	2,00	1,00	1,00	20,00	#NULL!
58,00	1,00	2,00	1,00	2,00	2,00	1,00	28,00	#NULL!
61,00	1,00	1,00	2,00	2,00	1,00	2,00	#NULL!	#NULL!
69,00	1,00	1,00	2,00	2,00	1,00	1,00	15,00	#NULL!
75,00	1,00	1,00	2,00	2,00	1,00	2,00	30,00	30,00
76,00	2,00	1,00	1,00	2,00	2,00	1,00	15,00	#NULL!
56,00	2,00	1,00	2,00	2,00	2,00	2,00	#NULL!	#NULL!
59,00	1,00	1,00	2,00	2,00	2,00	2,00	#NULL!	#NULL!
58,00	1,00	1,00	2,00	2,00	2,00	2,00	#NULL!	#NULL!
61,00	2,00	1,00	1,00	2,00	2,00	2,00	#NULL!	#NULL!
55,00	1,00	1,00	2,00	2,00	2,00	2,00	#NULL!	#NULL!
48,00	1,00	1,00	1,00	2,00	2,00	2,00	#NULL!	#NULL!
77,00	1,00	1,00	1,00	2,00	1,00	2,00	#NULL!	#NULL!
57,00	1,00	1,00	2,00	2,00	2,00	2,00	#NULL!	#NULL!
54,00	2,00	2,00	1,00	2,00	2,00	2,00	#NULL!	#NULL!
56,00	2,00	2,00	2,00	2,00	2,00	2,00	#NULL!	#NULL!
52,00	2,00	1,00	1,00	2,00	2,00	2,00	#NULL!	#NULL!
54,00	1,00	1,00	1,00	2,00	2,00	2,00	#NULL!	#NULL!
55,00	1,00	2,00	2,00	2,00	2,00	2,00	#NULL!	#NULL!
57,00	1,00	1,00	1,00	2,00	2,00	1,00	20,00	#NULL!
50,00	1,00	1,00	1,00	2,00	2,00	1,00	12,00	#NULL!
52,00	2,00	1,00	1,00	2,00	2,00	2,00	#NULL!	#NULL!
54,00	2,00	2,00	2,00	2,00	2,00	2,00	#NULL!	#NULL!
54,00	1,00	2,00	2,00	2,00	2,00	2,00	#NULL!	#NULL!
46,00	1,00	2,00	2,00	2,00	2,00	2,00	#NULL!	#NULL!
48,00	1,00	2,00	2,00	2,00	2,00	2,00	#NULL!	#NULL!
49,00	1,00	2,00	2,00	2,00	2,00	2,00	#NULL!	#NULL!
56,00	1,00	1,00	2,00	2,00	2,00	2,00	#NULL!	#NULL!
65,00	1,00	2,00	2,00	2,00	2,00	2,00	#NULL!	#NULL!

69,00	2,00	2,00	2,00	2,00	2,00	2,00	#NULL!	#NULL!
49,00	1,00	2,00	2,00	2,00	2,00	2,00	#NULL!	#NULL!
52,00	2,00	2,00	2,00	2,00	2,00	2,00	#NULL!	#NULL!
52,00	1,00	1,00	1,00	2,00	2,00	1,00	10,00	#NULL!
52,00	2,00	1,00	2,00	2,00	2,00	2,00	#NULL!	#NULL!
54,00	2,00	2,00	2,00	2,00	2,00	2,00	#NULL!	#NULL!
55,00	1,00	2,00	2,00	2,00	2,00	2,00	#NULL!	#NULL!
53,00	2,00	2,00	2,00	2,00	2,00	2,00	#NULL!	#NULL!
55,00	1,00	2,00	2,00	2,00	2,00	2,00	#NULL!	#NULL!
55,00	1,00	1,00	1,00	2,00	2,00	1,00	20,00	#NULL!
56,00	2,00	2,00	2,00	2,00	2,00	2,00	#NULL!	#NULL!
53,00	1,00	1,00	1,00	2,00	2,00	2,00	#NULL!	#NULL!
52,00	2,00	1,00	1,00	2,00	2,00	2,00	#NULL!	#NULL!
55,00	2,00	2,00	2,00	2,00	2,00	2,00	#NULL!	#NULL!
53,00	1,00	2,00	2,00	2,00	2,00	2,00	#NULL!	#NULL!
53,00	1,00	1,00	1,00	2,00	2,00	2,00	#NULL!	#NULL!
54,00	2,00	1,00	1,00	2,00	2,00	2,00	#NULL!	#NULL!
54,00	2,00	1,00	1,00	2,00	2,00	1,00	28,00	#NULL!
57,00	1,00	1,00	1,00	2,00	2,00	1,00	30,00	#NULL!
56,00	1,00	1,00	1,00	2,00	2,00	1,00	30,00	#NULL!
54,00	2,00	1,00	1,00	2,00	2,00	2,00	#NULL!	#NULL!
58,00	1,00	2,00	2,00	2,00	2,00	2,00	#NULL!	#NULL!
57,00	2,00	2,00	2,00	2,00	2,00	2,00	#NULL!	#NULL!
52,00	1,00	2,00	2,00	2,00	2,00	2,00	#NULL!	#NULL!
52,00	2,00	2,00	2,00	2,00	2,00	2,00	#NULL!	#NULL!
57,00	1,00	1,00	1,00	2,00	2,00	2,00	#NULL!	#NULL!
57,00	2,00	1,00	1,00	2,00	2,00	2,00	#NULL!	#NULL!
52,00	1,00	1,00	1,00	2,00	2,00	2,00	#NULL!	#NULL!
50,00	2,00	1,00	1,00	2,00	2,00	2,00	#NULL!	#NULL!
55,00	1,00	1,00	1,00	2,00	2,00	1,00	26,00	#NULL!
53,00	2,00	1,00	1,00	2,00	2,00	2,00	#NULL!	#NULL!
53,00	2,00	1,00	2,00	2,00	2,00	2,00	#NULL!	#NULL!
53,00	1,00	2,00	1,00	2,00	2,00	2,00	#NULL!	#NULL!
55,00	1,00	2,00	2,00	2,00	2,00	2,00	#NULL!	#NULL!
53,00	2,00	2,00	2,00	2,00	2,00	2,00	#NULL!	#NULL!
56,00	2,00	2,00	2,00	2,00	2,00	2,00	#NULL!	#NULL!
55,00	2,00	2,00	2,00	2,00	2,00	2,00	#NULL!	#NULL!
54,00	1,00	1,00	2,00	2,00	2,00	1,00	30,00	#NULL!
52,00	2,00	2,00	1,00	2,00	2,00	2,00	#NULL!	#NULL!
56,00	2,00	1,00	1,00	2,00	2,00	2,00	#NULL!	#NULL!
55,00	1,00	1,00	1,00	2,00	2,00	2,00	#NULL!	#NULL!
55,00	1,00	1,00	2,00	2,00	2,00	2,00	#NULL!	#NULL!
55,00	2,00	2,00	1,00	2,00	2,00	2,00	#NULL!	#NULL!
56,00	1,00	1,00	2,00	2,00	2,00	2,00	#NULL!	#NULL!
59,00	1,00	1,00	2,00	2,00	1,00	1,00	20,00	#NULL!
58,00	1,00	2,00	1,00	2,00	1,00	2,00	#NULL!	#NULL!
45,00	1,00	2,00	2,00	2,00	2,00	2,00	#NULL!	#NULL!
70,00	1,00	1,00	1,00	2,00	1,00	2,00	90,00	10,00
66,00	1,00	1,00	2,00	2,00	2,00	2,00	#NULL!	#NULL!
56,00	2,00	1,00	1,00	2,00	1,00	2,00	#NULL!	#NULL!

66,00	1,00	2,00	2,00	2,00	1,00	1,00	20,00	#NULL!
49,00	1,00	2,00	2,00	2,00	2,00	2,00	#NULL!	#NULL!
45,00	1,00	2,00	2,00	2,00	2,00	2,00	#NULL!	#NULL!
46,00	2,00	1,00	2,00	2,00	1,00	1,00	25,00	#NULL!
48,00	1,00	1,00	2,00	2,00	2,00	2,00	2,00	#NULL!
45,00	2,00	2,00	2,00	2,00	2,00	2,00	#NULL!	#NULL!
46,00	2,00	2,00	2,00	2,00	2,00	2,00	#NULL!	#NULL!
51,00	2,00	2,00	2,00	2,00	2,00	2,00	#NULL!	#NULL!
56,00	2,00	2,00	2,00	2,00	2,00	2,00	#NULL!	#NULL!
55,00	1,00	1,00	2,00	2,00	1,00	1,00	30,00	#NULL!
47,00	2,00	2,00	2,00	2,00	2,00	2,00	#NULL!	#NULL!
46,00	1,00	2,00	2,00	2,00	2,00	2,00	#NULL!	#NULL!
77,00	1,00	1,00	1,00	2,00	1,00	1,00	10,00	30,00
56,00	1,00	1,00	2,00	2,00	1,00	1,00	45,00	#NULL!
47,00	1,00	2,00	2,00	2,00	2,00	2,00	#NULL!	#NULL!
68,00	1,00	1,00	2,00	2,00	1,00	1,00	30,00	#NULL!
70,00	1,00	2,00	2,00	2,00	2,00	2,00	#NULL!	#NULL!
50,00	2,00	2,00	2,00	2,00	2,00	2,00	#NULL!	#NULL!
52,00	1,00	1,00	2,00	2,00	2,00	2,00	#NULL!	#NULL!
46,00	2,00	1,00	1,00	2,00	2,00	2,00	#NULL!	#NULL!
49,00	1,00	2,00	2,00	2,00	2,00	2,00	#NULL!	#NULL!
52,00	2,00	1,00	2,00	2,00	2,00	2,00	#NULL!	#NULL!
48,00	1,00	2,00	1,00	2,00	2,00	2,00	#NULL!	#NULL!
46,00	2,00	2,00	2,00	2,00	2,00	2,00	#NULL!	#NULL!
45,00	1,00	2,00	2,00	2,00	2,00	2,00	#NULL!	#NULL!
77,00	2,00	1,00	1,00	2,00	1,00	2,00	#NULL!	#NULL!
70,00	1,00	1,00	2,00	2,00	2,00	2,00	#NULL!	#NULL!
60,00	2,00	1,00	2,00	2,00	2,00	2,00	#NULL!	#NULL!
58,00	1,00	1,00	2,00	2,00	2,00	2,00	#NULL!	#NULL!
46,00	2,00	2,00	2,00	2,00	2,00	2,00	#NULL!	#NULL!
48,00	1,00	2,00	2,00	2,00	2,00	2,00	#NULL!	#NULL!
46,00	1,00	2,00	2,00	2,00	2,00	2,00	#NULL!	#NULL!
56,00	1,00	1,00	2,00	2,00	2,00	2,00	#NULL!	#NULL!
59,00	2,00	1,00	2,00	2,00	2,00	2,00	#NULL!	#NULL!
58,00	1,00	2,00	1,00	1,00	1,00	2,00	#NULL!	#NULL!
56,00	2,00	1,00	2,00	2,00	2,00	1,00	80,00	#NULL!
62,00	2,00	1,00	1,00	1,00	2,00	2,00	#NULL!	#NULL!
62,00	1,00	2,00	2,00	2,00	2,00	2,00	#NULL!	#NULL!
65,00	2,00	2,00	2,00	2,00	2,00	2,00	#NULL!	#NULL!
60,00	2,00	1,00	1,00	1,00	1,00	1,00	30,00	#NULL!
63,00	1,00	2,00	2,00	2,00	2,00	2,00	#NULL!	#NULL!
74,00	2,00	1,00	2,00	1,00	2,00	2,00	#NULL!	#NULL!
65,00	1,00	2,00	2,00	2,00	2,00	2,00	#NULL!	#NULL!
50,00	2,00	2,00	2,00	2,00	2,00	1,00	25,00	#NULL!
55,00	1,00	1,00	2,00	2,00	2,00	2,00	#NULL!	#NULL!
55,00	2,00	2,00	2,00	2,00	2,00	2,00	#NULL!	#NULL!
83,00	1,00	2,00	2,00	2,00	2,00	2,00	#NULL!	#NULL!
83,00	2,00	1,00	1,00	2,00	2,00	2,00	#NULL!	#NULL!
84,00	1,00	2,00	2,00	2,00	2,00	1,00	90,00	#NULL!
50,00	1,00	2,00	2,00	2,00	2,00	1,00	30,00	#NULL!

45,00	2,00	2,00	2,00	2,00	2,00	2,00	#NULL!	#NULL!
76,00	1,00	2,00	1,00	1,00	2,00	2,00	#NULL!	#NULL!
77,00	2,00	1,00	2,00	2,00	2,00	2,00	#NULL!	#NULL!
50,00	2,00	2,00	2,00	2,00	2,00	2,00	#NULL!	#NULL!
45,00	1,00	2,00	2,00	2,00	2,00	2,00	#NULL!	#NULL!
85,00	2,00	1,00	2,00	1,00	2,00	2,00	#NULL!	#NULL!
60,00	1,00	2,00	2,00	2,00	2,00	2,00	#NULL!	#NULL!
58,00	1,00	1,00	1,00	2,00	2,00	2,00	#NULL!	#NULL!
60,00	1,00	1,00	2,00	2,00	2,00	1,00	60,00	#NULL!
55,00	1,00	2,00	2,00	2,00	2,00	1,00	15,00	#NULL!
62,00	2,00	1,00	1,00	2,00	2,00	1,00	120,00	#NULL!
50,00	1,00	2,00	1,00	1,00	2,00	2,00	#NULL!	#NULL!
55,00	2,00	1,00	2,00	1,00	2,00	2,00	#NULL!	#NULL!
73,00	2,00	2,00	2,00	2,00	2,00	2,00	#NULL!	#NULL!
53,00	2,00	2,00	2,00	2,00	2,00	2,00	#NULL!	#NULL!
45,00	1,00	2,00	2,00	2,00	2,00	1,00	60,00	#NULL!
45,00	2,00	2,00	2,00	2,00	2,00	1,00	20,00	#NULL!
56,00	2,00	2,00	1,00	2,00	2,00	2,00	#NULL!	#NULL!
58,00	1,00	2,00	2,00	2,00	2,00	2,00	#NULL!	#NULL!
62,00	1,00	2,00	1,00	2,00	1,00	1,00	60,00	#NULL!
59,00	2,00	2,00	2,00	2,00	2,00	2,00	#NULL!	#NULL!
46,00	1,00	1,00	2,00	2,00	2,00	1,00	30,00	#NULL!
52,00	2,00	1,00	2,00	2,00	2,00	2,00	#NULL!	#NULL!
74,00	2,00	2,00	2,00	2,00	2,00	2,00	#NULL!	#NULL!
72,00	2,00	1,00	2,00	2,00	2,00	2,00	#NULL!	#NULL!
61,00	2,00	1,00	1,00	1,00	1,00	2,00	#NULL!	#NULL!
59,00	1,00	1,00	2,00	2,00	2,00	1,00	8,00	#NULL!
66,00	1,00	2,00	2,00	1,00	1,00	2,00	#NULL!	#NULL!
69,00	1,00	2,00	2,00	2,00	2,00	2,00	#NULL!	#NULL!
72,00	1,00	1,00	1,00	2,00	2,00	2,00	#NULL!	#NULL!
60,00	2,00	1,00	1,00	2,00	2,00	2,00	#NULL!	#NULL!
56,00	1,00	2,00	1,00	2,00	2,00	2,00	#NULL!	#NULL!
62,00	1,00	2,00	2,00	2,00	2,00	1,00	200,00	#NULL!
45,00	1,00	2,00	1,00	2,00	2,00	1,00	60,00	#NULL!
50,00	2,00	1,00	1,00	2,00	1,00	2,00	#NULL!	#NULL!
75,00	1,00	2,00	2,00	2,00	2,00	2,00	#NULL!	#NULL!
65,00	1,00	2,00	2,00	2,00	1,00	1,00	25,00	#NULL!
48,00	2,00	1,00	2,00	2,00	2,00	2,00	#NULL!	#NULL!
53,00	1,00	1,00	1,00	2,00	1,00	1,00	40,00	#NULL!
67,00	1,00	2,00	2,00	1,00	1,00	2,00	#NULL!	#NULL!
50,00	2,00	1,00	1,00	2,00	2,00	1,00	40,00	#NULL!
45,00	2,00	1,00	1,00	2,00	1,00	1,00	30,00	#NULL!
50,00	2,00	1,00	1,00	1,00	2,00	1,00	30,00	#NULL!
48,00	1,00	2,00	2,00	2,00	2,00	2,00	#NULL!	#NULL!
56,00	2,00	2,00	2,00	2,00	2,00	2,00	#NULL!	#NULL!
58,00	1,00	1,00	1,00	2,00	2,00	1,00	30,00	#NULL!
48,00	2,00	2,00	2,00	2,00	2,00	2,00	#NULL!	#NULL!
59,00	1,00	1,00	2,00	2,00	2,00	1,00	15,00	#NULL!
64,00	2,00	2,00	2,00	2,00	2,00	2,00	#NULL!	#NULL!
45,00	1,00	2,00	2,00	2,00	2,00	1,00	28,00	#NULL!

46,00	1,00	2,00	2,00	2,00	2,00	1,00	32,00	#NULL!
45,00	2,00	2,00	2,00	2,00	2,00	1,00	20,00	#NULL!
49,00	1,00	1,00	2,00	2,00	2,00	1,00	25,00	#NULL!
56,00	2,00	1,00	2,00	2,00	2,00	2,00	#NULL!	#NULL!
62,00	1,00	2,00	1,00	2,00	1,00	2,00	#NULL!	#NULL!
59,00	1,00	1,00	2,00	2,00	1,00	1,00	30,00	#NULL!
50,00	2,00	1,00	2,00	1,00	1,00	2,00	#NULL!	#NULL!
47,00	1,00	1,00	2,00	1,00	2,00	1,00	30,00	#NULL!
50,00	2,00	1,00	1,00	2,00	2,00	2,00	#NULL!	#NULL!
80,00	1,00	1,00	1,00	2,00	2,00	2,00	#NULL!	#NULL!
65,00	2,00	2,00	1,00	2,00	2,00	2,00	#NULL!	#NULL!
45,00	2,00	2,00	2,00	2,00	2,00	2,00	#NULL!	#NULL!
47,00	1,00	2,00	2,00	2,00	2,00	1,00	30,00	#NULL!
47,00	2,00	1,00	2,00	2,00	2,00	2,00	#NULL!	#NULL!
51,00	2,00	2,00	2,00	2,00	2,00	2,00	#NULL!	#NULL!
45,00	1,00	2,00	2,00	2,00	2,00	1,00	100,00	#NULL!
48,00	1,00	2,00	2,00	2,00	2,00	1,00	60,00	#NULL!
45,00	2,00	2,00	2,00	2,00	2,00	2,00	#NULL!	#NULL!
49,00	1,00	2,00	2,00	2,00	2,00	2,00	#NULL!	#NULL!
56,00	2,00	1,00	1,00	1,00	1,00	2,00	#NULL!	#NULL!
49,00	1,00	2,00	2,00	2,00	2,00	2,00	#NULL!	#NULL!
48,00	2,00	1,00	2,00	2,00	2,00	2,00	#NULL!	#NULL!
52,00	1,00	2,00	2,00	2,00	2,00	2,00	#NULL!	#NULL!
45,00	2,00	2,00	2,00	2,00	2,00	2,00	#NULL!	#NULL!
46,00	1,00	2,00	2,00	2,00	2,00	2,00	#NULL!	#NULL!
53,00	1,00	1,00	1,00	1,00	2,00	2,00	#NULL!	#NULL!
55,00	2,00	1,00	1,00	2,00	1,00	2,00	#NULL!	#NULL!
49,00	2,00	2,00	2,00	2,00	2,00	2,00	#NULL!	#NULL!
46,00	2,00	2,00	1,00	2,00	2,00	2,00	#NULL!	#NULL!
45,00	1,00	2,00	2,00	2,00	2,00	1,00	30,00	#NULL!
46,00	2,00	2,00	2,00	2,00	2,00	1,00	15,00	#NULL!
45,00	1,00	2,00	2,00	2,00	2,00	1,00	30,00	#NULL!
46,00	2,00	1,00	1,00	2,00	2,00	2,00	#NULL!	#NULL!
50,00	2,00	2,00	2,00	2,00	2,00	2,00	#NULL!	#NULL!
55,00	1,00	2,00	2,00	2,00	2,00	2,00	#NULL!	#NULL!
45,00	2,00	2,00	2,00	2,00	2,00	2,00	#NULL!	#NULL!
50,00	1,00	2,00	2,00	2,00	2,00	2,00	#NULL!	#NULL!
62,00	1,00	2,00	2,00	2,00	2,00	2,00	#NULL!	#NULL!
55,00	2,00	2,00	2,00	2,00	2,00	2,00	#NULL!	#NULL!
70,00	2,00	2,00	2,00	2,00	2,00	2,00	#NULL!	#NULL!
75,00	1,00	2,00	2,00	2,00	2,00	2,00	#NULL!	#NULL!
65,00	1,00	2,00	2,00	2,00	2,00	2,00	#NULL!	#NULL!
55,00	2,00	2,00	2,00	2,00	2,00	2,00	#NULL!	#NULL!
70,00	1,00	2,00	2,00	2,00	2,00	2,00	#NULL!	#NULL!
60,00	2,00	2,00	2,00	2,00	2,00	2,00	#NULL!	#NULL!
65,00	2,00	2,00	2,00	2,00	2,00	2,00	#NULL!	#NULL!
70,00	1,00	2,00	2,00	2,00	2,00	2,00	#NULL!	#NULL!
70,00	1,00	2,00	2,00	2,00	2,00	2,00	#NULL!	#NULL!
60,00	2,00	2,00	2,00	2,00	2,00	2,00	#NULL!	#NULL!
55,00	2,00	2,00	2,00	2,00	2,00	2,00	#NULL!	#NULL!

60,00	1,00	2,00	2,00	2,00	2,00	2,00	#NULL!	#NULL!
60,00	2,00	2,00	2,00	2,00	2,00	2,00	#NULL!	#NULL!
65,00	1,00	2,00	2,00	2,00	2,00	2,00	#NULL!	#NULL!
45,00	2,00	2,00	2,00	2,00	2,00	2,00	#NULL!	#NULL!
51,00	1,00	2,00	2,00	2,00	2,00	2,00	#NULL!	#NULL!
46,00	2,00	2,00	2,00	2,00	2,00	2,00	#NULL!	#NULL!
50,00	1,00	1,00	2,00	2,00	2,00	2,00	#NULL!	#NULL!
45,00	2,00	2,00	2,00	2,00	2,00	2,00	#NULL!	#NULL!
47,00	1,00	2,00	2,00	2,00	2,00	2,00	#NULL!	#NULL!
82,00	1,00	1,00	1,00	2,00	2,00	2,00	#NULL!	#NULL!
59,00	2,00	2,00	1,00	2,00	2,00	2,00	#NULL!	#NULL!
53,00	1,00	2,00	2,00	2,00	2,00	2,00	#NULL!	#NULL!
48,00	1,00	2,00	2,00	2,00	2,00	2,00	#NULL!	#NULL!
59,00	1,00	1,00	2,00	2,00	2,00	1,00	30,00	#NULL!
45,00	2,00	2,00	2,00	2,00	2,00	1,00	25,00	#NULL!
45,00	2,00	2,00	2,00	2,00	2,00	2,00	#NULL!	#NULL!
45,00	1,00	2,00	2,00	2,00	2,00	2,00	#NULL!	#NULL!
56,00	1,00	1,00	2,00	2,00	2,00	2,00	#NULL!	#NULL!
49,00	1,00	2,00	2,00	2,00	2,00	2,00	#NULL!	#NULL!
49,00	1,00	2,00	2,00	2,00	2,00	2,00	#NULL!	#NULL!
48,00	1,00	2,00	2,00	2,00	2,00	2,00	#NULL!	#NULL!
45,00	2,00	2,00	2,00	2,00	2,00	1,00	25,00	#NULL!
46,00	2,00	2,00	2,00	2,00	2,00	2,00	#NULL!	#NULL!
56,00	2,00	1,00	2,00	2,00	2,00	2,00	#NULL!	#NULL!
60,00	1,00	2,00	2,00	2,00	2,00	2,00	#NULL!	#NULL!
55,00	2,00	2,00	2,00	2,00	1,00	2,00	#NULL!	#NULL!
60,00	1,00	1,00	2,00	2,00	2,00	2,00	#NULL!	#NULL!
62,00	1,00	2,00	1,00	1,00	2,00	2,00	#NULL!	#NULL!
62,00	2,00	1,00	1,00	2,00	2,00	2,00	#NULL!	#NULL!
47,00	1,00	1,00	2,00	2,00	2,00	2,00	#NULL!	#NULL!
73,00	2,00	1,00	1,00	2,00	2,00	2,00	#NULL!	#NULL!
54,00	2,00	2,00	2,00	2,00	1,00	2,00	#NULL!	#NULL!
52,00	1,00	1,00	2,00	2,00	2,00	2,00	#NULL!	#NULL!
56,00	2,00	1,00	2,00	2,00	2,00	2,00	#NULL!	#NULL!
48,00	2,00	2,00	2,00	2,00	2,00	2,00	#NULL!	#NULL!
57,00	1,00	1,00	2,00	1,00	2,00	2,00	#NULL!	#NULL!
56,00	2,00	1,00	2,00	2,00	2,00	2,00	#NULL!	#NULL!
63,00	2,00	1,00	2,00	1,00	1,00	2,00	#NULL!	#NULL!
62,00	2,00	1,00	2,00	2,00	2,00	2,00	#NULL!	#NULL!
57,00	2,00	2,00	2,00	2,00	2,00	2,00	#NULL!	#NULL!
50,00	1,00	2,00	2,00	2,00	2,00	1,00	25,00	#NULL!
45,00	2,00	2,00	2,00	2,00	2,00	2,00	#NULL!	#NULL!
51,00	2,00	1,00	2,00	2,00	2,00	2,00	#NULL!	#NULL!
68,00	1,00	1,00	2,00	2,00	1,00	2,00	#NULL!	#NULL!
58,00	2,00	1,00	2,00	2,00	2,00	2,00	#NULL!	#NULL!
59,00	2,00	1,00	2,00	2,00	2,00	2,00	#NULL!	#NULL!
65,00	1,00	1,00	1,00	2,00	2,00	2,00	#NULL!	#NULL!
48,00	1,00	2,00	2,00	2,00	1,00	2,00	#NULL!	#NULL!
55,00	1,00	1,00	2,00	2,00	2,00	2,00	#NULL!	#NULL!
50,00	1,00	1,00	2,00	2,00	2,00	2,00	20,00	10,00

50,00	2,00	1,00	2,00	2,00	1,00	2,00	#NULL!	#NULL!
53,00	1,00	2,00	2,00	2,00	2,00	2,00	#NULL!	#NULL!
45,00	2,00	2,00	2,00	2,00	2,00	2,00	#NULL!	#NULL!
50,00	2,00	1,00	2,00	2,00	2,00	2,00	#NULL!	#NULL!
52,00	1,00	2,00	2,00	2,00	2,00	2,00	#NULL!	#NULL!
45,00	1,00	2,00	2,00	2,00	2,00	2,00	#NULL!	#NULL!
51,00	1,00	1,00	2,00	2,00	2,00	2,00	#NULL!	#NULL!
59,00	1,00	1,00	2,00	2,00	2,00	2,00	#NULL!	#NULL!
50,00	1,00	2,00	2,00	2,00	2,00	2,00	#NULL!	#NULL!
50,00	2,00	2,00	2,00	2,00	2,00	2,00	#NULL!	#NULL!
55,00	1,00	2,00	2,00	2,00	2,00	2,00	#NULL!	#NULL!
61,00	1,00	1,00	2,00	2,00	2,00	2,00	#NULL!	#NULL!
69,00	1,00	1,00	2,00	2,00	1,00	1,00	30,00	#NULL!
45,00	2,00	1,00	2,00	2,00	2,00	2,00	#NULL!	#NULL!
47,00	2,00	2,00	2,00	2,00	2,00	2,00	#NULL!	#NULL!
48,00	2,00	2,00	2,00	2,00	2,00	2,00	#NULL!	#NULL!
46,00	1,00	2,00	2,00	2,00	2,00	2,00	#NULL!	#NULL!
59,00	2,00	2,00	2,00	2,00	2,00	2,00	#NULL!	#NULL!
60,00	1,00	1,00	1,00	2,00	2,00	2,00	#NULL!	#NULL!
53,00	2,00	1,00	2,00	2,00	2,00	2,00	#NULL!	#NULL!
55,00	1,00	1,00	2,00	2,00	2,00	2,00	#NULL!	#NULL!
78,00	2,00	1,00	1,00	2,00	2,00	2,00	#NULL!	#NULL!
57,00	2,00	2,00	1,00	2,00	1,00	2,00	#NULL!	#NULL!
61,00	2,00	1,00	2,00	2,00	2,00	1,00	40,00	#NULL!
58,00	2,00	2,00	2,00	2,00	2,00	2,00	#NULL!	#NULL!
50,00	2,00	2,00	2,00	2,00	2,00	2,00	#NULL!	#NULL!
55,00	1,00	2,00	2,00	2,00	2,00	2,00	#NULL!	#NULL!
50,00	1,00	2,00	2,00	2,00	2,00	2,00	#NULL!	#NULL!
45,00	1,00	2,00	2,00	2,00	2,00	2,00	#NULL!	#NULL!
55,00	2,00	2,00	2,00	2,00	2,00	2,00	#NULL!	#NULL!
60,00	1,00	2,00	2,00	2,00	2,00	2,00	#NULL!	#NULL!
55,00	1,00	2,00	2,00	2,00	2,00	2,00	#NULL!	#NULL!
50,00	2,00	2,00	2,00	2,00	2,00	2,00	#NULL!	#NULL!
55,00	2,00	2,00	2,00	2,00	2,00	2,00	#NULL!	#NULL!
60,00	1,00	2,00	2,00	2,00	2,00	2,00	#NULL!	#NULL!
50,00	1,00	2,00	2,00	2,00	2,00	2,00	#NULL!	#NULL!
55,00	1,00	2,00	2,00	2,00	2,00	2,00	#NULL!	#NULL!
70,00	1,00	2,00	2,00	2,00	2,00	2,00	#NULL!	#NULL!
65,00	2,00	2,00	2,00	2,00	2,00	2,00	#NULL!	#NULL!
60,00	2,00	2,00	2,00	2,00	2,00	2,00	#NULL!	#NULL!
65,00	1,00	2,00	2,00	2,00	2,00	2,00	#NULL!	#NULL!
65,00	1,00	2,00	2,00	2,00	2,00	2,00	#NULL!	#NULL!
60,00	2,00	2,00	2,00	2,00	2,00	2,00	#NULL!	#NULL!
60,00	1,00	2,00	2,00	2,00	2,00	2,00	#NULL!	#NULL!
55,00	2,00	2,00	2,00	2,00	2,00	2,00	#NULL!	#NULL!
63,00	1,00	2,00	2,00	2,00	2,00	2,00	#NULL!	#NULL!
55,00	1,00	2,00	2,00	2,00	2,00	2,00	#NULL!	#NULL!
50,00	1,00	2,00	2,00	2,00	2,00	2,00	#NULL!	#NULL!
45,00	2,00	2,00	2,00	2,00	2,00	2,00	#NULL!	#NULL!
60,00	2,00	2,00	2,00	2,00	2,00	2,00	#NULL!	#NULL!

55,00	2,00	2,00	2,00	2,00	2,00	2,00	#NULL!	#NULL!
50,00	2,00	2,00	2,00	2,00	2,00	2,00	#NULL!	#NULL!
55,00	1,00	2,00	2,00	2,00	2,00	1,00	40,00	#NULL!
50,00	1,00	2,00	2,00	2,00	2,00	2,00	#NULL!	#NULL!
45,00	2,00	2,00	2,00	2,00	2,00	2,00	#NULL!	#NULL!
60,00	1,00	2,00	2,00	2,00	2,00	2,00	#NULL!	#NULL!
55,00	2,00	2,00	2,00	2,00	2,00	2,00	#NULL!	#NULL!
70,00	1,00	2,00	2,00	2,00	2,00	2,00	#NULL!	#NULL!
60,00	2,00	2,00	2,00	2,00	2,00	2,00	#NULL!	#NULL!
50,00	1,00	2,00	2,00	2,00	2,00	2,00	#NULL!	#NULL!
45,00	2,00	2,00	1,00	2,00	2,00	2,00	#NULL!	#NULL!
50,00	2,00	2,00	2,00	2,00	2,00	2,00	#NULL!	#NULL!
55,00	1,00	2,00	2,00	2,00	2,00	2,00	#NULL!	#NULL!
60,00	1,00	2,00	2,00	2,00	2,00	2,00	#NULL!	#NULL!
55,00	2,00	2,00	2,00	2,00	2,00	2,00	#NULL!	#NULL!
45,00	2,00	2,00	2,00	2,00	2,00	2,00	#NULL!	#NULL!
49,00	1,00	2,00	2,00	2,00	2,00	2,00	#NULL!	#NULL!
77,00	1,00	2,00	2,00	2,00	2,00	1,00	30,00	#NULL!
51,00	1,00	2,00	2,00	2,00	2,00	2,00	#NULL!	#NULL!
60,00	1,00	2,00	2,00	2,00	2,00	2,00	#NULL!	#NULL!
55,00	2,00	2,00	2,00	2,00	2,00	2,00	#NULL!	#NULL!
60,00	1,00	2,00	2,00	2,00	2,00	2,00	#NULL!	#NULL!
55,00	2,00	2,00	2,00	2,00	2,00	2,00	#NULL!	#NULL!
50,00	1,00	2,00	2,00	2,00	2,00	2,00	#NULL!	#NULL!
55,00	1,00	2,00	2,00	2,00	2,00	2,00	#NULL!	#NULL!
60,00	1,00	2,00	2,00	2,00	2,00	2,00	#NULL!	#NULL!
55,00	1,00	2,00	2,00	2,00	2,00	2,00	#NULL!	#NULL!
60,00	1,00	2,00	2,00	2,00	2,00	2,00	#NULL!	#NULL!
60,00	1,00	2,00	2,00	2,00	2,00	2,00	#NULL!	#NULL!
50,00	1,00	2,00	2,00	2,00	2,00	2,00	#NULL!	#NULL!
55,00	1,00	2,00	2,00	2,00	2,00	2,00	#NULL!	#NULL!
50,00	1,00	2,00	2,00	2,00	2,00	2,00	#NULL!	#NULL!
60,00	1,00	2,00	2,00	2,00	2,00	2,00	#NULL!	#NULL!
65,00	1,00	2,00	2,00	2,00	2,00	2,00	#NULL!	#NULL!
60,00	2,00	2,00	2,00	2,00	2,00	2,00	#NULL!	#NULL!
65,00	1,00	2,00	2,00	2,00	2,00	2,00	#NULL!	#NULL!
60,00	2,00	2,00	2,00	2,00	2,00	2,00	#NULL!	#NULL!
50,00	2,00	2,00	2,00	2,00	2,00	2,00	#NULL!	#NULL!
55,00	1,00	2,00	2,00	2,00	2,00	2,00	#NULL!	#NULL!
50,00	1,00	2,00	2,00	2,00	2,00	2,00	#NULL!	#NULL!
55,00	1,00	2,00	2,00	2,00	2,00	2,00	#NULL!	#NULL!
55,00	2,00	2,00	2,00	2,00	2,00	2,00	#NULL!	#NULL!
60,00	1,00	2,00	2,00	2,00	2,00	2,00	#NULL!	#NULL!
50,00	1,00	2,00	2,00	2,00	2,00	2,00	#NULL!	#NULL!
45,00	2,00	2,00	2,00	2,00	2,00	2,00	#NULL!	#NULL!
45,00	2,00	2,00	2,00	2,00	2,00	2,00	#NULL!	#NULL!
50,00	1,00	2,00	2,00	2,00	2,00	2,00	#NULL!	#NULL!
50,00	1,00	2,00	2,00	2,00	2,00	2,00	#NULL!	#NULL!
45,00	2,00	2,00	2,00	2,00	2,00	2,00	#NULL!	#NULL!
50,00	1,00	2,00	2,00	2,00	2,00	2,00	#NULL!	#NULL!

55,00	2,00	2,00	2,00	2,00	2,00	2,00	#NULL!	#NULL!
49,00	2,00	2,00	2,00	2,00	2,00	2,00	#NULL!	#NULL!
50,00	1,00	1,00	2,00	2,00	2,00	2,00	#NULL!	#NULL!
45,00	2,00	2,00	2,00	2,00	2,00	2,00	#NULL!	#NULL!
46,00	1,00	2,00	2,00	2,00	2,00	2,00	#NULL!	#NULL!
45,00	2,00	2,00	2,00	2,00	2,00	2,00	#NULL!	#NULL!
46,00	1,00	2,00	2,00	2,00	2,00	2,00	#NULL!	#NULL!
46,00	2,00	2,00	2,00	2,00	2,00	2,00	#NULL!	#NULL!
65,00	2,00	2,00	2,00	2,00	2,00	2,00	#NULL!	#NULL!
55,00	1,00	2,00	2,00	2,00	2,00	2,00	#NULL!	#NULL!
50,00	1,00	2,00	2,00	2,00	2,00	2,00	#NULL!	#NULL!
60,00	1,00	2,00	2,00	2,00	2,00	2,00	#NULL!	#NULL!
55,00	1,00	2,00	2,00	2,00	2,00	2,00	#NULL!	#NULL!
45,00	2,00	2,00	2,00	2,00	2,00	2,00	#NULL!	#NULL!
45,00	1,00	2,00	2,00	2,00	2,00	2,00	#NULL!	#NULL!
50,00	2,00	2,00	2,00	2,00	2,00	2,00	#NULL!	#NULL!
55,00	1,00	2,00	2,00	2,00	2,00	2,00	#NULL!	#NULL!
45,00	2,00	2,00	2,00	2,00	2,00	2,00	#NULL!	#NULL!
50,00	1,00	2,00	2,00	2,00	2,00	2,00	#NULL!	#NULL!
60,00	2,00	2,00	2,00	2,00	2,00	2,00	#NULL!	#NULL!
70,00	1,00	2,00	2,00	2,00	2,00	2,00	#NULL!	#NULL!
45,00	2,00	2,00	2,00	2,00	2,00	2,00	#NULL!	#NULL!
50,00	2,00	2,00	2,00	2,00	2,00	2,00	#NULL!	#NULL!
60,00	1,00	2,00	2,00	2,00	2,00	2,00	#NULL!	#NULL!
55,00	2,00	2,00	2,00	2,00	2,00	2,00	#NULL!	#NULL!
70,00	1,00	2,00	2,00	2,00	2,00	2,00	#NULL!	#NULL!
65,00	1,00	2,00	2,00	2,00	2,00	2,00	#NULL!	#NULL!
60,00	1,00	2,00	2,00	2,00	2,00	2,00	#NULL!	#NULL!
50,00	1,00	2,00	2,00	2,00	2,00	2,00	#NULL!	#NULL!
68,00	1,00	1,00	1,00	1,00	1,00	2,00	#NULL!	#NULL!
68,00	1,00	1,00	1,00	1,00	1,00	2,00	#NULL!	#NULL!
68,00	1,00	1,00	2,00	2,00	2,00	2,00	#NULL!	#NULL!
74,00	1,00	1,00	2,00	2,00	2,00	2,00	#NULL!	#NULL!
76,00	1,00	2,00	2,00	1,00	2,00	2,00	#NULL!	#NULL!
48,00	1,00	2,00	2,00	2,00	2,00	2,00	#NULL!	#NULL!
63,00	1,00	2,00	1,00	1,00	1,00	2,00	#NULL!	#NULL!
76,00	1,00	2,00	2,00	1,00	1,00	2,00	#NULL!	#NULL!
48,00	1,00	2,00	2,00	2,00	2,00	1,00	50,00	#NULL!
84,00	2,00	1,00	2,00	1,00	2,00	2,00	#NULL!	#NULL!
50,00	1,00	2,00	2,00	2,00	2,00	2,00	#NULL!	#NULL!
50,00	1,00	2,00	2,00	2,00	2,00	2,00	#NULL!	#NULL!
58,00	1,00	2,00	2,00	2,00	2,00	1,00	40,00	#NULL!
57,00	1,00	2,00	2,00	2,00	2,00	1,00	25,00	#NULL!
56,00	1,00	2,00	2,00	2,00	2,00	2,00	#NULL!	#NULL!
70,00	1,00	2,00	2,00	2,00	2,00	2,00	#NULL!	#NULL!
53,00	2,00	1,00	2,00	2,00	2,00	2,00	#NULL!	#NULL!
56,00	1,00	2,00	2,00	2,00	2,00	2,00	#NULL!	#NULL!
55,00	1,00	1,00	2,00	2,00	2,00	1,00	30,00	#NULL!
70,00	1,00	2,00	1,00	2,00	2,00	2,00	#NULL!	#NULL!
66,00	2,00	1,00	1,00	2,00	2,00	2,00	#NULL!	#NULL!

76,00	1,00	1,00	2,00	2,00	2,00	2,00	#NULL!	#NULL!
48,00	2,00	2,00	2,00	2,00	2,00	2,00	#NULL!	#NULL!
50,00	1,00	2,00	2,00	2,00	2,00	2,00	#NULL!	#NULL!
75,00	1,00	2,00	2,00	2,00	2,00	2,00	#NULL!	#NULL!
88,00	2,00	1,00	1,00	2,00	2,00	2,00	#NULL!	#NULL!
77,00	1,00	1,00	2,00	1,00	1,00	2,00	#NULL!	#NULL!
50,00	1,00	1,00	2,00	2,00	2,00	2,00	#NULL!	#NULL!
63,00	1,00	2,00	2,00	2,00	2,00	2,00	#NULL!	#NULL!
55,00	1,00	2,00	1,00	2,00	2,00	2,00	#NULL!	#NULL!
48,00	1,00	2,00	1,00	2,00	2,00	2,00	#NULL!	#NULL!
45,00	2,00	1,00	2,00	2,00	2,00	2,00	#NULL!	#NULL!
53,00	1,00	2,00	2,00	2,00	2,00	2,00	#NULL!	#NULL!
53,00	1,00	2,00	2,00	2,00	2,00	2,00	#NULL!	#NULL!
65,00	1,00	2,00	2,00	2,00	2,00	2,00	#NULL!	#NULL!
60,00	2,00	2,00	2,00	2,00	2,00	2,00	#NULL!	#NULL!
70,00	1,00	2,00	2,00	2,00	2,00	2,00	#NULL!	#NULL!
60,00	1,00	2,00	2,00	2,00	2,00	2,00	#NULL!	#NULL!
60,00	1,00	2,00	2,00	2,00	2,00	2,00	#NULL!	#NULL!
65,00	1,00	2,00	2,00	2,00	2,00	2,00	#NULL!	#NULL!
52,00	2,00	2,00	2,00	1,00	2,00	2,00	#NULL!	#NULL!
53,00	1,00	2,00	2,00	2,00	2,00	1,00	20,00	#NULL!
63,00	2,00	1,00	1,00	2,00	2,00	2,00	#NULL!	#NULL!
65,00	1,00	1,00	2,00	2,00	1,00	1,00	40,00	#NULL!
51,00	2,00	2,00	2,00	2,00	2,00	2,00	#NULL!	#NULL!
51,00	1,00	2,00	2,00	2,00	2,00	2,00	#NULL!	#NULL!
63,00	2,00	1,00	1,00	1,00	1,00	2,00	#NULL!	#NULL!
64,00	1,00	1,00	1,00	2,00	2,00	2,00	#NULL!	#NULL!
82,00	2,00	2,00	2,00	2,00	1,00	2,00	#NULL!	#NULL!
53,00	1,00	1,00	2,00	2,00	2,00	2,00	#NULL!	#NULL!
50,00	1,00	2,00	1,00	2,00	2,00	1,00	20,00	#NULL!
59,00	2,00	2,00	2,00	2,00	2,00	2,00	#NULL!	#NULL!
76,00	2,00	2,00	2,00	2,00	1,00	2,00	#NULL!	#NULL!
49,00	2,00	2,00	2,00	2,00	2,00	2,00	#NULL!	#NULL!
65,00	2,00	1,00	2,00	2,00	1,00	2,00	#NULL!	#NULL!
45,00	1,00	2,00	2,00	2,00	1,00	1,00	40,00	#NULL!
48,00	2,00	2,00	2,00	2,00	2,00	2,00	#NULL!	#NULL!
49,00	1,00	2,00	2,00	2,00	2,00	2,00	#NULL!	#NULL!
50,00	2,00	2,00	2,00	2,00	2,00	2,00	#NULL!	#NULL!
51,00	1,00	2,00	2,00	2,00	2,00	2,00	#NULL!	#NULL!
64,00	2,00	2,00	2,00	2,00	2,00	2,00	#NULL!	#NULL!
65,00	1,00	1,00	2,00	2,00	2,00	2,00	#NULL!	#NULL!
49,00	1,00	2,00	2,00	2,00	2,00	2,00	#NULL!	#NULL!
48,00	2,00	2,00	2,00	2,00	2,00	2,00	#NULL!	#NULL!
45,00	2,00	2,00	2,00	2,00	2,00	2,00	#NULL!	#NULL!
48,00	1,00	2,00	2,00	2,00	2,00	2,00	#NULL!	#NULL!
45,00	2,00	2,00	2,00	2,00	2,00	2,00	#NULL!	#NULL!
56,00	1,00	1,00	2,00	2,00	2,00	2,00	#NULL!	#NULL!
48,00	2,00	2,00	2,00	2,00	2,00	2,00	#NULL!	#NULL!
45,00	1,00	2,00	2,00	2,00	2,00	2,00	#NULL!	#NULL!
46,00	1,00	2,00	2,00	2,00	2,00	2,00	#NULL!	#NULL!

45,00	2,00	2,00	2,00	2,00	2,00	2,00	#NULL!	#NULL!
65,00	2,00	2,00	2,00	2,00	2,00	2,00	#NULL!	#NULL!
45,00	1,00	2,00	2,00	2,00	2,00	2,00	#NULL!	#NULL!
46,00	1,00	2,00	2,00	2,00	2,00	2,00	#NULL!	#NULL!
45,00	1,00	2,00	2,00	2,00	2,00	2,00	#NULL!	#NULL!
45,00	2,00	2,00	2,00	2,00	2,00	1,00	20,00	#NULL!
46,00	2,00	2,00	2,00	2,00	2,00	2,00	#NULL!	#NULL!
45,00	2,00	2,00	2,00	2,00	2,00	2,00	#NULL!	#NULL!
46,00	1,00	2,00	1,00	2,00	2,00	2,00	#NULL!	#NULL!
52,00	1,00	2,00	2,00	2,00	1,00	2,00	#NULL!	#NULL!
45,00	2,00	2,00	2,00	2,00	1,00	2,00	#NULL!	#NULL!
48,00	2,00	2,00	2,00	2,00	2,00	2,00	#NULL!	#NULL!
52,00	1,00	1,00	2,00	2,00	2,00	2,00	#NULL!	#NULL!
58,00	1,00	1,00	2,00	2,00	2,00	2,00	#NULL!	#NULL!
58,00	1,00	1,00	1,00	2,00	2,00	2,00	#NULL!	#NULL!
45,00	2,00	2,00	2,00	2,00	2,00	2,00	#NULL!	#NULL!
58,00	1,00	1,00	2,00	2,00	2,00	2,00	#NULL!	#NULL!
52,00	2,00	1,00	2,00	1,00	1,00	2,00	#NULL!	#NULL!
54,00	1,00	2,00	1,00	2,00	2,00	2,00	#NULL!	#NULL!
58,00	2,00	1,00	2,00	1,00	2,00	2,00	#NULL!	#NULL!
60,00	1,00	2,00	2,00	2,00	2,00	2,00	#NULL!	#NULL!
52,00	1,00	2,00	2,00	2,00	2,00	1,00	80,00	#NULL!
62,00	2,00	2,00	2,00	2,00	2,00	1,00	15,00	#NULL!
45,00	1,00	2,00	2,00	2,00	2,00	1,00	30,00	#NULL!
47,00	1,00	1,00	1,00	2,00	2,00	1,00	15,00	#NULL!
52,00	1,00	2,00	2,00	2,00	2,00	1,00	40,00	#NULL!
54,00	2,00	2,00	2,00	2,00	2,00	2,00	#NULL!	#NULL!
54,00	2,00	1,00	2,00	1,00	2,00	2,00	#NULL!	#NULL!
60,00	1,00	1,00	2,00	1,00	1,00	2,00	#NULL!	#NULL!
45,00	1,00	2,00	2,00	2,00	2,00	1,00	15,00	#NULL!
48,00	2,00	2,00	2,00	2,00	2,00	2,00	#NULL!	#NULL!
48,00	1,00	2,00	2,00	2,00	2,00	2,00	#NULL!	#NULL!
52,00	2,00	2,00	2,00	2,00	2,00	2,00	#NULL!	#NULL!
54,00	2,00	1,00	1,00	1,00	1,00	2,00	#NULL!	#NULL!
56,00	2,00	1,00	2,00	2,00	2,00	2,00	#NULL!	#NULL!
45,00	2,00	2,00	2,00	2,00	2,00	2,00	#NULL!	#NULL!
52,00	1,00	1,00	2,00	1,00	2,00	2,00	#NULL!	#NULL!
48,00	2,00	1,00	2,00	2,00	2,00	1,00	30,00	#NULL!
45,00	2,00	1,00	1,00	2,00	2,00	2,00	#NULL!	#NULL!
46,00	1,00	2,00	2,00	2,00	2,00	1,00	30,00	#NULL!
48,00	2,00	1,00	1,00	2,00	2,00	2,00	#NULL!	#NULL!
45,00	2,00	2,00	2,00	2,00	2,00	2,00	#NULL!	#NULL!
47,00	1,00	1,00	1,00	1,00	1,00	2,00	#NULL!	#NULL!
46,00	2,00	2,00	2,00	2,00	2,00	2,00	#NULL!	#NULL!
49,00	1,00	2,00	2,00	2,00	2,00	2,00	30,00	8,00
80,00	2,00	1,00	2,00	2,00	2,00	1,00	40,00	#NULL!
45,00	1,00	2,00	2,00	2,00	2,00	2,00	#NULL!	#NULL!
52,00	2,00	1,00	2,00	1,00	1,00	2,00	#NULL!	#NULL!
50,00	2,00	2,00	2,00	2,00	2,00	2,00	#NULL!	#NULL!
52,00	1,00	2,00	2,00	2,00	2,00	1,00	30,00	15,00

45,00	2,00	2,00	2,00	2,00	2,00	2,00	#NULL!	#NULL!
48,00	2,00	1,00	1,00	2,00	2,00	2,00	#NULL!	#NULL!
50,00	1,00	2,00	2,00	2,00	2,00	2,00	#NULL!	#NULL!
53,00	1,00	2,00	2,00	2,00	2,00	2,00	#NULL!	#NULL!
49,00	2,00	2,00	2,00	2,00	2,00	2,00	#NULL!	#NULL!
61,00	1,00	1,00	2,00	2,00	1,00	1,00	10,00	20,00
71,00	1,00	2,00	2,00	2,00	2,00	2,00	#NULL!	#NULL!
61,00	1,00	1,00	2,00	2,00	1,00	2,00	#NULL!	#NULL!
45,00	2,00	2,00	2,00	2,00	2,00	2,00	#NULL!	#NULL!
72,00	1,00	2,00	2,00	2,00	2,00	2,00	#NULL!	#NULL!
59,00	2,00	2,00	2,00	2,00	2,00	2,00	#NULL!	#NULL!
45,00	1,00	2,00	1,00	2,00	1,00	1,00	5,00	#NULL!
54,00	1,00	2,00	2,00	2,00	2,00	2,00	#NULL!	#NULL!
47,00	2,00	2,00	2,00	1,00	2,00	2,00	#NULL!	#NULL!
48,00	2,00	2,00	2,00	2,00	2,00	2,00	#NULL!	#NULL!
55,00	1,00	2,00	2,00	2,00	2,00	1,00	20,00	#NULL!
46,00	1,00	2,00	2,00	2,00	2,00	2,00	#NULL!	#NULL!
59,00	2,00	1,00	1,00	1,00	2,00	2,00	#NULL!	#NULL!
64,00	1,00	1,00	2,00	1,00	2,00	2,00	#NULL!	#NULL!
68,00	2,00	1,00	1,00	2,00	1,00	2,00	#NULL!	#NULL!
72,00	1,00	2,00	2,00	2,00	2,00	2,00	#NULL!	#NULL!
58,00	2,00	1,00	1,00	1,00	1,00	1,00	20,00	#NULL!
62,00	1,00	1,00	2,00	2,00	2,00	1,00	25,00	#NULL!
68,00	2,00	1,00	1,00	2,00	1,00	2,00	#NULL!	#NULL!
72,00	1,00	2,00	2,00	2,00	2,00	2,00	#NULL!	#NULL!
67,00	1,00	1,00	2,00	2,00	2,00	2,00	#NULL!	#NULL!
62,00	2,00	1,00	2,00	2,00	2,00	2,00	#NULL!	#NULL!
59,00	2,00	1,00	1,00	1,00	1,00	2,00	#NULL!	#NULL!
70,00	1,00	1,00	1,00	1,00	2,00	1,00	30,00	#NULL!
58,00	1,00	1,00	2,00	2,00	2,00	1,00	40,00	#NULL!
60,00	1,00	2,00	2,00	2,00	2,00	2,00	#NULL!	#NULL!
68,00	2,00	1,00	2,00	1,00	1,00	2,00	#NULL!	#NULL!
60,00	2,00	2,00	2,00	2,00	2,00	2,00	#NULL!	#NULL!
45,00	2,00	2,00	2,00	2,00	2,00	2,00	#NULL!	#NULL!
45,00	2,00	2,00	2,00	2,00	2,00	2,00	#NULL!	#NULL!
70,00	2,00	1,00	2,00	2,00	2,00	2,00	#NULL!	#NULL!
71,00	1,00	2,00	2,00	2,00	2,00	2,00	#NULL!	#NULL!
45,00	2,00	2,00	2,00	2,00	2,00	2,00	#NULL!	#NULL!
49,00	2,00	2,00	2,00	2,00	2,00	2,00	#NULL!	#NULL!
55,00	2,00	1,00	2,00	2,00	2,00	2,00	#NULL!	#NULL!
54,00	2,00	2,00	2,00	2,00	2,00	2,00	#NULL!	#NULL!
68,00	2,00	1,00	1,00	1,00	1,00	2,00	114,00	1,00
71,00	1,00	1,00	1,00	1,00	1,00	1,00	60,00	#NULL!
56,00	1,00	2,00	2,00	2,00	2,00	2,00	#NULL!	#NULL!
50,00	2,00	2,00	2,00	2,00	2,00	2,00	#NULL!	#NULL!
50,00	2,00	2,00	2,00	2,00	2,00	2,00	#NULL!	#NULL!
46,00	2,00	2,00	2,00	2,00	2,00	2,00	#NULL!	#NULL!
72,00	1,00	1,00	2,00	1,00	1,00	1,00	30,00	#NULL!
70,00	1,00	1,00	2,00	2,00	2,00	2,00	#NULL!	#NULL!
56,00	1,00	2,00	2,00	2,00	2,00	2,00	#NULL!	#NULL!

58,00	1,00	2,00	2,00	2,00	2,00	2,00	#NULL!	#NULL!
56,00	1,00	1,00	2,00	2,00	2,00	2,00	#NULL!	#NULL!
50,00	2,00	1,00	1,00	2,00	2,00	2,00	#NULL!	#NULL!
50,00	2,00	2,00	2,00	2,00	2,00	2,00	#NULL!	#NULL!
46,00	2,00	2,00	2,00	2,00	2,00	2,00	#NULL!	#NULL!
83,00	2,00	1,00	2,00	2,00	2,00	2,00	#NULL!	#NULL!
56,00	2,00	1,00	2,00	2,00	2,00	2,00	#NULL!	#NULL!
64,00	1,00	1,00	1,00	1,00	1,00	2,00	#NULL!	#NULL!
75,00	2,00	1,00	1,00	1,00	2,00	2,00	#NULL!	#NULL!
50,00	1,00	2,00	2,00	1,00	2,00	1,00	30,00	#NULL!
64,00	1,00	1,00	2,00	1,00	2,00	2,00	#NULL!	#NULL!
65,00	2,00	1,00	2,00	2,00	2,00	1,00	20,00	#NULL!
58,00	1,00	2,00	2,00	1,00	2,00	2,00	#NULL!	#NULL!
48,00	1,00	2,00	1,00	2,00	2,00	2,00	#NULL!	#NULL!
48,00	2,00	2,00	2,00	2,00	2,00	2,00	#NULL!	#NULL!
67,00	1,00	2,00	1,00	2,00	2,00	2,00	#NULL!	#NULL!
65,00	2,00	1,00	1,00	2,00	2,00	2,00	#NULL!	#NULL!
55,00	2,00	2,00	2,00	2,00	2,00	2,00	#NULL!	#NULL!
60,00	1,00	2,00	2,00	2,00	2,00	2,00	#NULL!	#NULL!
56,00	2,00	2,00	2,00	2,00	2,00	2,00	#NULL!	#NULL!
55,00	2,00	2,00	2,00	2,00	2,00	2,00	#NULL!	#NULL!
55,00	1,00	2,00	2,00	2,00	2,00	2,00	#NULL!	#NULL!
48,00	2,00	2,00	2,00	2,00	2,00	2,00	#NULL!	#NULL!
45,00	2,00	2,00	2,00	2,00	2,00	2,00	#NULL!	#NULL!
47,00	1,00	2,00	2,00	2,00	2,00	2,00	#NULL!	#NULL!
45,00	2,00	2,00	2,00	2,00	2,00	2,00	#NULL!	#NULL!
45,00	1,00	2,00	2,00	2,00	2,00	2,00	#NULL!	#NULL!
50,00	2,00	2,00	2,00	2,00	2,00	2,00	#NULL!	#NULL!
55,00	1,00	2,00	2,00	2,00	2,00	2,00	#NULL!	#NULL!
50,00	2,00	2,00	2,00	2,00	2,00	2,00	#NULL!	#NULL!
55,00	1,00	2,00	2,00	2,00	2,00	2,00	#NULL!	#NULL!
45,00	2,00	2,00	2,00	2,00	2,00	2,00	#NULL!	#NULL!
45,00	1,00	2,00	2,00	2,00	2,00	2,00	#NULL!	#NULL!
60,00	1,00	2,00	2,00	2,00	2,00	2,00	#NULL!	#NULL!
55,00	2,00	2,00	2,00	2,00	2,00	2,00	#NULL!	#NULL!
58,00	2,00	2,00	2,00	2,00	2,00	2,00	#NULL!	#NULL!
51,00	1,00	2,00	2,00	2,00	2,00	2,00	#NULL!	#NULL!
54,00	2,00	2,00	2,00	2,00	2,00	2,00	#NULL!	#NULL!
45,00	2,00	2,00	2,00	2,00	2,00	2,00	#NULL!	#NULL!
46,00	2,00	2,00	2,00	2,00	2,00	2,00	#NULL!	#NULL!
52,00	1,00	2,00	2,00	2,00	2,00	2,00	#NULL!	#NULL!
48,00	2,00	2,00	2,00	2,00	2,00	2,00	#NULL!	#NULL!
56,00	1,00	2,00	2,00	2,00	2,00	2,00	#NULL!	#NULL!
55,00	2,00	2,00	2,00	2,00	2,00	2,00	#NULL!	#NULL!
54,00	2,00	2,00	2,00	2,00	2,00	2,00	#NULL!	#NULL!
46,00	2,00	2,00	2,00	2,00	2,00	2,00	#NULL!	#NULL!
50,00	1,00	2,00	2,00	2,00	2,00	2,00	#NULL!	#NULL!
45,00	2,00	2,00	2,00	2,00	2,00	2,00	#NULL!	#NULL!
47,00	2,00	2,00	2,00	2,00	2,00	2,00	#NULL!	#NULL!
60,00	1,00	2,00	2,00	2,00	2,00	2,00	#NULL!	#NULL!

85,00	2,00	2,00	2,00	2,00	2,00	2,00	#NULL!	#NULL!
80,00	2,00	1,00	2,00	2,00	2,00	2,00	#NULL!	#NULL!
50,00	2,00	2,00	2,00	2,00	2,00	2,00	#NULL!	#NULL!
50,00	2,00	2,00	2,00	2,00	2,00	2,00	#NULL!	#NULL!
58,00	2,00	2,00	2,00	2,00	2,00	2,00	#NULL!	#NULL!
45,00	2,00	2,00	2,00	2,00	2,00	2,00	#NULL!	#NULL!
58,00	2,00	2,00	2,00	2,00	2,00	2,00	#NULL!	#NULL!
56,00	1,00	2,00	2,00	2,00	2,00	2,00	#NULL!	#NULL!
55,00	2,00	2,00	2,00	2,00	2,00	2,00	#NULL!	#NULL!
50,00	2,00	2,00	2,00	2,00	2,00	2,00	#NULL!	#NULL!
58,00	1,00	2,00	2,00	2,00	2,00	2,00	#NULL!	#NULL!
55,00	1,00	2,00	2,00	2,00	2,00	2,00	#NULL!	#NULL!
53,00	1,00	2,00	2,00	2,00	2,00	2,00	#NULL!	#NULL!
57,00	2,00	2,00	2,00	2,00	2,00	2,00	#NULL!	#NULL!
45,00	1,00	2,00	2,00	2,00	2,00	2,00	#NULL!	#NULL!
62,00	1,00	2,00	2,00	2,00	2,00	2,00	#NULL!	#NULL!
47,00	2,00	2,00	2,00	2,00	2,00	2,00	#NULL!	#NULL!
65,00	1,00	2,00	2,00	2,00	2,00	2,00	#NULL!	#NULL!
62,00	2,00	2,00	2,00	2,00	2,00	2,00	#NULL!	#NULL!
63,00	2,00	2,00	2,00	2,00	2,00	2,00	#NULL!	#NULL!
63,00	2,00	2,00	2,00	2,00	2,00	2,00	#NULL!	#NULL!
55,00	1,00	2,00	2,00	2,00	2,00	2,00	#NULL!	#NULL!
47,00	2,00	2,00	2,00	2,00	2,00	2,00	#NULL!	#NULL!
45,00	1,00	2,00	2,00	2,00	2,00	2,00	#NULL!	#NULL!
68,00	2,00	2,00	2,00	2,00	2,00	2,00	#NULL!	#NULL!
48,00	2,00	2,00	2,00	2,00	2,00	2,00	#NULL!	#NULL!
57,00	1,00	2,00	2,00	2,00	2,00	2,00	#NULL!	#NULL!
45,00	1,00	2,00	2,00	2,00	2,00	2,00	#NULL!	#NULL!
58,00	2,00	2,00	2,00	2,00	2,00	2,00	#NULL!	#NULL!
52,00	1,00	2,00	2,00	2,00	2,00	2,00	#NULL!	#NULL!
55,00	1,00	2,00	2,00	2,00	2,00	2,00	#NULL!	#NULL!
45,00	1,00	2,00	2,00	2,00	2,00	2,00	#NULL!	#NULL!
60,00	1,00	2,00	2,00	2,00	2,00	2,00	#NULL!	#NULL!
60,00	2,00	2,00	2,00	2,00	2,00	2,00	#NULL!	#NULL!
47,00	2,00	2,00	2,00	2,00	2,00	2,00	#NULL!	#NULL!
55,00	1,00	2,00	2,00	2,00	2,00	2,00	#NULL!	#NULL!
45,00	1,00	2,00	2,00	2,00	2,00	2,00	#NULL!	#NULL!
48,00	1,00	2,00	2,00	2,00	2,00	2,00	#NULL!	#NULL!
55,00	1,00	2,00	2,00	2,00	2,00	2,00	#NULL!	#NULL!
52,00	1,00	2,00	2,00	2,00	2,00	2,00	#NULL!	#NULL!
54,00	2,00	2,00	2,00	2,00	2,00	2,00	#NULL!	#NULL!
50,00	1,00	2,00	2,00	2,00	2,00	2,00	#NULL!	#NULL!
47,00	2,00	2,00	2,00	2,00	2,00	2,00	#NULL!	#NULL!
51,00	1,00	2,00	2,00	2,00	2,00	2,00	#NULL!	#NULL!
55,00	1,00	2,00	2,00	2,00	2,00	2,00	#NULL!	#NULL!
54,00	2,00	2,00	2,00	2,00	2,00	2,00	#NULL!	#NULL!
45,00	1,00	2,00	2,00	2,00	2,00	2,00	#NULL!	#NULL!
45,00	2,00	2,00	2,00	2,00	2,00	2,00	#NULL!	#NULL!
51,00	2,00	2,00	2,00	2,00	2,00	2,00	#NULL!	#NULL!
60,00	1,00	2,00	2,00	2,00	2,00	2,00	#NULL!	#NULL!

55,00	1,00	2,00	2,00	2,00	2,00	2,00	#NULL!	#NULL!
55,00	1,00	2,00	2,00	2,00	2,00	2,00	#NULL!	#NULL!
76,00	2,00	2,00	2,00	2,00	2,00	2,00	#NULL!	#NULL!
46,00	2,00	2,00	2,00	2,00	2,00	2,00	#NULL!	#NULL!
45,00	1,00	2,00	2,00	2,00	2,00	2,00	#NULL!	#NULL!
54,00	2,00	1,00	2,00	2,00	2,00	2,00	#NULL!	#NULL!
63,00	1,00	2,00	2,00	1,00	1,00	1,00	15,00	#NULL!
87,00	2,00	1,00	2,00	2,00	2,00	2,00	#NULL!	#NULL!
74,00	2,00	1,00	1,00	1,00	1,00	2,00	#NULL!	#NULL!
70,00	2,00	2,00	1,00	2,00	2,00	2,00	#NULL!	#NULL!
83,00	1,00	1,00	2,00	1,00	2,00	2,00	#NULL!	#NULL!
53,00	2,00	2,00	2,00	2,00	2,00	2,00	#NULL!	#NULL!
60,00	1,00	2,00	2,00	2,00	2,00	2,00	#NULL!	#NULL!
83,00	2,00	1,00	2,00	2,00	2,00	2,00	#NULL!	#NULL!
67,00	2,00	1,00	2,00	1,00	2,00	2,00	#NULL!	#NULL!
73,00	1,00	2,00	2,00	2,00	1,00	1,00	10,00	#NULL!
78,00	1,00	2,00	1,00	2,00	1,00	1,00	15,00	#NULL!
50,00	2,00	2,00	2,00	2,00	2,00	2,00	#NULL!	#NULL!
57,00	1,00	1,00	2,00	2,00	2,00	2,00	#NULL!	#NULL!
61,00	1,00	1,00	1,00	2,00	2,00	2,00	#NULL!	#NULL!
57,00	1,00	2,00	2,00	1,00	2,00	2,00	#NULL!	#NULL!
51,00	2,00	2,00	2,00	2,00	2,00	2,00	#NULL!	#NULL!
51,00	1,00	2,00	2,00	2,00	2,00	2,00	#NULL!	#NULL!
89,00	2,00	1,00	2,00	2,00	2,00	2,00	#NULL!	#NULL!
79,00	2,00	1,00	1,00	2,00	2,00	2,00	#NULL!	#NULL!
82,00	1,00	2,00	2,00	1,00	2,00	2,00	#NULL!	#NULL!
71,00	2,00	1,00	1,00	1,00	1,00	2,00	#NULL!	#NULL!
76,00	1,00	1,00	1,00	2,00	2,00	2,00	#NULL!	#NULL!
60,00	2,00	2,00	1,00	2,00	2,00	2,00	#NULL!	#NULL!
46,00	1,00	2,00	2,00	2,00	2,00	2,00	#NULL!	#NULL!
48,00	1,00	2,00	2,00	2,00	2,00	2,00	#NULL!	#NULL!
67,00	2,00	1,00	2,00	2,00	2,00	2,00	#NULL!	#NULL!
60,00	2,00	1,00	1,00	2,00	2,00	2,00	#NULL!	#NULL!
65,00	1,00	1,00	2,00	2,00	2,00	2,00	#NULL!	#NULL!
67,00	2,00	2,00	1,00	2,00	2,00	2,00	#NULL!	#NULL!
71,00	2,00	2,00	2,00	1,00	2,00	2,00	#NULL!	#NULL!
66,00	2,00	1,00	2,00	2,00	2,00	2,00	#NULL!	#NULL!
95,00	2,00	1,00	1,00	1,00	1,00	2,00	#NULL!	#NULL!
52,00	2,00	2,00	2,00	2,00	2,00	2,00	#NULL!	#NULL!
57,00	2,00	2,00	2,00	2,00	2,00	2,00	#NULL!	#NULL!
56,00	2,00	2,00	1,00	2,00	2,00	2,00	#NULL!	#NULL!
54,00	1,00	2,00	2,00	2,00	2,00	2,00	#NULL!	#NULL!
54,00	1,00	2,00	2,00	2,00	2,00	2,00	#NULL!	#NULL!
47,00	2,00	2,00	2,00	2,00	2,00	2,00	#NULL!	#NULL!
56,00	2,00	2,00	2,00	2,00	1,00	2,00	#NULL!	#NULL!
58,00	1,00	2,00	2,00	2,00	2,00	2,00	#NULL!	#NULL!
68,00	2,00	2,00	2,00	2,00	2,00	2,00	#NULL!	#NULL!
51,00	1,00	2,00	2,00	2,00	2,00	2,00	#NULL!	#NULL!
72,00	1,00	1,00	2,00	1,00	2,00	2,00	#NULL!	#NULL!
49,00	2,00	2,00	2,00	2,00	2,00	2,00	#NULL!	#NULL!

57,00	2,00	1,00	2,00	1,00	2,00	2,00	#NULL!	#NULL!
53,00	1,00	2,00	2,00	1,00	2,00	1,00	30,00	#NULL!
56,00	2,00	1,00	2,00	1,00	2,00	2,00	#NULL!	#NULL!
65,00	1,00	1,00	2,00	2,00	2,00	2,00	#NULL!	#NULL!
74,00	2,00	1,00	1,00	1,00	1,00	2,00	#NULL!	#NULL!
83,00	2,00	1,00	1,00	2,00	2,00	2,00	#NULL!	#NULL!
46,00	2,00	2,00	2,00	2,00	2,00	2,00	#NULL!	#NULL!
52,00	1,00	1,00	2,00	2,00	2,00	2,00	#NULL!	#NULL!
54,00	1,00	1,00	2,00	2,00	2,00	2,00	#NULL!	#NULL!
72,00	2,00	1,00	1,00	2,00	2,00	2,00	#NULL!	#NULL!
51,00	1,00	1,00	2,00	1,00	2,00	1,00	20,00	#NULL!
65,00	2,00	1,00	2,00	2,00	2,00	2,00	#NULL!	#NULL!
68,00	1,00	1,00	1,00	2,00	2,00	2,00	#NULL!	#NULL!
89,00	2,00	1,00	2,00	1,00	2,00	2,00	#NULL!	#NULL!
56,00	2,00	1,00	2,00	2,00	2,00	1,00	5,00	#NULL!
55,00	1,00	2,00	2,00	2,00	2,00	2,00	#NULL!	#NULL!
55,00	2,00	2,00	2,00	2,00	2,00	2,00	#NULL!	#NULL!
64,00	1,00	2,00	2,00	2,00	2,00	2,00	#NULL!	#NULL!
58,00	2,00	2,00	2,00	2,00	2,00	2,00	#NULL!	#NULL!
45,00	2,00	2,00	2,00	2,00	2,00	2,00	#NULL!	#NULL!
46,00	1,00	2,00	2,00	2,00	2,00	2,00	#NULL!	#NULL!
45,00	2,00	2,00	2,00	2,00	2,00	2,00	#NULL!	#NULL!
45,00	2,00	2,00	2,00	2,00	2,00	2,00	#NULL!	#NULL!
50,00	1,00	2,00	2,00	2,00	2,00	2,00	#NULL!	#NULL!
60,00	1,00	2,00	2,00	2,00	2,00	2,00	#NULL!	#NULL!
73,00	2,00	2,00	2,00	1,00	1,00	2,00	#NULL!	#NULL!
45,00	2,00	2,00	2,00	2,00	2,00	2,00	#NULL!	#NULL!
68,00	1,00	2,00	2,00	2,00	2,00	2,00	#NULL!	#NULL!
65,00	2,00	2,00	2,00	2,00	2,00	2,00	#NULL!	#NULL!
60,00	2,00	2,00	2,00	2,00	2,00	2,00	#NULL!	#NULL!
62,00	1,00	2,00	2,00	2,00	2,00	2,00	#NULL!	#NULL!
73,00	1,00	2,00	2,00	2,00	2,00	2,00	#NULL!	#NULL!
61,00	1,00	2,00	2,00	2,00	2,00	2,00	#NULL!	#NULL!
49,00	1,00	2,00	2,00	2,00	2,00	2,00	#NULL!	#NULL!
50,00	2,00	1,00	2,00	2,00	2,00	2,00	#NULL!	#NULL!
58,00	2,00	2,00	2,00	2,00	2,00	2,00	#NULL!	#NULL!
50,00	2,00	2,00	2,00	2,00	2,00	2,00	#NULL!	#NULL!
51,00	2,00	2,00	2,00	2,00	2,00	2,00	#NULL!	#NULL!
55,00	1,00	1,00	2,00	2,00	2,00	2,00	#NULL!	#NULL!
50,00	2,00	1,00	2,00	2,00	2,00	2,00	#NULL!	#NULL!
55,00	1,00	2,00	2,00	2,00	2,00	2,00	#NULL!	#NULL!
68,00	2,00	1,00	2,00	1,00	2,00	2,00	#NULL!	#NULL!
64,00	1,00	2,00	1,00	1,00	1,00	2,00	#NULL!	#NULL!
74,00	2,00	1,00	2,00	2,00	2,00	2,00	30,00	20,00
46,00	1,00	2,00	2,00	2,00	2,00	2,00	#NULL!	#NULL!
66,00	2,00	1,00	2,00	2,00	2,00	2,00	#NULL!	#NULL!
67,00	2,00	1,00	1,00	2,00	2,00	2,00	#NULL!	#NULL!
78,00	2,00	1,00	2,00	2,00	2,00	2,00	#NULL!	#NULL!
80,00	1,00	2,00	2,00	2,00	1,00	2,00	#NULL!	#NULL!
49,00	1,00	2,00	2,00	2,00	2,00	2,00	#NULL!	#NULL!

76,00	2,00	1,00	2,00	1,00	2,00	2,00	#NULL!	#NULL!
77,00	2,00	1,00	2,00	2,00	2,00	2,00	#NULL!	#NULL!
50,00	2,00	2,00	2,00	2,00	2,00	2,00	#NULL!	#NULL!
56,00	2,00	2,00	2,00	2,00	2,00	2,00	#NULL!	#NULL!
60,00	1,00	1,00	2,00	2,00	2,00	2,00	#NULL!	#NULL!
45,00	2,00	2,00	2,00	2,00	2,00	2,00	#NULL!	#NULL!
45,00	1,00	2,00	2,00	2,00	2,00	2,00	#NULL!	#NULL!
66,00	1,00	2,00	2,00	2,00	2,00	2,00	#NULL!	#NULL!
56,00	2,00	1,00	2,00	2,00	2,00	2,00	#NULL!	#NULL!
55,00	1,00	2,00	2,00	2,00	2,00	1,00	25,00	#NULL!
53,00	2,00	1,00	2,00	2,00	2,00	2,00	#NULL!	#NULL!
57,00	1,00	2,00	2,00	2,00	2,00	2,00	#NULL!	#NULL!
68,00	2,00	1,00	2,00	1,00	2,00	2,00	#NULL!	#NULL!
45,00	2,00	2,00	2,00	2,00	2,00	2,00	#NULL!	#NULL!
55,00	2,00	1,00	2,00	1,00	1,00	1,00	15,00	#NULL!
64,00	1,00	2,00	2,00	1,00	1,00	1,00	40,00	#NULL!
59,00	1,00	1,00	2,00	2,00	2,00	1,00	7,00	#NULL!
87,00	1,00	1,00	2,00	1,00	1,00	1,00	100,00	#NULL!
52,00	2,00	1,00	1,00	2,00	2,00	2,00	#NULL!	#NULL!
62,00	2,00	1,00	2,00	2,00	2,00	2,00	#NULL!	#NULL!
45,00	2,00	2,00	2,00	2,00	2,00	2,00	#NULL!	#NULL!
52,00	1,00	1,00	2,00	2,00	2,00	1,00	15,00	#NULL!
49,00	1,00	2,00	2,00	2,00	2,00	1,00	5,00	#NULL!
59,00	1,00	2,00	1,00	2,00	2,00	2,00	#NULL!	#NULL!
78,00	2,00	1,00	1,00	1,00	1,00	2,00	#NULL!	#NULL!
79,00	2,00	1,00	1,00	2,00	2,00	2,00	#NULL!	#NULL!
45,00	1,00	2,00	2,00	2,00	2,00	2,00	#NULL!	#NULL!
46,00	2,00	2,00	2,00	2,00	2,00	2,00	#NULL!	#NULL!
45,00	1,00	2,00	2,00	2,00	2,00	2,00	#NULL!	#NULL!
45,00	2,00	2,00	2,00	2,00	2,00	2,00	#NULL!	#NULL!
45,00	1,00	2,00	2,00	2,00	2,00	2,00	#NULL!	#NULL!
56,00	1,00	2,00	1,00	2,00	1,00	1,00	80,00	#NULL!
46,00	1,00	2,00	2,00	2,00	2,00	2,00	#NULL!	#NULL!
45,00	1,00	2,00	2,00	2,00	2,00	2,00	#NULL!	#NULL!
46,00	1,00	2,00	2,00	1,00	2,00	1,00	30,00	#NULL!
60,00	1,00	2,00	1,00	2,00	2,00	2,00	#NULL!	#NULL!
45,00	1,00	2,00	2,00	2,00	2,00	2,00	#NULL!	#NULL!
51,00	2,00	2,00	2,00	2,00	2,00	2,00	#NULL!	#NULL!
50,00	2,00	2,00	2,00	2,00	2,00	2,00	#NULL!	#NULL!
63,00	2,00	1,00	1,00	2,00	2,00	2,00	#NULL!	#NULL!
46,00	1,00	2,00	2,00	2,00	2,00	1,00	20,00	#NULL!
82,00	1,00	2,00	2,00	1,00	1,00	1,00	50,00	#NULL!
47,00	2,00	2,00	2,00	2,00	2,00	2,00	#NULL!	#NULL!
49,00	2,00	2,00	2,00	2,00	2,00	2,00	#NULL!	#NULL!
60,00	1,00	1,00	2,00	2,00	2,00	1,00	80,00	#NULL!
78,00	2,00	1,00	2,00	2,00	1,00	2,00	#NULL!	#NULL!
54,00	2,00	1,00	2,00	2,00	1,00	1,00	15,00	#NULL!
54,00	2,00	2,00	2,00	1,00	2,00	2,00	#NULL!	#NULL!
66,00	2,00	1,00	2,00	1,00	2,00	2,00	#NULL!	#NULL!
67,00	1,00	2,00	2,00	2,00	2,00	2,00	#NULL!	#NULL!

72,00	1,00	1,00	2,00	1,00	2,00	2,00	#NULL!	#NULL!
78,00	1,00	1,00	2,00	2,00	1,00	2,00	#NULL!	#NULL!
66,00	2,00	1,00	1,00	2,00	1,00	2,00	#NULL!	#NULL!
77,00	1,00	1,00	2,00	2,00	2,00	2,00	#NULL!	#NULL!
46,00	1,00	2,00	2,00	2,00	2,00	2,00	#NULL!	#NULL!
50,00	1,00	2,00	2,00	2,00	2,00	1,00	30,00	#NULL!
55,00	2,00	2,00	2,00	2,00	2,00	2,00	#NULL!	#NULL!
50,00	2,00	1,00	2,00	2,00	2,00	2,00	#NULL!	#NULL!
45,00	2,00	2,00	2,00	2,00	2,00	2,00	#NULL!	#NULL!
55,00	2,00	2,00	2,00	2,00	1,00	2,00	#NULL!	#NULL!
58,00	1,00	2,00	2,00	2,00	1,00	2,00	#NULL!	#NULL!
46,00	2,00	1,00	2,00	2,00	2,00	2,00	#NULL!	#NULL!
48,00	1,00	2,00	2,00	2,00	2,00	2,00	#NULL!	#NULL!
45,00	1,00	2,00	2,00	2,00	2,00	2,00	#NULL!	#NULL!
63,00	1,00	1,00	2,00	2,00	2,00	2,00	#NULL!	#NULL!
86,00	2,00	1,00	1,00	1,00	1,00	2,00	#NULL!	#NULL!
69,00	1,00	2,00	1,00	2,00	2,00	1,00	3,00	#NULL!
84,00	2,00	1,00	2,00	2,00	2,00	2,00	#NULL!	#NULL!
65,00	2,00	2,00	2,00	2,00	2,00	2,00	#NULL!	#NULL!
55,00	1,00	2,00	2,00	2,00	2,00	1,00	15,00	#NULL!
74,00	2,00	2,00	1,00	1,00	1,00	2,00	#NULL!	#NULL!
66,00	1,00	1,00	2,00	2,00	2,00	1,00	20,00	#NULL!
69,00	2,00	2,00	2,00	2,00	1,00	2,00	#NULL!	#NULL!
76,00	1,00	2,00	2,00	2,00	2,00	2,00	#NULL!	#NULL!
79,00	2,00	2,00	1,00	1,00	2,00	2,00	#NULL!	#NULL!
53,00	2,00	2,00	2,00	2,00	1,00	2,00	#NULL!	#NULL!
76,00	2,00	1,00	2,00	1,00	2,00	2,00	#NULL!	#NULL!
84,00	1,00	1,00	1,00	2,00	2,00	2,00	#NULL!	#NULL!
45,00	2,00	2,00	2,00	2,00	2,00	2,00	#NULL!	#NULL!
47,00	2,00	2,00	2,00	2,00	2,00	1,00	10,00	#NULL!
54,00	1,00	2,00	2,00	2,00	2,00	1,00	15,00	#NULL!
54,00	1,00	2,00	2,00	2,00	2,00	2,00	#NULL!	#NULL!
73,00	2,00	2,00	1,00	2,00	1,00	2,00	#NULL!	#NULL!
68,00	2,00	1,00	1,00	2,00	2,00	2,00	#NULL!	#NULL!
75,00	2,00	1,00	2,00	2,00	2,00	2,00	#NULL!	#NULL!
50,00	2,00	2,00	2,00	2,00	2,00	1,00	5,00	#NULL!
45,00	2,00	2,00	2,00	2,00	2,00	1,00	2,00	#NULL!
53,00	2,00	1,00	22,00	2,00	2,00	2,00	#NULL!	#NULL!
78,00	2,00	1,00	1,00	2,00	2,00	2,00	#NULL!	#NULL!
72,00	1,00	2,00	1,00	2,00	2,00	2,00	#NULL!	#NULL!
59,00	2,00	2,00	2,00	1,00	2,00	2,00	#NULL!	#NULL!
63,00	1,00	2,00	1,00	2,00	2,00	2,00	#NULL!	#NULL!
59,00	1,00	1,00	2,00	1,00	1,00	1,00	30,00	#NULL!
53,00	2,00	2,00	2,00	2,00	2,00	2,00	#NULL!	#NULL!
62,00	1,00	1,00	2,00	1,00	2,00	2,00	#NULL!	#NULL!
72,00	2,00	1,00	2,00	2,00	2,00	2,00	#NULL!	#NULL!
45,00	2,00	2,00	2,00	2,00	2,00	2,00	#NULL!	#NULL!
56,00	2,00	2,00	2,00	2,00	2,00	2,00	#NULL!	#NULL!
51,00	2,00	2,00	2,00	2,00	2,00	2,00	#NULL!	#NULL!
58,00	2,00	1,00	1,00	2,00	2,00	2,00	#NULL!	#NULL!

47,00	2,00	2,00	2,00	2,00	2,00	2,00	#NULL!	#NULL!
51,00	1,00	1,00	2,00	2,00	2,00	2,00	#NULL!	#NULL!
55,00	1,00	1,00	1,00	2,00	2,00	2,00	#NULL!	#NULL!
50,00	2,00	2,00	2,00	2,00	2,00	2,00	#NULL!	#NULL!
45,00	2,00	2,00	2,00	2,00	2,00	2,00	#NULL!	#NULL!
50,00	1,00	2,00	2,00	2,00	2,00	2,00	#NULL!	#NULL!
45,00	2,00	2,00	2,00	2,00	2,00	2,00	#NULL!	#NULL!
45,00	2,00	2,00	2,00	2,00	2,00	2,00	#NULL!	#NULL!
50,00	1,00	2,00	2,00	2,00	2,00	2,00	#NULL!	#NULL!
45,00	1,00	2,00	2,00	2,00	2,00	2,00	#NULL!	#NULL!
45,00	2,00	2,00	2,00	2,00	2,00	2,00	#NULL!	#NULL!
48,00	1,00	2,00	2,00	2,00	2,00	1,00	10,00	#NULL!
58,00	1,00	2,00	1,00	2,00	2,00	2,00	#NULL!	#NULL!
51,00	2,00	2,00	2,00	2,00	2,00	2,00	#NULL!	#NULL!
53,00	2,00	2,00	2,00	2,00	2,00	1,00	30,00	#NULL!
53,00	1,00	2,00	2,00	2,00	2,00	2,00	#NULL!	#NULL!
45,00	1,00	2,00	2,00	2,00	2,00	2,00	#NULL!	#NULL!
64,00	2,00	1,00	2,00	2,00	2,00	2,00	#NULL!	#NULL!
48,00	2,00	2,00	2,00	2,00	1,00	2,00	#NULL!	#NULL!
73,00	2,00	1,00	2,00	2,00	2,00	2,00	#NULL!	#NULL!
76,00	1,00	1,00	1,00	2,00	2,00	2,00	#NULL!	#NULL!
48,00	1,00	2,00	2,00	2,00	2,00	2,00	#NULL!	#NULL!
46,00	1,00	2,00	2,00	2,00	2,00	1,00	10,00	#NULL!
51,00	2,00	2,00	2,00	2,00	2,00	2,00	#NULL!	#NULL!
45,00	1,00	2,00	2,00	2,00	2,00	1,00	5,00	#NULL!
54,00	2,00	1,00	2,00	2,00	2,00	2,00	#NULL!	#NULL!
56,00	1,00	1,00	2,00	2,00	2,00	2,00	#NULL!	#NULL!
50,00	1,00	2,00	1,00	2,00	2,00	2,00	#NULL!	#NULL!
49,00	2,00	2,00	2,00	2,00	2,00	2,00	#NULL!	#NULL!
54,00	2,00	1,00	2,00	2,00	2,00	2,00	#NULL!	#NULL!
56,00	1,00	1,00	1,00	2,00	1,00	2,00	#NULL!	#NULL!
48,00	1,00	2,00	2,00	2,00	2,00	2,00	#NULL!	#NULL!
45,00	2,00	2,00	2,00	2,00	2,00	2,00	#NULL!	#NULL!
54,00	1,00	2,00	2,00	2,00	2,00	1,00	15,00	#NULL!
45,00	1,00	2,00	2,00	2,00	2,00	1,00	10,00	#NULL!
58,00	2,00	2,00	2,00	1,00	2,00	2,00	#NULL!	#NULL!
68,00	2,00	1,00	2,00	1,00	2,00	2,00	#NULL!	#NULL!
73,00	1,00	1,00	2,00	2,00	2,00	2,00	#NULL!	#NULL!
45,00	2,00	2,00	2,00	2,00	2,00	2,00	#NULL!	#NULL!
63,00	2,00	2,00	1,00	1,00	1,00	2,00	#NULL!	#NULL!
48,00	1,00	2,00	2,00	2,00	2,00	1,00	10,00	#NULL!
63,00	1,00	1,00	2,00	2,00	1,00	2,00	#NULL!	#NULL!
54,00	2,00	1,00	2,00	2,00	2,00	2,00	#NULL!	#NULL!
61,00	2,00	1,00	2,00	2,00	2,00	2,00	#NULL!	#NULL!
45,00	1,00	2,00	2,00	2,00	2,00	2,00	#NULL!	#NULL!
48,00	1,00	2,00	2,00	2,00	2,00	1,00	10,00	#NULL!
64,00	2,00	1,00	2,00	2,00	2,00	2,00	#NULL!	#NULL!
49,00	2,00	2,00	2,00	2,00	2,00	2,00	#NULL!	#NULL!
54,00	2,00	1,00	2,00	2,00	2,00	2,00	#NULL!	#NULL!
48,00	2,00	2,00	2,00	2,00	2,00	2,00	#NULL!	#NULL!

61,00	2,00	1,00	2,00	2,00	2,00	2,00	#NULL!	#NULL!
59,00	1,00	1,00	2,00	2,00	2,00	2,00	#NULL!	#NULL!
59,00	1,00	1,00	1,00	2,00	2,00	2,00	#NULL!	#NULL!
62,00	1,00	2,00	2,00	1,00	2,00	2,00	#NULL!	#NULL!
79,00	2,00	2,00	2,00	1,00	1,00	2,00	#NULL!	#NULL!
45,00	1,00	2,00	2,00	2,00	2,00	1,00	20,00	#NULL!
51,00	2,00	2,00	1,00	2,00	1,00	2,00	#NULL!	#NULL!
51,00	2,00	1,00	2,00	2,00	2,00	2,00	#NULL!	#NULL!
59,00	1,00	2,00	1,00	2,00	1,00	2,00	#NULL!	#NULL!
55,00	1,00	2,00	1,00	2,00	1,00	2,00	#NULL!	#NULL!
66,00	2,00	1,00	2,00	2,00	2,00	2,00	#NULL!	#NULL!
68,00	2,00	1,00	2,00	1,00	2,00	2,00	#NULL!	#NULL!
48,00	1,00	1,00	2,00	2,00	2,00	2,00	#NULL!	#NULL!
65,00	1,00	2,00	2,00	2,00	2,00	2,00	#NULL!	#NULL!
65,00	1,00	1,00	2,00	1,00	2,00	2,00	15,00	10,00
87,00	2,00	1,00	1,00	2,00	2,00	1,00	#NULL!	#NULL!
52,00	2,00	1,00	2,00	2,00	2,00	2,00	#NULL!	#NULL!
76,00	2,00	1,00	1,00	2,00	2,00	2,00	#NULL!	#NULL!
67,00	2,00	1,00	1,00	2,00	2,00	2,00	#NULL!	#NULL!
55,00	2,00	2,00	2,00	2,00	2,00	2,00	#NULL!	#NULL!
73,00	2,00	1,00	2,00	2,00	2,00	2,00	#NULL!	#NULL!
72,00	2,00	2,00	2,00	2,00	2,00	2,00	#NULL!	#NULL!
78,00	1,00	2,00	2,00	2,00	2,00	2,00	#NULL!	#NULL!
45,00	2,00	2,00	2,00	2,00	2,00	2,00	#NULL!	#NULL!
50,00	1,00	2,00	2,00	2,00	2,00	2,00	#NULL!	#NULL!
73,00	2,00	2,00	2,00	1,00	2,00	2,00	#NULL!	#NULL!
72,00	1,00	2,00	2,00	1,00	2,00	2,00	#NULL!	#NULL!
80,00	2,00	1,00	2,00	2,00	2,00	2,00	#NULL!	#NULL!
86,00	2,00	1,00	1,00	1,00	2,00	2,00	#NULL!	#NULL!
59,00	1,00	2,00	2,00	2,00	2,00	2,00	#NULL!	#NULL!
55,00	2,00	2,00	2,00	2,00	2,00	2,00	#NULL!	#NULL!
45,00	2,00	2,00	2,00	2,00	2,00	2,00	#NULL!	#NULL!
45,00	1,00	2,00	2,00	2,00	2,00	2,00	#NULL!	#NULL!
45,00	1,00	2,00	2,00	2,00	2,00	2,00	#NULL!	#NULL!
46,00	1,00	2,00	2,00	2,00	2,00	2,00	#NULL!	#NULL!
49,00	2,00	2,00	2,00	2,00	2,00	2,00	#NULL!	#NULL!
45,00	1,00	2,00	2,00	2,00	2,00	2,00	#NULL!	#NULL!
60,00	2,00	2,00	2,00	2,00	2,00	2,00	#NULL!	#NULL!
46,00	1,00	1,00	2,00	2,00	2,00	2,00	#NULL!	#NULL!
49,00	1,00	2,00	2,00	2,00	2,00	2,00	#NULL!	#NULL!
45,00	1,00	2,00	2,00	2,00	2,00	2,00	#NULL!	#NULL!
60,00	1,00	2,00	2,00	2,00	2,00	1,00	30,00	#NULL!
56,00	2,00	1,00	2,00	2,00	2,00	2,00	#NULL!	#NULL!
64,00	2,00	1,00	2,00	2,00	2,00	1,00	10,00	#NULL!
66,00	2,00	1,00	2,00	2,00	1,00	2,00	#NULL!	#NULL!
66,00	1,00	2,00	2,00	2,00	2,00	2,00	#NULL!	#NULL!
71,00	2,00	1,00	2,00	1,00	2,00	1,00	50,00	#NULL!
48,00	2,00	1,00	2,00	2,00	2,00	2,00	#NULL!	#NULL!
54,00	1,00	1,00	2,00	1,00	2,00	1,00	30,00	#NULL!
54,00	1,00	1,00	2,00	2,00	2,00	2,00	#NULL!	#NULL!

52,00	2,00	2,00	2,00	2,00	2,00	2,00	#NULL!	#NULL!
47,00	2,00	1,00	2,00	2,00	2,00	2,00	#NULL!	#NULL!
52,00	2,00	2,00	2,00	2,00	2,00	2,00	#NULL!	#NULL!
54,00	2,00	1,00	1,00	2,00	1,00	2,00	#NULL!	#NULL!
56,00	1,00	2,00	2,00	2,00	2,00	2,00	#NULL!	#NULL!
45,00	2,00	1,00	2,00	2,00	2,00	2,00	#NULL!	#NULL!
48,00	2,00	2,00	2,00	2,00	2,00	2,00	#NULL!	#NULL!
57,00	2,00	1,00	1,00	1,00	1,00	2,00	#NULL!	#NULL!
70,00	1,00	1,00	1,00	2,00	2,00	2,00	#NULL!	#NULL!
67,00	2,00	1,00	2,00	2,00	2,00	2,00	#NULL!	#NULL!
69,00	2,00	1,00	2,00	2,00	2,00	2,00	#NULL!	#NULL!
65,00	1,00	2,00	2,00	2,00	2,00	2,00	#NULL!	#NULL!
61,00	2,00	2,00	2,00	2,00	2,00	2,00	#NULL!	#NULL!
61,00	2,00	1,00	2,00	2,00	1,00	2,00	#NULL!	#NULL!
70,00	2,00	1,00	2,00	2,00	2,00	2,00	#NULL!	#NULL!
79,00	1,00	1,00	2,00	1,00	2,00	2,00	#NULL!	#NULL!
76,00	1,00	2,00	2,00	2,00	2,00	2,00	#NULL!	#NULL!
58,00	2,00	1,00	2,00	1,00	2,00	2,00	#NULL!	#NULL!
65,00	1,00	1,00	2,00	1,00	2,00	2,00	#NULL!	#NULL!
54,00	2,00	1,00	2,00	2,00	2,00	2,00	#NULL!	#NULL!
69,00	1,00	1,00	2,00	2,00	2,00	2,00	#NULL!	#NULL!
60,00	2,00	1,00	2,00	2,00	2,00	2,00	#NULL!	#NULL!
45,00	1,00	2,00	2,00	2,00	2,00	2,00	#NULL!	#NULL!
65,00	2,00	2,00	2,00	2,00	2,00	2,00	#NULL!	#NULL!
67,00	1,00	1,00	2,00	2,00	2,00	2,00	#NULL!	#NULL!
91,00	1,00	1,00	2,00	1,00	2,00	2,00	#NULL!	#NULL!
53,00	1,00	2,00	2,00	2,00	2,00	2,00	#NULL!	#NULL!
65,00	1,00	1,00	1,00	2,00	2,00	2,00	#NULL!	#NULL!
68,00	1,00	2,00	1,00	2,00	1,00	2,00	#NULL!	#NULL!
47,00	2,00	2,00	2,00	2,00	2,00	2,00	#NULL!	#NULL!
54,00	1,00	2,00	2,00	2,00	2,00	2,00	#NULL!	#NULL!
62,00	1,00	2,00	2,00	2,00	2,00	2,00	#NULL!	#NULL!
57,00	2,00	2,00	2,00	2,00	2,00	2,00	#NULL!	#NULL!
53,00	1,00	2,00	2,00	2,00	2,00	1,00	15,00	#NULL!
48,00	2,00	2,00	2,00	2,00	2,00	2,00	#NULL!	#NULL!
52,00	1,00	2,00	2,00	2,00	2,00	2,00	#NULL!	#NULL!
48,00	2,00	2,00	2,00	2,00	2,00	2,00	#NULL!	#NULL!
56,00	1,00	2,00	2,00	2,00	2,00	2,00	#NULL!	#NULL!
52,00	2,00	2,00	2,00	2,00	2,00	2,00	#NULL!	#NULL!
61,00	1,00	2,00	2,00	2,00	2,00	2,00	#NULL!	#NULL!
56,00	2,00	2,00	2,00	2,00	2,00	2,00	#NULL!	#NULL!
62,00	1,00	2,00	2,00	2,00	2,00	2,00	#NULL!	#NULL!
56,00	2,00	2,00	2,00	2,00	2,00	2,00	#NULL!	#NULL!
56,00	1,00	2,00	2,00	2,00	2,00	1,00	30,00	#NULL!
54,00	2,00	2,00	2,00	2,00	2,00	2,00	#NULL!	#NULL!
51,00	1,00	2,00	2,00	2,00	2,00	2,00	#NULL!	#NULL!
45,00	2,00	2,00	2,00	2,00	2,00	2,00	#NULL!	#NULL!
55,00	1,00	2,00	2,00	2,00	2,00	2,00	#NULL!	#NULL!
53,00	2,00	2,00	2,00	2,00	2,00	2,00	#NULL!	#NULL!
50,00	2,00	2,00	2,00	2,00	2,00	2,00	#NULL!	#NULL!

55,00	1,00	2,00	2,00	2,00	2,00	1,00	30,00	#NULL!
60,00	1,00	2,00	2,00	2,00	2,00	2,00	#NULL!	#NULL!
65,00	1,00	2,00	2,00	2,00	2,00	2,00	#NULL!	#NULL!
85,00	2,00	2,00	2,00	2,00	2,00	2,00	#NULL!	#NULL!
62,00	1,00	2,00	2,00	2,00	2,00	2,00	#NULL!	#NULL!
50,00	1,00	2,00	2,00	2,00	2,00	2,00	#NULL!	#NULL!
45,00	2,00	2,00	2,00	2,00	2,00	2,00	#NULL!	#NULL!
55,00	2,00	2,00	2,00	2,00	2,00	1,00	15,00	#NULL!
62,00	1,00	2,00	2,00	2,00	2,00	2,00	#NULL!	#NULL!
65,00	1,00	2,00	2,00	2,00	2,00	2,00	#NULL!	#NULL!
60,00	1,00	2,00	2,00	2,00	2,00	2,00	#NULL!	#NULL!
70,00	2,00	2,00	2,00	2,00	2,00	2,00	#NULL!	#NULL!
62,00	1,00	1,00	2,00	2,00	2,00	1,00	18,00	#NULL!
71,00	1,00	2,00	2,00	2,00	2,00	1,00	50,00	#NULL!
78,00	1,00	2,00	2,00	2,00	2,00	2,00	#NULL!	#NULL!
58,00	1,00	1,00	1,00	2,00	2,00	2,00	20,00	3,00
54,00	2,00	1,00	2,00	2,00	1,00	2,00	#NULL!	#NULL!
46,00	2,00	2,00	2,00	2,00	2,00	2,00	#NULL!	#NULL!
49,00	2,00	1,00	2,00	2,00	2,00	2,00	#NULL!	#NULL!
55,00	1,00	2,00	2,00	1,00	2,00	2,00	#NULL!	#NULL!
50,00	2,00	2,00	2,00	2,00	2,00	2,00	#NULL!	#NULL!
51,00	1,00	2,00	2,00	1,00	2,00	2,00	#NULL!	#NULL!
55,00	1,00	2,00	2,00	1,00	2,00	2,00	#NULL!	#NULL!
49,00	1,00	1,00	2,00	2,00	1,00	1,00	11,00	#NULL!
45,00	2,00	2,00	2,00	2,00	2,00	2,00	#NULL!	#NULL!
62,00	1,00	1,00	2,00	2,00	2,00	2,00	#NULL!	#NULL!
58,00	2,00	2,00	2,00	2,00	2,00	2,00	#NULL!	#NULL!
57,00	2,00	2,00	1,00	2,00	2,00	2,00	#NULL!	#NULL!
69,00	1,00	1,00	2,00	2,00	2,00	2,00	#NULL!	#NULL!
55,00	1,00	2,00	2,00	2,00	2,00	2,00	#NULL!	#NULL!
56,00	2,00	2,00	2,00	2,00	2,00	2,00	#NULL!	#NULL!
79,00	1,00	2,00	2,00	2,00	2,00	2,00	#NULL!	#NULL!
72,00	2,00	1,00	2,00	2,00	2,00	2,00	#NULL!	#NULL!
86,00	1,00	1,00	1,00	2,00	2,00	2,00	#NULL!	#NULL!
76,00	2,00	1,00	2,00	2,00	2,00	2,00	#NULL!	#NULL!
58,00	2,00	1,00	2,00	2,00	2,00	2,00	#NULL!	#NULL!
65,00	1,00	1,00	1,00	2,00	2,00	2,00	#NULL!	#NULL!
76,00	2,00	1,00	1,00	2,00	2,00	2,00	#NULL!	#NULL!
60,00	1,00	2,00	2,00	2,00	2,00	2,00	#NULL!	#NULL!
64,00	1,00	1,00	2,00	2,00	2,00	1,00	20,00	#NULL!
45,00	2,00	2,00	2,00	2,00	2,00	1,00	10,00	#NULL!
62,00	2,00	2,00	1,00	2,00	1,00	2,00	#NULL!	#NULL!
59,00	1,00	1,00	2,00	2,00	1,00	1,00	20,00	#NULL!
62,00	1,00	1,00	2,00	1,00	2,00	2,00	#NULL!	#NULL!
59,00	2,00	1,00	2,00	1,00	2,00	2,00	#NULL!	#NULL!
59,00	2,00	1,00	2,00	2,00	2,00	2,00	#NULL!	#NULL!
60,00	2,00	1,00	2,00	2,00	2,00	2,00	#NULL!	#NULL!
69,00	2,00	1,00	2,00	1,00	2,00	1,00	15,00	#NULL!
64,00	1,00	1,00	2,00	2,00	2,00	2,00	#NULL!	#NULL!
45,00	2,00	2,00	2,00	2,00	2,00	2,00	#NULL!	#NULL!

55,00	1,00	1,00	2,00	2,00	2,00	2,00	#NULL!	#NULL!
47,00	2,00	2,00	2,00	2,00	2,00	2,00	#NULL!	#NULL!
55,00	1,00	1,00	2,00	2,00	2,00	2,00	#NULL!	#NULL!
74,00	2,00	2,00	1,00	1,00	2,00	2,00	#NULL!	#NULL!
62,00	1,00	1,00	2,00	2,00	2,00	2,00	#NULL!	#NULL!
60,00	2,00	2,00	2,00	1,00	2,00	2,00	#NULL!	#NULL!
58,00	2,00	1,00	2,00	1,00	2,00	2,00	#NULL!	#NULL!
52,00	2,00	2,00	2,00	2,00	1,00	2,00	#NULL!	#NULL!
45,00	1,00	2,00	2,00	1,00	2,00	2,00	#NULL!	#NULL!
61,00	2,00	1,00	2,00	2,00	2,00	2,00	#NULL!	#NULL!
64,00	2,00	2,00	1,00	2,00	2,00	2,00	#NULL!	#NULL!
55,00	2,00	1,00	2,00	1,00	2,00	2,00	#NULL!	#NULL!
46,00	1,00	2,00	2,00	2,00	2,00	1,00	20,00	#NULL!
57,00	1,00	1,00	2,00	2,00	2,00	2,00	#NULL!	#NULL!
53,00	1,00	2,00	2,00	1,00	2,00	1,00	30,00	#NULL!
46,00	2,00	2,00	2,00	2,00	2,00	2,00	#NULL!	#NULL!
52,00	1,00	2,00	2,00	2,00	2,00	1,00	7,00	#NULL!
60,00	1,00	2,00	2,00	1,00	2,00	1,00	80,00	#NULL!
45,00	2,00	2,00	2,00	2,00	2,00	2,00	#NULL!	#NULL!
52,00	1,00	1,00	2,00	2,00	2,00	2,00	#NULL!	#NULL!
51,00	2,00	2,00	2,00	2,00	2,00	2,00	#NULL!	#NULL!
55,00	1,00	2,00	2,00	1,00	2,00	2,00	#NULL!	#NULL!
70,00	2,00	1,00	2,00	2,00	2,00	2,00	#NULL!	#NULL!
51,00	2,00	2,00	2,00	2,00	2,00	2,00	#NULL!	#NULL!
51,00	1,00	2,00	2,00	2,00	2,00	2,00	#NULL!	#NULL!
48,00	1,00	2,00	2,00	2,00	2,00	1,00	5,00	#NULL!
82,00	1,00	1,00	2,00	1,00	1,00	2,00	#NULL!	#NULL!
52,00	2,00	1,00	2,00	2,00	2,00	1,00	3,00	#NULL!
49,00	1,00	2,00	2,00	1,00	2,00	1,00	15,00	#NULL!
48,00	2,00	2,00	2,00	2,00	2,00	2,00	#NULL!	#NULL!
63,00	1,00	1,00	2,00	2,00	2,00	1,00	40,00	#NULL!
52,00	1,00	2,00	2,00	2,00	2,00	2,00	#NULL!	#NULL!
62,00	1,00	1,00	2,00	2,00	2,00	2,00	#NULL!	#NULL!
72,00	1,00	1,00	1,00	2,00	2,00	2,00	#NULL!	#NULL!
69,00	2,00	1,00	2,00	2,00	1,00	2,00	#NULL!	#NULL!
49,00	1,00	1,00	1,00	2,00	2,00	2,00	#NULL!	#NULL!
46,00	2,00	1,00	1,00	2,00	2,00	2,00	#NULL!	#NULL!
52,00	1,00	1,00	2,00	2,00	2,00	2,00	#NULL!	#NULL!
53,00	1,00	2,00	1,00	2,00	2,00	2,00	#NULL!	#NULL!
45,00	2,00	2,00	2,00	2,00	2,00	2,00	#NULL!	#NULL!
47,00	2,00	2,00	2,00	2,00	1,00	2,00	#NULL!	#NULL!
58,00	1,00	2,00	2,00	2,00	2,00	2,00	#NULL!	#NULL!
80,00	1,00	1,00	2,00	1,00	1,00	1,00	50,00	#NULL!
45,00	2,00	2,00	2,00	2,00	2,00	2,00	#NULL!	#NULL!
77,00	2,00	1,00	2,00	2,00	2,00	2,00	#NULL!	#NULL!
51,00	2,00	1,00	2,00	2,00	2,00	2,00	#NULL!	#NULL!
78,00	2,00	1,00	1,00	2,00	2,00	2,00	#NULL!	#NULL!
53,00	1,00	2,00	2,00	2,00	2,00	2,00	#NULL!	#NULL!
50,00	1,00	2,00	2,00	2,00	2,00	2,00	#NULL!	#NULL!
58,00	1,00	2,00	2,00	2,00	2,00	2,00	#NULL!	#NULL!

49,00	2,00	1,00	2,00	2,00	2,00	2,00	#NULL!	#NULL!
47,00	1,00	2,00	2,00	2,00	2,00	2,00	#NULL!	#NULL!
51,00	2,00	2,00	2,00	2,00	1,00	2,00	#NULL!	#NULL!
58,00	1,00	2,00	2,00	1,00	2,00	1,00	15,00	#NULL!
46,00	2,00	2,00	2,00	2,00	2,00	2,00	#NULL!	#NULL!
45,00	1,00	2,00	22,00	2,00	2,00	1,00	10,00	#NULL!
55,00	2,00	1,00	2,00	2,00	2,00	2,00	#NULL!	#NULL!
45,00	1,00	2,00	2,00	2,00	2,00	2,00	#NULL!	#NULL!
59,00	1,00	1,00	2,00	2,00	1,00	2,00	#NULL!	#NULL!
50,00	2,00	1,00	2,00	2,00	2,00	2,00	#NULL!	#NULL!
57,00	1,00	1,00	2,00	1,00	2,00	1,00	80,00	#NULL!
49,00	2,00	2,00	2,00	2,00	2,00	2,00	#NULL!	#NULL!
59,00	1,00	2,00	2,00	1,00	2,00	1,00	15,00	#NULL!
72,00	2,00	2,00	2,00	1,00	2,00	2,00	#NULL!	#NULL!
45,00	2,00	2,00	2,00	2,00	2,00	2,00	#NULL!	#NULL!
85,00	2,00	1,00	2,00	2,00	2,00	2,00	#NULL!	#NULL!
52,00	1,00	2,00	2,00	2,00	2,00	1,00	8,00	#NULL!
45,00	2,00	2,00	2,00	2,00	2,00	2,00	#NULL!	#NULL!
58,00	1,00	1,00	2,00	2,00	2,00	1,00	15,00	#NULL!
78,00	1,00	1,00	2,00	1,00	2,00	2,00	#NULL!	#NULL!
48,00	2,00	2,00	2,00	2,00	2,00	2,00	#NULL!	#NULL!
78,00	1,00	1,00	2,00	2,00	2,00	2,00	#NULL!	#NULL!
79,00	2,00	1,00	1,00	2,00	2,00	2,00	#NULL!	#NULL!
49,00	1,00	2,00	2,00	2,00	2,00	1,00	30,00	#NULL!
73,00	2,00	1,00	2,00	1,00	2,00	2,00	#NULL!	#NULL!
45,00	2,00	2,00	2,00	2,00	2,00	2,00	#NULL!	#NULL!
46,00	1,00	2,00	2,00	2,00	2,00	1,00	6,00	#NULL!
45,00	2,00	2,00	2,00	2,00	2,00	2,00	#NULL!	#NULL!
51,00	2,00	2,00	2,00	2,00	2,00	2,00	#NULL!	#NULL!
76,00	2,00	1,00	2,00	1,00	2,00	2,00	#NULL!	#NULL!
46,00	2,00	2,00	2,00	2,00	2,00	2,00	#NULL!	#NULL!
54,00	1,00	2,00	2,00	2,00	2,00	2,00	#NULL!	#NULL!
56,00	1,00	1,00	2,00	2,00	2,00	2,00	#NULL!	#NULL!
46,00	2,00	2,00	2,00	2,00	2,00	1,00	4,00	#NULL!
47,00	2,00	2,00	2,00	2,00	2,00	2,00	#NULL!	#NULL!
75,00	2,00	1,00	1,00	2,00	2,00	2,00	#NULL!	#NULL!
78,00	1,00	1,00	2,00	1,00	1,00	1,00	40,00	#NULL!
59,00	1,00	1,00	2,00	1,00	2,00	2,00	#NULL!	#NULL!
52,00	2,00	2,00	1,00	2,00	2,00	2,00	#NULL!	#NULL!
49,00	1,00	2,00	2,00	2,00	2,00	1,00	25,00	#NULL!
54,00	1,00	1,00	2,00	2,00	2,00	2,00	#NULL!	#NULL!
45,00	2,00	2,00	2,00	2,00	2,00	1,00	15,00	#NULL!
55,00	2,00	1,00	2,00	2,00	2,00	2,00	#NULL!	#NULL!
82,00	1,00	1,00	1,00	2,00	2,00	2,00	#NULL!	#NULL!
67,00	2,00	1,00	1,00	2,00	1,00	2,00	#NULL!	#NULL!
47,00	1,00	2,00	2,00	2,00	2,00	2,00	#NULL!	#NULL!
80,00	1,00	1,00	1,00	1,00	1,00	1,00	80,00	#NULL!
49,00	2,00	2,00	2,00	2,00	2,00	2,00	#NULL!	#NULL!
52,00	1,00	2,00	2,00	2,00	2,00	2,00	#NULL!	#NULL!
80,00	1,00	1,00	2,00	1,00	1,00	1,00	10,00	#NULL!

47,00	2,00	2,00	2,00	2,00	2,00	2,00	#NULL!	#NULL!
64,00	2,00	1,00	1,00	2,00	2,00	2,00	#NULL!	#NULL!
66,00	2,00	1,00	2,00	2,00	2,00	2,00	#NULL!	#NULL!
65,00	1,00	2,00	2,00	2,00	2,00	2,00	#NULL!	#NULL!
63,00	2,00	1,00	1,00	1,00	1,00	2,00	#NULL!	#NULL!
46,00	1,00	2,00	2,00	2,00	2,00	2,00	#NULL!	#NULL!
45,00	2,00	2,00	2,00	2,00	2,00	2,00	#NULL!	#NULL!
46,00	1,00	1,00	2,00	2,00	1,00	1,00	30,00	#NULL!
45,00	1,00	2,00	2,00	2,00	2,00	2,00	#NULL!	#NULL!
46,00	2,00	2,00	2,00	2,00	2,00	2,00	#NULL!	#NULL!
49,00	1,00	2,00	2,00	2,00	2,00	2,00	#NULL!	#NULL!
50,00	1,00	1,00	2,00	2,00	2,00	2,00	#NULL!	#NULL!
49,00	2,00	2,00	2,00	2,00	2,00	2,00	#NULL!	#NULL!
70,00	1,00	2,00	2,00	2,00	1,00	1,00	7,00	#NULL!
75,00	1,00	2,00	2,00	2,00	1,00	2,00	#NULL!	#NULL!
55,00	1,00	2,00	2,00	2,00	2,00	2,00	#NULL!	#NULL!
50,00	2,00	2,00	2,00	2,00	2,00	2,00	#NULL!	#NULL!
48,00	1,00	2,00	2,00	2,00	2,00	2,00	#NULL!	#NULL!
45,00	2,00	2,00	2,00	2,00	2,00	2,00	#NULL!	#NULL!
45,00	1,00	2,00	2,00	2,00	2,00	2,00	#NULL!	#NULL!
52,00	2,00	2,00	2,00	2,00	2,00	2,00	#NULL!	#NULL!
50,00	2,00	1,00	2,00	2,00	1,00	2,00	#NULL!	#NULL!
48,00	1,00	2,00	2,00	2,00	2,00	2,00	#NULL!	#NULL!
45,00	2,00	2,00	2,00	2,00	2,00	2,00	#NULL!	#NULL!
49,00	1,00	2,00	2,00	2,00	2,00	2,00	#NULL!	#NULL!
48,00	2,00	2,00	2,00	2,00	2,00	2,00	#NULL!	#NULL!
58,00	1,00	1,00	2,00	2,00	2,00	2,00	#NULL!	#NULL!
45,00	2,00	2,00	2,00	2,00	2,00	2,00	#NULL!	#NULL!
55,00	2,00	2,00	2,00	2,00	2,00	2,00	#NULL!	#NULL!
72,00	2,00	1,00	1,00	2,00	2,00	2,00	#NULL!	#NULL!
75,00	1,00	1,00	2,00	2,00	2,00	2,00	#NULL!	#NULL!
51,00	1,00	2,00	2,00	2,00	2,00	2,00	#NULL!	#NULL!
46,00	2,00	2,00	2,00	2,00	2,00	2,00	#NULL!	#NULL!
52,00	1,00	2,00	2,00	2,00	2,00	2,00	#NULL!	#NULL!
55,00	2,00	1,00	1,00	1,00	2,00	2,00	#NULL!	#NULL!
56,00	1,00	2,00	2,00	2,00	2,00	2,00	#NULL!	#NULL!
54,00	2,00	2,00	2,00	1,00	2,00	2,00	#NULL!	#NULL!
53,00	1,00	2,00	2,00	2,00	2,00	2,00	#NULL!	#NULL!
51,00	2,00	2,00	2,00	2,00	2,00	2,00	#NULL!	#NULL!
58,00	1,00	2,00	2,00	2,00	2,00	2,00	#NULL!	#NULL!
56,00	2,00	2,00	1,00	2,00	2,00	2,00	#NULL!	#NULL!
54,00	1,00	2,00	2,00	2,00	2,00	2,00	#NULL!	#NULL!
52,00	2,00	1,00	2,00	2,00	2,00	2,00	#NULL!	#NULL!
64,00	1,00	1,00	1,00	1,00	1,00	2,00	#NULL!	#NULL!
61,00	2,00	1,00	2,00	2,00	1,00	2,00	#NULL!	#NULL!
56,00	1,00	1,00	1,00	2,00	1,00	2,00	#NULL!	#NULL!
51,00	2,00	1,00	1,00	2,00	2,00	2,00	#NULL!	#NULL!
50,00	1,00	2,00	2,00	2,00	2,00	2,00	#NULL!	#NULL!
47,00	2,00	1,00	2,00	2,00	1,00	2,00	#NULL!	#NULL!
56,00	1,00	1,00	1,00	1,00	1,00	2,00	#NULL!	#NULL!

53,00	2,00	2,00	2,00	2,00	2,00	2,00	#NULL!	#NULL!
50,00	1,00	2,00	2,00	2,00	2,00	2,00	#NULL!	#NULL!
47,00	2,00	1,00	2,00	2,00	1,00	2,00	#NULL!	#NULL!
72,00	1,00	1,00	2,00	2,00	2,00	2,00	#NULL!	#NULL!
53,00	1,00	1,00	2,00	2,00	1,00	2,00	#NULL!	#NULL!
60,00	1,00	1,00	2,00	2,00	2,00	2,00	10,00	6,00
63,00	1,00	1,00	2,00	2,00	2,00	2,00	#NULL!	#NULL!
60,00	1,00	2,00	2,00	2,00	2,00	1,00	30,00	#NULL!
55,00	2,00	1,00	2,00	1,00	1,00	2,00	#NULL!	#NULL!
53,00	1,00	2,00	2,00	2,00	2,00	1,00	10,00	#NULL!
53,00	2,00	2,00	2,00	2,00	2,00	2,00	#NULL!	#NULL!
59,00	1,00	1,00	1,00	2,00	1,00	2,00	#NULL!	#NULL!
54,00	2,00	1,00	1,00	2,00	2,00	2,00	#NULL!	#NULL!
50,00	1,00	2,00	2,00	2,00	2,00	2,00	#NULL!	#NULL!
54,00	1,00	2,00	2,00	2,00	2,00	2,00	#NULL!	#NULL!
62,00	1,00	2,00	2,00	2,00	2,00	2,00	#NULL!	#NULL!
56,00	2,00	2,00	2,00	2,00	2,00	2,00	#NULL!	#NULL!
80,00	1,00	1,00	2,00	2,00	2,00	2,00	#NULL!	#NULL!
45,00	2,00	2,00	2,00	2,00	2,00	2,00	#NULL!	#NULL!
55,00	1,00	1,00	2,00	2,00	2,00	2,00	#NULL!	#NULL!
54,00	1,00	1,00	2,00	2,00	1,00	2,00	#NULL!	#NULL!
76,00	1,00	2,00	2,00	2,00	2,00	2,00	#NULL!	#NULL!
73,00	2,00	1,00	2,00	2,00	1,00	2,00	#NULL!	#NULL!
80,00	2,00	1,00	2,00	2,00	2,00	2,00	#NULL!	#NULL!
54,00	1,00	2,00	2,00	2,00	2,00	2,00	#NULL!	#NULL!
48,00	1,00	2,00	2,00	2,00	2,00	2,00	#NULL!	#NULL!
60,00	1,00	2,00	2,00	2,00	2,00	2,00	#NULL!	#NULL!
47,00	1,00	2,00	2,00	2,00	2,00	2,00	#NULL!	#NULL!
73,00	1,00	2,00	2,00	2,00	2,00	2,00	#NULL!	#NULL!
70,00	1,00	2,00	2,00	2,00	2,00	2,00	#NULL!	#NULL!
65,00	2,00	2,00	2,00	2,00	2,00	2,00	#NULL!	#NULL!
50,00	1,00	2,00	2,00	2,00	2,00	2,00	#NULL!	#NULL!
45,00	2,00	1,00	2,00	2,00	2,00	2,00	#NULL!	#NULL!
51,00	2,00	1,00	2,00	2,00	1,00	2,00	#NULL!	#NULL!
56,00	2,00	2,00	2,00	2,00	2,00	2,00	#NULL!	#NULL!
70,00	1,00	1,00	2,00	2,00	2,00	1,00	25,00	#NULL!
63,00	1,00	1,00	2,00	2,00	2,00	2,00	#NULL!	#NULL!
68,00	1,00	1,00	1,00	2,00	2,00	2,00	40,00	5,00
66,00	2,00	2,00	2,00	2,00	2,00	2,00	#NULL!	#NULL!
46,00	1,00	2,00	2,00	2,00	2,00	2,00	#NULL!	#NULL!
66,00	1,00	1,00	2,00	1,00	1,00	2,00	#NULL!	#NULL!
52,00	2,00	2,00	2,00	2,00	2,00	2,00	#NULL!	#NULL!
46,00	1,00	2,00	2,00	1,00	2,00	2,00	#NULL!	#NULL!
41,00	2,00	2,00	2,00	2,00	2,00	1,00	10,00	#NULL!
56,00	1,00	1,00	1,00	2,00	2,00	2,00	#NULL!	#NULL!
65,00	2,00	1,00	2,00	1,00	2,00	2,00	#NULL!	#NULL!
76,00	1,00	1,00	2,00	2,00	2,00	2,00	#NULL!	#NULL!
50,00	2,00	2,00	2,00	2,00	2,00	1,00	60,00	#NULL!
56,00	1,00	2,00	2,00	2,00	2,00	1,00	25,00	#NULL!
60,00	2,00	1,00	1,00	1,00	1,00	2,00	#NULL!	#NULL!

66,00	1,00	1,00	2,00	1,00	1,00	2,00	#NULL!	#NULL!
52,00	2,00	2,00	2,00	2,00	2,00	2,00	#NULL!	#NULL!
57,00	1,00	2,00	1,00	2,00	2,00	1,00	20,00	#NULL!
66,00	2,00	1,00	2,00	2,00	2,00	2,00	80,00	13,00
66,00	1,00	1,00	2,00	2,00	2,00	2,00	60,00	10,00
50,00	2,00	2,00	2,00	2,00	2,00	2,00	#NULL!	#NULL!
57,00	1,00	2,00	2,00	1,00	2,00	1,00	45,00	#NULL!
65,00	2,00	1,00	2,00	1,00	2,00	2,00	#NULL!	#NULL!
70,00	1,00	2,00	2,00	2,00	2,00	2,00	#NULL!	#NULL!
75,00	2,00	2,00	2,00	2,00	2,00	2,00	#NULL!	#NULL!
80,00	1,00	1,00	2,00	2,00	1,00	2,00	#NULL!	#NULL!
60,00	2,00	1,00	1,00	2,00	1,00	2,00	#NULL!	#NULL!
60,00	1,00	1,00	2,00	2,00	1,00	1,00	75,00	#NULL!
56,00	1,00	1,00	2,00	2,00	2,00	2,00	#NULL!	#NULL!
46,00	1,00	1,00	2,00	2,00	2,00	2,00	#NULL!	#NULL!
58,00	2,00	1,00	2,00	2,00	2,00	2,00	#NULL!	#NULL!
66,00	1,00	1,00	1,00	2,00	2,00	2,00	#NULL!	#NULL!
60,00	2,00	2,00	2,00	2,00	2,00	2,00	#NULL!	#NULL!
68,00	1,00	1,00	2,00	2,00	2,00	2,00	#NULL!	#NULL!
57,00	2,00	1,00	2,00	2,00	2,00	2,00	#NULL!	#NULL!
65,00	1,00	1,00	2,00	2,00	2,00	2,00	#NULL!	#NULL!
51,00	2,00	1,00	2,00	1,00	2,00	1,00	3,00	#NULL!
56,00	1,00	1,00	2,00	2,00	2,00	2,00	#NULL!	#NULL!
76,00	1,00	1,00	1,00	1,00	2,00	2,00	#NULL!	#NULL!
80,00	1,00	1,00	2,00	1,00	2,00	2,00	#NULL!	#NULL!
62,00	1,00	1,00	2,00	2,00	2,00	2,00	#NULL!	#NULL!
60,00	2,00	2,00	2,00	2,00	2,00	2,00	#NULL!	#NULL!
56,00	1,00	2,00	2,00	2,00	2,00	2,00	#NULL!	#NULL!
58,00	1,00	2,00	2,00	2,00	2,00	2,00	#NULL!	#NULL!
49,00	2,00	2,00	2,00	2,00	2,00	2,00	#NULL!	#NULL!
56,00	2,00	2,00	2,00	2,00	2,00	2,00	#NULL!	#NULL!
45,00	1,00	2,00	2,00	2,00	2,00	2,00	#NULL!	#NULL!
48,00	1,00	2,00	2,00	2,00	2,00	2,00	#NULL!	#NULL!
48,00	1,00	2,00	2,00	2,00	2,00	2,00	#NULL!	#NULL!
45,00	1,00	2,00	2,00	2,00	2,00	2,00	#NULL!	#NULL!
55,00	1,00	2,00	2,00	2,00	2,00	1,00	10,00	#NULL!
59,00	2,00	2,00	2,00	2,00	2,00	2,00	#NULL!	#NULL!
60,00	1,00	2,00	2,00	2,00	2,00	2,00	#NULL!	#NULL!
58,00	2,00	2,00	2,00	2,00	2,00	2,00	#NULL!	#NULL!
46,00	2,00	2,00	2,00	2,00	2,00	2,00	#NULL!	#NULL!
59,00	2,00	2,00	2,00	2,00	2,00	2,00	#NULL!	#NULL!
60,00	1,00	2,00	2,00	2,00	2,00	2,00	#NULL!	#NULL!
58,00	2,00	2,00	2,00	2,00	2,00	2,00	#NULL!	#NULL!
72,00	2,00	2,00	2,00	1,00	1,00	2,00	#NULL!	#NULL!
75,00	1,00	2,00	1,00	2,00	2,00	2,00	#NULL!	#NULL!
58,00	2,00	1,00	2,00	2,00	2,00	2,00	#NULL!	#NULL!
69,00	1,00	2,00	2,00	2,00	2,00	2,00	#NULL!	#NULL!
72,00	1,00	1,00	1,00	2,00	2,00	2,00	#NULL!	#NULL!
68,00	2,00	1,00	1,00	2,00	2,00	2,00	#NULL!	#NULL!
59,00	2,00	2,00	2,00	2,00	2,00	2,00	#NULL!	#NULL!

65,00	1,00	2,00	1,00	2,00	2,00	2,00	#NULL!	#NULL!
70,00	1,00	2,00	2,00	2,00	2,00	2,00	#NULL!	#NULL!
56,00	1,00	2,00	2,00	2,00	2,00	2,00	#NULL!	#NULL!
52,00	1,00	1,00	1,00	2,00	2,00	2,00	#NULL!	#NULL!
70,00	2,00	2,00	2,00	2,00	2,00	2,00	#NULL!	#NULL!
79,00	1,00	1,00	1,00	2,00	2,00	2,00	30,00	30,00
70,00	2,00	2,00	2,00	1,00	1,00	2,00	#NULL!	#NULL!
49,00	1,00	1,00	2,00	2,00	1,00	1,00	20,00	#NULL!
46,00	2,00	2,00	2,00	2,00	2,00	2,00	#NULL!	#NULL!
48,00	1,00	1,00	2,00	2,00	2,00	1,00	20,00	#NULL!
50,00	1,00	2,00	2,00	1,00	1,00	2,00	#NULL!	#NULL!
85,00	2,00	1,00	2,00	2,00	1,00	2,00	#NULL!	#NULL!
70,00	1,00	1,00	2,00	2,00	2,00	2,00	#NULL!	#NULL!
57,00	1,00	2,00	2,00	2,00	2,00	2,00	20,00	20,00
53,00	2,00	2,00	2,00	2,00	2,00	2,00	#NULL!	#NULL!
76,00	2,00	1,00	1,00	2,00	1,00	2,00	#NULL!	#NULL!
78,00	1,00	1,00	2,00	1,00	1,00	2,00	#NULL!	#NULL!
66,00	2,00	1,00	2,00	2,00	2,00	2,00	#NULL!	#NULL!
69,00	1,00	1,00	1,00	1,00	1,00	1,00	60,00	#NULL!
48,00	2,00	2,00	2,00	2,00	2,00	2,00	#NULL!	#NULL!
57,00	1,00	1,00	2,00	2,00	2,00	2,00	#NULL!	#NULL!
59,00	2,00	2,00	2,00	2,00	2,00	2,00	#NULL!	#NULL!
62,00	1,00	2,00	2,00	2,00	2,00	2,00	#NULL!	#NULL!
48,00	2,00	2,00	1,00	2,00	2,00	2,00	#NULL!	#NULL!
55,00	1,00	1,00	2,00	2,00	1,00	2,00	#NULL!	#NULL!
58,00	1,00	2,00	2,00	2,00	2,00	2,00	#NULL!	#NULL!
60,00	2,00	2,00	2,00	2,00	2,00	2,00	#NULL!	#NULL!
58,00	2,00	1,00	1,00	1,00	2,00	2,00	#NULL!	#NULL!
48,00	2,00	2,00	2,00	2,00	2,00	2,00	#NULL!	#NULL!
62,00	2,00	1,00	2,00	2,00	2,00	2,00	#NULL!	#NULL!
65,00	2,00	1,00	2,00	2,00	1,00	2,00	#NULL!	#NULL!
56,00	2,00	2,00	1,00	2,00	2,00	1,00	30,00	#NULL!
62,00	1,00	2,00	1,00	2,00	2,00	2,00	#NULL!	#NULL!
57,00	1,00	1,00	2,00	2,00	2,00	1,00	37,00	#NULL!
68,00	2,00	1,00	2,00	2,00	2,00	2,00	#NULL!	#NULL!
45,00	1,00	1,00	2,00	1,00	2,00	2,00	#NULL!	#NULL!
76,00	1,00	1,00	1,00	2,00	2,00	2,00	#NULL!	#NULL!
52,00	1,00	1,00	2,00	1,00	2,00	1,00	15,00	#NULL!
45,00	2,00	2,00	2,00	2,00	2,00	2,00	#NULL!	#NULL!
62,00	2,00	1,00	2,00	1,00	1,00	2,00	#NULL!	#NULL!
49,00	2,00	2,00	1,00	2,00	1,00	2,00	#NULL!	#NULL!
68,00	2,00	1,00	2,00	1,00	1,00	2,00	#NULL!	#NULL!
71,00	1,00	1,00	2,00	2,00	2,00	2,00	#NULL!	#NULL!
52,00	2,00	1,00	2,00	2,00	2,00	2,00	#NULL!	#NULL!
53,00	1,00	2,00	2,00	2,00	2,00	2,00	#NULL!	#NULL!
56,00	1,00	1,00	1,00	2,00	2,00	1,00	20,00	#NULL!
52,00	2,00	2,00	2,00	2,00	2,00	2,00	#NULL!	#NULL!
51,00	2,00	1,00	2,00	2,00	1,00	2,00	#NULL!	#NULL!
55,00	1,00	2,00	2,00	2,00	2,00	1,00	20,00	#NULL!
65,00	2,00	1,00	1,00	1,00	1,00	2,00	#NULL!	#NULL!

48,00	2,00	1,00	1,00	2,00	2,00	1,00	#NULL!	#NULL!
55,00	1,00	1,00	2,00	2,00	2,00	1,00	30,00	#NULL!
56,00	2,00	1,00	2,00	1,00	1,00	2,00	#NULL!	#NULL!
50,00	2,00	1,00	2,00	1,00	2,00	2,00	#NULL!	#NULL!
57,00	2,00	2,00	2,00	2,00	2,00	1,00	15,00	#NULL!
60,00	2,00	2,00	2,00	2,00	2,00	2,00	#NULL!	#NULL!
58,00	2,00	2,00	1,00	2,00	2,00	2,00	#NULL!	#NULL!
64,00	1,00	2,00	2,00	2,00	2,00	1,00	30,00	#NULL!
58,00	2,00	2,00	2,00	2,00	2,00	2,00	#NULL!	#NULL!
63,00	1,00	1,00	1,00	1,00	1,00	2,00	#NULL!	#NULL!
80,00	2,00	1,00	1,00	1,00	2,00	2,00	#NULL!	#NULL!
54,00	2,00	2,00	2,00	2,00	2,00	2,00	#NULL!	#NULL!
59,00	1,00	2,00	2,00	2,00	2,00	2,00	#NULL!	#NULL!
51,00	1,00	2,00	2,00	2,00	2,00	2,00	#NULL!	#NULL!
64,00	1,00	2,00	2,00	2,00	2,00	2,00	#NULL!	#NULL!
64,00	1,00	1,00	2,00	2,00	2,00	1,00	40,00	#NULL!
59,00	1,00	1,00	2,00	1,00	2,00	1,00	15,00	#NULL!
56,00	2,00	2,00	2,00	2,00	2,00	2,00	#NULL!	#NULL!
59,00	1,00	2,00	2,00	2,00	2,00	2,00	#NULL!	#NULL!
45,00	2,00	2,00	2,00	2,00	2,00	2,00	#NULL!	#NULL!
46,00	1,00	2,00	2,00	2,00	2,00	2,00	#NULL!	#NULL!
51,00	1,00	2,00	2,00	2,00	2,00	2,00	#NULL!	#NULL!
55,00	1,00	2,00	2,00	2,00	2,00	2,00	#NULL!	#NULL!
47,00	2,00	1,00	2,00	2,00	2,00	2,00	#NULL!	#NULL!
51,00	2,00	2,00	2,00	2,00	2,00	2,00	#NULL!	#NULL!
47,00	2,00	2,00	2,00	2,00	2,00	1,00	30,00	#NULL!
45,00	2,00	2,00	2,00	2,00	2,00	2,00	#NULL!	#NULL!
53,00	2,00	2,00	2,00	2,00	2,00	2,00	#NULL!	#NULL!
56,00	2,00	2,00	2,00	2,00	2,00	1,00	28,00	#NULL!
51,00	2,00	2,00	2,00	2,00	2,00	2,00	#NULL!	#NULL!
56,00	1,00	2,00	2,00	1,00	2,00	2,00	#NULL!	#NULL!
80,00	2,00	1,00	1,00	1,00	1,00	2,00	#NULL!	#NULL!
51,00	2,00	2,00	2,00	2,00	2,00	2,00	#NULL!	#NULL!
55,00	1,00	1,00	2,00	2,00	2,00	2,00	#NULL!	#NULL!
50,00	2,00	2,00	1,00	2,00	2,00	2,00	#NULL!	#NULL!
51,00	1,00	2,00	2,00	2,00	1,00	1,00	26,00	#NULL!
47,00	2,00	1,00	2,00	1,00	1,00	1,00	20,00	#NULL!
50,00	1,00	1,00	1,00	1,00	1,00	1,00	80,00	#NULL!
46,00	2,00	2,00	2,00	2,00	2,00	2,00	#NULL!	#NULL!
61,00	1,00	2,00	1,00	1,00	1,00	2,00	#NULL!	#NULL!
46,00	2,00	1,00	2,00	2,00	2,00	2,00	#NULL!	#NULL!
60,00	2,00	1,00	1,00	2,00	2,00	2,00	#NULL!	#NULL!
50,00	1,00	2,00	1,00	2,00	1,00	2,00	#NULL!	#NULL!
66,00	1,00	2,00	2,00	1,00	2,00	2,00	#NULL!	#NULL!
60,00	2,00	2,00	2,00	2,00	2,00	2,00	#NULL!	#NULL!
59,00	1,00	1,00	2,00	2,00	2,00	2,00	#NULL!	#NULL!
51,00	1,00	2,00	2,00	2,00	1,00	2,00	#NULL!	#NULL!
45,00	2,00	2,00	2,00	2,00	2,00	2,00	#NULL!	#NULL!
54,00	2,00	2,00	2,00	2,00	2,00	2,00	#NULL!	#NULL!
76,00	2,00	2,00	2,00	2,00	1,00	2,00	#NULL!	#NULL!

70,00	2,00	1,00	2,00	1,00	1,00	2,00	#NULL!	#NULL!
52,00	1,00	1,00	2,00	2,00	2,00	2,00	#NULL!	#NULL!
49,00	1,00	1,00	2,00	2,00	2,00	1,00	40,00	#NULL!
59,00	2,00	1,00	1,00	2,00	1,00	2,00	#NULL!	#NULL!
45,00	2,00	2,00	2,00	2,00	2,00	2,00	#NULL!	#NULL!
51,00	2,00	2,00	2,00	2,00	2,00	2,00	#NULL!	#NULL!
56,00	2,00	1,00	2,00	2,00	2,00	2,00	#NULL!	#NULL!
55,00	1,00	2,00	2,00	2,00	2,00	2,00	#NULL!	#NULL!
53,00	2,00	1,00	2,00	2,00	2,00	2,00	#NULL!	#NULL!
45,00	1,00	2,00	2,00	2,00	2,00	1,00	64,00	#NULL!
48,00	1,00	2,00	2,00	2,00	2,00	2,00	#NULL!	#NULL!
56,00	1,00	2,00	2,00	2,00	2,00	2,00	#NULL!	#NULL!
59,00	1,00	2,00	2,00	2,00	2,00	2,00	#NULL!	#NULL!
46,00	2,00	1,00	1,00	2,00	2,00	2,00	#NULL!	#NULL!
49,00	1,00	2,00	2,00	2,00	2,00	2,00	#NULL!	#NULL!
61,00	1,00	2,00	2,00	2,00	2,00	2,00	#NULL!	#NULL!
64,00	1,00	2,00	2,00	2,00	2,00	2,00	#NULL!	#NULL!
56,00	1,00	2,00	2,00	2,00	2,00	2,00	#NULL!	#NULL!
50,00	2,00	2,00	2,00	2,00	2,00	2,00	#NULL!	#NULL!
55,00	2,00	1,00	2,00	2,00	1,00	2,00	#NULL!	#NULL!
56,00	2,00	1,00	2,00	2,00	2,00	2,00	#NULL!	#NULL!
51,00	2,00	2,00	1,00	2,00	1,00	2,00	#NULL!	#NULL!
45,00	1,00	1,00	2,00	2,00	2,00	2,00	#NULL!	#NULL!
69,00	1,00	2,00	2,00	2,00	2,00	2,00	#NULL!	#NULL!
59,00	1,00	2,00	2,00	2,00	2,00	2,00	#NULL!	#NULL!
60,00	2,00	2,00	2,00	2,00	2,00	2,00	#NULL!	#NULL!
51,00	1,00	2,00	2,00	2,00	2,00	2,00	#NULL!	#NULL!
51,00	1,00	1,00	2,00	2,00	2,00	2,00	#NULL!	#NULL!
50,00	2,00	1,00	2,00	2,00	2,00	2,00	#NULL!	#NULL!
88,00	1,00	1,00	1,00	2,00	2,00	2,00	#NULL!	#NULL!
78,00	2,00	2,00	2,00	2,00	2,00	2,00	#NULL!	#NULL!
59,00	1,00	2,00	2,00	2,00	1,00	2,00	#NULL!	#NULL!
49,00	2,00	2,00	2,00	2,00	1,00	2,00	#NULL!	#NULL!
79,00	1,00	1,00	1,00	2,00	2,00	2,00	#NULL!	#NULL!
60,00	1,00	1,00	2,00	2,00	2,00	2,00	#NULL!	#NULL!
55,00	1,00	1,00	2,00	2,00	2,00	2,00	#NULL!	#NULL!
46,00	2,00	2,00	2,00	2,00	2,00	2,00	#NULL!	#NULL!
51,00	2,00	2,00	1,00	2,00	1,00	2,00	#NULL!	#NULL!
53,00	1,00	1,00	2,00	2,00	2,00	2,00	#NULL!	#NULL!
85,00	2,00	1,00	1,00	1,00	1,00	2,00	#NULL!	#NULL!
89,00	1,00	1,00	2,00	2,00	2,00	2,00	#NULL!	#NULL!
62,00	1,00	1,00	2,00	2,00	2,00	2,00	#NULL!	#NULL!
65,00	1,00	2,00	1,00	1,00	1,00	2,00	#NULL!	#NULL!
59,00	1,00	1,00	2,00	2,00	2,00	2,00	#NULL!	#NULL!
59,00	2,00	1,00	1,00	1,00	2,00	2,00	#NULL!	#NULL!
70,00	2,00	1,00	1,00	2,00	2,00	2,00	#NULL!	#NULL!
77,00	2,00	1,00	1,00	1,00	1,00	2,00	#NULL!	#NULL!
45,00	2,00	2,00	2,00	2,00	2,00	2,00	#NULL!	#NULL!
49,00	1,00	2,00	2,00	2,00	2,00	2,00	#NULL!	#NULL!
48,00	1,00	2,00	2,00	2,00	2,00	2,00	#NULL!	#NULL!

45,00	2,00	2,00	2,00	2,00	2,00	2,00	#NULL!	#NULL!
55,00	2,00	2,00	2,00	2,00	2,00	2,00	#NULL!	#NULL!
59,00	2,00	2,00	2,00	2,00	2,00	2,00	#NULL!	#NULL!
46,00	1,00	2,00	2,00	2,00	2,00	2,00	#NULL!	#NULL!
49,00	1,00	2,00	2,00	2,00	2,00	2,00	#NULL!	#NULL!
69,00	1,00	1,00	2,00	1,00	2,00	1,00	32,00	#NULL!
67,00	2,00	1,00	2,00	2,00	2,00	1,00	25,00	#NULL!
46,00	2,00	2,00	2,00	2,00	2,00	2,00	#NULL!	#NULL!
50,00	1,00	1,00	2,00	2,00	2,00	2,00	#NULL!	#NULL!
62,00	2,00	1,00	1,00	1,00	1,00	1,00	20,00	#NULL!
60,00	2,00	1,00	2,00	2,00	2,00	1,00	10,00	#NULL!
52,00	2,00	1,00	2,00	1,00	2,00	2,00	#NULL!	#NULL!
56,00	2,00	1,00	2,00	2,00	2,00	2,00	#NULL!	#NULL!
59,00	2,00	1,00	2,00	1,00	2,00	2,00	#NULL!	#NULL!
60,00	1,00	2,00	1,00	2,00	1,00	1,00	40,00	#NULL!
60,00	2,00	1,00	2,00	2,00	2,00	2,00	#NULL!	#NULL!
62,00	1,00	1,00	2,00	2,00	2,00	2,00	#NULL!	#NULL!
46,00	2,00	1,00	1,00	2,00	2,00	2,00	#NULL!	#NULL!
49,00	1,00	1,00	2,00	1,00	1,00	1,00	30,00	#NULL!
46,00	2,00	1,00	2,00	2,00	1,00	2,00	#NULL!	#NULL!
49,00	2,00	1,00	1,00	2,00	2,00	2,00	#NULL!	#NULL!
59,00	1,00	2,00	2,00	2,00	2,00	2,00	#NULL!	#NULL!
58,00	2,00	2,00	2,00	1,00	1,00	2,00	#NULL!	#NULL!
46,00	1,00	1,00	2,00	2,00	2,00	2,00	#NULL!	#NULL!
47,00	2,00	1,00	2,00	2,00	2,00	2,00	#NULL!	#NULL!
52,00	1,00	1,00	2,00	2,00	2,00	2,00	#NULL!	#NULL!
50,00	2,00	2,00	2,00	2,00	2,00	2,00	#NULL!	#NULL!
55,00	1,00	1,00	2,00	2,00	2,00	2,00	#NULL!	#NULL!
57,00	1,00	2,00	1,00	2,00	2,00	2,00	#NULL!	#NULL!
49,00	2,00	2,00	2,00	2,00	2,00	2,00	#NULL!	#NULL!
48,00	2,00	2,00	2,00	2,00	2,00	2,00	#NULL!	#NULL!
46,00	2,00	2,00	2,00	2,00	2,00	2,00	#NULL!	#NULL!
55,00	1,00	1,00	2,00	1,00	2,00	2,00	#NULL!	#NULL!
46,00	2,00	2,00	2,00	2,00	2,00	2,00	#NULL!	#NULL!
46,00	1,00	2,00	2,00	2,00	2,00	2,00	#NULL!	#NULL!
58,00	2,00	1,00	2,00	1,00	1,00	1,00	30,00	#NULL!
61,00	2,00	1,00	1,00	2,00	2,00	2,00	#NULL!	#NULL!
51,00	2,00	2,00	2,00	2,00	2,00	2,00	#NULL!	#NULL!
58,00	1,00	1,00	2,00	1,00	1,00	1,00	15,00	#NULL!
52,00	2,00	1,00	2,00	1,00	2,00	2,00	#NULL!	#NULL!
59,00	1,00	2,00	1,00	1,00	2,00	1,00	40,00	#NULL!
66,00	2,00	1,00	2,00	2,00	2,00	2,00	#NULL!	#NULL!
70,00	1,00	1,00	2,00	1,00	1,00	2,00	#NULL!	#NULL!
62,00	2,00	2,00	2,00	2,00	2,00	2,00	#NULL!	#NULL!
63,00	1,00	2,00	2,00	2,00	2,00	1,00	40,00	#NULL!
76,00	1,00	2,00	2,00	1,00	1,00	1,00	40,00	#NULL!
74,00	2,00	1,00	2,00	2,00	2,00	2,00	#NULL!	#NULL!
70,00	2,00	1,00	2,00	1,00	2,00	1,00	10,00	#NULL!
76,00	2,00	1,00	1,00	2,00	2,00	1,00	20,00	#NULL!
78,00	2,00	1,00	2,00	1,00	2,00	2,00	#NULL!	#NULL!

80,00	1,00	2,00	2,00	1,00	1,00	2,00	#NULL!	#NULL!
62,00	2,00	1,00	1,00	2,00	2,00	2,00	#NULL!	#NULL!
69,00	1,00	2,00	1,00	2,00	1,00	2,00	#NULL!	#NULL!
46,00	2,00	2,00	2,00	2,00	2,00	2,00	#NULL!	#NULL!
49,00	1,00	2,00	2,00	2,00	2,00	2,00	#NULL!	#NULL!
46,00	2,00	2,00	2,00	2,00	2,00	2,00	#NULL!	#NULL!
50,00	1,00	1,00	2,00	2,00	2,00	2,00	#NULL!	#NULL!
63,00	1,00	1,00	2,00	2,00	2,00	2,00	#NULL!	#NULL!
59,00	2,00	1,00	2,00	2,00	2,00	2,00	#NULL!	#NULL!
55,00	1,00	2,00	2,00	2,00	2,00	2,00	#NULL!	#NULL!
59,00	2,00	1,00	2,00	1,00	1,00	2,00	#NULL!	#NULL!
54,00	2,00	1,00	2,00	2,00	2,00	2,00	#NULL!	#NULL!
60,00	1,00	1,00	1,00	2,00	2,00	2,00	#NULL!	#NULL!
65,00	1,00	1,00	2,00	2,00	2,00	1,00	20,00	#NULL!
60,00	2,00	2,00	2,00	2,00	2,00	1,00	15,00	#NULL!
45,00	2,00	2,00	2,00	2,00	2,00	2,00	#NULL!	#NULL!
46,00	1,00	2,00	2,00	2,00	2,00	2,00	#NULL!	#NULL!
59,00	2,00	2,00	2,00	2,00	2,00	1,00	40,00	#NULL!
61,00	1,00	1,00	2,00	1,00	1,00	2,00	#NULL!	#NULL!
65,00	2,00	1,00	2,00	2,00	2,00	2,00	#NULL!	#NULL!
58,00	2,00	1,00	1,00	2,00	1,00	2,00	#NULL!	#NULL!
58,00	2,00	1,00	2,00	2,00	2,00	2,00	#NULL!	#NULL!
60,00	2,00	1,00	2,00	2,00	2,00	2,00	#NULL!	#NULL!
46,00	1,00	2,00	2,00	2,00	2,00	2,00	#NULL!	#NULL!
45,00	2,00	2,00	2,00	2,00	2,00	2,00	#NULL!	#NULL!
71,00	1,00	1,00	2,00	2,00	2,00	2,00	60,00	4,00
69,00	2,00	1,00	2,00	2,00	2,00	2,00	#NULL!	#NULL!
70,00	2,00	1,00	2,00	2,00	2,00	2,00	#NULL!	#NULL!
50,00	2,00	2,00	2,00	2,00	2,00	2,00	#NULL!	#NULL!
54,00	1,00	1,00	1,00	2,00	1,00	1,00	35,00	#NULL!
51,00	2,00	1,00	1,00	2,00	2,00	2,00	#NULL!	#NULL!
55,00	2,00	2,00	2,00	2,00	1,00	2,00	#NULL!	#NULL!
58,00	1,00	2,00	2,00	2,00	1,00	2,00	#NULL!	#NULL!
52,00	1,00	2,00	2,00	2,00	2,00	1,00	12,00	#NULL!
50,00	2,00	2,00	2,00	2,00	2,00	2,00	#NULL!	#NULL!
45,00	1,00	2,00	2,00	2,00	2,00	1,00	30,00	#NULL!
47,00	1,00	2,00	2,00	2,00	1,00	2,00	#NULL!	#NULL!
53,00	2,00	2,00	1,00	2,00	1,00	2,00	#NULL!	#NULL!
45,00	2,00	2,00	21,00	2,00	2,00	2,00	#NULL!	#NULL!
55,00	1,00	2,00	2,00	2,00	2,00	1,00	60,00	#NULL!
48,00	1,00	2,00	2,00	2,00	2,00	2,00	#NULL!	#NULL!
46,00	2,00	2,00	2,00	2,00	2,00	1,00	30,00	#NULL!
74,00	2,00	1,00	2,00	2,00	2,00	2,00	#NULL!	#NULL!
48,00	2,00	2,00	1,00	2,00	2,00	2,00	#NULL!	#NULL!
49,00	1,00	2,00	2,00	2,00	2,00	2,00	#NULL!	#NULL!
48,00	2,00	2,00	2,00	2,00	2,00	2,00	#NULL!	#NULL!
54,00	2,00	1,00	1,00	1,00	1,00	2,00	#NULL!	#NULL!
65,00	2,00	2,00	2,00	2,00	2,00	2,00	#NULL!	#NULL!
70,00	1,00	1,00	2,00	1,00	2,00	2,00	#NULL!	#NULL!
58,00	2,00	2,00	2,00	2,00	1,00	2,00	#NULL!	#NULL!

58,00	1,00	2,00	2,00	1,00	1,00	2,00	#NULL!	#NULL!
51,00	2,00	2,00	2,00	2,00	2,00	2,00	#NULL!	#NULL!
62,00	2,00	1,00	2,00	2,00	2,00	2,00	#NULL!	#NULL!
52,00	2,00	1,00	2,00	1,00	1,00	2,00	#NULL!	#NULL!
46,00	2,00	2,00	2,00	2,00	2,00	2,00	#NULL!	#NULL!
48,00	2,00	2,00	2,00	2,00	2,00	1,00	40,00	#NULL!
60,00	1,00	1,00	2,00	2,00	2,00	2,00	#NULL!	#NULL!
80,00	2,00	1,00	1,00	1,00	1,00	2,00	#NULL!	#NULL!
50,00	2,00	2,00	2,00	2,00	2,00	1,00	20,00	#NULL!
73,00	2,00	1,00	1,00	2,00	2,00	2,00	#NULL!	#NULL!
40,00	2,00	2,00	2,00	2,00	2,00	2,00	#NULL!	#NULL!
55,00	2,00	2,00	2,00	2,00	2,00	2,00	#NULL!	#NULL!
60,00	1,00	2,00	2,00	2,00	2,00	2,00	#NULL!	#NULL!
50,00	2,00	1,00	2,00	1,00	2,00	1,00	10,00	#NULL!
50,00	2,00	1,00	2,00	2,00	2,00	1,00	15,00	#NULL!
45,00	2,00	2,00	2,00	2,00	2,00	2,00	#NULL!	#NULL!
62,00	2,00	1,00	2,00	2,00	2,00	2,00	#NULL!	#NULL!
45,00	2,00	2,00	2,00	2,00	2,00	2,00	#NULL!	#NULL!
53,00	1,00	2,00	1,00	2,00	2,00	2,00	#NULL!	#NULL!
72,00	1,00	1,00	2,00	1,00	2,00	2,00	#NULL!	#NULL!
52,00	2,00	1,00	2,00	2,00	1,00	2,00	#NULL!	#NULL!
81,00	1,00	1,00	1,00	2,00	2,00	2,00	#NULL!	#NULL!
75,00	2,00	1,00	1,00	1,00	1,00	2,00	#NULL!	#NULL!
57,00	1,00	2,00	2,00	2,00	2,00	2,00	#NULL!	#NULL!
80,00	2,00	1,00	2,00	2,00	2,00	2,00	#NULL!	#NULL!
47,00	1,00	2,00	2,00	2,00	2,00	1,00	30,00	#NULL!
71,00	2,00	1,00	2,00	1,00	2,00	2,00	#NULL!	#NULL!
48,00	2,00	2,00	2,00	2,00	2,00	1,00	20,00	#NULL!
45,00	2,00	2,00	2,00	2,00	2,00	2,00	#NULL!	#NULL!
50,00	2,00	2,00	1,00	2,00	2,00	2,00	#NULL!	#NULL!
45,00	2,00	1,00	2,00	1,00	2,00	2,00	#NULL!	#NULL!
47,00	2,00	2,00	2,00	2,00	2,00	2,00	#NULL!	#NULL!
55,00	1,00	1,00	2,00	2,00	1,00	2,00	#NULL!	#NULL!
58,00	1,00	2,00	2,00	2,00	2,00	2,00	#NULL!	#NULL!
82,00	2,00	1,00	2,00	1,00	2,00	2,00	#NULL!	#NULL!
70,00	1,00	1,00	2,00	2,00	2,00	2,00	#NULL!	#NULL!
54,00	2,00	1,00	2,00	2,00	2,00	1,00	20,00	#NULL!
65,00	1,00	1,00	2,00	2,00	2,00	1,00	40,00	#NULL!
56,00	2,00	2,00	1,00	2,00	2,00	2,00	#NULL!	#NULL!
50,00	2,00	2,00	2,00	1,00	1,00	2,00	#NULL!	#NULL!
79,00	2,00	1,00	2,00	2,00	2,00	2,00	#NULL!	#NULL!
76,00	1,00	2,00	2,00	2,00	2,00	2,00	#NULL!	#NULL!
69,00	2,00	1,00	2,00	2,00	1,00	2,00	#NULL!	#NULL!
70,00	1,00	1,00	2,00	2,00	2,00	2,00	#NULL!	#NULL!
65,00	2,00	2,00	2,00	2,00	2,00	2,00	#NULL!	#NULL!
52,00	2,00	1,00	2,00	1,00	1,00	2,00	#NULL!	#NULL!
51,00	2,00	2,00	2,00	2,00	2,00	2,00	#NULL!	#NULL!
52,00	2,00	2,00	2,00	2,00	2,00	1,00	30,00	#NULL!
60,00	2,00	1,00	1,00	1,00	1,00	2,00	#NULL!	#NULL!
72,00	2,00	1,00	2,00	2,00	2,00	2,00	#NULL!	#NULL!

80,00	1,00	1,00	1,00	2,00	1,00	2,00	#NULL!	#NULL!
55,00	1,00	2,00	1,00	2,00	2,00	2,00	#NULL!	#NULL!
54,00	2,00	2,00	2,00	2,00	2,00	2,00	#NULL!	#NULL!
77,00	2,00	2,00	1,00	2,00	2,00	2,00	#NULL!	#NULL!
46,00	2,00	2,00	2,00	2,00	2,00	2,00	#NULL!	#NULL!
67,00	2,00	1,00	2,00	2,00	2,00	1,00	40,00	#NULL!
60,00	2,00	1,00	1,00	2,00	2,00	2,00	#NULL!	#NULL!
68,00	1,00	2,00	2,00	2,00	1,00	2,00	#NULL!	#NULL!
71,00	2,00	1,00	1,00	1,00	2,00	2,00	#NULL!	#NULL!
45,00	2,00	1,00	2,00	2,00	2,00	2,00	#NULL!	#NULL!
72,00	1,00	1,00	2,00	2,00	2,00	2,00	#NULL!	#NULL!
64,00	1,00	1,00	2,00	2,00	2,00	2,00	#NULL!	#NULL!
56,00	2,00	2,00	2,00	2,00	2,00	2,00	#NULL!	#NULL!
58,00	2,00	2,00	2,00	2,00	1,00	2,00	#NULL!	#NULL!
65,00	2,00	1,00	2,00	1,00	2,00	2,00	#NULL!	#NULL!
49,00	1,00	2,00	2,00	1,00	2,00	2,00	#NULL!	#NULL!
46,00	2,00	2,00	2,00	2,00	2,00	1,00	20,00	#NULL!
51,00	2,00	2,00	2,00	2,00	2,00	2,00	#NULL!	#NULL!
67,00	2,00	2,00	1,00	1,00	2,00	2,00	20,00	2,00
62,00	2,00	1,00	2,00	2,00	2,00	2,00	#NULL!	#NULL!
65,00	2,00	2,00	2,00	1,00	2,00	2,00	#NULL!	#NULL!
54,00	2,00	2,00	2,00	2,00	2,00	2,00	#NULL!	#NULL!
81,00	2,00	1,00	2,00	1,00	1,00	2,00	30,00	5,00
50,00	1,00	2,00	2,00	2,00	2,00	2,00	#NULL!	#NULL!
82,00	2,00	2,00	2,00	2,00	2,00	2,00	#NULL!	#NULL!
59,00	1,00	1,00	2,00	1,00	2,00	2,00	#NULL!	#NULL!
48,00	1,00	2,00	2,00	2,00	2,00	1,00	20,00	#NULL!
49,00	2,00	1,00	2,00	2,00	2,00	1,00	30,00	#NULL!
48,00	2,00	2,00	2,00	2,00	2,00	2,00	#NULL!	#NULL!
66,00	1,00	1,00	2,00	1,00	1,00	1,00	92,00	#NULL!
60,00	2,00	1,00	2,00	2,00	2,00	1,00	20,00	#NULL!
89,00	1,00	1,00	2,00	2,00	2,00	2,00	30,00	39,00
85,00	2,00	1,00	1,00	2,00	2,00	2,00	#NULL!	#NULL!
57,00	2,00	1,00	2,00	1,00	2,00	2,00	#NULL!	#NULL!
60,00	1,00	1,00	1,00	1,00	1,00	1,00	80,00	#NULL!
46,00	2,00	2,00	2,00	2,00	2,00	2,00	#NULL!	#NULL!
49,00	2,00	2,00	2,00	2,00	2,00	2,00	#NULL!	#NULL!
47,00	1,00	2,00	2,00	1,00	2,00	2,00	#NULL!	#NULL!
61,00	2,00	1,00	1,00	1,00	1,00	2,00	#NULL!	#NULL!
64,00	1,00	1,00	1,00	1,00	1,00	2,00	#NULL!	#NULL!
70,00	2,00	1,00	2,00	2,00	2,00	2,00	#NULL!	#NULL!
58,00	2,00	2,00	2,00	2,00	2,00	1,00	120,00	#NULL!
60,00	2,00	1,00	2,00	2,00	2,00	2,00	#NULL!	#NULL!
50,00	1,00	2,00	2,00	2,00	2,00	2,00	#NULL!	#NULL!
70,00	2,00	1,00	1,00	1,00	2,00	2,00	#NULL!	#NULL!
69,00	1,00	1,00	2,00	2,00	2,00	2,00	#NULL!	#NULL!
62,00	2,00	1,00	1,00	2,00	1,00	2,00	#NULL!	#NULL!
58,00	1,00	2,00	2,00	2,00	2,00	1,00	20,00	#NULL!
53,00	2,00	1,00	1,00	2,00	2,00	2,00	#NULL!	#NULL!
71,00	1,00	1,00	1,00	2,00	1,00	1,00	50,00	#NULL!

65,00	2,00	2,00	2,00	2,00	2,00	2,00	#NULL!	#NULL!

alkol	inme	svotipi	düzelme	günlükiş	son6ay	VAR00001
2,00	2,00	#NULL!	#NULL!	#NULL!	#NULL!	#NULL!
2,00	2,00	#NULL!	#NULL!	#NULL!	#NULL!	#NULL!
2,00	2,00	#NULL!	#NULL!	#NULL!	#NULL!	#NULL!
2,00	2,00	#NULL!	#NULL!	#NULL!	#NULL!	#NULL!
2,00	2,00	#NULL!	#NULL!	#NULL!	#NULL!	#NULL!
2,00	2,00	#NULL!	#NULL!	#NULL!	#NULL!	#NULL!
2,00	2,00	#NULL!	#NULL!	#NULL!	#NULL!	#NULL!
1,00	2,00	#NULL!	#NULL!	#NULL!	#NULL!	#NULL!
2,00	2,00	#NULL!	#NULL!	#NULL!	#NULL!	#NULL!
2,00	2,00	#NULL!	#NULL!	#NULL!	#NULL!	#NULL!
2,00	2,00	#NULL!	#NULL!	#NULL!	#NULL!	#NULL!
2,00	2,00	#NULL!	#NULL!	#NULL!	#NULL!	#NULL!
2,00	2,00	#NULL!	#NULL!	#NULL!	#NULL!	#NULL!
2,00	2,00	#NULL!	#NULL!	#NULL!	#NULL!	#NULL!
2,00	2,00	#NULL!	#NULL!	#NULL!	#NULL!	#NULL!
2,00	2,00	#NULL!	#NULL!	#NULL!	#NULL!	#NULL!
2,00	2,00	#NULL!	#NULL!	#NULL!	#NULL!	#NULL!
2,00	2,00	#NULL!	#NULL!	#NULL!	#NULL!	#NULL!
2,00	2,00	#NULL!	#NULL!	#NULL!	#NULL!	#NULL!
1,00	2,00	#NULL!	#NULL!	#NULL!	#NULL!	#NULL!
2,00	2,00	#NULL!	#NULL!	#NULL!	#NULL!	#NULL!
2,00	2,00	#NULL!	#NULL!	#NULL!	#NULL!	#NULL!
2,00	2,00	#NULL!	#NULL!	#NULL!	#NULL!	#NULL!
2,00	2,00	#NULL!	#NULL!	#NULL!	#NULL!	#NULL!
2,00	2,00	#NULL!	#NULL!	#NULL!	#NULL!	#NULL!
2,00	2,00	#NULL!	#NULL!	#NULL!	#NULL!	#NULL!
2,00	2,00	#NULL!	#NULL!	#NULL!	#NULL!	#NULL!
2,00	2,00	#NULL!	#NULL!	#NULL!	#NULL!	#NULL!
2,00	2,00	#NULL!	#NULL!	#NULL!	#NULL!	#NULL!
2,00	2,00	#NULL!	#NULL!	#NULL!	#NULL!	#NULL!
2,00	2,00	#NULL!	#NULL!	#NULL!	#NULL!	#NULL!
2,00	2,00	#NULL!	#NULL!	#NULL!	#NULL!	#NULL!
2,00	2,00	#NULL!	#NULL!	#NULL!	#NULL!	#NULL!
2,00	2,00	#NULL!	#NULL!	#NULL!	#NULL!	#NULL!
2,00	2,00	#NULL!	#NULL!	#NULL!	#NULL!	#NULL!
2,00	2,00	#NULL!	#NULL!	#NULL!	#NULL!	#NULL!
2,00	2,00	#NULL!	#NULL!	#NULL!	#NULL!	#NULL!
2,00	2,00	#NULL!	#NULL!	#NULL!	#NULL!	#NULL!
2,00	2,00	#NULL!	#NULL!	#NULL!	#NULL!	#NULL!
2,00	2,00	#NULL!	#NULL!	#NULL!	#NULL!	#NULL!
2,00	2,00	#NULL!	#NULL!	#NULL!	#NULL!	#NULL!
2,00	2,00	#NULL!	#NULL!	#NULL!	#NULL!	#NULL!
2,00	2,00	#NULL!	#NULL!	#NULL!	#NULL!	#NULL!
2,00	2,00	#NULL!	#NULL!	#NULL!	#NULL!	#NULL!
2,00	2,00	#NULL!	#NULL!	#NULL!	#NULL!	#NULL!
2,00	2,00	#NULL!	#NULL!	#NULL!	#NULL!	#NULL!
2,00	2,00	#NULL!	#NULL!	#NULL!	#NULL!	#NULL!
2,00	2,00	#NULL!	#NULL!	#NULL!	#NULL!	#NULL!
2,00	2,00	#NULL!	#NULL!	#NULL!	#NULL!	#NULL!

2,00	2,00	#NULL!	#NULL!	#NULL!	#NULL!	#NULL!
2,00	2,00	#NULL!	#NULL!	#NULL!	#NULL!	#NULL!
2,00	2,00	#NULL!	#NULL!	#NULL!	#NULL!	#NULL!
2,00	2,00	#NULL!	#NULL!	#NULL!	#NULL!	#NULL!
2,00	2,00	#NULL!	#NULL!	#NULL!	#NULL!	#NULL!
2,00	2,00	#NULL!	#NULL!	#NULL!	#NULL!	#NULL!
2,00	2,00	#NULL!	#NULL!	#NULL!	#NULL!	#NULL!
2,00	2,00	#NULL!	#NULL!	#NULL!	#NULL!	#NULL!
2,00	2,00	#NULL!	#NULL!	#NULL!	#NULL!	#NULL!
2,00	2,00	#NULL!	#NULL!	#NULL!	#NULL!	#NULL!
2,00	2,00	#NULL!	#NULL!	#NULL!	#NULL!	#NULL!
2,00	2,00	#NULL!	#NULL!	#NULL!	#NULL!	#NULL!
2,00	2,00	#NULL!	#NULL!	#NULL!	#NULL!	#NULL!
2,00	2,00	#NULL!	#NULL!	#NULL!	#NULL!	#NULL!
2,00	2,00	#NULL!	#NULL!	#NULL!	#NULL!	#NULL!
2,00	2,00	#NULL!	#NULL!	#NULL!	#NULL!	#NULL!
2,00	2,00	#NULL!	#NULL!	#NULL!	#NULL!	#NULL!
2,00	2,00	#NULL!	#NULL!	#NULL!	#NULL!	#NULL!
2,00	2,00	#NULL!	#NULL!	#NULL!	#NULL!	#NULL!
2,00	2,00	#NULL!	#NULL!	#NULL!	#NULL!	#NULL!
2,00	2,00	#NULL!	#NULL!	#NULL!	#NULL!	#NULL!
2,00	2,00	#NULL!	#NULL!	#NULL!	#NULL!	#NULL!
2,00	2,00	#NULL!	#NULL!	#NULL!	#NULL!	#NULL!
2,00	2,00	#NULL!	#NULL!	#NULL!	#NULL!	#NULL!
2,00	2,00	#NULL!	#NULL!	#NULL!	#NULL!	#NULL!
2,00	2,00	#NULL!	#NULL!	#NULL!	#NULL!	#NULL!
2,00	2,00	#NULL!	#NULL!	#NULL!	#NULL!	#NULL!
2,00	2,00	#NULL!	#NULL!	#NULL!	#NULL!	#NULL!
2,00	2,00	#NULL!	#NULL!	#NULL!	#NULL!	#NULL!
2,00	2,00	#NULL!	#NULL!	#NULL!	#NULL!	#NULL!
2,00	2,00	#NULL!	#NULL!	#NULL!	#NULL!	#NULL!
2,00	2,00	#NULL!	#NULL!	#NULL!	#NULL!	#NULL!
2,00	2,00	#NULL!	#NULL!	#NULL!	#NULL!	#NULL!
2,00	2,00	#NULL!	#NULL!	#NULL!	#NULL!	#NULL!
2,00	2,00	#NULL!	#NULL!	#NULL!	#NULL!	#NULL!
2,00	2,00	#NULL!	#NULL!	#NULL!	#NULL!	#NULL!
2,00	2,00	#NULL!	#NULL!	#NULL!	#NULL!	#NULL!
2,00	2,00	#NULL!	#NULL!	#NULL!	#NULL!	#NULL!
2,00	2,00	#NULL!	#NULL!	#NULL!	#NULL!	#NULL!
2,00	2,00	#NULL!	#NULL!	#NULL!	#NULL!	#NULL!
2,00	2,00	#NULL!	#NULL!	#NULL!	#NULL!	#NULL!
2,00	2,00	#NULL!	#NULL!	#NULL!	#NULL!	#NULL!
2,00	2,00	#NULL!	#NULL!	#NULL!	#NULL!	#NULL!
2,00	2,00	#NULL!	#NULL!	#NULL!	#NULL!	#NULL!
2,00	2,00	#NULL!	#NULL!	#NULL!	#NULL!	#NULL!
2,00	2,00	#NULL!	#NULL!	#NULL!	#NULL!	#NULL!
2,00	2,00	#NULL!	#NULL!	#NULL!	#NULL!	#NULL!
2,00	2,00	#NULL!	#NULL!	#NULL!	#NULL!	#NULL!
2,00	2,00	#NULL!	#NULL!	#NULL!	#NULL!	#NULL!
2,00	2,00	#NULL!	#NULL!	#NULL!	#NULL!	#NULL!

2,00	2,00	#NULL!	#NULL!	#NULL!	#NULL!	#NULL!
2,00	2,00	#NULL!	#NULL!	#NULL!	#NULL!	#NULL!
2,00	2,00	#NULL!	#NULL!	#NULL!	#NULL!	#NULL!
2,00	2,00	#NULL!	#NULL!	#NULL!	#NULL!	#NULL!
2,00	2,00	#NULL!	#NULL!	#NULL!	#NULL!	#NULL!
2,00	2,00	#NULL!	#NULL!	#NULL!	#NULL!	#NULL!
2,00	2,00	#NULL!	#NULL!	#NULL!	#NULL!	#NULL!
2,00	2,00	#NULL!	#NULL!	#NULL!	#NULL!	#NULL!
2,00	2,00	#NULL!	#NULL!	#NULL!	#NULL!	#NULL!
2,00	2,00	#NULL!	#NULL!	#NULL!	#NULL!	#NULL!
2,00	2,00	#NULL!	#NULL!	#NULL!	#NULL!	#NULL!
2,00	2,00	#NULL!	#NULL!	#NULL!	#NULL!	#NULL!
2,00	2,00	#NULL!	#NULL!	#NULL!	#NULL!	#NULL!
2,00	2,00	#NULL!	#NULL!	#NULL!	#NULL!	#NULL!
2,00	2,00	#NULL!	#NULL!	#NULL!	#NULL!	#NULL!
2,00	2,00	#NULL!	#NULL!	#NULL!	#NULL!	#NULL!
2,00	2,00	#NULL!	#NULL!	#NULL!	#NULL!	#NULL!
2,00	2,00	#NULL!	#NULL!	#NULL!	#NULL!	#NULL!
2,00	2,00	#NULL!	#NULL!	#NULL!	#NULL!	#NULL!
2,00	2,00	#NULL!	#NULL!	#NULL!	#NULL!	#NULL!
2,00	2,00	#NULL!	#NULL!	#NULL!	#NULL!	#NULL!
2,00	2,00	#NULL!	#NULL!	#NULL!	#NULL!	#NULL!
2,00	2,00	#NULL!	#NULL!	#NULL!	#NULL!	#NULL!
2,00	2,00	#NULL!	#NULL!	#NULL!	#NULL!	#NULL!
2,00	2,00	#NULL!	#NULL!	#NULL!	#NULL!	#NULL!
1,00	2,00	#NULL!	#NULL!	#NULL!	#NULL!	#NULL!
1,00	2,00	#NULL!	#NULL!	#NULL!	#NULL!	#NULL!
2,00	2,00	#NULL!	#NULL!	#NULL!	#NULL!	#NULL!
2,00	2,00	#NULL!	#NULL!	#NULL!	#NULL!	#NULL!
1,00	2,00	#NULL!	#NULL!	#NULL!	#NULL!	#NULL!
2,00	2,00	#NULL!	#NULL!	#NULL!	#NULL!	#NULL!
2,00	2,00	#NULL!	#NULL!	#NULL!	#NULL!	#NULL!
2,00	2,00	#NULL!	#NULL!	#NULL!	#NULL!	#NULL!
1,00	2,00	#NULL!	#NULL!	#NULL!	#NULL!	#NULL!
2,00	2,00	#NULL!	#NULL!	#NULL!	#NULL!	#NULL!
2,00	2,00	#NULL!	#NULL!	#NULL!	#NULL!	#NULL!
2,00	2,00	#NULL!	#NULL!	#NULL!	#NULL!	#NULL!
2,00	2,00	#NULL!	#NULL!	#NULL!	#NULL!	#NULL!
2,00	2,00	#NULL!	#NULL!	#NULL!	#NULL!	#NULL!
2,00	2,00	#NULL!	#NULL!	#NULL!	#NULL!	#NULL!
2,00	2,00	#NULL!	#NULL!	#NULL!	#NULL!	#NULL!
2,00	2,00	#NULL!	#NULL!	#NULL!	#NULL!	#NULL!
2,00	2,00	#NULL!	#NULL!	#NULL!	#NULL!	#NULL!
2,00	2,00	#NULL!	#NULL!	#NULL!	#NULL!	#NULL!
2,00	2,00	#NULL!	#NULL!	#NULL!	#NULL!	#NULL!
2,00	2,00	#NULL!	#NULL!	#NULL!	#NULL!	#NULL!
2,00	2,00	#NULL!	#NULL!	#NULL!	#NULL!	#NULL!
2,00	2,00	#NULL!	#NULL!	#NULL!	#NULL!	#NULL!
2,00	2,00	#NULL!	#NULL!	#NULL!	#NULL!	#NULL!
2,00	2,00	#NULL!	#NULL!	#NULL!	#NULL!	#NULL!

2,00	2,00	#NULL!	#NULL!	#NULL!	#NULL!	#NULL!
2,00	2,00	#NULL!	#NULL!	#NULL!	#NULL!	#NULL!
2,00	2,00	#NULL!	#NULL!	#NULL!	#NULL!	#NULL!
2,00	2,00	#NULL!	#NULL!	#NULL!	#NULL!	#NULL!
2,00	2,00	#NULL!	#NULL!	#NULL!	#NULL!	#NULL!
2,00	2,00	#NULL!	#NULL!	#NULL!	#NULL!	#NULL!
2,00	2,00	#NULL!	#NULL!	#NULL!	#NULL!	#NULL!
2,00	2,00	#NULL!	#NULL!	#NULL!	#NULL!	#NULL!
2,00	2,00	#NULL!	#NULL!	#NULL!	#NULL!	#NULL!
2,00	2,00	#NULL!	#NULL!	#NULL!	#NULL!	#NULL!
2,00	2,00	#NULL!	#NULL!	#NULL!	#NULL!	#NULL!
2,00	2,00	#NULL!	#NULL!	#NULL!	#NULL!	#NULL!
2,00	2,00	#NULL!	#NULL!	#NULL!	#NULL!	#NULL!
2,00	2,00	#NULL!	#NULL!	#NULL!	#NULL!	#NULL!
2,00	2,00	#NULL!	#NULL!	#NULL!	#NULL!	#NULL!
2,00	2,00	#NULL!	#NULL!	#NULL!	#NULL!	#NULL!
2,00	2,00	#NULL!	#NULL!	#NULL!	#NULL!	#NULL!
2,00	2,00	#NULL!	#NULL!	#NULL!	#NULL!	#NULL!
2,00	2,00	#NULL!	#NULL!	#NULL!	#NULL!	#NULL!
2,00	2,00	#NULL!	#NULL!	#NULL!	#NULL!	#NULL!
1,00	2,00	#NULL!	#NULL!	#NULL!	#NULL!	#NULL!
2,00	2,00	#NULL!	#NULL!	#NULL!	#NULL!	#NULL!
2,00	2,00	#NULL!	#NULL!	#NULL!	#NULL!	#NULL!
2,00	2,00	#NULL!	#NULL!	#NULL!	#NULL!	#NULL!
2,00	2,00	#NULL!	#NULL!	#NULL!	#NULL!	#NULL!
2,00	2,00	#NULL!	#NULL!	#NULL!	#NULL!	#NULL!
2,00	2,00	#NULL!	#NULL!	#NULL!	#NULL!	#NULL!
2,00	2,00	#NULL!	#NULL!	#NULL!	#NULL!	#NULL!
2,00	2,00	#NULL!	#NULL!	#NULL!	#NULL!	#NULL!
2,00	2,00	#NULL!	#NULL!	#NULL!	#NULL!	#NULL!
2,00	2,00	#NULL!	#NULL!	#NULL!	#NULL!	#NULL!
2,00	2,00	#NULL!	#NULL!	#NULL!	#NULL!	#NULL!
2,00	2,00	#NULL!	#NULL!	#NULL!	#NULL!	#NULL!
2,00	2,00	#NULL!	#NULL!	#NULL!	#NULL!	#NULL!
2,00	2,00	#NULL!	#NULL!	#NULL!	#NULL!	#NULL!
2,00	2,00	#NULL!	#NULL!	#NULL!	#NULL!	#NULL!
2,00	2,00	#NULL!	#NULL!	#NULL!	#NULL!	#NULL!
2,00	2,00	#NULL!	#NULL!	#NULL!	#NULL!	#NULL!
2,00	2,00	#NULL!	#NULL!	#NULL!	#NULL!	#NULL!
2,00	2,00	#NULL!	#NULL!	#NULL!	#NULL!	#NULL!
2,00	2,00	#NULL!	#NULL!	#NULL!	#NULL!	#NULL!
2,00	2,00	#NULL!	#NULL!	#NULL!	#NULL!	#NULL!
2,00	2,00	#NULL!	#NULL!	#NULL!	#NULL!	#NULL!
2,00	2,00	#NULL!	#NULL!	#NULL!	#NULL!	#NULL!
2,00	2,00	#NULL!	#NULL!	#NULL!	#NULL!	#NULL!
2,00	2,00	#NULL!	#NULL!	#NULL!	#NULL!	#NULL!
2,00	2,00	#NULL!	#NULL!	#NULL!	#NULL!	#NULL!
2,00	2,00	#NULL!	#NULL!	#NULL!	#NULL!	#NULL!
2,00	2,00	#NULL!	#NULL!	#NULL!	#NULL!	#NULL!
2,00	2,00	#NULL!	#NULL!	#NULL!	#NULL!	#NULL!

2,00	2,00	#NULL!	#NULL!	#NULL!	#NULL!	#NULL!
2,00	2,00	#NULL!	#NULL!	#NULL!	#NULL!	#NULL!
2,00	2,00	#NULL!	#NULL!	#NULL!	#NULL!	#NULL!
2,00	2,00	#NULL!	#NULL!	#NULL!	#NULL!	#NULL!
2,00	2,00	#NULL!	#NULL!	#NULL!	#NULL!	#NULL!
2,00	2,00	#NULL!	#NULL!	#NULL!	#NULL!	#NULL!
2,00	2,00	#NULL!	#NULL!	#NULL!	#NULL!	#NULL!
2,00	2,00	#NULL!	#NULL!	#NULL!	#NULL!	#NULL!
2,00	2,00	#NULL!	#NULL!	#NULL!	#NULL!	#NULL!
2,00	2,00	#NULL!	#NULL!	#NULL!	#NULL!	#NULL!
1,00	2,00	#NULL!	#NULL!	#NULL!	#NULL!	#NULL!
2,00	2,00	#NULL!	#NULL!	#NULL!	#NULL!	#NULL!
2,00	2,00	#NULL!	#NULL!	#NULL!	#NULL!	#NULL!
2,00	2,00	#NULL!	#NULL!	#NULL!	#NULL!	#NULL!
2,00	2,00	#NULL!	#NULL!	#NULL!	#NULL!	#NULL!
2,00	2,00	#NULL!	#NULL!	#NULL!	#NULL!	#NULL!
2,00	2,00	#NULL!	#NULL!	#NULL!	#NULL!	#NULL!
1,00	2,00	#NULL!	#NULL!	#NULL!	#NULL!	#NULL!
2,00	2,00	#NULL!	#NULL!	#NULL!	#NULL!	#NULL!
1,00	2,00	#NULL!	#NULL!	#NULL!	#NULL!	#NULL!
2,00	2,00	#NULL!	#NULL!	#NULL!	#NULL!	#NULL!
2,00	2,00	#NULL!	#NULL!	#NULL!	#NULL!	#NULL!
2,00	2,00	#NULL!	#NULL!	#NULL!	#NULL!	#NULL!
2,00	2,00	#NULL!	#NULL!	#NULL!	#NULL!	#NULL!
2,00	2,00	#NULL!	#NULL!	#NULL!	#NULL!	#NULL!
2,00	2,00	#NULL!	#NULL!	#NULL!	#NULL!	#NULL!
2,00	2,00	#NULL!	#NULL!	#NULL!	#NULL!	#NULL!
1,00	2,00	#NULL!	#NULL!	#NULL!	#NULL!	#NULL!
2,00	2,00	#NULL!	#NULL!	#NULL!	#NULL!	#NULL!
2,00	2,00	#NULL!	#NULL!	#NULL!	#NULL!	#NULL!
2,00	2,00	#NULL!	#NULL!	#NULL!	#NULL!	#NULL!
1,00	2,00	#NULL!	#NULL!	#NULL!	#NULL!	#NULL!
2,00	2,00	#NULL!	#NULL!	#NULL!	#NULL!	#NULL!
2,00	2,00	#NULL!	#NULL!	#NULL!	#NULL!	#NULL!
2,00	2,00	#NULL!	#NULL!	#NULL!	#NULL!	#NULL!
2,00	2,00	#NULL!	#NULL!	#NULL!	#NULL!	#NULL!
2,00	2,00	#NULL!	#NULL!	#NULL!	#NULL!	#NULL!
2,00	2,00	#NULL!	#NULL!	#NULL!	#NULL!	#NULL!
2,00	2,00	#NULL!	#NULL!	#NULL!	#NULL!	#NULL!
2,00	2,00	#NULL!	#NULL!	#NULL!	#NULL!	#NULL!
2,00	2,00	#NULL!	#NULL!	#NULL!	#NULL!	#NULL!
2,00	2,00	#NULL!	#NULL!	#NULL!	#NULL!	#NULL!
2,00	2,00	#NULL!	#NULL!	#NULL!	#NULL!	#NULL!
2,00	2,00	#NULL!	#NULL!	#NULL!	#NULL!	#NULL!
2,00	2,00	#NULL!	#NULL!	#NULL!	#NULL!	#NULL!
2,00	2,00	#NULL!	#NULL!	#NULL!	#NULL!	#NULL!
2,00	2,00	#NULL!	#NULL!	#NULL!	#NULL!	#NULL!
2,00	2,00	#NULL!	#NULL!	#NULL!	#NULL!	#NULL!
2,00	2,00	#NULL!	#NULL!	#NULL!	#NULL!	#NULL!
2,00	2,00	#NULL!	#NULL!	#NULL!	#NULL!	#NULL!

2,00	2,00	#NULL!	#NULL!	#NULL!	#NULL!	#NULL!
2,00	2,00	#NULL!	#NULL!	#NULL!	#NULL!	#NULL!
2,00	2,00	#NULL!	#NULL!	#NULL!	#NULL!	#NULL!
2,00	2,00	#NULL!	#NULL!	#NULL!	#NULL!	#NULL!
2,00	2,00	#NULL!	#NULL!	#NULL!	#NULL!	#NULL!
2,00	2,00	#NULL!	#NULL!	#NULL!	#NULL!	#NULL!
2,00	2,00	#NULL!	#NULL!	#NULL!	#NULL!	#NULL!
2,00	2,00	#NULL!	#NULL!	#NULL!	#NULL!	#NULL!
2,00	2,00	#NULL!	#NULL!	#NULL!	#NULL!	#NULL!
2,00	2,00	#NULL!	#NULL!	#NULL!	#NULL!	#NULL!
2,00	2,00	#NULL!	#NULL!	#NULL!	#NULL!	#NULL!
2,00	2,00	#NULL!	#NULL!	#NULL!	#NULL!	#NULL!
2,00	2,00	#NULL!	#NULL!	#NULL!	#NULL!	#NULL!
2,00	2,00	#NULL!	#NULL!	#NULL!	#NULL!	#NULL!
2,00	2,00	#NULL!	#NULL!	#NULL!	#NULL!	#NULL!
2,00	2,00	#NULL!	#NULL!	#NULL!	#NULL!	#NULL!
2,00	2,00	#NULL!	#NULL!	#NULL!	#NULL!	#NULL!
2,00	2,00	#NULL!	#NULL!	#NULL!	#NULL!	#NULL!
2,00	2,00	#NULL!	#NULL!	#NULL!	#NULL!	#NULL!
2,00	2,00	#NULL!	#NULL!	#NULL!	#NULL!	#NULL!
2,00	2,00	#NULL!	#NULL!	#NULL!	#NULL!	#NULL!
2,00	2,00	#NULL!	#NULL!	#NULL!	#NULL!	#NULL!
2,00	2,00	#NULL!	#NULL!	#NULL!	#NULL!	#NULL!
2,00	2,00	#NULL!	#NULL!	#NULL!	#NULL!	#NULL!
2,00	2,00	#NULL!	#NULL!	#NULL!	#NULL!	#NULL!
2,00	2,00	#NULL!	#NULL!	#NULL!	#NULL!	#NULL!
2,00	2,00	#NULL!	#NULL!	#NULL!	#NULL!	#NULL!
2,00	2,00	#NULL!	#NULL!	#NULL!	#NULL!	#NULL!
2,00	2,00	#NULL!	#NULL!	#NULL!	#NULL!	#NULL!
2,00	2,00	#NULL!	#NULL!	#NULL!	#NULL!	#NULL!
2,00	2,00	#NULL!	#NULL!	#NULL!	#NULL!	#NULL!
2,00	2,00	#NULL!	#NULL!	#NULL!	#NULL!	#NULL!
2,00	2,00	#NULL!	#NULL!	#NULL!	#NULL!	#NULL!
1,00	2,00	#NULL!	#NULL!	#NULL!	#NULL!	#NULL!
2,00	2,00	#NULL!	#NULL!	#NULL!	#NULL!	#NULL!
2,00	2,00	#NULL!	#NULL!	#NULL!	#NULL!	#NULL!
2,00	2,00	#NULL!	#NULL!	#NULL!	#NULL!	#NULL!
2,00	2,00	#NULL!	#NULL!	#NULL!	#NULL!	#NULL!
2,00	2,00	#NULL!	#NULL!	#NULL!	#NULL!	#NULL!
2,00	2,00	#NULL!	#NULL!	#NULL!	#NULL!	#NULL!
2,00	2,00	#NULL!	#NULL!	#NULL!	#NULL!	#NULL!
2,00	2,00	#NULL!	#NULL!	#NULL!	#NULL!	#NULL!
2,00	2,00	#NULL!	#NULL!	#NULL!	#NULL!	#NULL!
2,00	2,00	#NULL!	#NULL!	#NULL!	#NULL!	#NULL!
2,00	2,00	#NULL!	#NULL!	#NULL!	#NULL!	#NULL!
2,00	2,00	#NULL!	#NULL!	#NULL!	#NULL!	#NULL!
2,00	2,00	#NULL!	#NULL!	#NULL!	#NULL!	#NULL!
2,00	2,00	#NULL!	#NULL!	#NULL!	#NULL!	#NULL!
2,00	2,00	#NULL!	#NULL!	#NULL!	#NULL!	#NULL!
2,00	2,00	#NULL!	#NULL!	#NULL!	#NULL!	#NULL!

2,00	2,00	#NULL!	#NULL!	#NULL!	#NULL!	#NULL!
2,00	2,00	#NULL!	#NULL!	#NULL!	#NULL!	#NULL!
2,00	2,00	#NULL!	#NULL!	#NULL!	#NULL!	#NULL!
2,00	2,00	#NULL!	#NULL!	#NULL!	#NULL!	#NULL!
1,00	2,00	#NULL!	#NULL!	#NULL!	#NULL!	#NULL!
1,00	2,00	#NULL!	#NULL!	#NULL!	#NULL!	#NULL!
2,00	2,00	#NULL!	#NULL!	#NULL!	#NULL!	#NULL!
2,00	2,00	#NULL!	#NULL!	#NULL!	#NULL!	#NULL!
2,00	2,00	#NULL!	#NULL!	#NULL!	#NULL!	#NULL!
2,00	2,00	#NULL!	#NULL!	#NULL!	#NULL!	#NULL!
2,00	2,00	#NULL!	#NULL!	#NULL!	#NULL!	#NULL!
2,00	2,00	#NULL!	#NULL!	#NULL!	#NULL!	#NULL!
2,00	2,00	#NULL!	#NULL!	#NULL!	#NULL!	#NULL!
2,00	2,00	#NULL!	#NULL!	#NULL!	#NULL!	#NULL!
1,00	2,00	#NULL!	#NULL!	#NULL!	#NULL!	#NULL!
2,00	2,00	#NULL!	#NULL!	#NULL!	#NULL!	#NULL!
2,00	2,00	#NULL!	#NULL!	#NULL!	#NULL!	#NULL!
1,00	2,00	#NULL!	#NULL!	#NULL!	#NULL!	#NULL!
2,00	2,00	#NULL!	#NULL!	#NULL!	#NULL!	#NULL!
2,00	2,00	#NULL!	#NULL!	#NULL!	#NULL!	#NULL!
1,00	2,00	#NULL!	#NULL!	#NULL!	#NULL!	#NULL!
2,00	2,00	#NULL!	#NULL!	#NULL!	#NULL!	#NULL!
2,00	2,00	#NULL!	#NULL!	#NULL!	#NULL!	#NULL!
2,00	2,00	#NULL!	#NULL!	#NULL!	#NULL!	#NULL!
2,00	2,00	#NULL!	#NULL!	#NULL!	#NULL!	#NULL!
2,00	2,00	#NULL!	#NULL!	#NULL!	#NULL!	#NULL!
2,00	2,00	#NULL!	#NULL!	#NULL!	#NULL!	#NULL!
2,00	2,00	#NULL!	#NULL!	#NULL!	#NULL!	#NULL!
2,00	2,00	#NULL!	#NULL!	#NULL!	#NULL!	#NULL!
2,00	2,00	#NULL!	#NULL!	#NULL!	#NULL!	#NULL!
2,00	2,00	#NULL!	#NULL!	#NULL!	#NULL!	#NULL!
2,00	2,00	#NULL!	#NULL!	#NULL!	#NULL!	#NULL!
2,00	2,00	#NULL!	#NULL!	#NULL!	#NULL!	#NULL!
1,00	2,00	#NULL!	#NULL!	#NULL!	#NULL!	#NULL!
2,00	2,00	#NULL!	#NULL!	#NULL!	#NULL!	#NULL!
2,00	2,00	#NULL!	#NULL!	#NULL!	#NULL!	#NULL!
2,00	2,00	#NULL!	#NULL!	#NULL!	#NULL!	#NULL!
2,00	2,00	#NULL!	#NULL!	#NULL!	#NULL!	#NULL!
2,00	2,00	#NULL!	#NULL!	#NULL!	#NULL!	#NULL!
2,00	2,00	#NULL!	#NULL!	#NULL!	#NULL!	#NULL!
2,00	2,00	#NULL!	#NULL!	#NULL!	#NULL!	#NULL!
2,00	2,00	#NULL!	#NULL!	#NULL!	#NULL!	#NULL!
2,00	2,00	#NULL!	#NULL!	#NULL!	#NULL!	#NULL!
2,00	2,00	#NULL!	#NULL!	#NULL!	#NULL!	#NULL!
2,00	2,00	#NULL!	#NULL!	#NULL!	#NULL!	#NULL!
2,00	2,00	#NULL!	#NULL!	#NULL!	#NULL!	#NULL!
2,00	2,00	#NULL!	#NULL!	#NULL!	#NULL!	#NULL!
2,00	2,00	#NULL!	#NULL!	#NULL!	#NULL!	#NULL!
2,00	2,00	#NULL!	#NULL!	#NULL!	#NULL!	#NULL!
2,00	2,00	#NULL!	#NULL!	#NULL!	#NULL!	#NULL!

2,00	2,00	#NULL!	#NULL!	#NULL!	#NULL!	#NULL!
2,00	2,00	#NULL!	#NULL!	#NULL!	#NULL!	#NULL!
2,00	2,00	#NULL!	#NULL!	#NULL!	#NULL!	#NULL!
2,00	2,00	#NULL!	#NULL!	#NULL!	#NULL!	#NULL!
2,00	2,00	#NULL!	#NULL!	#NULL!	#NULL!	#NULL!
2,00	2,00	#NULL!	#NULL!	#NULL!	#NULL!	#NULL!
2,00	2,00	#NULL!	#NULL!	#NULL!	#NULL!	#NULL!
1,00	2,00	#NULL!	#NULL!	#NULL!	#NULL!	#NULL!
2,00	2,00	#NULL!	#NULL!	#NULL!	#NULL!	#NULL!
2,00	2,00	#NULL!	#NULL!	#NULL!	#NULL!	#NULL!
2,00	2,00	#NULL!	#NULL!	#NULL!	#NULL!	#NULL!
2,00	2,00	#NULL!	#NULL!	#NULL!	#NULL!	#NULL!
2,00	2,00	#NULL!	#NULL!	#NULL!	#NULL!	#NULL!
2,00	2,00	#NULL!	#NULL!	#NULL!	#NULL!	#NULL!
2,00	2,00	#NULL!	#NULL!	#NULL!	#NULL!	#NULL!
2,00	2,00	#NULL!	#NULL!	#NULL!	#NULL!	#NULL!
2,00	2,00	#NULL!	#NULL!	#NULL!	#NULL!	#NULL!
2,00	2,00	#NULL!	#NULL!	#NULL!	#NULL!	#NULL!
2,00	2,00	#NULL!	#NULL!	#NULL!	#NULL!	#NULL!
2,00	2,00	#NULL!	#NULL!	#NULL!	#NULL!	#NULL!
2,00	2,00	#NULL!	#NULL!	#NULL!	#NULL!	#NULL!
2,00	2,00	#NULL!	#NULL!	#NULL!	#NULL!	#NULL!
2,00	2,00	#NULL!	#NULL!	#NULL!	#NULL!	#NULL!
2,00	2,00	#NULL!	#NULL!	#NULL!	#NULL!	#NULL!
2,00	2,00	#NULL!	#NULL!	#NULL!	#NULL!	#NULL!
2,00	2,00	#NULL!	#NULL!	#NULL!	#NULL!	#NULL!
2,00	2,00	#NULL!	#NULL!	#NULL!	#NULL!	#NULL!
2,00	2,00	#NULL!	#NULL!	#NULL!	#NULL!	#NULL!
2,00	2,00	#NULL!	#NULL!	#NULL!	#NULL!	#NULL!
2,00	2,00	#NULL!	#NULL!	#NULL!	#NULL!	#NULL!
2,00	2,00	#NULL!	#NULL!	#NULL!	#NULL!	#NULL!
1,00	2,00	#NULL!	#NULL!	#NULL!	#NULL!	#NULL!
1,00	2,00	#NULL!	#NULL!	#NULL!	#NULL!	#NULL!
2,00	2,00	#NULL!	#NULL!	#NULL!	#NULL!	#NULL!
2,00	2,00	#NULL!	#NULL!	#NULL!	#NULL!	#NULL!
2,00	2,00	#NULL!	#NULL!	#NULL!	#NULL!	#NULL!
2,00	2,00	#NULL!	#NULL!	#NULL!	#NULL!	#NULL!
2,00	2,00	#NULL!	#NULL!	#NULL!	#NULL!	#NULL!
2,00	2,00	#NULL!	#NULL!	#NULL!	#NULL!	#NULL!
1,00	2,00	#NULL!	#NULL!	#NULL!	#NULL!	#NULL!
1,00	2,00	#NULL!	#NULL!	#NULL!	#NULL!	#NULL!
2,00	2,00	#NULL!	#NULL!	#NULL!	#NULL!	#NULL!
2,00	2,00	#NULL!	#NULL!	#NULL!	#NULL!	#NULL!
2,00	2,00	#NULL!	#NULL!	#NULL!	#NULL!	#NULL!
2,00	2,00	#NULL!	#NULL!	#NULL!	#NULL!	#NULL!
2,00	2,00	#NULL!	#NULL!	#NULL!	#NULL!	#NULL!
2,00	2,00	#NULL!	#NULL!	#NULL!	#NULL!	#NULL!
2,00	2,00	#NULL!	#NULL!	#NULL!	#NULL!	#NULL!
2,00	2,00	#NULL!	#NULL!	#NULL!	#NULL!	#NULL!
2,00	2,00	#NULL!	#NULL!	#NULL!	#NULL!	#NULL!

2,00	2,00	#NULL!	#NULL!	#NULL!	#NULL!	#NULL!
2,00	2,00	#NULL!	#NULL!	#NULL!	#NULL!	#NULL!
2,00	2,00	#NULL!	#NULL!	#NULL!	#NULL!	#NULL!
2,00	2,00	#NULL!	#NULL!	#NULL!	#NULL!	#NULL!
2,00	2,00	#NULL!	#NULL!	#NULL!	#NULL!	#NULL!
2,00	2,00	#NULL!	#NULL!	#NULL!	#NULL!	#NULL!
2,00	2,00	#NULL!	#NULL!	#NULL!	#NULL!	#NULL!
2,00	2,00	#NULL!	#NULL!	#NULL!	#NULL!	#NULL!
2,00	2,00	#NULL!	#NULL!	#NULL!	#NULL!	#NULL!
2,00	2,00	#NULL!	#NULL!	#NULL!	#NULL!	#NULL!
2,00	2,00	#NULL!	#NULL!	#NULL!	#NULL!	#NULL!
2,00	2,00	#NULL!	#NULL!	#NULL!	#NULL!	#NULL!
2,00	2,00	#NULL!	#NULL!	#NULL!	#NULL!	#NULL!
2,00	2,00	#NULL!	#NULL!	#NULL!	#NULL!	#NULL!
2,00	2,00	#NULL!	#NULL!	#NULL!	#NULL!	#NULL!
2,00	2,00	#NULL!	#NULL!	#NULL!	#NULL!	#NULL!
2,00	2,00	#NULL!	#NULL!	#NULL!	#NULL!	#NULL!
2,00	2,00	#NULL!	#NULL!	#NULL!	#NULL!	#NULL!
2,00	2,00	#NULL!	#NULL!	#NULL!	#NULL!	#NULL!
2,00	2,00	#NULL!	#NULL!	#NULL!	#NULL!	#NULL!
2,00	2,00	#NULL!	#NULL!	#NULL!	#NULL!	#NULL!
2,00	2,00	#NULL!	#NULL!	#NULL!	#NULL!	#NULL!
2,00	2,00	#NULL!	#NULL!	#NULL!	#NULL!	#NULL!
2,00	2,00	#NULL!	#NULL!	#NULL!	#NULL!	#NULL!
2,00	2,00	#NULL!	#NULL!	#NULL!	#NULL!	#NULL!
2,00	2,00	#NULL!	#NULL!	#NULL!	#NULL!	#NULL!
2,00	2,00	#NULL!	#NULL!	#NULL!	#NULL!	#NULL!
2,00	2,00	#NULL!	#NULL!	#NULL!	#NULL!	#NULL!
2,00	2,00	#NULL!	#NULL!	#NULL!	#NULL!	#NULL!
2,00	2,00	#NULL!	#NULL!	#NULL!	#NULL!	#NULL!
2,00	2,00	#NULL!	#NULL!	#NULL!	#NULL!	#NULL!
2,00	2,00	#NULL!	#NULL!	#NULL!	#NULL!	#NULL!
2,00	2,00	#NULL!	#NULL!	#NULL!	#NULL!	#NULL!
2,00	2,00	#NULL!	#NULL!	#NULL!	#NULL!	#NULL!
2,00	2,00	#NULL!	#NULL!	#NULL!	#NULL!	#NULL!
2,00	2,00	#NULL!	#NULL!	#NULL!	#NULL!	#NULL!
2,00	2,00	#NULL!	#NULL!	#NULL!	#NULL!	#NULL!
2,00	2,00	#NULL!	#NULL!	#NULL!	#NULL!	#NULL!
2,00	2,00	#NULL!	#NULL!	#NULL!	#NULL!	#NULL!
2,00	2,00	#NULL!	#NULL!	#NULL!	#NULL!	#NULL!
2,00	2,00	#NULL!	#NULL!	#NULL!	#NULL!	#NULL!
2,00	2,00	#NULL!	#NULL!	#NULL!	#NULL!	#NULL!
2,00	2,00	#NULL!	#NULL!	#NULL!	#NULL!	#NULL!
2,00	2,00	#NULL!	#NULL!	#NULL!	#NULL!	#NULL!
2,00	2,00	#NULL!	#NULL!	#NULL!	#NULL!	#NULL!
2,00	2,00	#NULL!	#NULL!	#NULL!	#NULL!	#NULL!
2,00	2,00	#NULL!	#NULL!	#NULL!	#NULL!	#NULL!
2,00	2,00	#NULL!	#NULL!	#NULL!	#NULL!	#NULL!
2,00	2,00	#NULL!	#NULL!	#NULL!	#NULL!	#NULL!
2,00	2,00	#NULL!	#NULL!	#NULL!	#NULL!	#NULL!

2,00	2,00	#NULL!	#NULL!	#NULL!	#NULL!	#NULL!
2,00	2,00	#NULL!	#NULL!	#NULL!	#NULL!	#NULL!
2,00	2,00	#NULL!	#NULL!	#NULL!	#NULL!	#NULL!
2,00	2,00	#NULL!	#NULL!	#NULL!	#NULL!	#NULL!
2,00	2,00	#NULL!	#NULL!	#NULL!	#NULL!	#NULL!
2,00	2,00	#NULL!	#NULL!	#NULL!	#NULL!	#NULL!
2,00	2,00	#NULL!	#NULL!	#NULL!	#NULL!	#NULL!
2,00	2,00	#NULL!	#NULL!	#NULL!	#NULL!	#NULL!
2,00	2,00	#NULL!	#NULL!	#NULL!	#NULL!	#NULL!
2,00	2,00	#NULL!	#NULL!	#NULL!	#NULL!	#NULL!
2,00	2,00	#NULL!	#NULL!	#NULL!	#NULL!	#NULL!
2,00	2,00	#NULL!	#NULL!	#NULL!	#NULL!	#NULL!
2,00	2,00	#NULL!	#NULL!	#NULL!	#NULL!	#NULL!
2,00	2,00	#NULL!	#NULL!	#NULL!	#NULL!	#NULL!
2,00	2,00	#NULL!	#NULL!	#NULL!	#NULL!	#NULL!
2,00	2,00	#NULL!	#NULL!	#NULL!	#NULL!	#NULL!
2,00	2,00	#NULL!	#NULL!	#NULL!	#NULL!	#NULL!
2,00	2,00	#NULL!	#NULL!	#NULL!	#NULL!	#NULL!
2,00	2,00	#NULL!	#NULL!	#NULL!	#NULL!	#NULL!
2,00	2,00	#NULL!	#NULL!	#NULL!	#NULL!	#NULL!
2,00	2,00	#NULL!	#NULL!	#NULL!	#NULL!	#NULL!
2,00	2,00	#NULL!	#NULL!	#NULL!	#NULL!	#NULL!
2,00	2,00	#NULL!	#NULL!	#NULL!	#NULL!	#NULL!
2,00	2,00	#NULL!	#NULL!	#NULL!	#NULL!	#NULL!
2,00	2,00	#NULL!	#NULL!	#NULL!	#NULL!	#NULL!
2,00	2,00	#NULL!	#NULL!	#NULL!	#NULL!	#NULL!
2,00	2,00	#NULL!	#NULL!	#NULL!	#NULL!	#NULL!
2,00	2,00	#NULL!	#NULL!	#NULL!	#NULL!	#NULL!
2,00	2,00	#NULL!	#NULL!	#NULL!	#NULL!	#NULL!
2,00	2,00	#NULL!	#NULL!	#NULL!	#NULL!	#NULL!
2,00	2,00	#NULL!	#NULL!	#NULL!	#NULL!	#NULL!
2,00	2,00	#NULL!	#NULL!	#NULL!	#NULL!	#NULL!
1,00	2,00	#NULL!	#NULL!	#NULL!	#NULL!	#NULL!
2,00	2,00	#NULL!	#NULL!	#NULL!	#NULL!	#NULL!
2,00	2,00	#NULL!	#NULL!	#NULL!	#NULL!	#NULL!
2,00	2,00	#NULL!	#NULL!	#NULL!	#NULL!	#NULL!
2,00	2,00	#NULL!	#NULL!	#NULL!	#NULL!	#NULL!
2,00	2,00	#NULL!	#NULL!	#NULL!	#NULL!	#NULL!
2,00	2,00	#NULL!	#NULL!	#NULL!	#NULL!	#NULL!
2,00	2,00	#NULL!	#NULL!	#NULL!	#NULL!	#NULL!
2,00	2,00	#NULL!	#NULL!	#NULL!	#NULL!	#NULL!
2,00	2,00	#NULL!	#NULL!	#NULL!	#NULL!	#NULL!
2,00	2,00	#NULL!	#NULL!	#NULL!	#NULL!	#NULL!
2,00	2,00	#NULL!	#NULL!	#NULL!	#NULL!	#NULL!
2,00	2,00	#NULL!	#NULL!	#NULL!	#NULL!	#NULL!
2,00	2,00	#NULL!	#NULL!	#NULL!	#NULL!	#NULL!
2,00	2,00	#NULL!	#NULL!	#NULL!	#NULL!	#NULL!
2,00	2,00	#NULL!	#NULL!	#NULL!	#NULL!	#NULL!
2,00	2,00	#NULL!	#NULL!	#NULL!	#NULL!	#NULL!
2,00	2,00	#NULL!	#NULL!	#NULL!	#NULL!	#NULL!

2,00	2,00	#NULL!	#NULL!	#NULL!	#NULL!	#NULL!
2,00	2,00	#NULL!	#NULL!	#NULL!	#NULL!	#NULL!
2,00	2,00	#NULL!	#NULL!	#NULL!	#NULL!	#NULL!
2,00	2,00	#NULL!	#NULL!	#NULL!	#NULL!	#NULL!
2,00	2,00	#NULL!	#NULL!	#NULL!	#NULL!	#NULL!
2,00	2,00	#NULL!	#NULL!	#NULL!	#NULL!	#NULL!
2,00	2,00	#NULL!	#NULL!	#NULL!	#NULL!	#NULL!
2,00	2,00	#NULL!	#NULL!	#NULL!	#NULL!	#NULL!
2,00	2,00	#NULL!	#NULL!	#NULL!	#NULL!	#NULL!
2,00	2,00	#NULL!	#NULL!	#NULL!	#NULL!	#NULL!
2,00	2,00	#NULL!	#NULL!	#NULL!	#NULL!	#NULL!
2,00	2,00	#NULL!	#NULL!	#NULL!	#NULL!	#NULL!
2,00	2,00	#NULL!	#NULL!	#NULL!	#NULL!	#NULL!
2,00	2,00	#NULL!	#NULL!	#NULL!	#NULL!	#NULL!
2,00	2,00	#NULL!	#NULL!	#NULL!	#NULL!	#NULL!
2,00	2,00	#NULL!	#NULL!	#NULL!	#NULL!	#NULL!
2,00	2,00	#NULL!	#NULL!	#NULL!	#NULL!	#NULL!
2,00	2,00	#NULL!	#NULL!	#NULL!	#NULL!	#NULL!
2,00	2,00	#NULL!	#NULL!	#NULL!	#NULL!	#NULL!
2,00	2,00	#NULL!	#NULL!	#NULL!	#NULL!	#NULL!
2,00	2,00	#NULL!	#NULL!	#NULL!	#NULL!	#NULL!
2,00	2,00	#NULL!	#NULL!	#NULL!	#NULL!	#NULL!
2,00	2,00	#NULL!	#NULL!	#NULL!	#NULL!	#NULL!
2,00	2,00	#NULL!	#NULL!	#NULL!	#NULL!	#NULL!
2,00	2,00	#NULL!	#NULL!	#NULL!	#NULL!	#NULL!
2,00	2,00	#NULL!	#NULL!	#NULL!	#NULL!	#NULL!
2,00	2,00	#NULL!	#NULL!	#NULL!	#NULL!	#NULL!
2,00	2,00	#NULL!	#NULL!	#NULL!	#NULL!	#NULL!
2,00	2,00	#NULL!	#NULL!	#NULL!	#NULL!	#NULL!
2,00	2,00	#NULL!	#NULL!	#NULL!	#NULL!	#NULL!
2,00	2,00	#NULL!	#NULL!	#NULL!	#NULL!	#NULL!
2,00	2,00	#NULL!	#NULL!	#NULL!	#NULL!	#NULL!
2,00	2,00	#NULL!	#NULL!	#NULL!	#NULL!	#NULL!
2,00	2,00	#NULL!	#NULL!	#NULL!	#NULL!	#NULL!
2,00	2,00	#NULL!	#NULL!	#NULL!	#NULL!	#NULL!
2,00	2,00	#NULL!	#NULL!	#NULL!	#NULL!	#NULL!
2,00	2,00	#NULL!	#NULL!	#NULL!	#NULL!	#NULL!
1,00	2,00	#NULL!	#NULL!	#NULL!	#NULL!	#NULL!
2,00	2,00	#NULL!	#NULL!	#NULL!	#NULL!	#NULL!
2,00	2,00	#NULL!	#NULL!	#NULL!	#NULL!	#NULL!
2,00	2,00	#NULL!	#NULL!	#NULL!	#NULL!	#NULL!
2,00	2,00	#NULL!	#NULL!	#NULL!	#NULL!	#NULL!
2,00	2,00	#NULL!	#NULL!	#NULL!	#NULL!	#NULL!
2,00	2,00	#NULL!	#NULL!	#NULL!	#NULL!	#NULL!
2,00	2,00	#NULL!	#NULL!	#NULL!	#NULL!	#NULL!
2,00	2,00	#NULL!	#NULL!	#NULL!	#NULL!	#NULL!
2,00	2,00	#NULL!	#NULL!	#NULL!	#NULL!	#NULL!
2,00	2,00	#NULL!	#NULL!	#NULL!	#NULL!	#NULL!
2,00	2,00	#NULL!	#NULL!	#NULL!	#NULL!	#NULL!
2,00	2,00	#NULL!	#NULL!	#NULL!	#NULL!	#NULL!

2,00	2,00	#NULL!	#NULL!	#NULL!	#NULL!	#NULL!
2,00	2,00	#NULL!	#NULL!	#NULL!	#NULL!	#NULL!
2,00	2,00	#NULL!	#NULL!	#NULL!	#NULL!	#NULL!
2,00	2,00	#NULL!	#NULL!	#NULL!	#NULL!	#NULL!
2,00	2,00	#NULL!	#NULL!	#NULL!	#NULL!	#NULL!
2,00	2,00	#NULL!	#NULL!	#NULL!	#NULL!	#NULL!
1,00	2,00	#NULL!	#NULL!	#NULL!	#NULL!	#NULL!
2,00	2,00	#NULL!	#NULL!	#NULL!	#NULL!	#NULL!
2,00	2,00	#NULL!	#NULL!	#NULL!	#NULL!	#NULL!
2,00	2,00	#NULL!	#NULL!	#NULL!	#NULL!	#NULL!
2,00	2,00	#NULL!	#NULL!	#NULL!	#NULL!	#NULL!
2,00	2,00	#NULL!	#NULL!	#NULL!	#NULL!	#NULL!
2,00	2,00	#NULL!	#NULL!	#NULL!	#NULL!	#NULL!
2,00	2,00	#NULL!	#NULL!	#NULL!	#NULL!	#NULL!
2,00	2,00	#NULL!	#NULL!	#NULL!	#NULL!	#NULL!
2,00	2,00	#NULL!	#NULL!	#NULL!	#NULL!	#NULL!
2,00	2,00	#NULL!	#NULL!	#NULL!	#NULL!	#NULL!
2,00	2,00	#NULL!	#NULL!	#NULL!	#NULL!	#NULL!
2,00	2,00	#NULL!	#NULL!	#NULL!	#NULL!	#NULL!
2,00	2,00	#NULL!	#NULL!	#NULL!	#NULL!	#NULL!
2,00	2,00	#NULL!	#NULL!	#NULL!	#NULL!	#NULL!
2,00	2,00	#NULL!	#NULL!	#NULL!	#NULL!	#NULL!
2,00	2,00	#NULL!	#NULL!	#NULL!	#NULL!	#NULL!
2,00	2,00	#NULL!	#NULL!	#NULL!	#NULL!	#NULL!
2,00	2,00	#NULL!	#NULL!	#NULL!	#NULL!	#NULL!
2,00	2,00	#NULL!	#NULL!	#NULL!	#NULL!	#NULL!
2,00	2,00	#NULL!	#NULL!	#NULL!	#NULL!	#NULL!
2,00	2,00	#NULL!	#NULL!	#NULL!	#NULL!	#NULL!
2,00	2,00	#NULL!	#NULL!	#NULL!	#NULL!	#NULL!
2,00	2,00	#NULL!	#NULL!	#NULL!	#NULL!	#NULL!
2,00	2,00	#NULL!	#NULL!	#NULL!	#NULL!	#NULL!
2,00	2,00	#NULL!	#NULL!	#NULL!	#NULL!	#NULL!
2,00	2,00	#NULL!	#NULL!	#NULL!	#NULL!	#NULL!
2,00	2,00	#NULL!	#NULL!	#NULL!	#NULL!	#NULL!
2,00	2,00	#NULL!	#NULL!	#NULL!	#NULL!	#NULL!
2,00	2,00	#NULL!	#NULL!	#NULL!	#NULL!	#NULL!
2,00	2,00	#NULL!	#NULL!	#NULL!	#NULL!	#NULL!
2,00	2,00	#NULL!	#NULL!	#NULL!	#NULL!	#NULL!
2,00	2,00	#NULL!	#NULL!	#NULL!	#NULL!	#NULL!
2,00	2,00	#NULL!	#NULL!	#NULL!	#NULL!	#NULL!
2,00	2,00	#NULL!	#NULL!	#NULL!	#NULL!	#NULL!
2,00	2,00	#NULL!	#NULL!	#NULL!	#NULL!	#NULL!
2,00	2,00	#NULL!	#NULL!	#NULL!	#NULL!	#NULL!
2,00	2,00	#NULL!	#NULL!	#NULL!	#NULL!	#NULL!
2,00	2,00	#NULL!	#NULL!	#NULL!	#NULL!	#NULL!
2,00	2,00	#NULL!	#NULL!	#NULL!	#NULL!	#NULL!
2,00	2,00	#NULL!	#NULL!	#NULL!	#NULL!	#NULL!
2,00	2,00	#NULL!	#NULL!	#NULL!	#NULL!	#NULL!
2,00	2,00	#NULL!	#NULL!	#NULL!	#NULL!	#NULL!
2,00	2,00	#NULL!	#NULL!	#NULL!	#NULL!	#NULL!

2,00	2,00	#NULL!	#NULL!	#NULL!	#NULL!	#NULL!
2,00	2,00	#NULL!	#NULL!	#NULL!	#NULL!	#NULL!
2,00	2,00	#NULL!	#NULL!	#NULL!	#NULL!	#NULL!
2,00	2,00	#NULL!	#NULL!	#NULL!	#NULL!	#NULL!
2,00	2,00	#NULL!	#NULL!	#NULL!	#NULL!	#NULL!
2,00	2,00	#NULL!	#NULL!	#NULL!	#NULL!	#NULL!
2,00	2,00	#NULL!	#NULL!	#NULL!	#NULL!	#NULL!
2,00	2,00	#NULL!	#NULL!	#NULL!	#NULL!	#NULL!
2,00	2,00	#NULL!	#NULL!	#NULL!	#NULL!	#NULL!
2,00	2,00	#NULL!	#NULL!	#NULL!	#NULL!	#NULL!
2,00	2,00	#NULL!	#NULL!	#NULL!	#NULL!	#NULL!
2,00	2,00	#NULL!	#NULL!	#NULL!	#NULL!	#NULL!
2,00	2,00	#NULL!	#NULL!	#NULL!	#NULL!	#NULL!
2,00	2,00	#NULL!	#NULL!	#NULL!	#NULL!	#NULL!
2,00	2,00	#NULL!	#NULL!	#NULL!	#NULL!	#NULL!
2,00	2,00	#NULL!	#NULL!	#NULL!	#NULL!	#NULL!
2,00	2,00	#NULL!	#NULL!	#NULL!	#NULL!	#NULL!
2,00	2,00	#NULL!	#NULL!	#NULL!	#NULL!	#NULL!
2,00	2,00	#NULL!	#NULL!	#NULL!	#NULL!	#NULL!
2,00	2,00	#NULL!	#NULL!	#NULL!	#NULL!	#NULL!
2,00	2,00	#NULL!	#NULL!	#NULL!	#NULL!	#NULL!
2,00	2,00	#NULL!	#NULL!	#NULL!	#NULL!	#NULL!
2,00	2,00	#NULL!	#NULL!	#NULL!	#NULL!	#NULL!
2,00	2,00	#NULL!	#NULL!	#NULL!	#NULL!	#NULL!
2,00	2,00	#NULL!	#NULL!	#NULL!	#NULL!	#NULL!
2,00	2,00	#NULL!	#NULL!	#NULL!	#NULL!	#NULL!
1,00	2,00	#NULL!	#NULL!	#NULL!	#NULL!	#NULL!
2,00	2,00	#NULL!	#NULL!	#NULL!	#NULL!	#NULL!
2,00	2,00	#NULL!	#NULL!	#NULL!	#NULL!	#NULL!
2,00	2,00	#NULL!	#NULL!	#NULL!	#NULL!	#NULL!
2,00	2,00	#NULL!	#NULL!	#NULL!	#NULL!	#NULL!
2,00	2,00	#NULL!	#NULL!	#NULL!	#NULL!	#NULL!
2,00	2,00	#NULL!	#NULL!	#NULL!	#NULL!	#NULL!
1,00	2,00	#NULL!	#NULL!	#NULL!	#NULL!	#NULL!
1,00	2,00	#NULL!	#NULL!	#NULL!	#NULL!	#NULL!
2,00	2,00	#NULL!	#NULL!	#NULL!	#NULL!	#NULL!
2,00	2,00	#NULL!	#NULL!	#NULL!	#NULL!	#NULL!
2,00	2,00	#NULL!	#NULL!	#NULL!	#NULL!	#NULL!
2,00	2,00	#NULL!	#NULL!	#NULL!	#NULL!	#NULL!
2,00	2,00	#NULL!	#NULL!	#NULL!	#NULL!	#NULL!
2,00	2,00	#NULL!	#NULL!	#NULL!	#NULL!	#NULL!
2,00	2,00	#NULL!	#NULL!	#NULL!	#NULL!	#NULL!
2,00	2,00	#NULL!	#NULL!	#NULL!	#NULL!	#NULL!
2,00	2,00	#NULL!	#NULL!	#NULL!	#NULL!	#NULL!
2,00	2,00	#NULL!	#NULL!	#NULL!	#NULL!	#NULL!
2,00	2,00	#NULL!	#NULL!	#NULL!	#NULL!	#NULL!
2,00	2,00	#NULL!	#NULL!	#NULL!	#NULL!	#NULL!
2,00	2,00	#NULL!	#NULL!	#NULL!	#NULL!	#NULL!
2,00	2,00	#NULL!	#NULL!	#NULL!	#NULL!	#NULL!
2,00	2,00	#NULL!	#NULL!	#NULL!	#NULL!	#NULL!

2,00	2,00	#NULL!	#NULL!	#NULL!	#NULL!	#NULL!
2,00	2,00	#NULL!	#NULL!	#NULL!	#NULL!	#NULL!
2,00	2,00	#NULL!	#NULL!	#NULL!	#NULL!	#NULL!
2,00	2,00	#NULL!	#NULL!	#NULL!	#NULL!	#NULL!
1,00	2,00	#NULL!	#NULL!	#NULL!	#NULL!	#NULL!
2,00	2,00	#NULL!	#NULL!	#NULL!	#NULL!	#NULL!
2,00	2,00	#NULL!	#NULL!	#NULL!	#NULL!	#NULL!
2,00	2,00	#NULL!	#NULL!	#NULL!	#NULL!	#NULL!
2,00	2,00	#NULL!	#NULL!	#NULL!	#NULL!	#NULL!
2,00	2,00	#NULL!	#NULL!	#NULL!	#NULL!	#NULL!
2,00	2,00	#NULL!	#NULL!	#NULL!	#NULL!	#NULL!
1,00	2,00	#NULL!	#NULL!	#NULL!	#NULL!	#NULL!
2,00	2,00	#NULL!	#NULL!	#NULL!	#NULL!	#NULL!
2,00	2,00	#NULL!	#NULL!	#NULL!	#NULL!	#NULL!
2,00	2,00	#NULL!	#NULL!	#NULL!	#NULL!	#NULL!
2,00	2,00	#NULL!	#NULL!	#NULL!	#NULL!	#NULL!
1,00	2,00	#NULL!	#NULL!	#NULL!	#NULL!	#NULL!
2,00	2,00	#NULL!	#NULL!	#NULL!	#NULL!	#NULL!
2,00	2,00	#NULL!	#NULL!	#NULL!	#NULL!	#NULL!
1,00	2,00	#NULL!	#NULL!	#NULL!	#NULL!	#NULL!
2,00	2,00	#NULL!	#NULL!	#NULL!	#NULL!	#NULL!
2,00	2,00	#NULL!	#NULL!	#NULL!	#NULL!	#NULL!
2,00	2,00	#NULL!	#NULL!	#NULL!	#NULL!	#NULL!
2,00	2,00	#NULL!	#NULL!	#NULL!	#NULL!	#NULL!
2,00	2,00	#NULL!	#NULL!	#NULL!	#NULL!	#NULL!
2,00	2,00	#NULL!	#NULL!	#NULL!	#NULL!	#NULL!
2,00	2,00	#NULL!	#NULL!	#NULL!	#NULL!	#NULL!
2,00	2,00	#NULL!	#NULL!	#NULL!	#NULL!	#NULL!
2,00	2,00	#NULL!	#NULL!	#NULL!	#NULL!	#NULL!
2,00	2,00	#NULL!	#NULL!	#NULL!	#NULL!	#NULL!
2,00	2,00	#NULL!	#NULL!	#NULL!	#NULL!	#NULL!
1,00	2,00	#NULL!	#NULL!	#NULL!	#NULL!	#NULL!
2,00	2,00	#NULL!	#NULL!	#NULL!	#NULL!	#NULL!
2,00	2,00	#NULL!	#NULL!	#NULL!	#NULL!	#NULL!
2,00	2,00	#NULL!	#NULL!	#NULL!	#NULL!	#NULL!
2,00	2,00	#NULL!	#NULL!	#NULL!	#NULL!	#NULL!
2,00	2,00	#NULL!	#NULL!	#NULL!	#NULL!	#NULL!
2,00	2,00	#NULL!	#NULL!	#NULL!	#NULL!	#NULL!
2,00	2,00	#NULL!	#NULL!	#NULL!	#NULL!	#NULL!
2,00	2,00	#NULL!	#NULL!	#NULL!	#NULL!	#NULL!
2,00	2,00	#NULL!	#NULL!	#NULL!	#NULL!	#NULL!
2,00	2,00	#NULL!	#NULL!	#NULL!	#NULL!	#NULL!
2,00	2,00	#NULL!	#NULL!	#NULL!	#NULL!	#NULL!
2,00	2,00	#NULL!	#NULL!	#NULL!	#NULL!	#NULL!
2,00	2,00	#NULL!	#NULL!	#NULL!	#NULL!	#NULL!
2,00	2,00	#NULL!	#NULL!	#NULL!	#NULL!	#NULL!
2,00	2,00	#NULL!	#NULL!	#NULL!	#NULL!	#NULL!
2,00	2,00	#NULL!	#NULL!	#NULL!	#NULL!	#NULL!
2,00	2,00	#NULL!	#NULL!	#NULL!	#NULL!	#NULL!
2,00	2,00	#NULL!	#NULL!	#NULL!	#NULL!	#NULL!

2,00	2,00	#NULL!	#NULL!	#NULL!	#NULL!	#NULL!
2,00	2,00	#NULL!	#NULL!	#NULL!	#NULL!	#NULL!
2,00	2,00	#NULL!	#NULL!	#NULL!	#NULL!	#NULL!
2,00	2,00	#NULL!	#NULL!	#NULL!	#NULL!	#NULL!
2,00	2,00	#NULL!	#NULL!	#NULL!	#NULL!	#NULL!
2,00	2,00	#NULL!	#NULL!	#NULL!	#NULL!	#NULL!
2,00	2,00	#NULL!	#NULL!	#NULL!	#NULL!	#NULL!
2,00	2,00	#NULL!	#NULL!	#NULL!	#NULL!	#NULL!
2,00	2,00	#NULL!	#NULL!	#NULL!	#NULL!	#NULL!
2,00	2,00	#NULL!	#NULL!	#NULL!	#NULL!	#NULL!
2,00	2,00	#NULL!	#NULL!	#NULL!	#NULL!	#NULL!
2,00	2,00	#NULL!	#NULL!	#NULL!	#NULL!	#NULL!
2,00	2,00	#NULL!	#NULL!	#NULL!	#NULL!	#NULL!
2,00	2,00	#NULL!	#NULL!	#NULL!	#NULL!	#NULL!
2,00	2,00	#NULL!	#NULL!	#NULL!	#NULL!	#NULL!
2,00	2,00	#NULL!	#NULL!	#NULL!	#NULL!	#NULL!
2,00	2,00	#NULL!	#NULL!	#NULL!	#NULL!	#NULL!
2,00	2,00	#NULL!	#NULL!	#NULL!	#NULL!	#NULL!
2,00	2,00	#NULL!	#NULL!	#NULL!	#NULL!	#NULL!
2,00	2,00	#NULL!	#NULL!	#NULL!	#NULL!	#NULL!
2,00	2,00	#NULL!	#NULL!	#NULL!	#NULL!	#NULL!
2,00	2,00	#NULL!	#NULL!	#NULL!	#NULL!	#NULL!
2,00	2,00	#NULL!	#NULL!	#NULL!	#NULL!	#NULL!
2,00	2,00	#NULL!	#NULL!	#NULL!	#NULL!	#NULL!
2,00	2,00	#NULL!	#NULL!	#NULL!	#NULL!	#NULL!
2,00	2,00	#NULL!	#NULL!	#NULL!	#NULL!	#NULL!
2,00	2,00	#NULL!	#NULL!	#NULL!	#NULL!	#NULL!
2,00	2,00	#NULL!	#NULL!	#NULL!	#NULL!	#NULL!
2,00	2,00	#NULL!	#NULL!	#NULL!	#NULL!	#NULL!
2,00	2,00	#NULL!	#NULL!	#NULL!	#NULL!	#NULL!
2,00	2,00	#NULL!	#NULL!	#NULL!	#NULL!	#NULL!
2,00	2,00	#NULL!	#NULL!	#NULL!	#NULL!	#NULL!
2,00	2,00	#NULL!	#NULL!	#NULL!	#NULL!	#NULL!
2,00	2,00	#NULL!	#NULL!	#NULL!	#NULL!	#NULL!
2,00	2,00	#NULL!	#NULL!	#NULL!	#NULL!	#NULL!
2,00	2,00	#NULL!	#NULL!	#NULL!	#NULL!	#NULL!
2,00	2,00	#NULL!	#NULL!	#NULL!	#NULL!	#NULL!
2,00	2,00	#NULL!	#NULL!	#NULL!	#NULL!	#NULL!
2,00	2,00	#NULL!	#NULL!	#NULL!	#NULL!	#NULL!
2,00	2,00	#NULL!	#NULL!	#NULL!	#NULL!	#NULL!
2,00	2,00	#NULL!	#NULL!	#NULL!	#NULL!	#NULL!
2,00	2,00	#NULL!	#NULL!	#NULL!	#NULL!	#NULL!
2,00	2,00	#NULL!	#NULL!	#NULL!	#NULL!	#NULL!
2,00	2,00	#NULL!	#NULL!	#NULL!	#NULL!	#NULL!
2,00	2,00	#NULL!	#NULL!	#NULL!	#NULL!	#NULL!
2,00	2,00	#NULL!	#NULL!	#NULL!	#NULL!	#NULL!
2,00	2,00	#NULL!	#NULL!	#NULL!	#NULL!	#NULL!
2,00	2,00	#NULL!	#NULL!	#NULL!	#NULL!	#NULL!
2,00	2,00	#NULL!	#NULL!	#NULL!	#NULL!	#NULL!
2,00	2,00	#NULL!	#NULL!	#NULL!	#NULL!	#NULL!

2,00	2,00	#NULL!	#NULL!	#NULL!	#NULL!	#NULL!
2,00	2,00	#NULL!	#NULL!	#NULL!	#NULL!	#NULL!
2,00	2,00	#NULL!	#NULL!	#NULL!	#NULL!	#NULL!
2,00	2,00	#NULL!	#NULL!	#NULL!	#NULL!	#NULL!
2,00	2,00	#NULL!	#NULL!	#NULL!	#NULL!	#NULL!
2,00	2,00	#NULL!	#NULL!	#NULL!	#NULL!	#NULL!
2,00	2,00	#NULL!	#NULL!	#NULL!	#NULL!	#NULL!
2,00	2,00	#NULL!	#NULL!	#NULL!	#NULL!	#NULL!
2,00	2,00	#NULL!	#NULL!	#NULL!	#NULL!	#NULL!
2,00	2,00	#NULL!	#NULL!	#NULL!	#NULL!	#NULL!
2,00	2,00	#NULL!	#NULL!	#NULL!	#NULL!	#NULL!
2,00	2,00	#NULL!	#NULL!	#NULL!	#NULL!	#NULL!
2,00	2,00	#NULL!	#NULL!	#NULL!	#NULL!	#NULL!
2,00	2,00	#NULL!	#NULL!	#NULL!	#NULL!	#NULL!
2,00	2,00	#NULL!	#NULL!	#NULL!	#NULL!	#NULL!
2,00	2,00	#NULL!	#NULL!	#NULL!	#NULL!	#NULL!
2,00	2,00	#NULL!	#NULL!	#NULL!	#NULL!	#NULL!
2,00	2,00	#NULL!	#NULL!	#NULL!	#NULL!	#NULL!
2,00	2,00	#NULL!	#NULL!	#NULL!	#NULL!	#NULL!
2,00	2,00	#NULL!	#NULL!	#NULL!	#NULL!	#NULL!
2,00	2,00	#NULL!	#NULL!	#NULL!	#NULL!	#NULL!
2,00	2,00	#NULL!	#NULL!	#NULL!	#NULL!	#NULL!
2,00	2,00	#NULL!	#NULL!	#NULL!	#NULL!	#NULL!
2,00	2,00	#NULL!	#NULL!	#NULL!	#NULL!	#NULL!
2,00	2,00	#NULL!	#NULL!	#NULL!	#NULL!	#NULL!
2,00	2,00	#NULL!	#NULL!	#NULL!	#NULL!	#NULL!
2,00	2,00	#NULL!	#NULL!	#NULL!	#NULL!	#NULL!
2,00	2,00	#NULL!	#NULL!	#NULL!	#NULL!	#NULL!
2,00	2,00	#NULL!	#NULL!	#NULL!	#NULL!	#NULL!
2,00	2,00	#NULL!	#NULL!	#NULL!	#NULL!	#NULL!
2,00	2,00	#NULL!	#NULL!	#NULL!	#NULL!	#NULL!
2,00	2,00	#NULL!	#NULL!	#NULL!	#NULL!	#NULL!
2,00	2,00	#NULL!	#NULL!	#NULL!	#NULL!	#NULL!
2,00	2,00	#NULL!	#NULL!	#NULL!	#NULL!	#NULL!
2,00	2,00	#NULL!	#NULL!	#NULL!	#NULL!	#NULL!
2,00	2,00	#NULL!	#NULL!	#NULL!	#NULL!	#NULL!
2,00	2,00	#NULL!	#NULL!	#NULL!	#NULL!	#NULL!
2,00	2,00	#NULL!	#NULL!	#NULL!	#NULL!	#NULL!
2,00	2,00	#NULL!	#NULL!	#NULL!	#NULL!	#NULL!
2,00	2,00	#NULL!	#NULL!	#NULL!	#NULL!	#NULL!
2,00	2,00	#NULL!	#NULL!	#NULL!	#NULL!	#NULL!
2,00	2,00	#NULL!	#NULL!	#NULL!	#NULL!	#NULL!
2,00	2,00	#NULL!	#NULL!	#NULL!	#NULL!	#NULL!
2,00	2,00	#NULL!	#NULL!	#NULL!	#NULL!	#NULL!
2,00	2,00	#NULL!	#NULL!	#NULL!	#NULL!	#NULL!
2,00	2,00	#NULL!	#NULL!	#NULL!	#NULL!	#NULL!
2,00	2,00	#NULL!	#NULL!	#NULL!	#NULL!	#NULL!
2,00	2,00	#NULL!	#NULL!	#NULL!	#NULL!	#NULL!
2,00	2,00	#NULL!	#NULL!	#NULL!	#NULL!	#NULL!
2,00	2,00	#NULL!	#NULL!	#NULL!	#NULL!	#NULL!

2,00	2,00	#NULL!	#NULL!	#NULL!	#NULL!	#NULL!
2,00	2,00	#NULL!	#NULL!	#NULL!	#NULL!	#NULL!
2,00	2,00	#NULL!	#NULL!	#NULL!	#NULL!	#NULL!
2,00	2,00	#NULL!	#NULL!	#NULL!	#NULL!	#NULL!
2,00	2,00	#NULL!	#NULL!	#NULL!	#NULL!	#NULL!
2,00	2,00	#NULL!	#NULL!	#NULL!	#NULL!	#NULL!
2,00	2,00	#NULL!	#NULL!	#NULL!	#NULL!	#NULL!
2,00	2,00	#NULL!	#NULL!	#NULL!	#NULL!	#NULL!
2,00	2,00	#NULL!	#NULL!	#NULL!	#NULL!	#NULL!
2,00	2,00	#NULL!	#NULL!	#NULL!	#NULL!	#NULL!
2,00	2,00	#NULL!	#NULL!	#NULL!	#NULL!	#NULL!
2,00	2,00	#NULL!	#NULL!	#NULL!	#NULL!	#NULL!
2,00	2,00	#NULL!	#NULL!	#NULL!	#NULL!	#NULL!
2,00	2,00	#NULL!	#NULL!	#NULL!	#NULL!	#NULL!
2,00	2,00	#NULL!	#NULL!	#NULL!	#NULL!	#NULL!
2,00	2,00	#NULL!	#NULL!	#NULL!	#NULL!	#NULL!
2,00	2,00	#NULL!	#NULL!	#NULL!	#NULL!	#NULL!
2,00	2,00	#NULL!	#NULL!	#NULL!	#NULL!	#NULL!
2,00	2,00	#NULL!	#NULL!	#NULL!	#NULL!	#NULL!
2,00	2,00	#NULL!	#NULL!	#NULL!	#NULL!	#NULL!
2,00	2,00	#NULL!	#NULL!	#NULL!	#NULL!	#NULL!
2,00	2,00	#NULL!	#NULL!	#NULL!	#NULL!	#NULL!
2,00	2,00	#NULL!	#NULL!	#NULL!	#NULL!	#NULL!
2,00	2,00	#NULL!	#NULL!	#NULL!	#NULL!	#NULL!
2,00	2,00	#NULL!	#NULL!	#NULL!	#NULL!	#NULL!
1,00	2,00	#NULL!	#NULL!	#NULL!	#NULL!	#NULL!
2,00	2,00	#NULL!	#NULL!	#NULL!	#NULL!	#NULL!
2,00	2,00	#NULL!	#NULL!	#NULL!	#NULL!	#NULL!
2,00	2,00	#NULL!	#NULL!	#NULL!	#NULL!	#NULL!
2,00	2,00	#NULL!	#NULL!	#NULL!	#NULL!	#NULL!
1,00	2,00	#NULL!	#NULL!	#NULL!	#NULL!	#NULL!
2,00	2,00	#NULL!	#NULL!	#NULL!	#NULL!	#NULL!
2,00	2,00	#NULL!	#NULL!	#NULL!	#NULL!	#NULL!
2,00	2,00	#NULL!	#NULL!	#NULL!	#NULL!	#NULL!
2,00	2,00	#NULL!	#NULL!	#NULL!	#NULL!	#NULL!
2,00	2,00	#NULL!	#NULL!	#NULL!	#NULL!	#NULL!
2,00	2,00	#NULL!	#NULL!	#NULL!	#NULL!	#NULL!
2,00	2,00	#NULL!	#NULL!	#NULL!	#NULL!	#NULL!
2,00	2,00	#NULL!	#NULL!	#NULL!	#NULL!	#NULL!
1,00	2,00	#NULL!	#NULL!	#NULL!	#NULL!	#NULL!
2,00	2,00	#NULL!	#NULL!	#NULL!	#NULL!	#NULL!
2,00	2,00	#NULL!	#NULL!	#NULL!	#NULL!	#NULL!
2,00	2,00	#NULL!	#NULL!	#NULL!	#NULL!	#NULL!
2,00	2,00	#NULL!	#NULL!	#NULL!	#NULL!	#NULL!
1,00	2,00	#NULL!	#NULL!	#NULL!	#NULL!	#NULL!
2,00	2,00	#NULL!	#NULL!	#NULL!	#NULL!	#NULL!
2,00	2,00	#NULL!	#NULL!	#NULL!	#NULL!	#NULL!
2,00	2,00	#NULL!	#NULL!	#NULL!	#NULL!	#NULL!
2,00	2,00	#NULL!	#NULL!	#NULL!	#NULL!	#NULL!
2,00	2,00	#NULL!	#NULL!	#NULL!	#NULL!	#NULL!

2,00	2,00	#NULL!	#NULL!	#NULL!	#NULL!	#NULL!
2,00	2,00	#NULL!	#NULL!	#NULL!	#NULL!	#NULL!
2,00	2,00	#NULL!	#NULL!	#NULL!	#NULL!	#NULL!
1,00	2,00	#NULL!	#NULL!	#NULL!	#NULL!	#NULL!
2,00	2,00	#NULL!	#NULL!	#NULL!	#NULL!	#NULL!
2,00	2,00	#NULL!	#NULL!	#NULL!	#NULL!	#NULL!
2,00	2,00	#NULL!	#NULL!	#NULL!	#NULL!	#NULL!
2,00	2,00	#NULL!	#NULL!	#NULL!	#NULL!	#NULL!
2,00	2,00	#NULL!	#NULL!	#NULL!	#NULL!	#NULL!
2,00	2,00	#NULL!	#NULL!	#NULL!	#NULL!	#NULL!
2,00	2,00	#NULL!	#NULL!	#NULL!	#NULL!	#NULL!
2,00	2,00	#NULL!	#NULL!	#NULL!	#NULL!	#NULL!
2,00	2,00	#NULL!	#NULL!	#NULL!	#NULL!	#NULL!
2,00	2,00	#NULL!	#NULL!	#NULL!	#NULL!	#NULL!
2,00	2,00	#NULL!	#NULL!	#NULL!	#NULL!	#NULL!
2,00	2,00	#NULL!	#NULL!	#NULL!	#NULL!	#NULL!
2,00	2,00	#NULL!	#NULL!	#NULL!	#NULL!	#NULL!
2,00	2,00	#NULL!	#NULL!	#NULL!	#NULL!	#NULL!
2,00	2,00	#NULL!	#NULL!	#NULL!	#NULL!	#NULL!
2,00	2,00	#NULL!	#NULL!	#NULL!	#NULL!	#NULL!
2,00	2,00	#NULL!	#NULL!	#NULL!	#NULL!	#NULL!
2,00	2,00	#NULL!	#NULL!	#NULL!	#NULL!	#NULL!
2,00	2,00	#NULL!	#NULL!	#NULL!	#NULL!	#NULL!
2,00	2,00	#NULL!	#NULL!	#NULL!	#NULL!	#NULL!
2,00	2,00	#NULL!	#NULL!	#NULL!	#NULL!	#NULL!
2,00	2,00	#NULL!	#NULL!	#NULL!	#NULL!	#NULL!
2,00	2,00	#NULL!	#NULL!	#NULL!	#NULL!	#NULL!
2,00	2,00	#NULL!	#NULL!	#NULL!	#NULL!	#NULL!
2,00	2,00	#NULL!	#NULL!	#NULL!	#NULL!	#NULL!
2,00	2,00	#NULL!	#NULL!	#NULL!	#NULL!	#NULL!
2,00	2,00	#NULL!	#NULL!	#NULL!	#NULL!	#NULL!
2,00	2,00	#NULL!	#NULL!	#NULL!	#NULL!	#NULL!
2,00	2,00	#NULL!	#NULL!	#NULL!	#NULL!	#NULL!
2,00	2,00	#NULL!	#NULL!	#NULL!	#NULL!	#NULL!
2,00	2,00	#NULL!	#NULL!	#NULL!	#NULL!	#NULL!
2,00	2,00	#NULL!	#NULL!	#NULL!	#NULL!	#NULL!
2,00	2,00	#NULL!	#NULL!	#NULL!	#NULL!	#NULL!
2,00	2,00	#NULL!	#NULL!	#NULL!	#NULL!	#NULL!
2,00	2,00	#NULL!	#NULL!	#NULL!	#NULL!	#NULL!
2,00	2,00	#NULL!	#NULL!	#NULL!	#NULL!	#NULL!
2,00	2,00	#NULL!	#NULL!	#NULL!	#NULL!	#NULL!
2,00	2,00	#NULL!	#NULL!	#NULL!	#NULL!	#NULL!
2,00	2,00	#NULL!	#NULL!	#NULL!	#NULL!	#NULL!
2,00	2,00	#NULL!	#NULL!	#NULL!	#NULL!	#NULL!
2,00	2,00	#NULL!	#NULL!	#NULL!	#NULL!	#NULL!
2,00	2,00	#NULL!	#NULL!	#NULL!	#NULL!	#NULL!
2,00	2,00	#NULL!	#NULL!	#NULL!	#NULL!	#NULL!
2,00	2,00	#NULL!	#NULL!	#NULL!	#NULL!	#NULL!
2,00	2,00	#NULL!	#NULL!	#NULL!	#NULL!	#NULL!
2,00	2,00	#NULL!	#NULL!	#NULL!	#NULL!	#NULL!

2,00	2,00	#NULL!	#NULL!	#NULL!	#NULL!	#NULL!
2,00	2,00	#NULL!	#NULL!	#NULL!	#NULL!	#NULL!
2,00	2,00	#NULL!	#NULL!	#NULL!	#NULL!	#NULL!
2,00	2,00	#NULL!	#NULL!	#NULL!	#NULL!	#NULL!
2,00	2,00	#NULL!	#NULL!	#NULL!	#NULL!	#NULL!
2,00	2,00	#NULL!	#NULL!	#NULL!	#NULL!	#NULL!
2,00	2,00	#NULL!	#NULL!	#NULL!	#NULL!	#NULL!
2,00	2,00	#NULL!	#NULL!	#NULL!	#NULL!	#NULL!
2,00	2,00	#NULL!	#NULL!	#NULL!	#NULL!	#NULL!
2,00	2,00	#NULL!	#NULL!	#NULL!	#NULL!	#NULL!
2,00	2,00	#NULL!	#NULL!	#NULL!	#NULL!	#NULL!
2,00	2,00	#NULL!	#NULL!	#NULL!	#NULL!	#NULL!
2,00	2,00	#NULL!	#NULL!	#NULL!	#NULL!	#NULL!
2,00	2,00	#NULL!	#NULL!	#NULL!	#NULL!	#NULL!
2,00	2,00	#NULL!	#NULL!	#NULL!	#NULL!	#NULL!
2,00	2,00	#NULL!	#NULL!	#NULL!	#NULL!	#NULL!
2,00	2,00	#NULL!	#NULL!	#NULL!	#NULL!	#NULL!
2,00	2,00	#NULL!	#NULL!	#NULL!	#NULL!	#NULL!
2,00	2,00	#NULL!	#NULL!	#NULL!	#NULL!	#NULL!
2,00	2,00	#NULL!	#NULL!	#NULL!	#NULL!	#NULL!
2,00	2,00	#NULL!	#NULL!	#NULL!	#NULL!	#NULL!
2,00	2,00	#NULL!	#NULL!	#NULL!	#NULL!	#NULL!
2,00	2,00	#NULL!	#NULL!	#NULL!	#NULL!	#NULL!
2,00	2,00	#NULL!	#NULL!	#NULL!	#NULL!	#NULL!
2,00	2,00	#NULL!	#NULL!	#NULL!	#NULL!	#NULL!
2,00	2,00	#NULL!	#NULL!	#NULL!	#NULL!	#NULL!
2,00	2,00	#NULL!	#NULL!	#NULL!	#NULL!	#NULL!
2,00	2,00	#NULL!	#NULL!	#NULL!	#NULL!	#NULL!
2,00	2,00	#NULL!	#NULL!	#NULL!	#NULL!	#NULL!
2,00	2,00	#NULL!	#NULL!	#NULL!	#NULL!	#NULL!
2,00	2,00	#NULL!	#NULL!	#NULL!	#NULL!	#NULL!
2,00	2,00	#NULL!	#NULL!	#NULL!	#NULL!	#NULL!
2,00	2,00	#NULL!	#NULL!	#NULL!	#NULL!	#NULL!
2,00	2,00	#NULL!	#NULL!	#NULL!	#NULL!	#NULL!
2,00	2,00	#NULL!	#NULL!	#NULL!	#NULL!	#NULL!
2,00	2,00	#NULL!	#NULL!	#NULL!	#NULL!	#NULL!
2,00	2,00	#NULL!	#NULL!	#NULL!	#NULL!	#NULL!
2,00	2,00	#NULL!	#NULL!	#NULL!	#NULL!	#NULL!
2,00	2,00	#NULL!	#NULL!	#NULL!	#NULL!	#NULL!
2,00	2,00	#NULL!	#NULL!	#NULL!	#NULL!	#NULL!
2,00	2,00	#NULL!	#NULL!	#NULL!	#NULL!	#NULL!
2,00	2,00	#NULL!	#NULL!	#NULL!	#NULL!	#NULL!
2,00	2,00	#NULL!	#NULL!	#NULL!	#NULL!	#NULL!
2,00	2,00	#NULL!	#NULL!	#NULL!	#NULL!	#NULL!
2,00	2,00	#NULL!	#NULL!	#NULL!	#NULL!	#NULL!
2,00	2,00	#NULL!	#NULL!	#NULL!	#NULL!	#NULL!
2,00	2,00	#NULL!	#NULL!	#NULL!	#NULL!	#NULL!
2,00	2,00	#NULL!	#NULL!	#NULL!	#NULL!	#NULL!
2,00	2,00	#NULL!	#NULL!	#NULL!	#NULL!	#NULL!
2,00	2,00	#NULL!	#NULL!	#NULL!	#NULL!	#NULL!

2,00	2,00	#NULL!	#NULL!	#NULL!	#NULL!	#NULL!
2,00	2,00	#NULL!	#NULL!	#NULL!	#NULL!	#NULL!
2,00	2,00	#NULL!	#NULL!	#NULL!	#NULL!	#NULL!
2,00	2,00	#NULL!	#NULL!	#NULL!	#NULL!	#NULL!
2,00	2,00	#NULL!	#NULL!	#NULL!	#NULL!	#NULL!
2,00	2,00	#NULL!	#NULL!	#NULL!	#NULL!	#NULL!
2,00	2,00	#NULL!	#NULL!	#NULL!	#NULL!	#NULL!
2,00	2,00	#NULL!	#NULL!	#NULL!	#NULL!	#NULL!
2,00	2,00	#NULL!	#NULL!	#NULL!	#NULL!	#NULL!
2,00	2,00	#NULL!	#NULL!	#NULL!	#NULL!	#NULL!
2,00	2,00	#NULL!	#NULL!	#NULL!	#NULL!	#NULL!
2,00	2,00	#NULL!	#NULL!	#NULL!	#NULL!	#NULL!
2,00	2,00	#NULL!	#NULL!	#NULL!	#NULL!	#NULL!
2,00	2,00	#NULL!	#NULL!	#NULL!	#NULL!	#NULL!
2,00	2,00	#NULL!	#NULL!	#NULL!	#NULL!	#NULL!
2,00	2,00	#NULL!	#NULL!	#NULL!	#NULL!	#NULL!
2,00	2,00	#NULL!	#NULL!	#NULL!	#NULL!	#NULL!
2,00	2,00	#NULL!	#NULL!	#NULL!	#NULL!	#NULL!
2,00	2,00	#NULL!	#NULL!	#NULL!	#NULL!	#NULL!
2,00	2,00	#NULL!	#NULL!	#NULL!	#NULL!	#NULL!
2,00	2,00	#NULL!	#NULL!	#NULL!	#NULL!	#NULL!
2,00	2,00	#NULL!	#NULL!	#NULL!	#NULL!	#NULL!
2,00	2,00	#NULL!	#NULL!	#NULL!	#NULL!	#NULL!
2,00	2,00	#NULL!	#NULL!	#NULL!	#NULL!	#NULL!
2,00	2,00	#NULL!	#NULL!	#NULL!	#NULL!	#NULL!
2,00	2,00	#NULL!	#NULL!	#NULL!	#NULL!	#NULL!
2,00	2,00	#NULL!	#NULL!	#NULL!	#NULL!	#NULL!
2,00	2,00	#NULL!	#NULL!	#NULL!	#NULL!	#NULL!
2,00	2,00	#NULL!	#NULL!	#NULL!	#NULL!	#NULL!
2,00	2,00	#NULL!	#NULL!	#NULL!	#NULL!	#NULL!
2,00	2,00	#NULL!	#NULL!	#NULL!	#NULL!	#NULL!
2,00	2,00	#NULL!	#NULL!	#NULL!	#NULL!	#NULL!
2,00	2,00	#NULL!	#NULL!	#NULL!	#NULL!	#NULL!
2,00	2,00	#NULL!	#NULL!	#NULL!	#NULL!	#NULL!
2,00	2,00	#NULL!	#NULL!	#NULL!	#NULL!	#NULL!
2,00	2,00	#NULL!	#NULL!	#NULL!	#NULL!	#NULL!
2,00	2,00	#NULL!	#NULL!	#NULL!	#NULL!	#NULL!
2,00	2,00	#NULL!	#NULL!	#NULL!	#NULL!	#NULL!
2,00	2,00	#NULL!	#NULL!	#NULL!	#NULL!	#NULL!
2,00	2,00	#NULL!	#NULL!	#NULL!	#NULL!	#NULL!
2,00	2,00	#NULL!	#NULL!	#NULL!	#NULL!	#NULL!
2,00	2,00	#NULL!	#NULL!	#NULL!	#NULL!	#NULL!
2,00	2,00	#NULL!	#NULL!	#NULL!	#NULL!	#NULL!
2,00	2,00	#NULL!	#NULL!	#NULL!	#NULL!	#NULL!
2,00	2,00	#NULL!	#NULL!	#NULL!	#NULL!	#NULL!
2,00	2,00	#NULL!	#NULL!	#NULL!	#NULL!	#NULL!
2,00	2,00	#NULL!	#NULL!	#NULL!	#NULL!	#NULL!
2,00	2,00	#NULL!	#NULL!	#NULL!	#NULL!	#NULL!
2,00	2,00	#NULL!	#NULL!	#NULL!	#NULL!	#NULL!
2,00	2,00	#NULL!	#NULL!	#NULL!	#NULL!	#NULL!

2,00	2,00	#NULL!	#NULL!	#NULL!	#NULL!	#NULL!
2,00	2,00	#NULL!	#NULL!	#NULL!	#NULL!	#NULL!
2,00	2,00	#NULL!	#NULL!	#NULL!	#NULL!	#NULL!
2,00	2,00	#NULL!	#NULL!	#NULL!	#NULL!	#NULL!
2,00	2,00	#NULL!	#NULL!	#NULL!	#NULL!	#NULL!
2,00	2,00	#NULL!	#NULL!	#NULL!	#NULL!	#NULL!
2,00	2,00	#NULL!	#NULL!	#NULL!	#NULL!	#NULL!
2,00	2,00	#NULL!	#NULL!	#NULL!	#NULL!	#NULL!
2,00	2,00	#NULL!	#NULL!	#NULL!	#NULL!	#NULL!
2,00	2,00	#NULL!	#NULL!	#NULL!	#NULL!	#NULL!
2,00	2,00	#NULL!	#NULL!	#NULL!	#NULL!	#NULL!
2,00	2,00	#NULL!	#NULL!	#NULL!	#NULL!	#NULL!
1,00	2,00	#NULL!	#NULL!	#NULL!	#NULL!	#NULL!
2,00	2,00	#NULL!	#NULL!	#NULL!	#NULL!	#NULL!
2,00	2,00	#NULL!	#NULL!	#NULL!	#NULL!	#NULL!
2,00	2,00	#NULL!	#NULL!	#NULL!	#NULL!	#NULL!
2,00	2,00	#NULL!	#NULL!	#NULL!	#NULL!	#NULL!
2,00	2,00	#NULL!	#NULL!	#NULL!	#NULL!	#NULL!
2,00	2,00	#NULL!	#NULL!	#NULL!	#NULL!	#NULL!
2,00	2,00	#NULL!	#NULL!	#NULL!	#NULL!	#NULL!
2,00	2,00	#NULL!	#NULL!	#NULL!	#NULL!	#NULL!
2,00	2,00	#NULL!	#NULL!	#NULL!	#NULL!	#NULL!
2,00	2,00	#NULL!	#NULL!	#NULL!	#NULL!	#NULL!
2,00	2,00	#NULL!	#NULL!	#NULL!	#NULL!	#NULL!
2,00	2,00	#NULL!	#NULL!	#NULL!	#NULL!	#NULL!
2,00	2,00	#NULL!	#NULL!	#NULL!	#NULL!	#NULL!
2,00	2,00	#NULL!	#NULL!	#NULL!	#NULL!	#NULL!
2,00	2,00	#NULL!	#NULL!	#NULL!	#NULL!	#NULL!
2,00	2,00	#NULL!	#NULL!	#NULL!	#NULL!	#NULL!
2,00	2,00	#NULL!	#NULL!	#NULL!	#NULL!	#NULL!
2,00	2,00	#NULL!	#NULL!	#NULL!	#NULL!	#NULL!
2,00	2,00	#NULL!	#NULL!	#NULL!	#NULL!	#NULL!
2,00	2,00	#NULL!	#NULL!	#NULL!	#NULL!	#NULL!
1,00	2,00	#NULL!	#NULL!	#NULL!	#NULL!	#NULL!
2,00	2,00	#NULL!	#NULL!	#NULL!	#NULL!	#NULL!
2,00	2,00	#NULL!	#NULL!	#NULL!	#NULL!	#NULL!
2,00	2,00	#NULL!	#NULL!	#NULL!	#NULL!	#NULL!
2,00	2,00	#NULL!	#NULL!	#NULL!	#NULL!	#NULL!
2,00	2,00	#NULL!	#NULL!	#NULL!	#NULL!	#NULL!
2,00	2,00	#NULL!	#NULL!	#NULL!	#NULL!	#NULL!
2,00	2,00	#NULL!	#NULL!	#NULL!	#NULL!	#NULL!
2,00	2,00	#NULL!	#NULL!	#NULL!	#NULL!	#NULL!
2,00	2,00	#NULL!	#NULL!	#NULL!	#NULL!	#NULL!
2,00	2,00	#NULL!	#NULL!	#NULL!	#NULL!	#NULL!
2,00	2,00	#NULL!	#NULL!	#NULL!	#NULL!	#NULL!
2,00	2,00	#NULL!	#NULL!	#NULL!	#NULL!	#NULL!
2,00	2,00	#NULL!	#NULL!	#NULL!	#NULL!	#NULL!
2,00	2,00	#NULL!	#NULL!	#NULL!	#NULL!	#NULL!
2,00	2,00	#NULL!	#NULL!	#NULL!	#NULL!	#NULL!
2,00	2,00	#NULL!	#NULL!	#NULL!	#NULL!	#NULL!

2,00	2,00	#NULL!	#NULL!	#NULL!	#NULL!	#NULL!
2,00	2,00	#NULL!	#NULL!	#NULL!	#NULL!	#NULL!
2,00	2,00	#NULL!	#NULL!	#NULL!	#NULL!	#NULL!
2,00	2,00	#NULL!	#NULL!	#NULL!	#NULL!	#NULL!
2,00	2,00	#NULL!	#NULL!	#NULL!	#NULL!	#NULL!
2,00	2,00	#NULL!	#NULL!	#NULL!	#NULL!	#NULL!
2,00	2,00	#NULL!	#NULL!	#NULL!	#NULL!	#NULL!
2,00	2,00	#NULL!	#NULL!	#NULL!	#NULL!	#NULL!
2,00	2,00	#NULL!	#NULL!	#NULL!	#NULL!	#NULL!
2,00	2,00	#NULL!	#NULL!	#NULL!	#NULL!	#NULL!
2,00	2,00	#NULL!	#NULL!	#NULL!	#NULL!	#NULL!
2,00	2,00	#NULL!	#NULL!	#NULL!	#NULL!	#NULL!
2,00	2,00	#NULL!	#NULL!	#NULL!	#NULL!	#NULL!
2,00	2,00	#NULL!	#NULL!	#NULL!	#NULL!	#NULL!
2,00	2,00	#NULL!	#NULL!	#NULL!	#NULL!	#NULL!
2,00	2,00	#NULL!	#NULL!	#NULL!	#NULL!	#NULL!
2,00	2,00	#NULL!	#NULL!	#NULL!	#NULL!	#NULL!
2,00	2,00	#NULL!	#NULL!	#NULL!	#NULL!	#NULL!
2,00	2,00	#NULL!	#NULL!	#NULL!	#NULL!	#NULL!
2,00	2,00	#NULL!	#NULL!	#NULL!	#NULL!	#NULL!
2,00	2,00	#NULL!	#NULL!	#NULL!	#NULL!	#NULL!
2,00	2,00	#NULL!	#NULL!	#NULL!	#NULL!	#NULL!
2,00	2,00	#NULL!	#NULL!	#NULL!	#NULL!	#NULL!
2,00	2,00	#NULL!	#NULL!	#NULL!	#NULL!	#NULL!
2,00	2,00	#NULL!	#NULL!	#NULL!	#NULL!	#NULL!
2,00	2,00	#NULL!	#NULL!	#NULL!	#NULL!	#NULL!
2,00	2,00	#NULL!	#NULL!	#NULL!	#NULL!	#NULL!
2,00	2,00	#NULL!	#NULL!	#NULL!	#NULL!	#NULL!
2,00	2,00	#NULL!	#NULL!	#NULL!	#NULL!	#NULL!
2,00	2,00	#NULL!	#NULL!	#NULL!	#NULL!	#NULL!
2,00	2,00	#NULL!	#NULL!	#NULL!	#NULL!	#NULL!
2,00	2,00	#NULL!	#NULL!	#NULL!	#NULL!	#NULL!
2,00	2,00	#NULL!	#NULL!	#NULL!	#NULL!	#NULL!
2,00	2,00	#NULL!	#NULL!	#NULL!	#NULL!	#NULL!
2,00	2,00	#NULL!	#NULL!	#NULL!	#NULL!	#NULL!
2,00	2,00	#NULL!	#NULL!	#NULL!	#NULL!	#NULL!
2,00	2,00	#NULL!	#NULL!	#NULL!	#NULL!	#NULL!
2,00	2,00	#NULL!	#NULL!	#NULL!	#NULL!	#NULL!
2,00	2,00	#NULL!	#NULL!	#NULL!	#NULL!	#NULL!
2,00	2,00	#NULL!	#NULL!	#NULL!	#NULL!	#NULL!
2,00	2,00	#NULL!	#NULL!	#NULL!	#NULL!	#NULL!
2,00	2,00	#NULL!	#NULL!	#NULL!	#NULL!	#NULL!
2,00	2,00	#NULL!	#NULL!	#NULL!	#NULL!	#NULL!
2,00	2,00	#NULL!	#NULL!	#NULL!	#NULL!	#NULL!
2,00	2,00	#NULL!	#NULL!	#NULL!	#NULL!	#NULL!
2,00	2,00	#NULL!	#NULL!	#NULL!	#NULL!	#NULL!
2,00	2,00	#NULL!	#NULL!	#NULL!	#NULL!	#NULL!
2,00	2,00	#NULL!	#NULL!	#NULL!	#NULL!	#NULL!
2,00	2,00	#NULL!	#NULL!	#NULL!	#NULL!	#NULL!
2,00	2,00	#NULL!	#NULL!	#NULL!	#NULL!	#NULL!

2,00	2,00	#NULL!	#NULL!	#NULL!	#NULL!	#NULL!
2,00	2,00	#NULL!	#NULL!	#NULL!	#NULL!	#NULL!
2,00	2,00	#NULL!	#NULL!	#NULL!	#NULL!	#NULL!
2,00	2,00	#NULL!	#NULL!	#NULL!	#NULL!	#NULL!
2,00	2,00	#NULL!	#NULL!	#NULL!	#NULL!	#NULL!
2,00	2,00	#NULL!	#NULL!	#NULL!	#NULL!	#NULL!
2,00	2,00	#NULL!	#NULL!	#NULL!	#NULL!	#NULL!
2,00	2,00	#NULL!	#NULL!	#NULL!	#NULL!	#NULL!
2,00	2,00	#NULL!	#NULL!	#NULL!	#NULL!	#NULL!
2,00	2,00	#NULL!	#NULL!	#NULL!	#NULL!	#NULL!
2,00	2,00	#NULL!	#NULL!	#NULL!	#NULL!	#NULL!
2,00	2,00	#NULL!	#NULL!	#NULL!	#NULL!	#NULL!
2,00	2,00	#NULL!	#NULL!	#NULL!	#NULL!	#NULL!
2,00	2,00	#NULL!	#NULL!	#NULL!	#NULL!	#NULL!
2,00	2,00	#NULL!	#NULL!	#NULL!	#NULL!	#NULL!
2,00	2,00	#NULL!	#NULL!	#NULL!	#NULL!	#NULL!
2,00	2,00	#NULL!	#NULL!	#NULL!	#NULL!	#NULL!
2,00	2,00	#NULL!	#NULL!	#NULL!	#NULL!	#NULL!
2,00	2,00	#NULL!	#NULL!	#NULL!	#NULL!	#NULL!
2,00	2,00	#NULL!	#NULL!	#NULL!	#NULL!	#NULL!
2,00	2,00	#NULL!	#NULL!	#NULL!	#NULL!	#NULL!
2,00	2,00	#NULL!	#NULL!	#NULL!	#NULL!	#NULL!
2,00	2,00	#NULL!	#NULL!	#NULL!	#NULL!	#NULL!
2,00	2,00	#NULL!	#NULL!	#NULL!	#NULL!	#NULL!
2,00	2,00	#NULL!	#NULL!	#NULL!	#NULL!	#NULL!
2,00	2,00	#NULL!	#NULL!	#NULL!	#NULL!	#NULL!
2,00	2,00	#NULL!	#NULL!	#NULL!	#NULL!	#NULL!
2,00	2,00	#NULL!	#NULL!	#NULL!	#NULL!	#NULL!
2,00	2,00	#NULL!	#NULL!	#NULL!	#NULL!	#NULL!
2,00	2,00	#NULL!	#NULL!	#NULL!	#NULL!	#NULL!
2,00	2,00	#NULL!	#NULL!	#NULL!	#NULL!	#NULL!
2,00	2,00	#NULL!	#NULL!	#NULL!	#NULL!	#NULL!
2,00	2,00	#NULL!	#NULL!	#NULL!	#NULL!	#NULL!
2,00	2,00	#NULL!	#NULL!	#NULL!	#NULL!	#NULL!
2,00	2,00	#NULL!	#NULL!	#NULL!	#NULL!	#NULL!
2,00	2,00	#NULL!	#NULL!	#NULL!	#NULL!	#NULL!
2,00	2,00	#NULL!	#NULL!	#NULL!	#NULL!	#NULL!
2,00	2,00	#NULL!	#NULL!	#NULL!	#NULL!	#NULL!
2,00	2,00	#NULL!	#NULL!	#NULL!	#NULL!	#NULL!
2,00	2,00	#NULL!	#NULL!	#NULL!	#NULL!	#NULL!
2,00	2,00	#NULL!	#NULL!	#NULL!	#NULL!	#NULL!
2,00	2,00	#NULL!	#NULL!	#NULL!	#NULL!	#NULL!
2,00	2,00	#NULL!	#NULL!	#NULL!	#NULL!	#NULL!
2,00	2,00	#NULL!	#NULL!	#NULL!	#NULL!	#NULL!
2,00	2,00	#NULL!	#NULL!	#NULL!	#NULL!	#NULL!
2,00	2,00	#NULL!	#NULL!	#NULL!	#NULL!	#NULL!
2,00	2,00	#NULL!	#NULL!	#NULL!	#NULL!	#NULL!
2,00	2,00	#NULL!	#NULL!	#NULL!	#NULL!	#NULL!
2,00	2,00	#NULL!	#NULL!	#NULL!	#NULL!	#NULL!
2,00	2,00	#NULL!	#NULL!	#NULL!	#NULL!	#NULL!

2,00	2,00	#NULL!	#NULL!	#NULL!	#NULL!	#NULL!
2,00	2,00	#NULL!	#NULL!	#NULL!	#NULL!	#NULL!
2,00	2,00	#NULL!	#NULL!	#NULL!	#NULL!	#NULL!
2,00	2,00	#NULL!	#NULL!	#NULL!	#NULL!	#NULL!
2,00	2,00	#NULL!	#NULL!	#NULL!	#NULL!	#NULL!
2,00	2,00	#NULL!	#NULL!	#NULL!	#NULL!	#NULL!
2,00	2,00	#NULL!	#NULL!	#NULL!	#NULL!	#NULL!
2,00	2,00	#NULL!	#NULL!	#NULL!	#NULL!	#NULL!
2,00	2,00	#NULL!	#NULL!	#NULL!	#NULL!	#NULL!
2,00	2,00	#NULL!	#NULL!	#NULL!	#NULL!	#NULL!
2,00	2,00	#NULL!	#NULL!	#NULL!	#NULL!	#NULL!
2,00	2,00	#NULL!	#NULL!	#NULL!	#NULL!	#NULL!
2,00	2,00	#NULL!	#NULL!	#NULL!	#NULL!	#NULL!
2,00	2,00	#NULL!	#NULL!	#NULL!	#NULL!	#NULL!
2,00	2,00	#NULL!	#NULL!	#NULL!	#NULL!	#NULL!
1,00	2,00	#NULL!	#NULL!	#NULL!	#NULL!	#NULL!
2,00	2,00	#NULL!	#NULL!	#NULL!	#NULL!	#NULL!
2,00	2,00	#NULL!	#NULL!	#NULL!	#NULL!	#NULL!
2,00	2,00	#NULL!	#NULL!	#NULL!	#NULL!	#NULL!
2,00	2,00	#NULL!	#NULL!	#NULL!	#NULL!	#NULL!
2,00	2,00	#NULL!	#NULL!	#NULL!	#NULL!	#NULL!
2,00	2,00	#NULL!	#NULL!	#NULL!	#NULL!	#NULL!
2,00	2,00	#NULL!	#NULL!	#NULL!	#NULL!	#NULL!
2,00	2,00	#NULL!	#NULL!	#NULL!	#NULL!	#NULL!
2,00	2,00	#NULL!	#NULL!	#NULL!	#NULL!	#NULL!
2,00	2,00	#NULL!	#NULL!	#NULL!	#NULL!	#NULL!
2,00	2,00	#NULL!	#NULL!	#NULL!	#NULL!	#NULL!
2,00	2,00	#NULL!	#NULL!	#NULL!	#NULL!	#NULL!
2,00	2,00	#NULL!	#NULL!	#NULL!	#NULL!	#NULL!
2,00	2,00	#NULL!	#NULL!	#NULL!	#NULL!	#NULL!
2,00	2,00	#NULL!	#NULL!	#NULL!	#NULL!	#NULL!
2,00	2,00	#NULL!	#NULL!	#NULL!	#NULL!	#NULL!
2,00	2,00	#NULL!	#NULL!	#NULL!	#NULL!	#NULL!
2,00	2,00	#NULL!	#NULL!	#NULL!	#NULL!	#NULL!
2,00	2,00	#NULL!	#NULL!	#NULL!	#NULL!	#NULL!
2,00	2,00	#NULL!	#NULL!	#NULL!	#NULL!	#NULL!
2,00	2,00	#NULL!	#NULL!	#NULL!	#NULL!	#NULL!
2,00	2,00	#NULL!	#NULL!	#NULL!	#NULL!	#NULL!
2,00	2,00	#NULL!	#NULL!	#NULL!	#NULL!	#NULL!
2,00	2,00	#NULL!	#NULL!	#NULL!	#NULL!	#NULL!
2,00	2,00	#NULL!	#NULL!	#NULL!	#NULL!	#NULL!
2,00	2,00	#NULL!	#NULL!	#NULL!	#NULL!	#NULL!
2,00	2,00	#NULL!	#NULL!	#NULL!	#NULL!	#NULL!
2,00	2,00	#NULL!	#NULL!	#NULL!	#NULL!	#NULL!
2,00	2,00	#NULL!	#NULL!	#NULL!	#NULL!	#NULL!
2,00	2,00	#NULL!	#NULL!	#NULL!	#NULL!	#NULL!
2,00	2,00	#NULL!	#NULL!	#NULL!	#NULL!	#NULL!
2,00	2,00	#NULL!	#NULL!	#NULL!	#NULL!	#NULL!
2,00	2,00	#NULL!	#NULL!	#NULL!	#NULL!	#NULL!
2,00	2,00	#NULL!	#NULL!	#NULL!	#NULL!	#NULL!

2,00	2,00	#NULL!	#NULL!	#NULL!	#NULL!	#NULL!
2,00	2,00	#NULL!	#NULL!	#NULL!	#NULL!	#NULL!
2,00	2,00	#NULL!	#NULL!	#NULL!	#NULL!	#NULL!
2,00	2,00	#NULL!	#NULL!	#NULL!	#NULL!	#NULL!
2,00	2,00	#NULL!	#NULL!	#NULL!	#NULL!	#NULL!
2,00	2,00	#NULL!	#NULL!	#NULL!	#NULL!	#NULL!
2,00	2,00	#NULL!	#NULL!	#NULL!	#NULL!	#NULL!
2,00	2,00	#NULL!	#NULL!	#NULL!	#NULL!	#NULL!
2,00	2,00	#NULL!	#NULL!	#NULL!	#NULL!	#NULL!
2,00	2,00	#NULL!	#NULL!	#NULL!	#NULL!	#NULL!
2,00	2,00	#NULL!	#NULL!	#NULL!	#NULL!	#NULL!
2,00	2,00	#NULL!	#NULL!	#NULL!	#NULL!	#NULL!
2,00	2,00	#NULL!	#NULL!	#NULL!	#NULL!	#NULL!
2,00	2,00	#NULL!	#NULL!	#NULL!	#NULL!	#NULL!
2,00	2,00	#NULL!	#NULL!	#NULL!	#NULL!	#NULL!
2,00	2,00	#NULL!	#NULL!	#NULL!	#NULL!	#NULL!
2,00	2,00	#NULL!	#NULL!	#NULL!	#NULL!	#NULL!
2,00	2,00	#NULL!	#NULL!	#NULL!	#NULL!	#NULL!
2,00	2,00	#NULL!	#NULL!	#NULL!	#NULL!	#NULL!
2,00	2,00	#NULL!	#NULL!	#NULL!	#NULL!	#NULL!
2,00	2,00	#NULL!	#NULL!	#NULL!	#NULL!	#NULL!
2,00	2,00	#NULL!	#NULL!	#NULL!	#NULL!	#NULL!
2,00	2,00	#NULL!	#NULL!	#NULL!	#NULL!	#NULL!
2,00	2,00	#NULL!	#NULL!	#NULL!	#NULL!	#NULL!
2,00	2,00	#NULL!	#NULL!	#NULL!	#NULL!	#NULL!
2,00	2,00	#NULL!	#NULL!	#NULL!	#NULL!	#NULL!
2,00	2,00	#NULL!	#NULL!	#NULL!	#NULL!	#NULL!
2,00	2,00	#NULL!	#NULL!	#NULL!	#NULL!	#NULL!
2,00	2,00	#NULL!	#NULL!	#NULL!	#NULL!	#NULL!
2,00	2,00	#NULL!	#NULL!	#NULL!	#NULL!	#NULL!
2,00	2,00	#NULL!	#NULL!	#NULL!	#NULL!	#NULL!
2,00	2,00	#NULL!	#NULL!	#NULL!	#NULL!	#NULL!
2,00	2,00	#NULL!	#NULL!	#NULL!	#NULL!	#NULL!
2,00	2,00	#NULL!	#NULL!	#NULL!	#NULL!	#NULL!
2,00	2,00	#NULL!	#NULL!	#NULL!	#NULL!	#NULL!
2,00	2,00	#NULL!	#NULL!	#NULL!	#NULL!	#NULL!
2,00	2,00	#NULL!	#NULL!	#NULL!	#NULL!	#NULL!
2,00	2,00	#NULL!	#NULL!	#NULL!	#NULL!	#NULL!
2,00	2,00	#NULL!	#NULL!	#NULL!	#NULL!	#NULL!
2,00	2,00	#NULL!	#NULL!	#NULL!	#NULL!	#NULL!
2,00	2,00	#NULL!	#NULL!	#NULL!	#NULL!	#NULL!
2,00	2,00	#NULL!	#NULL!	#NULL!	#NULL!	#NULL!
2,00	2,00	#NULL!	#NULL!	#NULL!	#NULL!	#NULL!
2,00	2,00	#NULL!	#NULL!	#NULL!	#NULL!	#NULL!
1,00	2,00	#NULL!	#NULL!	#NULL!	#NULL!	#NULL!
2,00	2,00	#NULL!	#NULL!	#NULL!	#NULL!	#NULL!
2,00	2,00	#NULL!	#NULL!	#NULL!	#NULL!	#NULL!
2,00	2,00	#NULL!	#NULL!	#NULL!	#NULL!	#NULL!
2,00	2,00	#NULL!	#NULL!	#NULL!	#NULL!	#NULL!
2,00	2,00	#NULL!	#NULL!	#NULL!	#NULL!	#NULL!

2,00	2,00	#NULL!	#NULL!	#NULL!	#NULL!	#NULL!
2,00	2,00	#NULL!	#NULL!	#NULL!	#NULL!	#NULL!
2,00	2,00	#NULL!	#NULL!	#NULL!	#NULL!	#NULL!
2,00	2,00	#NULL!	#NULL!	#NULL!	#NULL!	#NULL!
2,00	2,00	#NULL!	#NULL!	#NULL!	#NULL!	#NULL!
2,00	2,00	#NULL!	#NULL!	#NULL!	#NULL!	#NULL!
2,00	2,00	#NULL!	#NULL!	#NULL!	#NULL!	#NULL!
2,00	2,00	#NULL!	#NULL!	#NULL!	#NULL!	#NULL!
2,00	2,00	#NULL!	#NULL!	#NULL!	#NULL!	#NULL!
2,00	2,00	#NULL!	#NULL!	#NULL!	#NULL!	#NULL!
2,00	2,00	#NULL!	#NULL!	#NULL!	#NULL!	#NULL!
2,00	2,00	#NULL!	#NULL!	#NULL!	#NULL!	#NULL!
2,00	2,00	#NULL!	#NULL!	#NULL!	#NULL!	#NULL!
2,00	2,00	#NULL!	#NULL!	#NULL!	#NULL!	#NULL!
2,00	2,00	#NULL!	#NULL!	#NULL!	#NULL!	#NULL!
2,00	2,00	#NULL!	#NULL!	#NULL!	#NULL!	#NULL!
2,00	2,00	#NULL!	#NULL!	#NULL!	#NULL!	#NULL!
2,00	2,00	#NULL!	#NULL!	#NULL!	#NULL!	#NULL!
2,00	2,00	#NULL!	#NULL!	#NULL!	#NULL!	#NULL!
2,00	2,00	#NULL!	#NULL!	#NULL!	#NULL!	#NULL!
1,00	2,00	#NULL!	#NULL!	#NULL!	#NULL!	#NULL!
2,00	1,00	1,00	2,00	1,00	2,00	#NULL!
2,00	2,00	#NULL!	#NULL!	#NULL!	#NULL!	#NULL!
2,00	2,00	#NULL!	#NULL!	#NULL!	#NULL!	#NULL!
2,00	1,00	1,00	1,00	2,00	2,00	#NULL!
2,00	2,00	#NULL!	#NULL!	#NULL!	#NULL!	#NULL!
2,00	2,00	#NULL!	#NULL!	#NULL!	#NULL!	#NULL!
2,00	2,00	#NULL!	#NULL!	#NULL!	#NULL!	#NULL!
2,00	2,00	#NULL!	#NULL!	#NULL!	#NULL!	#NULL!
2,00	1,00	1,00	1,00	2,00	2,00	#NULL!
2,00	2,00	#NULL!	#NULL!	#NULL!	#NULL!	#NULL!
2,00	2,00	#NULL!	#NULL!	#NULL!	#NULL!	#NULL!
2,00	2,00	#NULL!	#NULL!	#NULL!	#NULL!	#NULL!
2,00	1,00	1,00	1,00	2,00	2,00	#NULL!
2,00	2,00	#NULL!	#NULL!	#NULL!	#NULL!	#NULL!
2,00	2,00	#NULL!	#NULL!	#NULL!	#NULL!	#NULL!
2,00	2,00	#NULL!	#NULL!	#NULL!	#NULL!	#NULL!
2,00	2,00	#NULL!	#NULL!	#NULL!	#NULL!	#NULL!
2,00	2,00	#NULL!	#NULL!	#NULL!	#NULL!	#NULL!
2,00	1,00	1,00	3,00	2,00	2,00	#NULL!
2,00	2,00	#NULL!	#NULL!	#NULL!	#NULL!	#NULL!
2,00	2,00	#NULL!	#NULL!	#NULL!	#NULL!	#NULL!
2,00	2,00	#NULL!	#NULL!	#NULL!	#NULL!	#NULL!
2,00	2,00	#NULL!	#NULL!	#NULL!	#NULL!	#NULL!
2,00	2,00	#NULL!	#NULL!	#NULL!	#NULL!	#NULL!
2,00	2,00	#NULL!	#NULL!	#NULL!	#NULL!	#NULL!
2,00	1,00	1,00	1,00	2,00	2,00	#NULL!
2,00	2,00	#NULL!	#NULL!	#NULL!	#NULL!	#NULL!
2,00	2,00	#NULL!	#NULL!	#NULL!	#NULL!	#NULL!
2,00	2,00	#NULL!	#NULL!	#NULL!	#NULL!	#NULL!

2,00	2,00	#NULL!	#NULL!	#NULL!	#NULL!	#NULL!
2,00	2,00	#NULL!	#NULL!	#NULL!	#NULL!	#NULL!
2,00	1,00	1,00	1,00	2,00	2,00	#NULL!
2,00	1,00	1,00	1,00	2,00	1,00	#NULL!
2,00	2,00	#NULL!	#NULL!	#NULL!	#NULL!	#NULL!
2,00	2,00	#NULL!	#NULL!	#NULL!	#NULL!	#NULL!
2,00	2,00	#NULL!	#NULL!	#NULL!	#NULL!	#NULL!
2,00	1,00	1,00	1,00	2,00	1,00	#NULL!
2,00	2,00	#NULL!	#NULL!	#NULL!	#NULL!	#NULL!
2,00	1,00	1,00	2,00	2,00	1,00	#NULL!
2,00	2,00	#NULL!	#NULL!	#NULL!	#NULL!	#NULL!
2,00	2,00	#NULL!	#NULL!	#NULL!	#NULL!	#NULL!
2,00	2,00	#NULL!	#NULL!	#NULL!	#NULL!	#NULL!
2,00	1,00	1,00	2,00	1,00	2,00	#NULL!
2,00	2,00	#NULL!	#NULL!	#NULL!	#NULL!	#NULL!
2,00	2,00	#NULL!	#NULL!	#NULL!	#NULL!	#NULL!
2,00	2,00	#NULL!	#NULL!	#NULL!	#NULL!	#NULL!
2,00	2,00	#NULL!	#NULL!	#NULL!	#NULL!	#NULL!
2,00	1,00	1,00	1,00	2,00	2,00	#NULL!
2,00	2,00	#NULL!	#NULL!	#NULL!	#NULL!	#NULL!
2,00	2,00	#NULL!	#NULL!	#NULL!	#NULL!	#NULL!
2,00	1,00	1,00	2,00	1,00	2,00	#NULL!
2,00	2,00	#NULL!	#NULL!	#NULL!	#NULL!	#NULL!
2,00	2,00	#NULL!	#NULL!	#NULL!	#NULL!	#NULL!
2,00	2,00	#NULL!	#NULL!	#NULL!	#NULL!	#NULL!
2,00	1,00	1,00	3,00	2,00	2,00	#NULL!
2,00	2,00	#NULL!	#NULL!	#NULL!	#NULL!	#NULL!
2,00	2,00	#NULL!	#NULL!	#NULL!	#NULL!	#NULL!
2,00	2,00	#NULL!	#NULL!	#NULL!	#NULL!	#NULL!
2,00	1,00	1,00	2,00	1,00	2,00	#NULL!
2,00	2,00	#NULL!	#NULL!	#NULL!	#NULL!	#NULL!
2,00	2,00	#NULL!	#NULL!	#NULL!	#NULL!	#NULL!
2,00	2,00	#NULL!	#NULL!	#NULL!	#NULL!	#NULL!
2,00	1,00	1,00	1,00	2,00	2,00	#NULL!
2,00	1,00	1,00	1,00	2,00	2,00	#NULL!
2,00	2,00	#NULL!	#NULL!	#NULL!	#NULL!	#NULL!
2,00	1,00	1,00	1,00	2,00	2,00	#NULL!
2,00	2,00	#NULL!	#NULL!	#NULL!	#NULL!	#NULL!
2,00	2,00	#NULL!	#NULL!	#NULL!	#NULL!	#NULL!
2,00	1,00	2,00	2,00	1,00	2,00	#NULL!
2,00	2,00	#NULL!	#NULL!	#NULL!	#NULL!	#NULL!
2,00	2,00	#NULL!	#NULL!	#NULL!	#NULL!	#NULL!
2,00	2,00	#NULL!	#NULL!	#NULL!	#NULL!	#NULL!
2,00	1,00	1,00	2,00	2,00	2,00	#NULL!
2,00	2,00	#NULL!	#NULL!	#NULL!	#NULL!	#NULL!
2,00	2,00	#NULL!	#NULL!	#NULL!	#NULL!	#NULL!
2,00	2,00	#NULL!	#NULL!	#NULL!	#NULL!	#NULL!
2,00	1,00	1,00	1,00	2,00	2,00	#NULL!
2,00	2,00	#NULL!	#NULL!	#NULL!	#NULL!	#NULL!
1,00	2,00	#NULL!	#NULL!	#NULL!	#NULL!	#NULL!

2,00	2,00	#NULL!	#NULL!	#NULL!	#NULL!	#NULL!
2,00	1,00	1,00	1,00	2,00	1,00	#NULL!
2,00	2,00	#NULL!	#NULL!	#NULL!	#NULL!	#NULL!
2,00	2,00	#NULL!	#NULL!	#NULL!	#NULL!	#NULL!
2,00	2,00	#NULL!	#NULL!	#NULL!	#NULL!	#NULL!
2,00	2,00	#NULL!	#NULL!	#NULL!	#NULL!	#NULL!
2,00	2,00	#NULL!	#NULL!	#NULL!	#NULL!	#NULL!
2,00	2,00	#NULL!	#NULL!	#NULL!	#NULL!	#NULL!
2,00	2,00	#NULL!	#NULL!	#NULL!	#NULL!	#NULL!
2,00	2,00	#NULL!	#NULL!	#NULL!	#NULL!	#NULL!
2,00	2,00	#NULL!	#NULL!	#NULL!	#NULL!	#NULL!
2,00	1,00	1,00	2,00	1,00	2,00	#NULL!
2,00	2,00	#NULL!	#NULL!	#NULL!	#NULL!	#NULL!
2,00	2,00	#NULL!	#NULL!	#NULL!	#NULL!	#NULL!
2,00	2,00	#NULL!	#NULL!	#NULL!	#NULL!	#NULL!
2,00	2,00	#NULL!	#NULL!	#NULL!	#NULL!	#NULL!
2,00	2,00	#NULL!	#NULL!	#NULL!	#NULL!	#NULL!
2,00	2,00	#NULL!	#NULL!	#NULL!	#NULL!	#NULL!
2,00	2,00	#NULL!	#NULL!	#NULL!	#NULL!	#NULL!
2,00	2,00	#NULL!	#NULL!	#NULL!	#NULL!	#NULL!
2,00	2,00	#NULL!	#NULL!	#NULL!	#NULL!	#NULL!
2,00	2,00	#NULL!	#NULL!	#NULL!	#NULL!	#NULL!
2,00	2,00	#NULL!	#NULL!	#NULL!	#NULL!	#NULL!
2,00	2,00	#NULL!	#NULL!	#NULL!	#NULL!	#NULL!
2,00	2,00	#NULL!	#NULL!	#NULL!	#NULL!	#NULL!
2,00	2,00	#NULL!	#NULL!	#NULL!	#NULL!	#NULL!
2,00	1,00	1,00	1,00	2,00	2,00	#NULL!
2,00	2,00	#NULL!	#NULL!	#NULL!	#NULL!	#NULL!
2,00	2,00	#NULL!	#NULL!	#NULL!	#NULL!	#NULL!
2,00	1,00	1,00	1,00	2,00	2,00	#NULL!
2,00	2,00	#NULL!	#NULL!	#NULL!	#NULL!	#NULL!
2,00	2,00	#NULL!	#NULL!	#NULL!	#NULL!	#NULL!
2,00	2,00	#NULL!	#NULL!	#NULL!	#NULL!	#NULL!
2,00	2,00	#NULL!	#NULL!	#NULL!	#NULL!	#NULL!
2,00	1,00	1,00	2,00	2,00	2,00	#NULL!
2,00	2,00	#NULL!	#NULL!	#NULL!	#NULL!	#NULL!
2,00	2,00	#NULL!	#NULL!	#NULL!	#NULL!	#NULL!
2,00	1,00	2,00	3,00	1,00	2,00	#NULL!
2,00	2,00	#NULL!	#NULL!	#NULL!	#NULL!	#NULL!
1,00	2,00	#NULL!	#NULL!	#NULL!	#NULL!	#NULL!
2,00	2,00	#NULL!	#NULL!	#NULL!	#NULL!	#NULL!
2,00	1,00	1,00	2,00	2,00	2,00	#NULL!
2,00	2,00	#NULL!	#NULL!	#NULL!	#NULL!	#NULL!
2,00	2,00	#NULL!	#NULL!	#NULL!	#NULL!	#NULL!
2,00	2,00	#NULL!	#NULL!	#NULL!	#NULL!	#NULL!
2,00	1,00	1,00	2,00	2,00	2,00	#NULL!
2,00	2,00	#NULL!	#NULL!	#NULL!	#NULL!	#NULL!
2,00	2,00	#NULL!	#NULL!	#NULL!	#NULL!	#NULL!
2,00	2,00	#NULL!	#NULL!	#NULL!	#NULL!	#NULL!
2,00	1,00	1,00	2,00	1,00	2,00	#NULL!

2,00	2,00	#NULL!	#NULL!	#NULL!	#NULL!	#NULL!
2,00	2,00	#NULL!	#NULL!	#NULL!	#NULL!	#NULL!
2,00	2,00	#NULL!	#NULL!	#NULL!	#NULL!	#NULL!
2,00	2,00	#NULL!	#NULL!	#NULL!	#NULL!	#NULL!
2,00	1,00	2,00	1,00	2,00	2,00	#NULL!
2,00	2,00	#NULL!	#NULL!	#NULL!	#NULL!	#NULL!
2,00	2,00	#NULL!	#NULL!	#NULL!	#NULL!	#NULL!
2,00	2,00	#NULL!	#NULL!	#NULL!	#NULL!	#NULL!
2,00	2,00	#NULL!	#NULL!	#NULL!	#NULL!	#NULL!
2,00	2,00	#NULL!	#NULL!	#NULL!	#NULL!	#NULL!
2,00	2,00	#NULL!	#NULL!	#NULL!	#NULL!	#NULL!
1,00	1,00	2,00	1,00	2,00	2,00	#NULL!
2,00	2,00	#NULL!	#NULL!	#NULL!	#NULL!	#NULL!
2,00	2,00	#NULL!	#NULL!	#NULL!	#NULL!	#NULL!
2,00	2,00	#NULL!	#NULL!	#NULL!	#NULL!	#NULL!
2,00	2,00	#NULL!	#NULL!	#NULL!	#NULL!	#NULL!
2,00	2,00	#NULL!	#NULL!	#NULL!	#NULL!	#NULL!
2,00	2,00	#NULL!	#NULL!	#NULL!	#NULL!	#NULL!
2,00	2,00	#NULL!	#NULL!	#NULL!	#NULL!	#NULL!
2,00	1,00	1,00	1,00	2,00	2,00	#NULL!
2,00	2,00	#NULL!	#NULL!	#NULL!	#NULL!	#NULL!
2,00	2,00	#NULL!	#NULL!	#NULL!	#NULL!	#NULL!
2,00	2,00	#NULL!	#NULL!	#NULL!	#NULL!	#NULL!
2,00	1,00	1,00	1,00	2,00	2,00	#NULL!
2,00	2,00	#NULL!	#NULL!	#NULL!	#NULL!	#NULL!
2,00	2,00	#NULL!	#NULL!	#NULL!	#NULL!	#NULL!
2,00	2,00	#NULL!	#NULL!	#NULL!	#NULL!	#NULL!
2,00	1,00	1,00	2,00	1,00	2,00	#NULL!
2,00	2,00	#NULL!	#NULL!	#NULL!	#NULL!	#NULL!
2,00	2,00	#NULL!	#NULL!	#NULL!	#NULL!	#NULL!
2,00	2,00	#NULL!	#NULL!	#NULL!	#NULL!	#NULL!
2,00	1,00	1,00	2,00	1,00	2,00	#NULL!
2,00	2,00	#NULL!	#NULL!	#NULL!	#NULL!	#NULL!
2,00	2,00	#NULL!	#NULL!	#NULL!	#NULL!	#NULL!
2,00	2,00	#NULL!	#NULL!	#NULL!	#NULL!	#NULL!
2,00	2,00	#NULL!	#NULL!	#NULL!	#NULL!	#NULL!
2,00	1,00	1,00	2,00	2,00	2,00	#NULL!
2,00	2,00	#NULL!	#NULL!	#NULL!	#NULL!	#NULL!
1,00	2,00	#NULL!	#NULL!	#NULL!	#NULL!	#NULL!
2,00	2,00	#NULL!	#NULL!	#NULL!	#NULL!	#NULL!
2,00	2,00	#NULL!	#NULL!	#NULL!	#NULL!	#NULL!
2,00	2,00	#NULL!	#NULL!	#NULL!	#NULL!	#NULL!
2,00	2,00	#NULL!	#NULL!	#NULL!	#NULL!	#NULL!
2,00	2,00	#NULL!	#NULL!	#NULL!	#NULL!	#NULL!
2,00	2,00	#NULL!	#NULL!	#NULL!	#NULL!	#NULL!
2,00	2,00	#NULL!	#NULL!	#NULL!	#NULL!	#NULL!
2,00	2,00	#NULL!	#NULL!	#NULL!	#NULL!	#NULL!
2,00	2,00	#NULL!	#NULL!	#NULL!	#NULL!	#NULL!
2,00	2,00	#NULL!	#NULL!	#NULL!	#NULL!	#NULL!
2,00	2,00	#NULL!	#NULL!	#NULL!	#NULL!	#NULL!

2,00	2,00	#NULL!	#NULL!	#NULL!	#NULL!	#NULL!
2,00	2,00	#NULL!	#NULL!	#NULL!	#NULL!	#NULL!
2,00	2,00	#NULL!	#NULL!	#NULL!	#NULL!	#NULL!
2,00	2,00	#NULL!	#NULL!	#NULL!	#NULL!	#NULL!
2,00	2,00	#NULL!	#NULL!	#NULL!	#NULL!	#NULL!
2,00	2,00	#NULL!	#NULL!	#NULL!	#NULL!	#NULL!
2,00	2,00	#NULL!	#NULL!	#NULL!	#NULL!	#NULL!
2,00	2,00	#NULL!	#NULL!	#NULL!	#NULL!	#NULL!
2,00	2,00	#NULL!	#NULL!	#NULL!	#NULL!	#NULL!
2,00	2,00	#NULL!	#NULL!	#NULL!	#NULL!	#NULL!
2,00	2,00	#NULL!	#NULL!	#NULL!	#NULL!	#NULL!
2,00	2,00	#NULL!	#NULL!	#NULL!	#NULL!	#NULL!
2,00	2,00	#NULL!	#NULL!	#NULL!	#NULL!	#NULL!
2,00	2,00	#NULL!	#NULL!	#NULL!	#NULL!	#NULL!
2,00	2,00	#NULL!	#NULL!	#NULL!	#NULL!	#NULL!
2,00	2,00	#NULL!	#NULL!	#NULL!	#NULL!	#NULL!
2,00	2,00	#NULL!	#NULL!	#NULL!	#NULL!	#NULL!
2,00	2,00	#NULL!	#NULL!	#NULL!	#NULL!	#NULL!
2,00	2,00	#NULL!	#NULL!	#NULL!	#NULL!	#NULL!
2,00	2,00	#NULL!	#NULL!	#NULL!	#NULL!	#NULL!
2,00	2,00	#NULL!	#NULL!	#NULL!	#NULL!	#NULL!
2,00	2,00	#NULL!	#NULL!	#NULL!	#NULL!	#NULL!
2,00	2,00	#NULL!	#NULL!	#NULL!	#NULL!	#NULL!
2,00	2,00	#NULL!	#NULL!	#NULL!	#NULL!	#NULL!
2,00	2,00	#NULL!	#NULL!	#NULL!	#NULL!	#NULL!
2,00	2,00	#NULL!	#NULL!	#NULL!	#NULL!	#NULL!
2,00	2,00	#NULL!	#NULL!	#NULL!	#NULL!	#NULL!
2,00	2,00	#NULL!	#NULL!	#NULL!	#NULL!	#NULL!
2,00	2,00	#NULL!	#NULL!	#NULL!	#NULL!	#NULL!
2,00	2,00	#NULL!	#NULL!	#NULL!	#NULL!	#NULL!
2,00	2,00	#NULL!	#NULL!	#NULL!	#NULL!	#NULL!
2,00	2,00	#NULL!	#NULL!	#NULL!	#NULL!	#NULL!
2,00	2,00	#NULL!	#NULL!	#NULL!	#NULL!	#NULL!
2,00	2,00	#NULL!	#NULL!	#NULL!	#NULL!	#NULL!
2,00	2,00	#NULL!	#NULL!	#NULL!	#NULL!	#NULL!
2,00	2,00	#NULL!	#NULL!	#NULL!	#NULL!	#NULL!
2,00	2,00	#NULL!	#NULL!	#NULL!	#NULL!	#NULL!
2,00	2,00	#NULL!	#NULL!	#NULL!	#NULL!	#NULL!
2,00	2,00	#NULL!	#NULL!	#NULL!	#NULL!	#NULL!
2,00	2,00	#NULL!	#NULL!	#NULL!	#NULL!	#NULL!
2,00	2,00	#NULL!	#NULL!	#NULL!	#NULL!	#NULL!
2,00	2,00	#NULL!	#NULL!	#NULL!	#NULL!	#NULL!
2,00	2,00	#NULL!	#NULL!	#NULL!	#NULL!	#NULL!
2,00	2,00	#NULL!	#NULL!	#NULL!	#NULL!	#NULL!
2,00	2,00	#NULL!	#NULL!	#NULL!	#NULL!	#NULL!
2,00	2,00	#NULL!	#NULL!	#NULL!	#NULL!	#NULL!
2,00	2,00	#NULL!	#NULL!	#NULL!	#NULL!	#NULL!
2,00	2,00	#NULL!	#NULL!	#NULL!	#NULL!	#NULL!
2,00	2,00	#NULL!	#NULL!	#NULL!	#NULL!	#NULL!
2,00	2,00	#NULL!	#NULL!	#NULL!	#NULL!	#NULL!

2,00	2,00	#NULL!	#NULL!	#NULL!	#NULL!	#NULL!
2,00	2,00	#NULL!	#NULL!	#NULL!	#NULL!	#NULL!
2,00	2,00	#NULL!	#NULL!	#NULL!	#NULL!	#NULL!
2,00	2,00	#NULL!	#NULL!	#NULL!	#NULL!	#NULL!
2,00	2,00	#NULL!	#NULL!	#NULL!	#NULL!	#NULL!
2,00	2,00	#NULL!	#NULL!	#NULL!	#NULL!	#NULL!
2,00	2,00	#NULL!	#NULL!	#NULL!	#NULL!	#NULL!
2,00	2,00	#NULL!	#NULL!	#NULL!	#NULL!	#NULL!
2,00	2,00	#NULL!	#NULL!	#NULL!	#NULL!	#NULL!
2,00	2,00	#NULL!	#NULL!	#NULL!	#NULL!	#NULL!
2,00	2,00	#NULL!	#NULL!	#NULL!	#NULL!	#NULL!
2,00	2,00	#NULL!	#NULL!	#NULL!	#NULL!	#NULL!
2,00	2,00	#NULL!	#NULL!	#NULL!	#NULL!	#NULL!
2,00	2,00	#NULL!	#NULL!	#NULL!	#NULL!	#NULL!
2,00	2,00	#NULL!	#NULL!	#NULL!	#NULL!	#NULL!
2,00	2,00	#NULL!	#NULL!	#NULL!	#NULL!	#NULL!
2,00	2,00	#NULL!	#NULL!	#NULL!	#NULL!	#NULL!
2,00	2,00	#NULL!	#NULL!	#NULL!	#NULL!	#NULL!
2,00	2,00	#NULL!	#NULL!	#NULL!	#NULL!	#NULL!
2,00	2,00	#NULL!	#NULL!	#NULL!	#NULL!	#NULL!
2,00	2,00	#NULL!	#NULL!	#NULL!	#NULL!	#NULL!
2,00	2,00	#NULL!	#NULL!	#NULL!	#NULL!	#NULL!
2,00	2,00	#NULL!	#NULL!	#NULL!	#NULL!	#NULL!
2,00	2,00	#NULL!	#NULL!	#NULL!	#NULL!	#NULL!
2,00	2,00	#NULL!	#NULL!	#NULL!	#NULL!	#NULL!
1,00	2,00	#NULL!	#NULL!	#NULL!	#NULL!	#NULL!
2,00	2,00	#NULL!	#NULL!	#NULL!	#NULL!	#NULL!
2,00	2,00	#NULL!	#NULL!	#NULL!	#NULL!	#NULL!
2,00	2,00	#NULL!	#NULL!	#NULL!	#NULL!	#NULL!
2,00	2,00	#NULL!	#NULL!	#NULL!	#NULL!	#NULL!
2,00	2,00	#NULL!	#NULL!	#NULL!	#NULL!	#NULL!
2,00	2,00	#NULL!	#NULL!	#NULL!	#NULL!	#NULL!
2,00	2,00	#NULL!	#NULL!	#NULL!	#NULL!	#NULL!
2,00	2,00	#NULL!	#NULL!	#NULL!	#NULL!	#NULL!
2,00	2,00	#NULL!	#NULL!	#NULL!	#NULL!	#NULL!
2,00	2,00	#NULL!	#NULL!	#NULL!	#NULL!	#NULL!
2,00	2,00	#NULL!	#NULL!	#NULL!	#NULL!	#NULL!
2,00	2,00	#NULL!	#NULL!	#NULL!	#NULL!	#NULL!
2,00	2,00	#NULL!	#NULL!	#NULL!	#NULL!	#NULL!
1,00	2,00	#NULL!	#NULL!	#NULL!	#NULL!	#NULL!
2,00	2,00	#NULL!	#NULL!	#NULL!	#NULL!	#NULL!
2,00	2,00	#NULL!	#NULL!	#NULL!	#NULL!	#NULL!
2,00	2,00	#NULL!	#NULL!	#NULL!	#NULL!	#NULL!
2,00	2,00	#NULL!	#NULL!	#NULL!	#NULL!	#NULL!
2,00	2,00	#NULL!	#NULL!	#NULL!	#NULL!	#NULL!
2,00	2,00	#NULL!	#NULL!	#NULL!	#NULL!	#NULL!
2,00	2,00	#NULL!	#NULL!	#NULL!	#NULL!	#NULL!
2,00	2,00	#NULL!	#NULL!	#NULL!	#NULL!	#NULL!
2,00	2,00	#NULL!	#NULL!	#NULL!	#NULL!	#NULL!
2,00	2,00	#NULL!	#NULL!	#NULL!	#NULL!	#NULL!

2,00	2,00	#NULL!	#NULL!	#NULL!	#NULL!	#NULL!
2,00	2,00	#NULL!	#NULL!	#NULL!	#NULL!	#NULL!
2,00	2,00	#NULL!	#NULL!	#NULL!	#NULL!	#NULL!
2,00	2,00	#NULL!	#NULL!	#NULL!	#NULL!	#NULL!
2,00	2,00	#NULL!	#NULL!	#NULL!	#NULL!	#NULL!
2,00	2,00	#NULL!	#NULL!	#NULL!	#NULL!	#NULL!
2,00	2,00	#NULL!	#NULL!	#NULL!	#NULL!	#NULL!
2,00	2,00	#NULL!	#NULL!	#NULL!	#NULL!	#NULL!
2,00	2,00	#NULL!	#NULL!	#NULL!	#NULL!	#NULL!
2,00	2,00	#NULL!	#NULL!	#NULL!	#NULL!	#NULL!
2,00	2,00	#NULL!	#NULL!	#NULL!	#NULL!	#NULL!
2,00	2,00	#NULL!	#NULL!	#NULL!	#NULL!	#NULL!
2,00	2,00	#NULL!	#NULL!	#NULL!	#NULL!	#NULL!
2,00	2,00	#NULL!	#NULL!	#NULL!	#NULL!	#NULL!
2,00	2,00	#NULL!	#NULL!	#NULL!	#NULL!	#NULL!
2,00	2,00	#NULL!	#NULL!	#NULL!	#NULL!	#NULL!
2,00	2,00	#NULL!	#NULL!	#NULL!	#NULL!	#NULL!
2,00	2,00	#NULL!	#NULL!	#NULL!	#NULL!	#NULL!
2,00	2,00	#NULL!	#NULL!	#NULL!	#NULL!	#NULL!
2,00	2,00	#NULL!	#NULL!	#NULL!	#NULL!	#NULL!
2,00	2,00	#NULL!	#NULL!	#NULL!	#NULL!	#NULL!
2,00	2,00	#NULL!	#NULL!	#NULL!	#NULL!	#NULL!
2,00	2,00	#NULL!	#NULL!	#NULL!	#NULL!	#NULL!
2,00	2,00	#NULL!	#NULL!	#NULL!	#NULL!	#NULL!
2,00	2,00	#NULL!	#NULL!	#NULL!	#NULL!	#NULL!
2,00	2,00	#NULL!	#NULL!	#NULL!	#NULL!	#NULL!
2,00	2,00	#NULL!	#NULL!	#NULL!	#NULL!	#NULL!
2,00	2,00	#NULL!	#NULL!	#NULL!	#NULL!	#NULL!
2,00	2,00	#NULL!	#NULL!	#NULL!	#NULL!	#NULL!
2,00	2,00	#NULL!	#NULL!	#NULL!	#NULL!	#NULL!
2,00	2,00	#NULL!	#NULL!	#NULL!	#NULL!	#NULL!
2,00	2,00	#NULL!	#NULL!	#NULL!	#NULL!	#NULL!
2,00	2,00	#NULL!	#NULL!	#NULL!	#NULL!	#NULL!
2,00	2,00	#NULL!	#NULL!	#NULL!	#NULL!	#NULL!
2,00	2,00	#NULL!	#NULL!	#NULL!	#NULL!	#NULL!
2,00	2,00	#NULL!	#NULL!	#NULL!	#NULL!	#NULL!
2,00	2,00	#NULL!	#NULL!	#NULL!	#NULL!	#NULL!
2,00	2,00	#NULL!	#NULL!	#NULL!	#NULL!	#NULL!
2,00	2,00	#NULL!	#NULL!	#NULL!	#NULL!	#NULL!
2,00	2,00	#NULL!	#NULL!	#NULL!	#NULL!	#NULL!
2,00	2,00	#NULL!	#NULL!	#NULL!	#NULL!	#NULL!
2,00	2,00	#NULL!	#NULL!	#NULL!	#NULL!	#NULL!
2,00	2,00	#NULL!	#NULL!	#NULL!	#NULL!	#NULL!
2,00	2,00	#NULL!	#NULL!	#NULL!	#NULL!	#NULL!
2,00	2,00	#NULL!	#NULL!	#NULL!	#NULL!	#NULL!
2,00	2,00	#NULL!	#NULL!	#NULL!	#NULL!	#NULL!
2,00	2,00	#NULL!	#NULL!	#NULL!	#NULL!	#NULL!
2,00	2,00	#NULL!	#NULL!	#NULL!	#NULL!	#NULL!
2,00	2,00	#NULL!	#NULL!	#NULL!	#NULL!	#NULL!
2,00	2,00	#NULL!	#NULL!	#NULL!	#NULL!	#NULL!

2,00	2,00	#NULL!	#NULL!	#NULL!	#NULL!	#NULL!
2,00	2,00	#NULL!	#NULL!	#NULL!	#NULL!	#NULL!
2,00	2,00	#NULL!	#NULL!	#NULL!	#NULL!	#NULL!
2,00	2,00	#NULL!	#NULL!	#NULL!	#NULL!	#NULL!
2,00	2,00	#NULL!	#NULL!	#NULL!	#NULL!	#NULL!
2,00	2,00	#NULL!	#NULL!	#NULL!	#NULL!	#NULL!
2,00	2,00	#NULL!	#NULL!	#NULL!	#NULL!	#NULL!
2,00	2,00	#NULL!	#NULL!	#NULL!	#NULL!	#NULL!
2,00	2,00	#NULL!	#NULL!	#NULL!	#NULL!	#NULL!
2,00	2,00	#NULL!	#NULL!	#NULL!	#NULL!	#NULL!
2,00	2,00	#NULL!	#NULL!	#NULL!	#NULL!	#NULL!
2,00	2,00	#NULL!	#NULL!	#NULL!	#NULL!	#NULL!
2,00	2,00	#NULL!	#NULL!	#NULL!	#NULL!	#NULL!
2,00	2,00	#NULL!	#NULL!	#NULL!	#NULL!	#NULL!
2,00	2,00	#NULL!	#NULL!	#NULL!	#NULL!	#NULL!
2,00	2,00	#NULL!	#NULL!	#NULL!	#NULL!	#NULL!
2,00	2,00	#NULL!	#NULL!	#NULL!	#NULL!	#NULL!
2,00	2,00	#NULL!	#NULL!	#NULL!	#NULL!	#NULL!
2,00	2,00	#NULL!	#NULL!	#NULL!	#NULL!	#NULL!
2,00	2,00	#NULL!	#NULL!	#NULL!	#NULL!	#NULL!
2,00	2,00	#NULL!	#NULL!	#NULL!	#NULL!	#NULL!
2,00	2,00	#NULL!	#NULL!	#NULL!	#NULL!	#NULL!
2,00	2,00	#NULL!	#NULL!	#NULL!	#NULL!	#NULL!
2,00	2,00	#NULL!	#NULL!	#NULL!	#NULL!	#NULL!
2,00	2,00	#NULL!	#NULL!	#NULL!	#NULL!	#NULL!
2,00	2,00	#NULL!	#NULL!	#NULL!	#NULL!	#NULL!
2,00	2,00	#NULL!	#NULL!	#NULL!	#NULL!	#NULL!
2,00	2,00	#NULL!	#NULL!	#NULL!	#NULL!	#NULL!
2,00	2,00	#NULL!	#NULL!	#NULL!	#NULL!	#NULL!
2,00	2,00	#NULL!	#NULL!	#NULL!	#NULL!	#NULL!
2,00	2,00	#NULL!	#NULL!	#NULL!	#NULL!	#NULL!
2,00	2,00	#NULL!	#NULL!	#NULL!	#NULL!	#NULL!
2,00	2,00	#NULL!	#NULL!	#NULL!	#NULL!	#NULL!
2,00	2,00	#NULL!	#NULL!	#NULL!	#NULL!	#NULL!
2,00	2,00	#NULL!	#NULL!	#NULL!	#NULL!	#NULL!
2,00	2,00	#NULL!	#NULL!	#NULL!	#NULL!	#NULL!
2,00	2,00	#NULL!	#NULL!	#NULL!	#NULL!	#NULL!
2,00	2,00	#NULL!	#NULL!	#NULL!	#NULL!	#NULL!
2,00	2,00	#NULL!	#NULL!	#NULL!	#NULL!	#NULL!
2,00	2,00	#NULL!	#NULL!	#NULL!	#NULL!	#NULL!
1,00	2,00	#NULL!	#NULL!	#NULL!	#NULL!	#NULL!
2,00	2,00	#NULL!	#NULL!	#NULL!	#NULL!	#NULL!
2,00	2,00	#NULL!	#NULL!	#NULL!	#NULL!	#NULL!
2,00	2,00	#NULL!	#NULL!	#NULL!	#NULL!	#NULL!
2,00	2,00	#NULL!	#NULL!	#NULL!	#NULL!	#NULL!
2,00	2,00	#NULL!	#NULL!	#NULL!	#NULL!	#NULL!
2,00	2,00	#NULL!	#NULL!	#NULL!	#NULL!	#NULL!
2,00	2,00	#NULL!	#NULL!	#NULL!	#NULL!	#NULL!
2,00	2,00	#NULL!	#NULL!	#NULL!	#NULL!	#NULL!
2,00	2,00	#NULL!	#NULL!	#NULL!	#NULL!	#NULL!

2,00	2,00	#NULL!	#NULL!	#NULL!	#NULL!	#NULL!
2,00	2,00	#NULL!	#NULL!	#NULL!	#NULL!	#NULL!
2,00	2,00	#NULL!	#NULL!	#NULL!	#NULL!	#NULL!
2,00	2,00	#NULL!	#NULL!	#NULL!	#NULL!	#NULL!
2,00	2,00	#NULL!	#NULL!	#NULL!	#NULL!	#NULL!
2,00	2,00	#NULL!	#NULL!	#NULL!	#NULL!	#NULL!
2,00	2,00	#NULL!	#NULL!	#NULL!	#NULL!	#NULL!
2,00	2,00	#NULL!	#NULL!	#NULL!	#NULL!	#NULL!
2,00	2,00	#NULL!	#NULL!	#NULL!	#NULL!	#NULL!
2,00	2,00	#NULL!	#NULL!	#NULL!	#NULL!	#NULL!
2,00	2,00	#NULL!	#NULL!	#NULL!	#NULL!	#NULL!
2,00	2,00	#NULL!	#NULL!	#NULL!	#NULL!	#NULL!
2,00	2,00	#NULL!	#NULL!	#NULL!	#NULL!	#NULL!
2,00	2,00	#NULL!	#NULL!	#NULL!	#NULL!	#NULL!
2,00	2,00	#NULL!	#NULL!	#NULL!	#NULL!	#NULL!
2,00	2,00	#NULL!	#NULL!	#NULL!	#NULL!	#NULL!
2,00	2,00	#NULL!	#NULL!	#NULL!	#NULL!	#NULL!
2,00	2,00	#NULL!	#NULL!	#NULL!	#NULL!	#NULL!
2,00	2,00	#NULL!	#NULL!	#NULL!	#NULL!	#NULL!
2,00	2,00	#NULL!	#NULL!	#NULL!	#NULL!	#NULL!
2,00	2,00	#NULL!	#NULL!	#NULL!	#NULL!	#NULL!
2,00	2,00	#NULL!	#NULL!	#NULL!	#NULL!	#NULL!
2,00	2,00	#NULL!	#NULL!	#NULL!	#NULL!	#NULL!
2,00	2,00	#NULL!	#NULL!	#NULL!	#NULL!	#NULL!
2,00	2,00	#NULL!	#NULL!	#NULL!	#NULL!	#NULL!
2,00	2,00	#NULL!	#NULL!	#NULL!	#NULL!	#NULL!
2,00	2,00	#NULL!	#NULL!	#NULL!	#NULL!	#NULL!
2,00	2,00	#NULL!	#NULL!	#NULL!	#NULL!	#NULL!
2,00	2,00	#NULL!	#NULL!	#NULL!	#NULL!	#NULL!
2,00	2,00	#NULL!	#NULL!	#NULL!	#NULL!	#NULL!
2,00	2,00	#NULL!	#NULL!	#NULL!	#NULL!	#NULL!
2,00	2,00	#NULL!	#NULL!	#NULL!	#NULL!	#NULL!
2,00	2,00	#NULL!	#NULL!	#NULL!	#NULL!	#NULL!
2,00	2,00	#NULL!	#NULL!	#NULL!	#NULL!	#NULL!
2,00	2,00	#NULL!	#NULL!	#NULL!	#NULL!	#NULL!
2,00	2,00	#NULL!	#NULL!	#NULL!	#NULL!	#NULL!
2,00	2,00	#NULL!	#NULL!	#NULL!	#NULL!	#NULL!
2,00	2,00	#NULL!	#NULL!	#NULL!	#NULL!	#NULL!
2,00	2,00	#NULL!	#NULL!	#NULL!	#NULL!	#NULL!
2,00	2,00	#NULL!	#NULL!	#NULL!	#NULL!	#NULL!
2,00	2,00	#NULL!	#NULL!	#NULL!	#NULL!	#NULL!
2,00	2,00	#NULL!	#NULL!	#NULL!	#NULL!	#NULL!
2,00	2,00	#NULL!	#NULL!	#NULL!	#NULL!	#NULL!
2,00	2,00	#NULL!	#NULL!	#NULL!	#NULL!	#NULL!
2,00	2,00	#NULL!	#NULL!	#NULL!	#NULL!	#NULL!
2,00	2,00	#NULL!	#NULL!	#NULL!	#NULL!	#NULL!
2,00	2,00	#NULL!	#NULL!	#NULL!	#NULL!	#NULL!
2,00	2,00	#NULL!	#NULL!	#NULL!	#NULL!	#NULL!
2,00	2,00	#NULL!	#NULL!	#NULL!	#NULL!	#NULL!
2,00	2,00	#NULL!	#NULL!	#NULL!	#NULL!	#NULL!

2,00	2,00	#NULL!	#NULL!	#NULL!	#NULL!	#NULL!
2,00	2,00	#NULL!	#NULL!	#NULL!	#NULL!	#NULL!
2,00	2,00	#NULL!	#NULL!	#NULL!	#NULL!	#NULL!
2,00	2,00	#NULL!	#NULL!	#NULL!	#NULL!	#NULL!
2,00	2,00	#NULL!	#NULL!	#NULL!	#NULL!	#NULL!
2,00	2,00	#NULL!	#NULL!	#NULL!	#NULL!	#NULL!
2,00	2,00	#NULL!	#NULL!	#NULL!	#NULL!	#NULL!
2,00	2,00	#NULL!	#NULL!	#NULL!	#NULL!	#NULL!
2,00	2,00	#NULL!	#NULL!	#NULL!	#NULL!	#NULL!
2,00	2,00	#NULL!	#NULL!	#NULL!	#NULL!	#NULL!
2,00	2,00	#NULL!	#NULL!	#NULL!	#NULL!	#NULL!
2,00	2,00	#NULL!	#NULL!	#NULL!	#NULL!	#NULL!
2,00	2,00	#NULL!	#NULL!	#NULL!	#NULL!	#NULL!
2,00	2,00	#NULL!	#NULL!	#NULL!	#NULL!	#NULL!
2,00	2,00	#NULL!	#NULL!	#NULL!	#NULL!	#NULL!
2,00	2,00	#NULL!	#NULL!	#NULL!	#NULL!	#NULL!
2,00	2,00	#NULL!	#NULL!	#NULL!	#NULL!	#NULL!
2,00	2,00	#NULL!	#NULL!	#NULL!	#NULL!	#NULL!
2,00	2,00	#NULL!	#NULL!	#NULL!	#NULL!	#NULL!
2,00	2,00	#NULL!	#NULL!	#NULL!	#NULL!	#NULL!
2,00	2,00	#NULL!	#NULL!	#NULL!	#NULL!	#NULL!
2,00	2,00	#NULL!	#NULL!	#NULL!	#NULL!	#NULL!
2,00	2,00	#NULL!	#NULL!	#NULL!	#NULL!	#NULL!
2,00	2,00	#NULL!	#NULL!	#NULL!	#NULL!	#NULL!
2,00	2,00	#NULL!	#NULL!	#NULL!	#NULL!	#NULL!
2,00	2,00	#NULL!	#NULL!	#NULL!	#NULL!	#NULL!
2,00	2,00	#NULL!	#NULL!	#NULL!	#NULL!	#NULL!
2,00	2,00	#NULL!	#NULL!	#NULL!	#NULL!	#NULL!
2,00	2,00	#NULL!	#NULL!	#NULL!	#NULL!	#NULL!
2,00	2,00	#NULL!	#NULL!	#NULL!	#NULL!	#NULL!
2,00	2,00	#NULL!	#NULL!	#NULL!	#NULL!	#NULL!
2,00	2,00	#NULL!	#NULL!	#NULL!	#NULL!	#NULL!
2,00	2,00	#NULL!	#NULL!	#NULL!	#NULL!	#NULL!
2,00	2,00	#NULL!	#NULL!	#NULL!	#NULL!	#NULL!
2,00	2,00	#NULL!	#NULL!	#NULL!	#NULL!	#NULL!
2,00	2,00	#NULL!	#NULL!	#NULL!	#NULL!	#NULL!
2,00	2,00	#NULL!	#NULL!	#NULL!	#NULL!	#NULL!
2,00	2,00	#NULL!	#NULL!	#NULL!	#NULL!	#NULL!
2,00	2,00	#NULL!	#NULL!	#NULL!	#NULL!	#NULL!
2,00	2,00	#NULL!	#NULL!	#NULL!	#NULL!	#NULL!
2,00	2,00	#NULL!	#NULL!	#NULL!	#NULL!	#NULL!
2,00	2,00	#NULL!	#NULL!	#NULL!	#NULL!	#NULL!
2,00	2,00	#NULL!	#NULL!	#NULL!	#NULL!	#NULL!
2,00	2,00	#NULL!	#NULL!	#NULL!	#NULL!	#NULL!
2,00	2,00	#NULL!	#NULL!	#NULL!	#NULL!	#NULL!
2,00	2,00	#NULL!	#NULL!	#NULL!	#NULL!	#NULL!
2,00	2,00	#NULL!	#NULL!	#NULL!	#NULL!	#NULL!
2,00	2,00	#NULL!	#NULL!	#NULL!	#NULL!	#NULL!
2,00	2,00	#NULL!	#NULL!	#NULL!	#NULL!	#NULL!
2,00	2,00	#NULL!	#NULL!	#NULL!	#NULL!	#NULL!

2,00	2,00	#NULL!	#NULL!	#NULL!	#NULL!	#NULL!
2,00	2,00	#NULL!	#NULL!	#NULL!	#NULL!	#NULL!
2,00	2,00	#NULL!	#NULL!	#NULL!	#NULL!	#NULL!
2,00	2,00	#NULL!	#NULL!	#NULL!	#NULL!	#NULL!
2,00	2,00	#NULL!	#NULL!	#NULL!	#NULL!	#NULL!
2,00	2,00	#NULL!	#NULL!	#NULL!	#NULL!	#NULL!
2,00	2,00	#NULL!	#NULL!	#NULL!	#NULL!	#NULL!
2,00	2,00	#NULL!	#NULL!	#NULL!	#NULL!	#NULL!
2,00	2,00	#NULL!	#NULL!	#NULL!	#NULL!	#NULL!
2,00	2,00	#NULL!	#NULL!	#NULL!	#NULL!	#NULL!
2,00	2,00	#NULL!	#NULL!	#NULL!	#NULL!	#NULL!
2,00	2,00	#NULL!	#NULL!	#NULL!	#NULL!	#NULL!
2,00	2,00	#NULL!	#NULL!	#NULL!	#NULL!	#NULL!
2,00	2,00	#NULL!	#NULL!	#NULL!	#NULL!	#NULL!
2,00	2,00	#NULL!	#NULL!	#NULL!	#NULL!	#NULL!
2,00	2,00	#NULL!	#NULL!	#NULL!	#NULL!	#NULL!
2,00	2,00	#NULL!	#NULL!	#NULL!	#NULL!	#NULL!
2,00	2,00	#NULL!	#NULL!	#NULL!	#NULL!	#NULL!
2,00	2,00	#NULL!	#NULL!	#NULL!	#NULL!	#NULL!
2,00	1,00	1,00	2,00	1,00	2,00	#NULL!
2,00	2,00	#NULL!	#NULL!	#NULL!	#NULL!	#NULL!
2,00	2,00	#NULL!	#NULL!	#NULL!	#NULL!	#NULL!
2,00	2,00	#NULL!	#NULL!	#NULL!	#NULL!	#NULL!
2,00	1,00	1,00	1,00	2,00	2,00	#NULL!
2,00	2,00	#NULL!	#NULL!	#NULL!	#NULL!	#NULL!
2,00	2,00	#NULL!	#NULL!	#NULL!	#NULL!	#NULL!
2,00	2,00	#NULL!	#NULL!	#NULL!	#NULL!	#NULL!
2,00	1,00	1,00	1,00	2,00	2,00	#NULL!
2,00	1,00	1,00	2,00	1,00	2,00	#NULL!
2,00	2,00	#NULL!	#NULL!	#NULL!	#NULL!	#NULL!
2,00	2,00	#NULL!	#NULL!	#NULL!	#NULL!	#NULL!
2,00	2,00	#NULL!	#NULL!	#NULL!	#NULL!	#NULL!
2,00	1,00	2,00	2,00	1,00	2,00	#NULL!
2,00	2,00	#NULL!	#NULL!	#NULL!	#NULL!	#NULL!
2,00	2,00	#NULL!	#NULL!	#NULL!	#NULL!	#NULL!
2,00	1,00	2,00	1,00	2,00	2,00	#NULL!
2,00	2,00	#NULL!	#NULL!	#NULL!	#NULL!	#NULL!
2,00	2,00	#NULL!	#NULL!	#NULL!	#NULL!	#NULL!
2,00	2,00	#NULL!	#NULL!	#NULL!	#NULL!	#NULL!
2,00	2,00	#NULL!	#NULL!	#NULL!	#NULL!	#NULL!
2,00	2,00	#NULL!	#NULL!	#NULL!	#NULL!	#NULL!
2,00	2,00	#NULL!	#NULL!	#NULL!	#NULL!	#NULL!
2,00	2,00	#NULL!	#NULL!	#NULL!	#NULL!	#NULL!
2,00	2,00	#NULL!	#NULL!	#NULL!	#NULL!	#NULL!
2,00	2,00	#NULL!	#NULL!	#NULL!	#NULL!	#NULL!
2,00	2,00	#NULL!	#NULL!	#NULL!	#NULL!	#NULL!
2,00	2,00	#NULL!	#NULL!	#NULL!	#NULL!	#NULL!
2,00	2,00	#NULL!	#NULL!	#NULL!	#NULL!	#NULL!
2,00	2,00	#NULL!	#NULL!	#NULL!	#NULL!	#NULL!
2,00	2,00	#NULL!	#NULL!	#NULL!	#NULL!	#NULL!

2,00	2,00	#NULL!	#NULL!	#NULL!	#NULL!	#NULL!
2,00	2,00	#NULL!	#NULL!	#NULL!	#NULL!	#NULL!
2,00	2,00	#NULL!	#NULL!	#NULL!	#NULL!	#NULL!
1,00	2,00	#NULL!	#NULL!	#NULL!	#NULL!	#NULL!
2,00	2,00	#NULL!	#NULL!	#NULL!	#NULL!	#NULL!
2,00	2,00	#NULL!	#NULL!	#NULL!	#NULL!	#NULL!
2,00	2,00	#NULL!	#NULL!	#NULL!	#NULL!	#NULL!
2,00	2,00	#NULL!	#NULL!	#NULL!	#NULL!	#NULL!
1,00	2,00	#NULL!	#NULL!	#NULL!	#NULL!	#NULL!
2,00	2,00	#NULL!	#NULL!	#NULL!	#NULL!	#NULL!
2,00	2,00	#NULL!	#NULL!	#NULL!	#NULL!	#NULL!
2,00	2,00	#NULL!	#NULL!	#NULL!	#NULL!	#NULL!
2,00	2,00	#NULL!	#NULL!	#NULL!	#NULL!	#NULL!
2,00	2,00	#NULL!	#NULL!	#NULL!	#NULL!	#NULL!
2,00	2,00	#NULL!	#NULL!	#NULL!	#NULL!	#NULL!
2,00	2,00	#NULL!	#NULL!	#NULL!	#NULL!	#NULL!
2,00	2,00	#NULL!	#NULL!	#NULL!	#NULL!	#NULL!
2,00	2,00	#NULL!	#NULL!	#NULL!	#NULL!	#NULL!
2,00	2,00	#NULL!	#NULL!	#NULL!	#NULL!	#NULL!
2,00	2,00	#NULL!	#NULL!	#NULL!	#NULL!	#NULL!
2,00	2,00	#NULL!	#NULL!	#NULL!	#NULL!	#NULL!
2,00	2,00	#NULL!	#NULL!	#NULL!	#NULL!	#NULL!
2,00	2,00	#NULL!	#NULL!	#NULL!	#NULL!	#NULL!
2,00	2,00	#NULL!	#NULL!	#NULL!	#NULL!	#NULL!
2,00	2,00	#NULL!	#NULL!	#NULL!	#NULL!	#NULL!
2,00	2,00	#NULL!	#NULL!	#NULL!	#NULL!	#NULL!
2,00	2,00	#NULL!	#NULL!	#NULL!	#NULL!	#NULL!
2,00	2,00	#NULL!	#NULL!	#NULL!	#NULL!	#NULL!
2,00	2,00	#NULL!	#NULL!	#NULL!	#NULL!	#NULL!
2,00	2,00	#NULL!	#NULL!	#NULL!	#NULL!	#NULL!
2,00	2,00	#NULL!	#NULL!	#NULL!	#NULL!	#NULL!
1,00	2,00	#NULL!	#NULL!	#NULL!	#NULL!	#NULL!
2,00	2,00	#NULL!	#NULL!	#NULL!	#NULL!	#NULL!
2,00	2,00	#NULL!	#NULL!	#NULL!	#NULL!	#NULL!
2,00	2,00	#NULL!	#NULL!	#NULL!	#NULL!	#NULL!
2,00	2,00	#NULL!	#NULL!	#NULL!	#NULL!	#NULL!
2,00	2,00	#NULL!	#NULL!	#NULL!	#NULL!	#NULL!
2,00	2,00	#NULL!	#NULL!	#NULL!	#NULL!	#NULL!
2,00	2,00	#NULL!	#NULL!	#NULL!	#NULL!	#NULL!
2,00	2,00	#NULL!	#NULL!	#NULL!	#NULL!	#NULL!
2,00	2,00	#NULL!	#NULL!	#NULL!	#NULL!	#NULL!
2,00	2,00	#NULL!	#NULL!	#NULL!	#NULL!	#NULL!
2,00	2,00	#NULL!	#NULL!	#NULL!	#NULL!	#NULL!
2,00	2,00	#NULL!	#NULL!	#NULL!	#NULL!	#NULL!
1,00	2,00	#NULL!	#NULL!	#NULL!	#NULL!	#NULL!
2,00	2,00	#NULL!	#NULL!	#NULL!	#NULL!	#NULL!
2,00	2,00	#NULL!	#NULL!	#NULL!	#NULL!	#NULL!
2,00	2,00	#NULL!	#NULL!	#NULL!	#NULL!	#NULL!
2,00	2,00	#NULL!	#NULL!	#NULL!	#NULL!	#NULL!
2,00	2,00	#NULL!	#NULL!	#NULL!	#NULL!	#NULL!

2,00	2,00	#NULL!	#NULL!	#NULL!	#NULL!	#NULL!
2,00	2,00	#NULL!	#NULL!	#NULL!	#NULL!	#NULL!
2,00	2,00	#NULL!	#NULL!	#NULL!	#NULL!	#NULL!
2,00	2,00	#NULL!	#NULL!	#NULL!	#NULL!	#NULL!
2,00	2,00	#NULL!	#NULL!	#NULL!	#NULL!	#NULL!
2,00	2,00	#NULL!	#NULL!	#NULL!	#NULL!	#NULL!
2,00	2,00	#NULL!	#NULL!	#NULL!	#NULL!	#NULL!
2,00	2,00	#NULL!	#NULL!	#NULL!	#NULL!	#NULL!
2,00	2,00	#NULL!	#NULL!	#NULL!	#NULL!	#NULL!
2,00	2,00	#NULL!	#NULL!	#NULL!	#NULL!	#NULL!
1,00	2,00	#NULL!	#NULL!	#NULL!	#NULL!	#NULL!
2,00	2,00	#NULL!	#NULL!	#NULL!	#NULL!	#NULL!
2,00	2,00	#NULL!	#NULL!	#NULL!	#NULL!	#NULL!
2,00	2,00	#NULL!	#NULL!	#NULL!	#NULL!	#NULL!
1,00	2,00	#NULL!	#NULL!	#NULL!	#NULL!	#NULL!
2,00	2,00	#NULL!	#NULL!	#NULL!	#NULL!	#NULL!
2,00	2,00	#NULL!	#NULL!	#NULL!	#NULL!	#NULL!
2,00	2,00	#NULL!	#NULL!	#NULL!	#NULL!	#NULL!
2,00	2,00	#NULL!	#NULL!	#NULL!	#NULL!	#NULL!
2,00	2,00	#NULL!	#NULL!	#NULL!	#NULL!	#NULL!
2,00	2,00	#NULL!	#NULL!	#NULL!	#NULL!	#NULL!
2,00	2,00	#NULL!	#NULL!	#NULL!	#NULL!	#NULL!
2,00	2,00	#NULL!	#NULL!	#NULL!	#NULL!	#NULL!
2,00	2,00	#NULL!	#NULL!	#NULL!	#NULL!	#NULL!
1,00	2,00	#NULL!	#NULL!	#NULL!	#NULL!	#NULL!
2,00	2,00	#NULL!	#NULL!	#NULL!	#NULL!	#NULL!
2,00	2,00	#NULL!	#NULL!	#NULL!	#NULL!	#NULL!
2,00	2,00	#NULL!	#NULL!	#NULL!	#NULL!	#NULL!
2,00	2,00	#NULL!	#NULL!	#NULL!	#NULL!	#NULL!
1,00	2,00	#NULL!	#NULL!	#NULL!	#NULL!	#NULL!
2,00	2,00	#NULL!	#NULL!	#NULL!	#NULL!	#NULL!
2,00	2,00	#NULL!	#NULL!	#NULL!	#NULL!	#NULL!
2,00	2,00	#NULL!	#NULL!	#NULL!	#NULL!	#NULL!
2,00	2,00	#NULL!	#NULL!	#NULL!	#NULL!	#NULL!
2,00	2,00	#NULL!	#NULL!	#NULL!	#NULL!	#NULL!
2,00	2,00	#NULL!	#NULL!	#NULL!	#NULL!	#NULL!
2,00	2,00	#NULL!	#NULL!	#NULL!	#NULL!	#NULL!
2,00	2,00	#NULL!	#NULL!	#NULL!	#NULL!	#NULL!
2,00	2,00	#NULL!	#NULL!	#NULL!	#NULL!	#NULL!
2,00	2,00	#NULL!	#NULL!	#NULL!	#NULL!	#NULL!
2,00	2,00	#NULL!	#NULL!	#NULL!	#NULL!	#NULL!
2,00	2,00	#NULL!	#NULL!	#NULL!	#NULL!	#NULL!
2,00	2,00	#NULL!	#NULL!	#NULL!	#NULL!	#NULL!
2,00	2,00	#NULL!	#NULL!	#NULL!	#NULL!	#NULL!
2,00	2,00	#NULL!	#NULL!	#NULL!	#NULL!	#NULL!
2,00	2,00	#NULL!	#NULL!	#NULL!	#NULL!	#NULL!
2,00	2,00	#NULL!	#NULL!	#NULL!	#NULL!	#NULL!
2,00	2,00	#NULL!	#NULL!	#NULL!	#NULL!	#NULL!
2,00	2,00	#NULL!	#NULL!	#NULL!	#NULL!	#NULL!
2,00	2,00	#NULL!	#NULL!	#NULL!	#NULL!	#NULL!

2,00	2,00	#NULL!	#NULL!	#NULL!	#NULL!	#NULL!
2,00	2,00	#NULL!	#NULL!	#NULL!	#NULL!	#NULL!
1,00	2,00	#NULL!	#NULL!	#NULL!	#NULL!	#NULL!
2,00	2,00	#NULL!	#NULL!	#NULL!	#NULL!	#NULL!
2,00	2,00	#NULL!	#NULL!	#NULL!	#NULL!	#NULL!
2,00	2,00	#NULL!	#NULL!	#NULL!	#NULL!	#NULL!
2,00	2,00	#NULL!	#NULL!	#NULL!	#NULL!	#NULL!
2,00	2,00	#NULL!	#NULL!	#NULL!	#NULL!	#NULL!
2,00	2,00	#NULL!	#NULL!	#NULL!	#NULL!	#NULL!
2,00	1,00	1,00	1,00	2,00	2,00	#NULL!
2,00	2,00	#NULL!	#NULL!	#NULL!	#NULL!	#NULL!
2,00	1,00	1,00	1,00	2,00	1,00	#NULL!
1,00	2,00	#NULL!	#NULL!	#NULL!	#NULL!	#NULL!
2,00	2,00	#NULL!	#NULL!	#NULL!	#NULL!	#NULL!
2,00	2,00	#NULL!	#NULL!	#NULL!	#NULL!	#NULL!
2,00	1,00	2,00	1,00	2,00	2,00	#NULL!
2,00	2,00	#NULL!	#NULL!	#NULL!	#NULL!	#NULL!
2,00	2,00	#NULL!	#NULL!	#NULL!	#NULL!	#NULL!
2,00	2,00	#NULL!	#NULL!	#NULL!	#NULL!	#NULL!
2,00	1,00	1,00	1,00	2,00	2,00	#NULL!
2,00	2,00	#NULL!	#NULL!	#NULL!	#NULL!	#NULL!
2,00	2,00	#NULL!	#NULL!	#NULL!	#NULL!	#NULL!
2,00	2,00	#NULL!	#NULL!	#NULL!	#NULL!	#NULL!
2,00	2,00	#NULL!	#NULL!	#NULL!	#NULL!	#NULL!
2,00	2,00	#NULL!	#NULL!	#NULL!	#NULL!	#NULL!
2,00	2,00	#NULL!	#NULL!	#NULL!	#NULL!	#NULL!
2,00	2,00	#NULL!	#NULL!	#NULL!	#NULL!	#NULL!
2,00	2,00	#NULL!	#NULL!	#NULL!	#NULL!	#NULL!
2,00	2,00	#NULL!	#NULL!	#NULL!	#NULL!	#NULL!
2,00	2,00	#NULL!	#NULL!	#NULL!	#NULL!	#NULL!
2,00	2,00	#NULL!	#NULL!	#NULL!	#NULL!	#NULL!
2,00	2,00	#NULL!	#NULL!	#NULL!	#NULL!	#NULL!
2,00	2,00	#NULL!	#NULL!	#NULL!	#NULL!	#NULL!
2,00	2,00	#NULL!	#NULL!	#NULL!	#NULL!	#NULL!
2,00	2,00	#NULL!	#NULL!	#NULL!	#NULL!	#NULL!
2,00	2,00	#NULL!	#NULL!	#NULL!	#NULL!	#NULL!
2,00	2,00	#NULL!	#NULL!	#NULL!	#NULL!	#NULL!
2,00	2,00	#NULL!	#NULL!	#NULL!	#NULL!	#NULL!
2,00	2,00	#NULL!	#NULL!	#NULL!	#NULL!	#NULL!
2,00	2,00	#NULL!	#NULL!	#NULL!	#NULL!	#NULL!
2,00	2,00	#NULL!	#NULL!	#NULL!	#NULL!	#NULL!
2,00	2,00	#NULL!	#NULL!	#NULL!	#NULL!	#NULL!
2,00	2,00	#NULL!	#NULL!	#NULL!	#NULL!	#NULL!
2,00	2,00	#NULL!	#NULL!	#NULL!	#NULL!	#NULL!
2,00	2,00	#NULL!	#NULL!	#NULL!	#NULL!	#NULL!
2,00	2,00	#NULL!	#NULL!	#NULL!	#NULL!	#NULL!
2,00	2,00	#NULL!	#NULL!	#NULL!	#NULL!	#NULL!
2,00	2,00	#NULL!	#NULL!	#NULL!	#NULL!	#NULL!
2,00	2,00	#NULL!	#NULL!	#NULL!	#NULL!	#NULL!
2,00	2,00	#NULL!	#NULL!	#NULL!	#NULL!	#NULL!

2,00	2,00	#NULL!	#NULL!	#NULL!	#NULL!	#NULL!
2,00	2,00	#NULL!	#NULL!	#NULL!	#NULL!	#NULL!
2,00	2,00	#NULL!	#NULL!	#NULL!	#NULL!	#NULL!
2,00	2,00	#NULL!	#NULL!	#NULL!	#NULL!	#NULL!
2,00	2,00	#NULL!	#NULL!	#NULL!	#NULL!	#NULL!
2,00	2,00	#NULL!	#NULL!	#NULL!	#NULL!	#NULL!
2,00	2,00	#NULL!	#NULL!	#NULL!	#NULL!	#NULL!
2,00	2,00	#NULL!	#NULL!	#NULL!	#NULL!	#NULL!
2,00	2,00	#NULL!	#NULL!	#NULL!	#NULL!	#NULL!
2,00	2,00	#NULL!	#NULL!	#NULL!	#NULL!	#NULL!
2,00	2,00	#NULL!	#NULL!	#NULL!	#NULL!	#NULL!
2,00	2,00	#NULL!	#NULL!	#NULL!	#NULL!	#NULL!
2,00	2,00	#NULL!	#NULL!	#NULL!	#NULL!	#NULL!
2,00	2,00	#NULL!	#NULL!	#NULL!	#NULL!	#NULL!
2,00	2,00	#NULL!	#NULL!	#NULL!	#NULL!	#NULL!
2,00	2,00	#NULL!	#NULL!	#NULL!	#NULL!	#NULL!
2,00	2,00	#NULL!	#NULL!	#NULL!	#NULL!	#NULL!
2,00	2,00	#NULL!	#NULL!	#NULL!	#NULL!	#NULL!
2,00	2,00	#NULL!	#NULL!	#NULL!	#NULL!	#NULL!
2,00	2,00	#NULL!	#NULL!	#NULL!	#NULL!	#NULL!
2,00	2,00	#NULL!	#NULL!	#NULL!	#NULL!	#NULL!
2,00	2,00	#NULL!	#NULL!	#NULL!	#NULL!	#NULL!
2,00	2,00	#NULL!	#NULL!	#NULL!	#NULL!	#NULL!
2,00	2,00	#NULL!	#NULL!	#NULL!	#NULL!	#NULL!
2,00	2,00	#NULL!	#NULL!	#NULL!	#NULL!	#NULL!
2,00	2,00	#NULL!	#NULL!	#NULL!	#NULL!	#NULL!
2,00	2,00	#NULL!	#NULL!	#NULL!	#NULL!	#NULL!
2,00	2,00	#NULL!	#NULL!	#NULL!	#NULL!	#NULL!
2,00	2,00	#NULL!	#NULL!	#NULL!	#NULL!	#NULL!
2,00	2,00	#NULL!	#NULL!	#NULL!	#NULL!	#NULL!
2,00	2,00	#NULL!	#NULL!	#NULL!	#NULL!	#NULL!
2,00	2,00	#NULL!	#NULL!	#NULL!	#NULL!	#NULL!
2,00	2,00	#NULL!	#NULL!	#NULL!	#NULL!	#NULL!
2,00	2,00	#NULL!	#NULL!	#NULL!	#NULL!	#NULL!
2,00	2,00	#NULL!	#NULL!	#NULL!	#NULL!	#NULL!
2,00	2,00	#NULL!	#NULL!	#NULL!	#NULL!	#NULL!
2,00	2,00	#NULL!	#NULL!	#NULL!	#NULL!	#NULL!
2,00	2,00	#NULL!	#NULL!	#NULL!	#NULL!	#NULL!
2,00	2,00	#NULL!	#NULL!	#NULL!	#NULL!	#NULL!
2,00	2,00	#NULL!	#NULL!	#NULL!	#NULL!	#NULL!
2,00	2,00	#NULL!	#NULL!	#NULL!	#NULL!	#NULL!
2,00	2,00	#NULL!	#NULL!	#NULL!	#NULL!	#NULL!
2,00	2,00	#NULL!	#NULL!	#NULL!	#NULL!	#NULL!
2,00	2,00	#NULL!	#NULL!	#NULL!	#NULL!	#NULL!
2,00	2,00	#NULL!	#NULL!	#NULL!	#NULL!	#NULL!
2,00	2,00	#NULL!	#NULL!	#NULL!	#NULL!	#NULL!
2,00	2,00	#NULL!	#NULL!	#NULL!	#NULL!	#NULL!
2,00	2,00	#NULL!	#NULL!	#NULL!	#NULL!	#NULL!
2,00	2,00	#NULL!	#NULL!	#NULL!	#NULL!	#NULL!
2,00	2,00	#NULL!	#NULL!	#NULL!	#NULL!	#NULL!

2,00	2,00	#NULL!	#NULL!	#NULL!	#NULL!	#NULL!
2,00	2,00	#NULL!	#NULL!	#NULL!	#NULL!	#NULL!
2,00	2,00	#NULL!	#NULL!	#NULL!	#NULL!	#NULL!
2,00	2,00	#NULL!	#NULL!	#NULL!	#NULL!	#NULL!
2,00	2,00	#NULL!	#NULL!	#NULL!	#NULL!	#NULL!
2,00	2,00	#NULL!	#NULL!	#NULL!	#NULL!	#NULL!
2,00	2,00	#NULL!	#NULL!	#NULL!	#NULL!	#NULL!
2,00	2,00	#NULL!	#NULL!	#NULL!	#NULL!	#NULL!
2,00	2,00	#NULL!	#NULL!	#NULL!	#NULL!	#NULL!
2,00	2,00	#NULL!	#NULL!	#NULL!	#NULL!	#NULL!
2,00	2,00	#NULL!	#NULL!	#NULL!	#NULL!	#NULL!
2,00	2,00	#NULL!	#NULL!	#NULL!	#NULL!	#NULL!
2,00	2,00	#NULL!	#NULL!	#NULL!	#NULL!	#NULL!
2,00	2,00	#NULL!	#NULL!	#NULL!	#NULL!	#NULL!
2,00	2,00	#NULL!	#NULL!	#NULL!	#NULL!	#NULL!
2,00	2,00	#NULL!	#NULL!	#NULL!	#NULL!	#NULL!
2,00	2,00	#NULL!	#NULL!	#NULL!	#NULL!	#NULL!
2,00	2,00	#NULL!	#NULL!	#NULL!	#NULL!	#NULL!
2,00	2,00	#NULL!	#NULL!	#NULL!	#NULL!	#NULL!
2,00	2,00	#NULL!	#NULL!	#NULL!	#NULL!	#NULL!
2,00	2,00	#NULL!	#NULL!	#NULL!	#NULL!	#NULL!
2,00	2,00	#NULL!	#NULL!	#NULL!	#NULL!	#NULL!
2,00	2,00	#NULL!	#NULL!	#NULL!	#NULL!	#NULL!
1,00	2,00	#NULL!	#NULL!	#NULL!	#NULL!	#NULL!
2,00	2,00	#NULL!	#NULL!	#NULL!	#NULL!	#NULL!
2,00	2,00	#NULL!	#NULL!	#NULL!	#NULL!	#NULL!
2,00	2,00	#NULL!	#NULL!	#NULL!	#NULL!	#NULL!
2,00	2,00	#NULL!	#NULL!	#NULL!	#NULL!	#NULL!
2,00	2,00	#NULL!	#NULL!	#NULL!	#NULL!	#NULL!
2,00	2,00	#NULL!	#NULL!	#NULL!	#NULL!	#NULL!
2,00	2,00	#NULL!	#NULL!	#NULL!	#NULL!	#NULL!
2,00	2,00	#NULL!	#NULL!	#NULL!	#NULL!	#NULL!
2,00	2,00	#NULL!	#NULL!	#NULL!	#NULL!	#NULL!
2,00	2,00	#NULL!	#NULL!	#NULL!	#NULL!	#NULL!
2,00	2,00	#NULL!	#NULL!	#NULL!	#NULL!	#NULL!
1,00	2,00	#NULL!	#NULL!	#NULL!	#NULL!	#NULL!
2,00	2,00	#NULL!	#NULL!	#NULL!	#NULL!	#NULL!
2,00	2,00	#NULL!	#NULL!	#NULL!	#NULL!	#NULL!
2,00	2,00	#NULL!	#NULL!	#NULL!	#NULL!	#NULL!
2,00	2,00	#NULL!	#NULL!	#NULL!	#NULL!	#NULL!
2,00	2,00	#NULL!	#NULL!	#NULL!	#NULL!	#NULL!
2,00	2,00	#NULL!	#NULL!	#NULL!	#NULL!	#NULL!
2,00	2,00	#NULL!	#NULL!	#NULL!	#NULL!	#NULL!
2,00	2,00	#NULL!	#NULL!	#NULL!	#NULL!	#NULL!
2,00	2,00	#NULL!	#NULL!	#NULL!	#NULL!	#NULL!
2,00	2,00	#NULL!	#NULL!	#NULL!	#NULL!	#NULL!
2,00	2,00	#NULL!	#NULL!	#NULL!	#NULL!	#NULL!
2,00	2,00	#NULL!	#NULL!	#NULL!	#NULL!	#NULL!
2,00	2,00	#NULL!	#NULL!	#NULL!	#NULL!	#NULL!
2,00	2,00	#NULL!	#NULL!	#NULL!	#NULL!	#NULL!

2,00	2,00	#NULL!	#NULL!	#NULL!	#NULL!	#NULL!
2,00	2,00	#NULL!	#NULL!	#NULL!	#NULL!	#NULL!
2,00	2,00	#NULL!	#NULL!	#NULL!	#NULL!	#NULL!
1,00	2,00	#NULL!	#NULL!	#NULL!	#NULL!	#NULL!
2,00	2,00	#NULL!	#NULL!	#NULL!	#NULL!	#NULL!
2,00	2,00	#NULL!	#NULL!	#NULL!	#NULL!	#NULL!
2,00	2,00	#NULL!	#NULL!	#NULL!	#NULL!	#NULL!
1,00	2,00	#NULL!	#NULL!	#NULL!	#NULL!	#NULL!
2,00	2,00	#NULL!	#NULL!	#NULL!	#NULL!	#NULL!
2,00	2,00	#NULL!	#NULL!	#NULL!	#NULL!	#NULL!
2,00	2,00	#NULL!	#NULL!	#NULL!	#NULL!	#NULL!
2,00	2,00	#NULL!	#NULL!	#NULL!	#NULL!	#NULL!
2,00	2,00	#NULL!	#NULL!	#NULL!	#NULL!	#NULL!
2,00	2,00	#NULL!	#NULL!	#NULL!	#NULL!	#NULL!
2,00	2,00	#NULL!	#NULL!	#NULL!	#NULL!	#NULL!
2,00	2,00	#NULL!	#NULL!	#NULL!	#NULL!	#NULL!
2,00	2,00	#NULL!	#NULL!	#NULL!	#NULL!	#NULL!
2,00	2,00	#NULL!	#NULL!	#NULL!	#NULL!	#NULL!
2,00	2,00	#NULL!	#NULL!	#NULL!	#NULL!	#NULL!
2,00	2,00	#NULL!	#NULL!	#NULL!	#NULL!	#NULL!
2,00	2,00	#NULL!	#NULL!	#NULL!	#NULL!	#NULL!
1,00	2,00	#NULL!	#NULL!	#NULL!	#NULL!	#NULL!
1,00	2,00	#NULL!	#NULL!	#NULL!	#NULL!	#NULL!
2,00	2,00	#NULL!	#NULL!	#NULL!	#NULL!	#NULL!
2,00	2,00	#NULL!	#NULL!	#NULL!	#NULL!	#NULL!
2,00	2,00	#NULL!	#NULL!	#NULL!	#NULL!	#NULL!
2,00	2,00	#NULL!	#NULL!	#NULL!	#NULL!	#NULL!
2,00	2,00	#NULL!	#NULL!	#NULL!	#NULL!	#NULL!
2,00	2,00	#NULL!	#NULL!	#NULL!	#NULL!	#NULL!
2,00	2,00	#NULL!	#NULL!	#NULL!	#NULL!	#NULL!
2,00	2,00	#NULL!	#NULL!	#NULL!	#NULL!	#NULL!
2,00	2,00	#NULL!	#NULL!	#NULL!	#NULL!	#NULL!
2,00	2,00	#NULL!	#NULL!	#NULL!	#NULL!	#NULL!
2,00	2,00	#NULL!	#NULL!	#NULL!	#NULL!	#NULL!
2,00	2,00	#NULL!	#NULL!	#NULL!	#NULL!	#NULL!
2,00	2,00	#NULL!	#NULL!	#NULL!	#NULL!	#NULL!
2,00	2,00	#NULL!	#NULL!	#NULL!	#NULL!	#NULL!
2,00	2,00	#NULL!	#NULL!	#NULL!	#NULL!	#NULL!
2,00	2,00	#NULL!	#NULL!	#NULL!	#NULL!	#NULL!
2,00	2,00	#NULL!	#NULL!	#NULL!	#NULL!	#NULL!
2,00	2,00	#NULL!	#NULL!	#NULL!	#NULL!	#NULL!
2,00	2,00	#NULL!	#NULL!	#NULL!	#NULL!	#NULL!
2,00	2,00	#NULL!	#NULL!	#NULL!	#NULL!	#NULL!
2,00	2,00	#NULL!	#NULL!	#NULL!	#NULL!	#NULL!
2,00	2,00	#NULL!	#NULL!	#NULL!	#NULL!	#NULL!
2,00	2,00	#NULL!	#NULL!	#NULL!	#NULL!	#NULL!
2,00	2,00	#NULL!	#NULL!	#NULL!	#NULL!	#NULL!
2,00	2,00	#NULL!	#NULL!	#NULL!	#NULL!	#NULL!
2,00	2,00	#NULL!	#NULL!	#NULL!	#NULL!	#NULL!
2,00	2,00	#NULL!	#NULL!	#NULL!	#NULL!	#NULL!

2,00	2,00	#NULL!	#NULL!	#NULL!	#NULL!	#NULL!
2,00	2,00	#NULL!	#NULL!	#NULL!	#NULL!	#NULL!
2,00	2,00	#NULL!	#NULL!	#NULL!	#NULL!	#NULL!
1,00	2,00	#NULL!	#NULL!	#NULL!	#NULL!	#NULL!
2,00	2,00	#NULL!	#NULL!	#NULL!	#NULL!	#NULL!
2,00	2,00	#NULL!	#NULL!	#NULL!	#NULL!	#NULL!
2,00	2,00	#NULL!	#NULL!	#NULL!	#NULL!	#NULL!
2,00	2,00	#NULL!	#NULL!	#NULL!	#NULL!	#NULL!
2,00	2,00	#NULL!	#NULL!	#NULL!	#NULL!	#NULL!
1,00	2,00	#NULL!	#NULL!	#NULL!	#NULL!	#NULL!
2,00	2,00	#NULL!	#NULL!	#NULL!	#NULL!	#NULL!
2,00	2,00	#NULL!	#NULL!	#NULL!	#NULL!	#NULL!
2,00	2,00	#NULL!	#NULL!	#NULL!	#NULL!	#NULL!
2,00	2,00	#NULL!	#NULL!	#NULL!	#NULL!	#NULL!
2,00	2,00	#NULL!	#NULL!	#NULL!	#NULL!	#NULL!
2,00	2,00	#NULL!	#NULL!	#NULL!	#NULL!	#NULL!
2,00	2,00	#NULL!	#NULL!	#NULL!	#NULL!	#NULL!
1,00	2,00	#NULL!	#NULL!	#NULL!	#NULL!	#NULL!
1,00	2,00	#NULL!	#NULL!	#NULL!	#NULL!	#NULL!
1,00	2,00	#NULL!	#NULL!	#NULL!	#NULL!	#NULL!
2,00	2,00	#NULL!	#NULL!	#NULL!	#NULL!	#NULL!
2,00	2,00	#NULL!	#NULL!	#NULL!	#NULL!	#NULL!
2,00	2,00	#NULL!	#NULL!	#NULL!	#NULL!	#NULL!
2,00	2,00	#NULL!	#NULL!	#NULL!	#NULL!	#NULL!
2,00	2,00	#NULL!	#NULL!	#NULL!	#NULL!	#NULL!
2,00	2,00	#NULL!	#NULL!	#NULL!	#NULL!	#NULL!
2,00	2,00	#NULL!	#NULL!	#NULL!	#NULL!	#NULL!
2,00	2,00	#NULL!	#NULL!	#NULL!	#NULL!	#NULL!
2,00	2,00	#NULL!	#NULL!	#NULL!	#NULL!	#NULL!
1,00	2,00	#NULL!	#NULL!	#NULL!	#NULL!	#NULL!
2,00	2,00	#NULL!	#NULL!	#NULL!	#NULL!	#NULL!
2,00	2,00	#NULL!	#NULL!	#NULL!	#NULL!	#NULL!
2,00	2,00	#NULL!	#NULL!	#NULL!	#NULL!	#NULL!
2,00	2,00	#NULL!	#NULL!	#NULL!	#NULL!	#NULL!
2,00	2,00	#NULL!	#NULL!	#NULL!	#NULL!	#NULL!
2,00	2,00	#NULL!	#NULL!	#NULL!	#NULL!	#NULL!
2,00	2,00	#NULL!	#NULL!	#NULL!	#NULL!	#NULL!
1,00	2,00	#NULL!	#NULL!	#NULL!	#NULL!	#NULL!
2,00	2,00	#NULL!	#NULL!	#NULL!	#NULL!	#NULL!
2,00	2,00	#NULL!	#NULL!	#NULL!	#NULL!	#NULL!
2,00	2,00	#NULL!	#NULL!	#NULL!	#NULL!	#NULL!
2,00	2,00	#NULL!	#NULL!	#NULL!	#NULL!	#NULL!
2,00	2,00	#NULL!	#NULL!	#NULL!	#NULL!	#NULL!
2,00	2,00	#NULL!	#NULL!	#NULL!	#NULL!	#NULL!
2,00	2,00	#NULL!	#NULL!	#NULL!	#NULL!	#NULL!
2,00	2,00	#NULL!	#NULL!	#NULL!	#NULL!	#NULL!
2,00	2,00	#NULL!	#NULL!	#NULL!	#NULL!	#NULL!
2,00	2,00	#NULL!	#NULL!	#NULL!	#NULL!	#NULL!
2,00	2,00	#NULL!	#NULL!	#NULL!	#NULL!	#NULL!
2,00	2,00	#NULL!	#NULL!	#NULL!	#NULL!	#NULL!

2,00	2,00	#NULL!	#NULL!	#NULL!	#NULL!	#NULL!
2,00	2,00	#NULL!	#NULL!	#NULL!	#NULL!	#NULL!
2,00	2,00	#NULL!	#NULL!	#NULL!	#NULL!	#NULL!
1,00	2,00	#NULL!	#NULL!	#NULL!	#NULL!	#NULL!
2,00	2,00	#NULL!	#NULL!	#NULL!	#NULL!	#NULL!
2,00	2,00	#NULL!	#NULL!	#NULL!	#NULL!	#NULL!
2,00	2,00	#NULL!	#NULL!	#NULL!	#NULL!	#NULL!
2,00	2,00	#NULL!	#NULL!	#NULL!	#NULL!	#NULL!
2,00	2,00	#NULL!	#NULL!	#NULL!	#NULL!	#NULL!
2,00	2,00	#NULL!	#NULL!	#NULL!	#NULL!	#NULL!
2,00	2,00	#NULL!	#NULL!	#NULL!	#NULL!	#NULL!
2,00	2,00	#NULL!	#NULL!	#NULL!	#NULL!	#NULL!
2,00	2,00	#NULL!	#NULL!	#NULL!	#NULL!	#NULL!
1,00	2,00	#NULL!	#NULL!	#NULL!	#NULL!	#NULL!
2,00	2,00	#NULL!	#NULL!	#NULL!	#NULL!	#NULL!
2,00	2,00	#NULL!	#NULL!	#NULL!	#NULL!	#NULL!
2,00	2,00	#NULL!	#NULL!	#NULL!	#NULL!	#NULL!
2,00	2,00	#NULL!	#NULL!	#NULL!	#NULL!	#NULL!
2,00	2,00	#NULL!	#NULL!	#NULL!	#NULL!	#NULL!
2,00	2,00	#NULL!	#NULL!	#NULL!	#NULL!	#NULL!
2,00	2,00	#NULL!	#NULL!	#NULL!	#NULL!	#NULL!
2,00	2,00	#NULL!	#NULL!	#NULL!	#NULL!	#NULL!
2,00	2,00	#NULL!	#NULL!	#NULL!	#NULL!	#NULL!
2,00	2,00	#NULL!	#NULL!	#NULL!	#NULL!	#NULL!
2,00	2,00	#NULL!	#NULL!	#NULL!	#NULL!	#NULL!
2,00	1,00	1,00	2,00	1,00	2,00	#NULL!
2,00	2,00	#NULL!	#NULL!	#NULL!	#NULL!	#NULL!
2,00	2,00	#NULL!	#NULL!	#NULL!	#NULL!	#NULL!
2,00	2,00	#NULL!	#NULL!	#NULL!	#NULL!	#NULL!
2,00	2,00	#NULL!	#NULL!	#NULL!	#NULL!	#NULL!
2,00	2,00	#NULL!	#NULL!	#NULL!	#NULL!	#NULL!
2,00	2,00	#NULL!	#NULL!	#NULL!	#NULL!	#NULL!
2,00	2,00	#NULL!	#NULL!	#NULL!	#NULL!	#NULL!
2,00	2,00	#NULL!	#NULL!	#NULL!	#NULL!	#NULL!
2,00	2,00	#NULL!	#NULL!	#NULL!	#NULL!	#NULL!
2,00	1,00	1,00	1,00	2,00	2,00	#NULL!
2,00	2,00	#NULL!	#NULL!	#NULL!	#NULL!	#NULL!
2,00	2,00	#NULL!	#NULL!	#NULL!	#NULL!	#NULL!
2,00	2,00	#NULL!	#NULL!	#NULL!	#NULL!	#NULL!
2,00	2,00	#NULL!	#NULL!	#NULL!	#NULL!	#NULL!
2,00	2,00	#NULL!	#NULL!	#NULL!	#NULL!	#NULL!
2,00	2,00	#NULL!	#NULL!	#NULL!	#NULL!	#NULL!
2,00	2,00	#NULL!	#NULL!	#NULL!	#NULL!	#NULL!
2,00	2,00	#NULL!	#NULL!	#NULL!	#NULL!	#NULL!
2,00	1,00	1,00	1,00	2,00	2,00	#NULL!
2,00	2,00	#NULL!	#NULL!	#NULL!	#NULL!	#NULL!
2,00	1,00	1,00	1,00	2,00	2,00	#NULL!
2,00	1,00	1,00	1,00	2,00	2,00	#NULL!
2,00	2,00	#NULL!	#NULL!	#NULL!	#NULL!	#NULL!
2,00	2,00	#NULL!	#NULL!	#NULL!	#NULL!	#NULL!

2,00	2,00	#NULL!	#NULL!	#NULL!	#NULL!	#NULL!
2,00	2,00	#NULL!	#NULL!	#NULL!	#NULL!	#NULL!
2,00	1,00	2,00	1,00	2,00	2,00	#NULL!
2,00	2,00	#NULL!	#NULL!	#NULL!	#NULL!	#NULL!
2,00	2,00	#NULL!	#NULL!	#NULL!	#NULL!	#NULL!
2,00	1,00	2,00	1,00	1,00	1,00	#NULL!
2,00	2,00	#NULL!	#NULL!	#NULL!	#NULL!	#NULL!
2,00	2,00	#NULL!	#NULL!	#NULL!	#NULL!	#NULL!
2,00	2,00	#NULL!	#NULL!	#NULL!	#NULL!	#NULL!
1,00	2,00	#NULL!	#NULL!	#NULL!	#NULL!	#NULL!
2,00	2,00	#NULL!	#NULL!	#NULL!	#NULL!	#NULL!
2,00	2,00	#NULL!	#NULL!	#NULL!	#NULL!	#NULL!
2,00	2,00	#NULL!	#NULL!	#NULL!	#NULL!	#NULL!
2,00	2,00	#NULL!	#NULL!	#NULL!	#NULL!	#NULL!
2,00	2,00	#NULL!	#NULL!	#NULL!	#NULL!	#NULL!
1,00	2,00	#NULL!	#NULL!	#NULL!	#NULL!	#NULL!
2,00	2,00	#NULL!	#NULL!	#NULL!	#NULL!	#NULL!
2,00	2,00	#NULL!	#NULL!	#NULL!	#NULL!	#NULL!
2,00	2,00	#NULL!	#NULL!	#NULL!	#NULL!	#NULL!
2,00	2,00	#NULL!	#NULL!	#NULL!	#NULL!	#NULL!
2,00	2,00	#NULL!	#NULL!	#NULL!	#NULL!	#NULL!
2,00	2,00	#NULL!	#NULL!	#NULL!	#NULL!	#NULL!
2,00	2,00	#NULL!	#NULL!	#NULL!	#NULL!	#NULL!
2,00	2,00	#NULL!	#NULL!	#NULL!	#NULL!	#NULL!
2,00	2,00	#NULL!	#NULL!	#NULL!	#NULL!	#NULL!
2,00	2,00	#NULL!	#NULL!	#NULL!	#NULL!	#NULL!
2,00	2,00	#NULL!	#NULL!	#NULL!	#NULL!	#NULL!
2,00	2,00	#NULL!	#NULL!	#NULL!	#NULL!	#NULL!
2,00	2,00	#NULL!	#NULL!	#NULL!	#NULL!	#NULL!
2,00	2,00	#NULL!	#NULL!	#NULL!	#NULL!	#NULL!
2,00	2,00	#NULL!	#NULL!	#NULL!	#NULL!	#NULL!
1,00	2,00	#NULL!	#NULL!	#NULL!	#NULL!	#NULL!
1,00	2,00	#NULL!	#NULL!	#NULL!	#NULL!	#NULL!
1,00	2,00	#NULL!	#NULL!	#NULL!	#NULL!	#NULL!
2,00	2,00	#NULL!	#NULL!	#NULL!	#NULL!	#NULL!
2,00	2,00	#NULL!	#NULL!	#NULL!	#NULL!	#NULL!
2,00	2,00	#NULL!	#NULL!	#NULL!	#NULL!	#NULL!
2,00	2,00	#NULL!	#NULL!	#NULL!	#NULL!	#NULL!
2,00	2,00	#NULL!	#NULL!	#NULL!	#NULL!	#NULL!
2,00	2,00	#NULL!	#NULL!	#NULL!	#NULL!	#NULL!
2,00	2,00	#NULL!	#NULL!	#NULL!	#NULL!	#NULL!
2,00	2,00	#NULL!	#NULL!	#NULL!	#NULL!	#NULL!
2,00	2,00	#NULL!	#NULL!	#NULL!	#NULL!	#NULL!
2,00	2,00	#NULL!	#NULL!	#NULL!	#NULL!	#NULL!
2,00	2,00	#NULL!	#NULL!	#NULL!	#NULL!	#NULL!
1,00	2,00	#NULL!	#NULL!	#NULL!	#NULL!	#NULL!
2,00	2,00	#NULL!	#NULL!	#NULL!	#NULL!	#NULL!
2,00	2,00	#NULL!	#NULL!	#NULL!	#NULL!	#NULL!
2,00	2,00	#NULL!	#NULL!	#NULL!	#NULL!	#NULL!
2,00	2,00	#NULL!	#NULL!	#NULL!	#NULL!	#NULL!

2,00	2,00	#NULL!	#NULL!	#NULL!	#NULL!	#NULL!
2,00	2,00	#NULL!	#NULL!	#NULL!	#NULL!	#NULL!
1,00	2,00	#NULL!	#NULL!	#NULL!	#NULL!	#NULL!
2,00	2,00	#NULL!	#NULL!	#NULL!	#NULL!	#NULL!
2,00	2,00	#NULL!	#NULL!	#NULL!	#NULL!	#NULL!
2,00	2,00	#NULL!	#NULL!	#NULL!	#NULL!	#NULL!
2,00	2,00	#NULL!	#NULL!	#NULL!	#NULL!	#NULL!
2,00	2,00	#NULL!	#NULL!	#NULL!	#NULL!	#NULL!
2,00	2,00	#NULL!	#NULL!	#NULL!	#NULL!	#NULL!
2,00	2,00	#NULL!	#NULL!	#NULL!	#NULL!	#NULL!
2,00	2,00	#NULL!	#NULL!	#NULL!	#NULL!	#NULL!
2,00	2,00	#NULL!	#NULL!	#NULL!	#NULL!	#NULL!
1,00	2,00	#NULL!	#NULL!	#NULL!	#NULL!	#NULL!
2,00	2,00	#NULL!	#NULL!	#NULL!	#NULL!	#NULL!
2,00	2,00	#NULL!	#NULL!	#NULL!	#NULL!	#NULL!
2,00	2,00	#NULL!	#NULL!	#NULL!	#NULL!	#NULL!
2,00	2,00	#NULL!	#NULL!	#NULL!	#NULL!	#NULL!
2,00	2,00	#NULL!	#NULL!	#NULL!	#NULL!	#NULL!
2,00	2,00	#NULL!	#NULL!	#NULL!	#NULL!	#NULL!
2,00	2,00	#NULL!	#NULL!	#NULL!	#NULL!	#NULL!
2,00	2,00	#NULL!	#NULL!	#NULL!	#NULL!	#NULL!
2,00	2,00	#NULL!	#NULL!	#NULL!	#NULL!	#NULL!
2,00	2,00	#NULL!	#NULL!	#NULL!	#NULL!	#NULL!
2,00	2,00	#NULL!	#NULL!	#NULL!	#NULL!	#NULL!
2,00	2,00	#NULL!	#NULL!	#NULL!	#NULL!	#NULL!
2,00	2,00	#NULL!	#NULL!	#NULL!	#NULL!	#NULL!
2,00	2,00	#NULL!	#NULL!	#NULL!	#NULL!	#NULL!
2,00	2,00	#NULL!	#NULL!	#NULL!	#NULL!	#NULL!
2,00	2,00	#NULL!	#NULL!	#NULL!	#NULL!	#NULL!
2,00	2,00	#NULL!	#NULL!	#NULL!	#NULL!	#NULL!
2,00	2,00	#NULL!	#NULL!	#NULL!	#NULL!	#NULL!
2,00	2,00	#NULL!	#NULL!	#NULL!	#NULL!	#NULL!
2,00	2,00	#NULL!	#NULL!	#NULL!	#NULL!	#NULL!
2,00	2,00	#NULL!	#NULL!	#NULL!	#NULL!	#NULL!
2,00	2,00	#NULL!	#NULL!	#NULL!	#NULL!	#NULL!
2,00	2,00	#NULL!	#NULL!	#NULL!	#NULL!	#NULL!
2,00	2,00	#NULL!	#NULL!	#NULL!	#NULL!	#NULL!
2,00	2,00	#NULL!	#NULL!	#NULL!	#NULL!	#NULL!
2,00	2,00	#NULL!	#NULL!	#NULL!	#NULL!	#NULL!
2,00	2,00	#NULL!	#NULL!	#NULL!	#NULL!	#NULL!
2,00	2,00	#NULL!	#NULL!	#NULL!	#NULL!	#NULL!
2,00	2,00	#NULL!	#NULL!	#NULL!	#NULL!	#NULL!
2,00	2,00	#NULL!	#NULL!	#NULL!	#NULL!	#NULL!
2,00	2,00	#NULL!	#NULL!	#NULL!	#NULL!	#NULL!
2,00	2,00	#NULL!	#NULL!	#NULL!	#NULL!	#NULL!
2,00	2,00	#NULL!	#NULL!	#NULL!	#NULL!	#NULL!
2,00	2,00	#NULL!	#NULL!	#NULL!	#NULL!	#NULL!
2,00	2,00	#NULL!	#NULL!	#NULL!	#NULL!	#NULL!
2,00	2,00	#NULL!	#NULL!	#NULL!	#NULL!	#NULL!
2,00	2,00	#NULL!	#NULL!	#NULL!	#NULL!	#NULL!

2,00	2,00	#NULL!	#NULL!	#NULL!	#NULL!	#NULL!
2,00	2,00	#NULL!	#NULL!	#NULL!	#NULL!	#NULL!
2,00	2,00	#NULL!	#NULL!	#NULL!	#NULL!	#NULL!
2,00	2,00	#NULL!	#NULL!	#NULL!	#NULL!	#NULL!
2,00	2,00	#NULL!	#NULL!	#NULL!	#NULL!	#NULL!
2,00	2,00	#NULL!	#NULL!	#NULL!	#NULL!	#NULL!
2,00	2,00	#NULL!	#NULL!	#NULL!	#NULL!	#NULL!
2,00	2,00	#NULL!	#NULL!	#NULL!	#NULL!	#NULL!
2,00	2,00	#NULL!	#NULL!	#NULL!	#NULL!	#NULL!
2,00	2,00	#NULL!	#NULL!	#NULL!	#NULL!	#NULL!
2,00	2,00	#NULL!	#NULL!	#NULL!	#NULL!	#NULL!
2,00	2,00	#NULL!	#NULL!	#NULL!	#NULL!	#NULL!
2,00	2,00	#NULL!	#NULL!	#NULL!	#NULL!	#NULL!
2,00	2,00	#NULL!	#NULL!	#NULL!	#NULL!	#NULL!
2,00	2,00	#NULL!	#NULL!	#NULL!	#NULL!	#NULL!
2,00	2,00	#NULL!	#NULL!	#NULL!	#NULL!	#NULL!
2,00	2,00	#NULL!	#NULL!	#NULL!	#NULL!	#NULL!
2,00	2,00	#NULL!	#NULL!	#NULL!	#NULL!	#NULL!
2,00	2,00	#NULL!	#NULL!	#NULL!	#NULL!	#NULL!
2,00	2,00	#NULL!	#NULL!	#NULL!	#NULL!	#NULL!
2,00	2,00	#NULL!	#NULL!	#NULL!	#NULL!	#NULL!
2,00	2,00	#NULL!	#NULL!	#NULL!	#NULL!	#NULL!
2,00	2,00	#NULL!	#NULL!	#NULL!	#NULL!	#NULL!
2,00	2,00	#NULL!	#NULL!	#NULL!	#NULL!	#NULL!
2,00	2,00	#NULL!	#NULL!	#NULL!	#NULL!	#NULL!
2,00	2,00	#NULL!	#NULL!	#NULL!	#NULL!	#NULL!
2,00	2,00	#NULL!	#NULL!	#NULL!	#NULL!	#NULL!
2,00	2,00	#NULL!	#NULL!	#NULL!	#NULL!	#NULL!
2,00	2,00	#NULL!	#NULL!	#NULL!	#NULL!	#NULL!
2,00	2,00	#NULL!	#NULL!	#NULL!	#NULL!	#NULL!
2,00	2,00	#NULL!	#NULL!	#NULL!	#NULL!	#NULL!
2,00	2,00	#NULL!	#NULL!	#NULL!	#NULL!	#NULL!
2,00	2,00	#NULL!	#NULL!	#NULL!	#NULL!	#NULL!
2,00	2,00	#NULL!	#NULL!	#NULL!	#NULL!	#NULL!
2,00	2,00	#NULL!	#NULL!	#NULL!	#NULL!	#NULL!
2,00	2,00	#NULL!	#NULL!	#NULL!	#NULL!	#NULL!
2,00	2,00	#NULL!	#NULL!	#NULL!	#NULL!	#NULL!
2,00	2,00	#NULL!	#NULL!	#NULL!	#NULL!	#NULL!
2,00	2,00	#NULL!	#NULL!	#NULL!	#NULL!	#NULL!
2,00	2,00	#NULL!	#NULL!	#NULL!	#NULL!	#NULL!
2,00	2,00	#NULL!	#NULL!	#NULL!	#NULL!	#NULL!
2,00	2,00	#NULL!	#NULL!	#NULL!	#NULL!	#NULL!
2,00	2,00	#NULL!	#NULL!	#NULL!	#NULL!	#NULL!
2,00	2,00	#NULL!	#NULL!	#NULL!	#NULL!	#NULL!
2,00	2,00	#NULL!	#NULL!	#NULL!	#NULL!	#NULL!
2,00	2,00	#NULL!	#NULL!	#NULL!	#NULL!	#NULL!
2,00	2,00	#NULL!	#NULL!	#NULL!	#NULL!	#NULL!
2,00	2,00	#NULL!	#NULL!	#NULL!	#NULL!	#NULL!
2,00	2,00	#NULL!	#NULL!	#NULL!	#NULL!	#NULL!
2,00	2,00	#NULL!	#NULL!	#NULL!	#NULL!	#NULL!

2,00	2,00	#NULL!	#NULL!	#NULL!	#NULL!	#NULL!
2,00	2,00	#NULL!	#NULL!	#NULL!	#NULL!	#NULL!
2,00	2,00	#NULL!	#NULL!	#NULL!	#NULL!	#NULL!
2,00	2,00	#NULL!	#NULL!	#NULL!	#NULL!	#NULL!
2,00	2,00	#NULL!	#NULL!	#NULL!	#NULL!	#NULL!
2,00	2,00	#NULL!	#NULL!	#NULL!	#NULL!	#NULL!
2,00	2,00	#NULL!	#NULL!	#NULL!	#NULL!	#NULL!
2,00	2,00	#NULL!	#NULL!	#NULL!	#NULL!	#NULL!
2,00	2,00	#NULL!	#NULL!	#NULL!	#NULL!	#NULL!
2,00	2,00	#NULL!	#NULL!	#NULL!	#NULL!	#NULL!
2,00	2,00	#NULL!	#NULL!	#NULL!	#NULL!	#NULL!
2,00	2,00	#NULL!	#NULL!	#NULL!	#NULL!	#NULL!
2,00	2,00	#NULL!	#NULL!	#NULL!	#NULL!	#NULL!
2,00	2,00	#NULL!	#NULL!	#NULL!	#NULL!	#NULL!
2,00	2,00	#NULL!	#NULL!	#NULL!	#NULL!	#NULL!
2,00	2,00	#NULL!	#NULL!	#NULL!	#NULL!	#NULL!
2,00	2,00	#NULL!	#NULL!	#NULL!	#NULL!	#NULL!
2,00	2,00	#NULL!	#NULL!	#NULL!	#NULL!	#NULL!
2,00	2,00	#NULL!	#NULL!	#NULL!	#NULL!	#NULL!
2,00	2,00	#NULL!	#NULL!	#NULL!	#NULL!	#NULL!
2,00	2,00	#NULL!	#NULL!	#NULL!	#NULL!	#NULL!
2,00	2,00	#NULL!	#NULL!	#NULL!	#NULL!	#NULL!
2,00	2,00	#NULL!	#NULL!	#NULL!	#NULL!	#NULL!
2,00	2,00	#NULL!	#NULL!	#NULL!	#NULL!	#NULL!
2,00	2,00	#NULL!	#NULL!	#NULL!	#NULL!	#NULL!
2,00	2,00	#NULL!	#NULL!	#NULL!	#NULL!	#NULL!
2,00	2,00	#NULL!	#NULL!	#NULL!	#NULL!	#NULL!
2,00	2,00	#NULL!	#NULL!	#NULL!	#NULL!	#NULL!
2,00	2,00	#NULL!	#NULL!	#NULL!	#NULL!	#NULL!
2,00	2,00	#NULL!	#NULL!	#NULL!	#NULL!	#NULL!
2,00	2,00	#NULL!	#NULL!	#NULL!	#NULL!	#NULL!
2,00	2,00	#NULL!	#NULL!	#NULL!	#NULL!	#NULL!
2,00	2,00	#NULL!	#NULL!	#NULL!	#NULL!	#NULL!
2,00	2,00	#NULL!	#NULL!	#NULL!	#NULL!	#NULL!
2,00	2,00	#NULL!	#NULL!	#NULL!	#NULL!	#NULL!
2,00	2,00	#NULL!	#NULL!	#NULL!	#NULL!	#NULL!
2,00	2,00	#NULL!	#NULL!	#NULL!	#NULL!	#NULL!
2,00	2,00	#NULL!	#NULL!	#NULL!	#NULL!	#NULL!
2,00	2,00	#NULL!	#NULL!	#NULL!	#NULL!	#NULL!
2,00	2,00	#NULL!	#NULL!	#NULL!	#NULL!	#NULL!
2,00	2,00	#NULL!	#NULL!	#NULL!	#NULL!	#NULL!
2,00	2,00	#NULL!	#NULL!	#NULL!	#NULL!	#NULL!
2,00	2,00	#NULL!	#NULL!	#NULL!	#NULL!	#NULL!
2,00	2,00	#NULL!	#NULL!	#NULL!	#NULL!	#NULL!
2,00	2,00	#NULL!	#NULL!	#NULL!	#NULL!	#NULL!
2,00	2,00	#NULL!	#NULL!	#NULL!	#NULL!	#NULL!
2,00	2,00	#NULL!	#NULL!	#NULL!	#NULL!	#NULL!
2,00	2,00	#NULL!	#NULL!	#NULL!	#NULL!	#NULL!
2,00	2,00	#NULL!	#NULL!	#NULL!	#NULL!	#NULL!
2,00	2,00	#NULL!	#NULL!	#NULL!	#NULL!	#NULL!

2,00	2,00	#NULL!	#NULL!	#NULL!	#NULL!	#NULL!
2,00	2,00	#NULL!	#NULL!	#NULL!	#NULL!	#NULL!
2,00	2,00	#NULL!	#NULL!	#NULL!	#NULL!	#NULL!
2,00	2,00	#NULL!	#NULL!	#NULL!	#NULL!	#NULL!
2,00	2,00	#NULL!	#NULL!	#NULL!	#NULL!	#NULL!
2,00	2,00	#NULL!	#NULL!	#NULL!	#NULL!	#NULL!
2,00	2,00	#NULL!	#NULL!	#NULL!	#NULL!	#NULL!
2,00	2,00	#NULL!	#NULL!	#NULL!	#NULL!	#NULL!
2,00	2,00	#NULL!	#NULL!	#NULL!	#NULL!	#NULL!
2,00	2,00	#NULL!	#NULL!	#NULL!	#NULL!	#NULL!
2,00	2,00	#NULL!	#NULL!	#NULL!	#NULL!	#NULL!
2,00	2,00	#NULL!	#NULL!	#NULL!	#NULL!	#NULL!
2,00	2,00	#NULL!	#NULL!	#NULL!	#NULL!	#NULL!
2,00	2,00	#NULL!	#NULL!	#NULL!	#NULL!	#NULL!
2,00	2,00	#NULL!	#NULL!	#NULL!	#NULL!	#NULL!
2,00	2,00	#NULL!	#NULL!	#NULL!	#NULL!	#NULL!
2,00	2,00	#NULL!	#NULL!	#NULL!	#NULL!	#NULL!
2,00	2,00	#NULL!	#NULL!	#NULL!	#NULL!	#NULL!
2,00	2,00	#NULL!	#NULL!	#NULL!	#NULL!	#NULL!
2,00	2,00	#NULL!	#NULL!	#NULL!	#NULL!	#NULL!
2,00	2,00	#NULL!	#NULL!	#NULL!	#NULL!	#NULL!
2,00	2,00	#NULL!	#NULL!	#NULL!	#NULL!	#NULL!
2,00	2,00	#NULL!	#NULL!	#NULL!	#NULL!	#NULL!
2,00	2,00	#NULL!	#NULL!	#NULL!	#NULL!	#NULL!
2,00	2,00	#NULL!	#NULL!	#NULL!	#NULL!	#NULL!
2,00	2,00	#NULL!	#NULL!	#NULL!	#NULL!	#NULL!
2,00	2,00	#NULL!	#NULL!	#NULL!	#NULL!	#NULL!
2,00	2,00	#NULL!	#NULL!	#NULL!	#NULL!	#NULL!
2,00	2,00	#NULL!	#NULL!	#NULL!	#NULL!	#NULL!
2,00	2,00	#NULL!	#NULL!	#NULL!	#NULL!	#NULL!
2,00	2,00	#NULL!	#NULL!	#NULL!	#NULL!	#NULL!
2,00	2,00	#NULL!	#NULL!	#NULL!	#NULL!	#NULL!
2,00	2,00	#NULL!	#NULL!	#NULL!	#NULL!	#NULL!
2,00	2,00	#NULL!	#NULL!	#NULL!	#NULL!	#NULL!
2,00	2,00	#NULL!	#NULL!	#NULL!	#NULL!	#NULL!
2,00	2,00	#NULL!	#NULL!	#NULL!	#NULL!	#NULL!
2,00	2,00	#NULL!	#NULL!	#NULL!	#NULL!	#NULL!
2,00	2,00	#NULL!	#NULL!	#NULL!	#NULL!	#NULL!
2,00	2,00	#NULL!	#NULL!	#NULL!	#NULL!	#NULL!
2,00	2,00	#NULL!	#NULL!	#NULL!	#NULL!	#NULL!
2,00	2,00	#NULL!	#NULL!	#NULL!	#NULL!	#NULL!
2,00	2,00	#NULL!	#NULL!	#NULL!	#NULL!	#NULL!
2,00	2,00	#NULL!	#NULL!	#NULL!	#NULL!	#NULL!
2,00	2,00	#NULL!	#NULL!	#NULL!	#NULL!	#NULL!
2,00	2,00	#NULL!	#NULL!	#NULL!	#NULL!	#NULL!
2,00	2,00	#NULL!	#NULL!	#NULL!	#NULL!	#NULL!
2,00	2,00	#NULL!	#NULL!	#NULL!	#NULL!	#NULL!
2,00	2,00	#NULL!	#NULL!	#NULL!	#NULL!	#NULL!
2,00	2,00	#NULL!	#NULL!	#NULL!	#NULL!	#NULL!
2,00	2,00	#NULL!	#NULL!	#NULL!	#NULL!	#NULL!

2,00	2,00	#NULL!	#NULL!	#NULL!	#NULL!	#NULL!
2,00	2,00	#NULL!	#NULL!	#NULL!	#NULL!	#NULL!
2,00	2,00	#NULL!	#NULL!	#NULL!	#NULL!	#NULL!
2,00	2,00	#NULL!	#NULL!	#NULL!	#NULL!	#NULL!
2,00	2,00	#NULL!	#NULL!	#NULL!	#NULL!	#NULL!
2,00	2,00	#NULL!	#NULL!	#NULL!	#NULL!	#NULL!
2,00	2,00	#NULL!	#NULL!	#NULL!	#NULL!	#NULL!
2,00	2,00	#NULL!	#NULL!	#NULL!	#NULL!	#NULL!
2,00	2,00	#NULL!	#NULL!	#NULL!	#NULL!	#NULL!
2,00	2,00	#NULL!	#NULL!	#NULL!	#NULL!	#NULL!
2,00	2,00	#NULL!	#NULL!	#NULL!	#NULL!	#NULL!
2,00	2,00	#NULL!	#NULL!	#NULL!	#NULL!	#NULL!
2,00	2,00	#NULL!	#NULL!	#NULL!	#NULL!	#NULL!
2,00	2,00	#NULL!	#NULL!	#NULL!	#NULL!	#NULL!
2,00	2,00	#NULL!	#NULL!	#NULL!	#NULL!	#NULL!
2,00	2,00	#NULL!	#NULL!	#NULL!	#NULL!	#NULL!
2,00	2,00	#NULL!	#NULL!	#NULL!	#NULL!	#NULL!
2,00	2,00	#NULL!	#NULL!	#NULL!	#NULL!	#NULL!
2,00	2,00	#NULL!	#NULL!	#NULL!	#NULL!	#NULL!
2,00	2,00	#NULL!	#NULL!	#NULL!	#NULL!	#NULL!
2,00	2,00	#NULL!	#NULL!	#NULL!	#NULL!	#NULL!
2,00	2,00	#NULL!	#NULL!	#NULL!	#NULL!	#NULL!
2,00	2,00	#NULL!	#NULL!	#NULL!	#NULL!	#NULL!
2,00	2,00	#NULL!	#NULL!	#NULL!	#NULL!	#NULL!
2,00	2,00	#NULL!	#NULL!	#NULL!	#NULL!	#NULL!
2,00	2,00	#NULL!	#NULL!	#NULL!	#NULL!	#NULL!
2,00	2,00	#NULL!	#NULL!	#NULL!	#NULL!	#NULL!
2,00	2,00	#NULL!	#NULL!	#NULL!	#NULL!	#NULL!
2,00	2,00	#NULL!	#NULL!	#NULL!	#NULL!	#NULL!
2,00	2,00	#NULL!	#NULL!	#NULL!	#NULL!	#NULL!
2,00	2,00	#NULL!	#NULL!	#NULL!	#NULL!	#NULL!
2,00	2,00	#NULL!	#NULL!	#NULL!	#NULL!	#NULL!
2,00	2,00	#NULL!	#NULL!	#NULL!	#NULL!	#NULL!
2,00	2,00	#NULL!	#NULL!	#NULL!	#NULL!	#NULL!
2,00	2,00	#NULL!	#NULL!	#NULL!	#NULL!	#NULL!
2,00	2,00	#NULL!	#NULL!	#NULL!	#NULL!	#NULL!
2,00	2,00	#NULL!	#NULL!	#NULL!	#NULL!	#NULL!
2,00	2,00	#NULL!	#NULL!	#NULL!	#NULL!	#NULL!
2,00	2,00	#NULL!	#NULL!	#NULL!	#NULL!	#NULL!
2,00	2,00	#NULL!	#NULL!	#NULL!	#NULL!	#NULL!
2,00	2,00	#NULL!	#NULL!	#NULL!	#NULL!	#NULL!
2,00	2,00	#NULL!	#NULL!	#NULL!	#NULL!	#NULL!
2,00	2,00	#NULL!	#NULL!	#NULL!	#NULL!	#NULL!
2,00	2,00	#NULL!	#NULL!	#NULL!	#NULL!	#NULL!
2,00	2,00	#NULL!	#NULL!	#NULL!	#NULL!	#NULL!
2,00	2,00	#NULL!	#NULL!	#NULL!	#NULL!	#NULL!
2,00	2,00	#NULL!	#NULL!	#NULL!	#NULL!	#NULL!
2,00	2,00	#NULL!	#NULL!	#NULL!	#NULL!	#NULL!
2,00	2,00	#NULL!	#NULL!	#NULL!	#NULL!	#NULL!
2,00	2,00	#NULL!	#NULL!	#NULL!	#NULL!	#NULL!

2,00	2,00	#NULL!	#NULL!	#NULL!	#NULL!	#NULL!
2,00	2,00	#NULL!	#NULL!	#NULL!	#NULL!	#NULL!
2,00	2,00	#NULL!	#NULL!	#NULL!	#NULL!	#NULL!
2,00	2,00	#NULL!	#NULL!	#NULL!	#NULL!	#NULL!
2,00	2,00	#NULL!	#NULL!	#NULL!	#NULL!	#NULL!
2,00	2,00	#NULL!	#NULL!	#NULL!	#NULL!	#NULL!
2,00	2,00	#NULL!	#NULL!	#NULL!	#NULL!	#NULL!
2,00	2,00	#NULL!	#NULL!	#NULL!	#NULL!	#NULL!
2,00	2,00	#NULL!	#NULL!	#NULL!	#NULL!	#NULL!
2,00	2,00	#NULL!	#NULL!	#NULL!	#NULL!	#NULL!
2,00	2,00	#NULL!	#NULL!	#NULL!	#NULL!	#NULL!
2,00	2,00	#NULL!	#NULL!	#NULL!	#NULL!	#NULL!
2,00	2,00	#NULL!	#NULL!	#NULL!	#NULL!	#NULL!
2,00	2,00	#NULL!	#NULL!	#NULL!	#NULL!	#NULL!
2,00	2,00	#NULL!	#NULL!	#NULL!	#NULL!	#NULL!
2,00	2,00	#NULL!	#NULL!	#NULL!	#NULL!	#NULL!
2,00	2,00	#NULL!	#NULL!	#NULL!	#NULL!	#NULL!
2,00	2,00	#NULL!	#NULL!	#NULL!	#NULL!	#NULL!
2,00	2,00	#NULL!	#NULL!	#NULL!	#NULL!	#NULL!
2,00	2,00	#NULL!	#NULL!	#NULL!	#NULL!	#NULL!
2,00	2,00	#NULL!	#NULL!	#NULL!	#NULL!	#NULL!
2,00	2,00	#NULL!	#NULL!	#NULL!	#NULL!	#NULL!
2,00	2,00	#NULL!	#NULL!	#NULL!	#NULL!	#NULL!
2,00	2,00	#NULL!	#NULL!	#NULL!	#NULL!	#NULL!
2,00	2,00	#NULL!	#NULL!	#NULL!	#NULL!	#NULL!
2,00	2,00	#NULL!	#NULL!	#NULL!	#NULL!	#NULL!
2,00	2,00	#NULL!	#NULL!	#NULL!	#NULL!	#NULL!
2,00	2,00	#NULL!	#NULL!	#NULL!	#NULL!	#NULL!
2,00	2,00	#NULL!	#NULL!	#NULL!	#NULL!	#NULL!
2,00	2,00	#NULL!	#NULL!	#NULL!	#NULL!	#NULL!
2,00	2,00	#NULL!	#NULL!	#NULL!	#NULL!	#NULL!
2,00	2,00	#NULL!	#NULL!	#NULL!	#NULL!	#NULL!
2,00	2,00	#NULL!	#NULL!	#NULL!	#NULL!	#NULL!
2,00	2,00	#NULL!	#NULL!	#NULL!	#NULL!	#NULL!
2,00	2,00	#NULL!	#NULL!	#NULL!	#NULL!	#NULL!
2,00	2,00	#NULL!	#NULL!	#NULL!	#NULL!	#NULL!
2,00	2,00	#NULL!	#NULL!	#NULL!	#NULL!	#NULL!
2,00	2,00	#NULL!	#NULL!	#NULL!	#NULL!	#NULL!
2,00	2,00	#NULL!	#NULL!	#NULL!	#NULL!	#NULL!
2,00	2,00	#NULL!	#NULL!	#NULL!	#NULL!	#NULL!
2,00	2,00	#NULL!	#NULL!	#NULL!	#NULL!	#NULL!
2,00	2,00	#NULL!	#NULL!	#NULL!	#NULL!	#NULL!
2,00	2,00	#NULL!	#NULL!	#NULL!	#NULL!	#NULL!
2,00	2,00	#NULL!	#NULL!	#NULL!	#NULL!	#NULL!
2,00	2,00	#NULL!	#NULL!	#NULL!	#NULL!	#NULL!
2,00	2,00	#NULL!	#NULL!	#NULL!	#NULL!	#NULL!
2,00	2,00	#NULL!	#NULL!	#NULL!	#NULL!	#NULL!
2,00	2,00	#NULL!	#NULL!	#NULL!	#NULL!	#NULL!
2,00	2,00	#NULL!	#NULL!	#NULL!	#NULL!	#NULL!
2,00	2,00	#NULL!	#NULL!	#NULL!	#NULL!	#NULL!

2,00	2,00	#NULL!	#NULL!	#NULL!	#NULL!	#NULL!
2,00	2,00	#NULL!	#NULL!	#NULL!	#NULL!	#NULL!
2,00	2,00	#NULL!	#NULL!	#NULL!	#NULL!	#NULL!
2,00	2,00	#NULL!	#NULL!	#NULL!	#NULL!	#NULL!
2,00	2,00	#NULL!	#NULL!	#NULL!	#NULL!	#NULL!
2,00	2,00	#NULL!	#NULL!	#NULL!	#NULL!	#NULL!
2,00	2,00	#NULL!	#NULL!	#NULL!	#NULL!	#NULL!
2,00	2,00	#NULL!	#NULL!	#NULL!	#NULL!	#NULL!
2,00	2,00	#NULL!	#NULL!	#NULL!	#NULL!	#NULL!
2,00	2,00	#NULL!	#NULL!	#NULL!	#NULL!	#NULL!
2,00	2,00	#NULL!	#NULL!	#NULL!	#NULL!	#NULL!
2,00	2,00	#NULL!	#NULL!	#NULL!	#NULL!	#NULL!
2,00	2,00	#NULL!	#NULL!	#NULL!	#NULL!	#NULL!
2,00	2,00	#NULL!	#NULL!	#NULL!	#NULL!	#NULL!
2,00	2,00	#NULL!	#NULL!	#NULL!	#NULL!	#NULL!
2,00	2,00	#NULL!	#NULL!	#NULL!	#NULL!	#NULL!
2,00	2,00	#NULL!	#NULL!	#NULL!	#NULL!	#NULL!
2,00	2,00	#NULL!	#NULL!	#NULL!	#NULL!	#NULL!
2,00	2,00	#NULL!	#NULL!	#NULL!	#NULL!	#NULL!
2,00	2,00	#NULL!	#NULL!	#NULL!	#NULL!	#NULL!
2,00	2,00	#NULL!	#NULL!	#NULL!	#NULL!	#NULL!
2,00	2,00	#NULL!	#NULL!	#NULL!	#NULL!	#NULL!
2,00	2,00	#NULL!	#NULL!	#NULL!	#NULL!	#NULL!
1,00	2,00	#NULL!	#NULL!	#NULL!	#NULL!	#NULL!
2,00	2,00	#NULL!	#NULL!	#NULL!	#NULL!	#NULL!
2,00	2,00	#NULL!	#NULL!	#NULL!	#NULL!	#NULL!
2,00	2,00	#NULL!	#NULL!	#NULL!	#NULL!	#NULL!
2,00	2,00	#NULL!	#NULL!	#NULL!	#NULL!	#NULL!
2,00	2,00	#NULL!	#NULL!	#NULL!	#NULL!	#NULL!
2,00	2,00	#NULL!	#NULL!	#NULL!	#NULL!	#NULL!
2,00	2,00	#NULL!	#NULL!	#NULL!	#NULL!	#NULL!
2,00	2,00	#NULL!	#NULL!	#NULL!	#NULL!	#NULL!
2,00	2,00	#NULL!	#NULL!	#NULL!	#NULL!	#NULL!
2,00	2,00	#NULL!	#NULL!	#NULL!	#NULL!	#NULL!
2,00	2,00	#NULL!	#NULL!	#NULL!	#NULL!	#NULL!
2,00	2,00	#NULL!	#NULL!	#NULL!	#NULL!	#NULL!
2,00	2,00	#NULL!	#NULL!	#NULL!	#NULL!	#NULL!
2,00	2,00	#NULL!	#NULL!	#NULL!	#NULL!	#NULL!
2,00	2,00	#NULL!	#NULL!	#NULL!	#NULL!	#NULL!
1,00	2,00	#NULL!	#NULL!	#NULL!	#NULL!	#NULL!
2,00	2,00	#NULL!	#NULL!	#NULL!	#NULL!	#NULL!
2,00	2,00	#NULL!	#NULL!	#NULL!	#NULL!	#NULL!
2,00	2,00	#NULL!	#NULL!	#NULL!	#NULL!	#NULL!
2,00	2,00	#NULL!	#NULL!	#NULL!	#NULL!	#NULL!
2,00	2,00	#NULL!	#NULL!	#NULL!	#NULL!	#NULL!
2,00	2,00	#NULL!	#NULL!	#NULL!	#NULL!	#NULL!
2,00	2,00	#NULL!	#NULL!	#NULL!	#NULL!	#NULL!
2,00	2,00	#NULL!	#NULL!	#NULL!	#NULL!	#NULL!
2,00	2,00	#NULL!	#NULL!	#NULL!	#NULL!	#NULL!
1,00	2,00	#NULL!	#NULL!	#NULL!	#NULL!	#NULL!

2,00	2,00	#NULL!	#NULL!	#NULL!	#NULL!	#NULL!
2,00	2,00	#NULL!	#NULL!	#NULL!	#NULL!	#NULL!
2,00	2,00	#NULL!	#NULL!	#NULL!	#NULL!	#NULL!
2,00	2,00	#NULL!	#NULL!	#NULL!	#NULL!	#NULL!
2,00	2,00	#NULL!	#NULL!	#NULL!	#NULL!	#NULL!
2,00	2,00	#NULL!	#NULL!	#NULL!	#NULL!	#NULL!
2,00	2,00	#NULL!	#NULL!	#NULL!	#NULL!	#NULL!
2,00	2,00	#NULL!	#NULL!	#NULL!	#NULL!	#NULL!
2,00	2,00	#NULL!	#NULL!	#NULL!	#NULL!	#NULL!
2,00	2,00	#NULL!	#NULL!	#NULL!	#NULL!	#NULL!
2,00	2,00	#NULL!	#NULL!	#NULL!	#NULL!	#NULL!
2,00	2,00	#NULL!	#NULL!	#NULL!	#NULL!	#NULL!
2,00	2,00	#NULL!	#NULL!	#NULL!	#NULL!	#NULL!
2,00	2,00	#NULL!	#NULL!	#NULL!	#NULL!	#NULL!
2,00	2,00	#NULL!	#NULL!	#NULL!	#NULL!	#NULL!
2,00	2,00	#NULL!	#NULL!	#NULL!	#NULL!	#NULL!
2,00	2,00	#NULL!	#NULL!	#NULL!	#NULL!	#NULL!
2,00	2,00	#NULL!	#NULL!	#NULL!	#NULL!	#NULL!
2,00	2,00	#NULL!	#NULL!	#NULL!	#NULL!	#NULL!
2,00	2,00	#NULL!	#NULL!	#NULL!	#NULL!	#NULL!
2,00	2,00	#NULL!	#NULL!	#NULL!	#NULL!	#NULL!
2,00	2,00	#NULL!	#NULL!	#NULL!	#NULL!	#NULL!
1,00	2,00	#NULL!	#NULL!	#NULL!	#NULL!	#NULL!
2,00	2,00	#NULL!	#NULL!	#NULL!	#NULL!	#NULL!
2,00	2,00	#NULL!	#NULL!	#NULL!	#NULL!	#NULL!
2,00	2,00	#NULL!	#NULL!	#NULL!	#NULL!	#NULL!
2,00	2,00	#NULL!	#NULL!	#NULL!	#NULL!	#NULL!
2,00	2,00	#NULL!	#NULL!	#NULL!	#NULL!	#NULL!
2,00	2,00	#NULL!	#NULL!	#NULL!	#NULL!	#NULL!
1,00	2,00	#NULL!	#NULL!	#NULL!	#NULL!	#NULL!
2,00	2,00	#NULL!	#NULL!	#NULL!	#NULL!	#NULL!
2,00	1,00	1,00	2,00	1,00	2,00	#NULL!
2,00	2,00	#NULL!	#NULL!	#NULL!	#NULL!	#NULL!
2,00	2,00	#NULL!	#NULL!	#NULL!	#NULL!	#NULL!
2,00	2,00	#NULL!	#NULL!	#NULL!	#NULL!	#NULL!
2,00	1,00	2,00	1,00	2,00	2,00	#NULL!
2,00	2,00	#NULL!	#NULL!	#NULL!	#NULL!	#NULL!
2,00	2,00	#NULL!	#NULL!	#NULL!	#NULL!	#NULL!
2,00	2,00	#NULL!	#NULL!	#NULL!	#NULL!	#NULL!
2,00	2,00	#NULL!	#NULL!	#NULL!	#NULL!	#NULL!
2,00	2,00	#NULL!	#NULL!	#NULL!	#NULL!	#NULL!
2,00	1,00	1,00	2,00	1,00	2,00	#NULL!
2,00	1,00	1,00	1,00	2,00	2,00	#NULL!
2,00	2,00	#NULL!	#NULL!	#NULL!	#NULL!	#NULL!
2,00	2,00	#NULL!	#NULL!	#NULL!	#NULL!	#NULL!
2,00	2,00	#NULL!	#NULL!	#NULL!	#NULL!	#NULL!
2,00	2,00	#NULL!	#NULL!	#NULL!	#NULL!	#NULL!
2,00	1,00	1,00	1,00	2,00	2,00	#NULL!
2,00	2,00	#NULL!	#NULL!	#NULL!	#NULL!	#NULL!
2,00	2,00	#NULL!	#NULL!	#NULL!	#NULL!	#NULL!

2,00	2,00	#NULL!	#NULL!	#NULL!	#NULL!	#NULL!
2,00	2,00	#NULL!	#NULL!	#NULL!	#NULL!	#NULL!
2,00	2,00	#NULL!	#NULL!	#NULL!	#NULL!	#NULL!
2,00	2,00	#NULL!	#NULL!	#NULL!	#NULL!	#NULL!
2,00	2,00	#NULL!	#NULL!	#NULL!	#NULL!	#NULL!
2,00	2,00	#NULL!	#NULL!	#NULL!	#NULL!	#NULL!
2,00	2,00	#NULL!	#NULL!	#NULL!	#NULL!	#NULL!
2,00	1,00	1,00	1,00	2,00	2,00	#NULL!
2,00	2,00	#NULL!	#NULL!	#NULL!	#NULL!	#NULL!
2,00	2,00	#NULL!	#NULL!	#NULL!	#NULL!	#NULL!
2,00	1,00	1,00	1,00	2,00	2,00	#NULL!
2,00	1,00	2,00	1,00	2,00	2,00	#NULL!
2,00	2,00	#NULL!	#NULL!	#NULL!	#NULL!	#NULL!
2,00	2,00	#NULL!	#NULL!	#NULL!	#NULL!	#NULL!
2,00	2,00	#NULL!	#NULL!	#NULL!	#NULL!	#NULL!
2,00	2,00	#NULL!	#NULL!	#NULL!	#NULL!	#NULL!
2,00	1,00	1,00	1,00	2,00	2,00	#NULL!
2,00	2,00	#NULL!	#NULL!	#NULL!	#NULL!	#NULL!
2,00	2,00	#NULL!	#NULL!	#NULL!	#NULL!	#NULL!
2,00	2,00	#NULL!	#NULL!	#NULL!	#NULL!	#NULL!
2,00	2,00	#NULL!	#NULL!	#NULL!	#NULL!	#NULL!
2,00	2,00	#NULL!	#NULL!	#NULL!	#NULL!	#NULL!
2,00	2,00	#NULL!	#NULL!	#NULL!	#NULL!	#NULL!
2,00	2,00	#NULL!	#NULL!	#NULL!	#NULL!	#NULL!
2,00	2,00	#NULL!	#NULL!	#NULL!	#NULL!	#NULL!
2,00	2,00	#NULL!	#NULL!	#NULL!	#NULL!	#NULL!
2,00	2,00	#NULL!	#NULL!	#NULL!	#NULL!	#NULL!
2,00	2,00	#NULL!	#NULL!	#NULL!	#NULL!	#NULL!
2,00	2,00	#NULL!	#NULL!	#NULL!	#NULL!	#NULL!
2,00	2,00	#NULL!	#NULL!	#NULL!	#NULL!	#NULL!
2,00	2,00	#NULL!	#NULL!	#NULL!	#NULL!	#NULL!
2,00	2,00	#NULL!	#NULL!	#NULL!	#NULL!	#NULL!
2,00	2,00	#NULL!	#NULL!	#NULL!	#NULL!	#NULL!
2,00	2,00	#NULL!	#NULL!	#NULL!	#NULL!	#NULL!
2,00	2,00	#NULL!	#NULL!	#NULL!	#NULL!	#NULL!
2,00	2,00	#NULL!	#NULL!	#NULL!	#NULL!	#NULL!
2,00	2,00	#NULL!	#NULL!	#NULL!	#NULL!	#NULL!
2,00	2,00	#NULL!	#NULL!	#NULL!	#NULL!	#NULL!
2,00	2,00	#NULL!	#NULL!	#NULL!	#NULL!	#NULL!
2,00	2,00	#NULL!	#NULL!	#NULL!	#NULL!	#NULL!
2,00	2,00	#NULL!	#NULL!	#NULL!	#NULL!	#NULL!
2,00	2,00	#NULL!	#NULL!	#NULL!	#NULL!	#NULL!
2,00	2,00	#NULL!	#NULL!	#NULL!	#NULL!	#NULL!
2,00	2,00	#NULL!	#NULL!	#NULL!	#NULL!	#NULL!
2,00	2,00	#NULL!	#NULL!	#NULL!	#NULL!	#NULL!
2,00	2,00	#NULL!	#NULL!	#NULL!	#NULL!	#NULL!
2,00	2,00	#NULL!	#NULL!	#NULL!	#NULL!	#NULL!
2,00	2,00	#NULL!	#NULL!	#NULL!	#NULL!	#NULL!
2,00	2,00	#NULL!	#NULL!	#NULL!	#NULL!	#NULL!
2,00	2,00	#NULL!	#NULL!	#NULL!	#NULL!	#NULL!

2,00	2,00	#NULL!	#NULL!	#NULL!	#NULL!	#NULL!
2,00	2,00	#NULL!	#NULL!	#NULL!	#NULL!	#NULL!
2,00	2,00	#NULL!	#NULL!	#NULL!	#NULL!	#NULL!
2,00	2,00	#NULL!	#NULL!	#NULL!	#NULL!	#NULL!
2,00	2,00	#NULL!	#NULL!	#NULL!	#NULL!	#NULL!
2,00	2,00	#NULL!	#NULL!	#NULL!	#NULL!	#NULL!
2,00	2,00	#NULL!	#NULL!	#NULL!	#NULL!	#NULL!
2,00	2,00	#NULL!	#NULL!	#NULL!	#NULL!	#NULL!
2,00	2,00	#NULL!	#NULL!	#NULL!	#NULL!	#NULL!
2,00	2,00	#NULL!	#NULL!	#NULL!	#NULL!	#NULL!
2,00	2,00	#NULL!	#NULL!	#NULL!	#NULL!	#NULL!
2,00	2,00	#NULL!	#NULL!	#NULL!	#NULL!	#NULL!
2,00	2,00	#NULL!	#NULL!	#NULL!	#NULL!	#NULL!
2,00	2,00	#NULL!	#NULL!	#NULL!	#NULL!	#NULL!
2,00	2,00	#NULL!	#NULL!	#NULL!	#NULL!	#NULL!
2,00	2,00	#NULL!	#NULL!	#NULL!	#NULL!	#NULL!
2,00	2,00	#NULL!	#NULL!	#NULL!	#NULL!	#NULL!
2,00	2,00	#NULL!	#NULL!	#NULL!	#NULL!	#NULL!
2,00	2,00	#NULL!	#NULL!	#NULL!	#NULL!	#NULL!
2,00	2,00	#NULL!	#NULL!	#NULL!	#NULL!	#NULL!
2,00	2,00	#NULL!	#NULL!	#NULL!	#NULL!	#NULL!
2,00	2,00	#NULL!	#NULL!	#NULL!	#NULL!	#NULL!
2,00	2,00	#NULL!	#NULL!	#NULL!	#NULL!	#NULL!
2,00	2,00	#NULL!	#NULL!	#NULL!	#NULL!	#NULL!
2,00	2,00	#NULL!	#NULL!	#NULL!	#NULL!	#NULL!
2,00	2,00	#NULL!	#NULL!	#NULL!	#NULL!	#NULL!
2,00	2,00	#NULL!	#NULL!	#NULL!	#NULL!	#NULL!
2,00	2,00	#NULL!	#NULL!	#NULL!	#NULL!	#NULL!
2,00	2,00	#NULL!	#NULL!	#NULL!	#NULL!	#NULL!
2,00	2,00	#NULL!	#NULL!	#NULL!	#NULL!	#NULL!
2,00	2,00	#NULL!	#NULL!	#NULL!	#NULL!	#NULL!
2,00	2,00	#NULL!	#NULL!	#NULL!	#NULL!	#NULL!
2,00	2,00	#NULL!	#NULL!	#NULL!	#NULL!	#NULL!
2,00	2,00	#NULL!	#NULL!	#NULL!	#NULL!	#NULL!
2,00	2,00	#NULL!	#NULL!	#NULL!	#NULL!	#NULL!
2,00	2,00	#NULL!	#NULL!	#NULL!	#NULL!	#NULL!
2,00	2,00	#NULL!	#NULL!	#NULL!	#NULL!	#NULL!
2,00	2,00	#NULL!	#NULL!	#NULL!	#NULL!	#NULL!
2,00	2,00	#NULL!	#NULL!	#NULL!	#NULL!	#NULL!
2,00	2,00	#NULL!	#NULL!	#NULL!	#NULL!	#NULL!
2,00	2,00	#NULL!	#NULL!	#NULL!	#NULL!	#NULL!
2,00	2,00	#NULL!	#NULL!	#NULL!	#NULL!	#NULL!
2,00	2,00	#NULL!	#NULL!	#NULL!	#NULL!	#NULL!
2,00	2,00	#NULL!	#NULL!	#NULL!	#NULL!	#NULL!
2,00	2,00	#NULL!	#NULL!	#NULL!	#NULL!	#NULL!
2,00	2,00	#NULL!	#NULL!	#NULL!	#NULL!	#NULL!
2,00	2,00	#NULL!	#NULL!	#NULL!	#NULL!	#NULL!
2,00	2,00	#NULL!	#NULL!	#NULL!	#NULL!	#NULL!
2,00	2,00	#NULL!	#NULL!	#NULL!	#NULL!	#NULL!
2,00	2,00	#NULL!	#NULL!	#NULL!	#NULL!	#NULL!

2,00	2,00	#NULL!	#NULL!	#NULL!	#NULL!	#NULL!
2,00	2,00	#NULL!	#NULL!	#NULL!	#NULL!	#NULL!
2,00	2,00	#NULL!	#NULL!	#NULL!	#NULL!	#NULL!
2,00	2,00	#NULL!	#NULL!	#NULL!	#NULL!	#NULL!
2,00	2,00	#NULL!	#NULL!	#NULL!	#NULL!	#NULL!
2,00	2,00	#NULL!	#NULL!	#NULL!	#NULL!	#NULL!
2,00	2,00	#NULL!	#NULL!	#NULL!	#NULL!	#NULL!
2,00	2,00	#NULL!	#NULL!	#NULL!	#NULL!	#NULL!
2,00	2,00	#NULL!	#NULL!	#NULL!	#NULL!	#NULL!
2,00	2,00	#NULL!	#NULL!	#NULL!	#NULL!	#NULL!
2,00	2,00	#NULL!	#NULL!	#NULL!	#NULL!	#NULL!
2,00	2,00	#NULL!	#NULL!	#NULL!	#NULL!	#NULL!
2,00	2,00	#NULL!	#NULL!	#NULL!	#NULL!	#NULL!
2,00	2,00	#NULL!	#NULL!	#NULL!	#NULL!	#NULL!
2,00	2,00	#NULL!	#NULL!	#NULL!	#NULL!	#NULL!
2,00	2,00	#NULL!	#NULL!	#NULL!	#NULL!	#NULL!
2,00	2,00	#NULL!	#NULL!	#NULL!	#NULL!	#NULL!
2,00	2,00	#NULL!	#NULL!	#NULL!	#NULL!	#NULL!
2,00	2,00	#NULL!	#NULL!	#NULL!	#NULL!	#NULL!
2,00	2,00	#NULL!	#NULL!	#NULL!	#NULL!	#NULL!
2,00	2,00	#NULL!	#NULL!	#NULL!	#NULL!	#NULL!
2,00	2,00	#NULL!	#NULL!	#NULL!	#NULL!	#NULL!
2,00	2,00	#NULL!	#NULL!	#NULL!	#NULL!	#NULL!
2,00	2,00	#NULL!	#NULL!	#NULL!	#NULL!	#NULL!
2,00	2,00	#NULL!	#NULL!	#NULL!	#NULL!	#NULL!
2,00	2,00	#NULL!	#NULL!	#NULL!	#NULL!	#NULL!
2,00	2,00	#NULL!	#NULL!	#NULL!	#NULL!	#NULL!
2,00	2,00	#NULL!	#NULL!	#NULL!	#NULL!	#NULL!
2,00	2,00	#NULL!	#NULL!	#NULL!	#NULL!	#NULL!
2,00	2,00	#NULL!	#NULL!	#NULL!	#NULL!	#NULL!
2,00	2,00	#NULL!	#NULL!	#NULL!	#NULL!	#NULL!
2,00	2,00	#NULL!	#NULL!	#NULL!	#NULL!	#NULL!
2,00	2,00	#NULL!	#NULL!	#NULL!	#NULL!	#NULL!
2,00	2,00	#NULL!	#NULL!	#NULL!	#NULL!	#NULL!
2,00	2,00	#NULL!	#NULL!	#NULL!	#NULL!	#NULL!
2,00	2,00	#NULL!	#NULL!	#NULL!	#NULL!	#NULL!
2,00	2,00	#NULL!	#NULL!	#NULL!	#NULL!	#NULL!
2,00	2,00	#NULL!	#NULL!	#NULL!	#NULL!	#NULL!
2,00	2,00	#NULL!	#NULL!	#NULL!	#NULL!	#NULL!
2,00	2,00	#NULL!	#NULL!	#NULL!	#NULL!	#NULL!
2,00	2,00	#NULL!	#NULL!	#NULL!	#NULL!	#NULL!
2,00	2,00	#NULL!	#NULL!	#NULL!	#NULL!	#NULL!
2,00	2,00	#NULL!	#NULL!	#NULL!	#NULL!	#NULL!
2,00	2,00	#NULL!	#NULL!	#NULL!	#NULL!	#NULL!
2,00	2,00	#NULL!	#NULL!	#NULL!	#NULL!	#NULL!
2,00	2,00	#NULL!	#NULL!	#NULL!	#NULL!	#NULL!
2,00	2,00	#NULL!	#NULL!	#NULL!	#NULL!	#NULL!
2,00	2,00	#NULL!	#NULL!	#NULL!	#NULL!	#NULL!
2,00	2,00	#NULL!	#NULL!	#NULL!	#NULL!	#NULL!
2,00	2,00	#NULL!	#NULL!	#NULL!	#NULL!	#NULL!

2,00	2,00	#NULL!	#NULL!	#NULL!	#NULL!	#NULL!
1,00	2,00	#NULL!	#NULL!	#NULL!	#NULL!	#NULL!
2,00	2,00	#NULL!	#NULL!	#NULL!	#NULL!	#NULL!
2,00	2,00	#NULL!	#NULL!	#NULL!	#NULL!	#NULL!
2,00	2,00	#NULL!	#NULL!	#NULL!	#NULL!	#NULL!
2,00	2,00	#NULL!	#NULL!	#NULL!	#NULL!	#NULL!
2,00	2,00	#NULL!	#NULL!	#NULL!	#NULL!	#NULL!
2,00	2,00	#NULL!	#NULL!	#NULL!	#NULL!	#NULL!
2,00	2,00	#NULL!	#NULL!	#NULL!	#NULL!	#NULL!
2,00	2,00	#NULL!	#NULL!	#NULL!	#NULL!	#NULL!
2,00	2,00	#NULL!	#NULL!	#NULL!	#NULL!	#NULL!
2,00	2,00	#NULL!	#NULL!	#NULL!	#NULL!	#NULL!
2,00	2,00	#NULL!	#NULL!	#NULL!	#NULL!	#NULL!
2,00	2,00	#NULL!	#NULL!	#NULL!	#NULL!	#NULL!
2,00	2,00	#NULL!	#NULL!	#NULL!	#NULL!	#NULL!
2,00	2,00	#NULL!	#NULL!	#NULL!	#NULL!	#NULL!
2,00	2,00	#NULL!	#NULL!	#NULL!	#NULL!	#NULL!
2,00	2,00	#NULL!	#NULL!	#NULL!	#NULL!	#NULL!
2,00	2,00	#NULL!	#NULL!	#NULL!	#NULL!	#NULL!
2,00	2,00	#NULL!	#NULL!	#NULL!	#NULL!	#NULL!
2,00	2,00	#NULL!	#NULL!	#NULL!	#NULL!	#NULL!
2,00	2,00	#NULL!	#NULL!	#NULL!	#NULL!	#NULL!
2,00	2,00	#NULL!	#NULL!	#NULL!	#NULL!	#NULL!
2,00	2,00	#NULL!	#NULL!	#NULL!	#NULL!	#NULL!
2,00	2,00	#NULL!	#NULL!	#NULL!	#NULL!	#NULL!
2,00	2,00	#NULL!	#NULL!	#NULL!	#NULL!	#NULL!
2,00	2,00	#NULL!	#NULL!	#NULL!	#NULL!	#NULL!
2,00	2,00	#NULL!	#NULL!	#NULL!	#NULL!	#NULL!
2,00	2,00	#NULL!	#NULL!	#NULL!	#NULL!	#NULL!
2,00	2,00	#NULL!	#NULL!	#NULL!	#NULL!	#NULL!
2,00	2,00	#NULL!	#NULL!	#NULL!	#NULL!	#NULL!
2,00	2,00	#NULL!	#NULL!	#NULL!	#NULL!	#NULL!
2,00	2,00	#NULL!	#NULL!	#NULL!	#NULL!	#NULL!
2,00	2,00	#NULL!	#NULL!	#NULL!	#NULL!	#NULL!
2,00	2,00	#NULL!	#NULL!	#NULL!	#NULL!	#NULL!
2,00	2,00	#NULL!	#NULL!	#NULL!	#NULL!	#NULL!
2,00	2,00	#NULL!	#NULL!	#NULL!	#NULL!	#NULL!
2,00	2,00	#NULL!	#NULL!	#NULL!	#NULL!	#NULL!
2,00	2,00	#NULL!	#NULL!	#NULL!	#NULL!	#NULL!
2,00	2,00	#NULL!	#NULL!	#NULL!	#NULL!	#NULL!
2,00	2,00	#NULL!	#NULL!	#NULL!	#NULL!	#NULL!
2,00	2,00	#NULL!	#NULL!	#NULL!	#NULL!	#NULL!
2,00	1,00	#NULL!	#NULL!	#NULL!	#NULL!	#NULL!
2,00	2,00	#NULL!	#NULL!	#NULL!	#NULL!	#NULL!
2,00	2,00	#NULL!	#NULL!	#NULL!	#NULL!	#NULL!
2,00	2,00	#NULL!	#NULL!	#NULL!	#NULL!	#NULL!
2,00	2,00	#NULL!	#NULL!	#NULL!	#NULL!	#NULL!
2,00	2,00	#NULL!	#NULL!	#NULL!	#NULL!	#NULL!
2,00	2,00	#NULL!	#NULL!	#NULL!	#NULL!	#NULL!
2,00	2,00	#NULL!	#NULL!	#NULL!	#NULL!	#NULL!

2,00	2,00	#NULL!	#NULL!	#NULL!	#NULL!	#NULL!
2,00	2,00	#NULL!	#NULL!	#NULL!	#NULL!	#NULL!
2,00	2,00	#NULL!	#NULL!	#NULL!	#NULL!	#NULL!
2,00	2,00	#NULL!	#NULL!	#NULL!	#NULL!	#NULL!
2,00	2,00	#NULL!	#NULL!	#NULL!	#NULL!	#NULL!
2,00	2,00	#NULL!	#NULL!	#NULL!	#NULL!	#NULL!
2,00	2,00	#NULL!	#NULL!	#NULL!	#NULL!	#NULL!
2,00	2,00	#NULL!	#NULL!	#NULL!	#NULL!	#NULL!
2,00	2,00	#NULL!	#NULL!	#NULL!	#NULL!	#NULL!
2,00	2,00	#NULL!	#NULL!	#NULL!	#NULL!	#NULL!
2,00	2,00	#NULL!	#NULL!	#NULL!	#NULL!	#NULL!
2,00	2,00	#NULL!	#NULL!	#NULL!	#NULL!	#NULL!
2,00	2,00	#NULL!	#NULL!	#NULL!	#NULL!	#NULL!
2,00	2,00	#NULL!	#NULL!	#NULL!	#NULL!	#NULL!
2,00	2,00	#NULL!	#NULL!	#NULL!	#NULL!	#NULL!
1,00	2,00	#NULL!	#NULL!	#NULL!	#NULL!	#NULL!
1,00	2,00	#NULL!	#NULL!	#NULL!	#NULL!	#NULL!
2,00	2,00	#NULL!	#NULL!	#NULL!	#NULL!	#NULL!
2,00	2,00	#NULL!	#NULL!	#NULL!	#NULL!	#NULL!
2,00	2,00	#NULL!	#NULL!	#NULL!	#NULL!	#NULL!
2,00	2,00	#NULL!	#NULL!	#NULL!	#NULL!	#NULL!
1,00	2,00	#NULL!	#NULL!	#NULL!	#NULL!	#NULL!
1,00	2,00	#NULL!	#NULL!	#NULL!	#NULL!	#NULL!
2,00	2,00	#NULL!	#NULL!	#NULL!	#NULL!	#NULL!
2,00	2,00	#NULL!	#NULL!	#NULL!	#NULL!	#NULL!
2,00	2,00	#NULL!	#NULL!	#NULL!	#NULL!	#NULL!
2,00	2,00	#NULL!	#NULL!	#NULL!	#NULL!	#NULL!
2,00	2,00	#NULL!	#NULL!	#NULL!	#NULL!	#NULL!
2,00	2,00	#NULL!	#NULL!	#NULL!	#NULL!	#NULL!
2,00	2,00	#NULL!	#NULL!	#NULL!	#NULL!	#NULL!
2,00	2,00	#NULL!	#NULL!	#NULL!	#NULL!	#NULL!
1,00	2,00	#NULL!	#NULL!	#NULL!	#NULL!	#NULL!
2,00	2,00	#NULL!	#NULL!	#NULL!	#NULL!	#NULL!
2,00	2,00	#NULL!	#NULL!	#NULL!	#NULL!	#NULL!
2,00	2,00	#NULL!	#NULL!	#NULL!	#NULL!	#NULL!
2,00	2,00	#NULL!	#NULL!	#NULL!	#NULL!	#NULL!
2,00	2,00	#NULL!	#NULL!	#NULL!	#NULL!	#NULL!
2,00	2,00	#NULL!	#NULL!	#NULL!	#NULL!	#NULL!
2,00	2,00	#NULL!	#NULL!	#NULL!	#NULL!	#NULL!
2,00	2,00	#NULL!	#NULL!	#NULL!	#NULL!	#NULL!
1,00	2,00	#NULL!	#NULL!	#NULL!	#NULL!	#NULL!
2,00	2,00	#NULL!	#NULL!	#NULL!	#NULL!	#NULL!
2,00	2,00	#NULL!	#NULL!	#NULL!	#NULL!	#NULL!
1,00	2,00	#NULL!	#NULL!	#NULL!	#NULL!	#NULL!
2,00	2,00	#NULL!	#NULL!	#NULL!	#NULL!	#NULL!
2,00	2,00	#NULL!	#NULL!	#NULL!	#NULL!	#NULL!
1,00	2,00	#NULL!	#NULL!	#NULL!	#NULL!	#NULL!
2,00	2,00	#NULL!	#NULL!	#NULL!	#NULL!	#NULL!
2,00	2,00	#NULL!	#NULL!	#NULL!	#NULL!	#NULL!
2,00	2,00	#NULL!	#NULL!	#NULL!	#NULL!	#NULL!

2,00	2,00	#NULL!	#NULL!	#NULL!	#NULL!	#NULL!
2,00	2,00	#NULL!	#NULL!	#NULL!	#NULL!	#NULL!
2,00	2,00	#NULL!	#NULL!	#NULL!	#NULL!	#NULL!
2,00	2,00	#NULL!	#NULL!	#NULL!	#NULL!	#NULL!
2,00	2,00	#NULL!	#NULL!	#NULL!	#NULL!	#NULL!
2,00	2,00	#NULL!	#NULL!	#NULL!	#NULL!	#NULL!
2,00	2,00	#NULL!	#NULL!	#NULL!	#NULL!	#NULL!
2,00	2,00	#NULL!	#NULL!	#NULL!	#NULL!	#NULL!
2,00	2,00	#NULL!	#NULL!	#NULL!	#NULL!	#NULL!
2,00	2,00	#NULL!	#NULL!	#NULL!	#NULL!	#NULL!
1,00	2,00	#NULL!	#NULL!	#NULL!	#NULL!	#NULL!
2,00	2,00	#NULL!	#NULL!	#NULL!	#NULL!	#NULL!
2,00	2,00	#NULL!	#NULL!	#NULL!	#NULL!	#NULL!
2,00	2,00	#NULL!	#NULL!	#NULL!	#NULL!	#NULL!
2,00	2,00	#NULL!	#NULL!	#NULL!	#NULL!	#NULL!
2,00	2,00	#NULL!	#NULL!	#NULL!	#NULL!	#NULL!
2,00	2,00	#NULL!	#NULL!	#NULL!	#NULL!	#NULL!
2,00	2,00	#NULL!	#NULL!	#NULL!	#NULL!	#NULL!
2,00	2,00	#NULL!	#NULL!	#NULL!	#NULL!	#NULL!
1,00	2,00	#NULL!	#NULL!	#NULL!	#NULL!	#NULL!
2,00	2,00	#NULL!	#NULL!	#NULL!	#NULL!	#NULL!
2,00	2,00	#NULL!	#NULL!	#NULL!	#NULL!	#NULL!
2,00	2,00	#NULL!	#NULL!	#NULL!	#NULL!	#NULL!
2,00	2,00	#NULL!	#NULL!	#NULL!	#NULL!	#NULL!
2,00	2,00	#NULL!	#NULL!	#NULL!	#NULL!	#NULL!
2,00	2,00	#NULL!	#NULL!	#NULL!	#NULL!	#NULL!
2,00	2,00	#NULL!	#NULL!	#NULL!	#NULL!	#NULL!
2,00	2,00	#NULL!	#NULL!	#NULL!	#NULL!	#NULL!
2,00	2,00	#NULL!	#NULL!	#NULL!	#NULL!	#NULL!
1,00	2,00	#NULL!	#NULL!	#NULL!	#NULL!	#NULL!
1,00	2,00	#NULL!	#NULL!	#NULL!	#NULL!	#NULL!
2,00	2,00	#NULL!	#NULL!	#NULL!	#NULL!	#NULL!
2,00	2,00	#NULL!	#NULL!	#NULL!	#NULL!	#NULL!
2,00	2,00	#NULL!	#NULL!	#NULL!	#NULL!	#NULL!
2,00	2,00	#NULL!	#NULL!	#NULL!	#NULL!	#NULL!
2,00	2,00	#NULL!	#NULL!	#NULL!	#NULL!	#NULL!
2,00	2,00	#NULL!	#NULL!	#NULL!	#NULL!	#NULL!
2,00	2,00	#NULL!	#NULL!	#NULL!	#NULL!	#NULL!
2,00	2,00	#NULL!	#NULL!	#NULL!	#NULL!	#NULL!
2,00	2,00	#NULL!	#NULL!	#NULL!	#NULL!	#NULL!
2,00	2,00	#NULL!	#NULL!	#NULL!	#NULL!	#NULL!
2,00	2,00	#NULL!	#NULL!	#NULL!	#NULL!	#NULL!
2,00	2,00	#NULL!	#NULL!	#NULL!	#NULL!	#NULL!
2,00	2,00	#NULL!	#NULL!	#NULL!	#NULL!	#NULL!
1,00	2,00	#NULL!	#NULL!	#NULL!	#NULL!	#NULL!
2,00	2,00	#NULL!	#NULL!	#NULL!	#NULL!	#NULL!
2,00	2,00	#NULL!	#NULL!	#NULL!	#NULL!	#NULL!
2,00	2,00	#NULL!	#NULL!	#NULL!	#NULL!	#NULL!
2,00	2,00	#NULL!	#NULL!	#NULL!	#NULL!	#NULL!
2,00	2,00	#NULL!	#NULL!	#NULL!	#NULL!	#NULL!

2,00	2,00	#NULL!	#NULL!	#NULL!	#NULL!	#NULL!
2,00	2,00	#NULL!	#NULL!	#NULL!	#NULL!	#NULL!
2,00	2,00	#NULL!	#NULL!	#NULL!	#NULL!	#NULL!
2,00	2,00	#NULL!	#NULL!	#NULL!	#NULL!	#NULL!
2,00	2,00	#NULL!	#NULL!	#NULL!	#NULL!	#NULL!
2,00	2,00	#NULL!	#NULL!	#NULL!	#NULL!	#NULL!
2,00	2,00	#NULL!	#NULL!	#NULL!	#NULL!	#NULL!
2,00	2,00	#NULL!	#NULL!	#NULL!	#NULL!	#NULL!
2,00	2,00	#NULL!	#NULL!	#NULL!	#NULL!	#NULL!
2,00	2,00	#NULL!	#NULL!	#NULL!	#NULL!	#NULL!
2,00	2,00	#NULL!	#NULL!	#NULL!	#NULL!	#NULL!
1,00	2,00	#NULL!	#NULL!	#NULL!	#NULL!	#NULL!
2,00	2,00	#NULL!	#NULL!	#NULL!	#NULL!	#NULL!
2,00	2,00	#NULL!	#NULL!	#NULL!	#NULL!	#NULL!
1,00	2,00	#NULL!	#NULL!	#NULL!	#NULL!	#NULL!
2,00	2,00	#NULL!	#NULL!	#NULL!	#NULL!	#NULL!
2,00	2,00	#NULL!	#NULL!	#NULL!	#NULL!	#NULL!
2,00	2,00	#NULL!	#NULL!	#NULL!	#NULL!	#NULL!
2,00	2,00	#NULL!	#NULL!	#NULL!	#NULL!	#NULL!
2,00	2,00	#NULL!	#NULL!	#NULL!	#NULL!	#NULL!
2,00	2,00	#NULL!	#NULL!	#NULL!	#NULL!	#NULL!
2,00	2,00	#NULL!	#NULL!	#NULL!	#NULL!	#NULL!
1,00	2,00	#NULL!	#NULL!	#NULL!	#NULL!	#NULL!
2,00	2,00	#NULL!	#NULL!	#NULL!	#NULL!	#NULL!
1,00	2,00	#NULL!	#NULL!	#NULL!	#NULL!	#NULL!
2,00	2,00	#NULL!	#NULL!	#NULL!	#NULL!	#NULL!
2,00	2,00	#NULL!	#NULL!	#NULL!	#NULL!	#NULL!
2,00	2,00	#NULL!	#NULL!	#NULL!	#NULL!	#NULL!
2,00	2,00	#NULL!	#NULL!	#NULL!	#NULL!	#NULL!
2,00	2,00	#NULL!	#NULL!	#NULL!	#NULL!	#NULL!
2,00	2,00	#NULL!	#NULL!	#NULL!	#NULL!	#NULL!
2,00	2,00	#NULL!	#NULL!	#NULL!	#NULL!	#NULL!
2,00	2,00	#NULL!	#NULL!	#NULL!	#NULL!	#NULL!
2,00	2,00	#NULL!	#NULL!	#NULL!	#NULL!	#NULL!
2,00	2,00	#NULL!	#NULL!	#NULL!	#NULL!	#NULL!
2,00	2,00	#NULL!	#NULL!	#NULL!	#NULL!	#NULL!
2,00	2,00	#NULL!	#NULL!	#NULL!	#NULL!	#NULL!
2,00	2,00	#NULL!	#NULL!	#NULL!	#NULL!	#NULL!
2,00	2,00	#NULL!	#NULL!	#NULL!	#NULL!	#NULL!
2,00	2,00	#NULL!	#NULL!	#NULL!	#NULL!	#NULL!
2,00	2,00	#NULL!	#NULL!	#NULL!	#NULL!	#NULL!
2,00	2,00	#NULL!	#NULL!	#NULL!	#NULL!	#NULL!
2,00	2,00	#NULL!	#NULL!	#NULL!	#NULL!	#NULL!
2,00	2,00	#NULL!	#NULL!	#NULL!	#NULL!	#NULL!
2,00	2,00	#NULL!	#NULL!	#NULL!	#NULL!	#NULL!
2,00	2,00	#NULL!	#NULL!	#NULL!	#NULL!	#NULL!
2,00	2,00	#NULL!	#NULL!	#NULL!	#NULL!	#NULL!
2,00	2,00	#NULL!	#NULL!	#NULL!	#NULL!	#NULL!
2,00	2,00	#NULL!	#NULL!	#NULL!	#NULL!	#NULL!
2,00	2,00	#NULL!	#NULL!	#NULL!	#NULL!	#NULL!

2,00	2,00	#NULL!	#NULL!	#NULL!	#NULL!	#NULL!
2,00	2,00	#NULL!	#NULL!	#NULL!	#NULL!	#NULL!
2,00	2,00	#NULL!	#NULL!	#NULL!	#NULL!	#NULL!
2,00	2,00	#NULL!	#NULL!	#NULL!	#NULL!	#NULL!
2,00	2,00	#NULL!	#NULL!	#NULL!	#NULL!	#NULL!
1,00	2,00	#NULL!	#NULL!	#NULL!	#NULL!	#NULL!
2,00	2,00	#NULL!	#NULL!	#NULL!	#NULL!	#NULL!
2,00	2,00	#NULL!	#NULL!	#NULL!	#NULL!	#NULL!
2,00	2,00	#NULL!	#NULL!	#NULL!	#NULL!	#NULL!
2,00	2,00	#NULL!	#NULL!	#NULL!	#NULL!	#NULL!
2,00	2,00	#NULL!	#NULL!	#NULL!	#NULL!	#NULL!
2,00	2,00	#NULL!	#NULL!	#NULL!	#NULL!	#NULL!
1,00	2,00	#NULL!	#NULL!	#NULL!	#NULL!	#NULL!
2,00	2,00	#NULL!	#NULL!	#NULL!	#NULL!	#NULL!
2,00	2,00	#NULL!	#NULL!	#NULL!	#NULL!	#NULL!
2,00	2,00	#NULL!	#NULL!	#NULL!	#NULL!	#NULL!
2,00	2,00	#NULL!	#NULL!	#NULL!	#NULL!	#NULL!
2,00	2,00	#NULL!	#NULL!	#NULL!	#NULL!	#NULL!
2,00	2,00	#NULL!	#NULL!	#NULL!	#NULL!	#NULL!
2,00	2,00	#NULL!	#NULL!	#NULL!	#NULL!	#NULL!
2,00	2,00	#NULL!	#NULL!	#NULL!	#NULL!	#NULL!
2,00	2,00	#NULL!	#NULL!	#NULL!	#NULL!	#NULL!
2,00	2,00	#NULL!	#NULL!	#NULL!	#NULL!	#NULL!
2,00	2,00	#NULL!	#NULL!	#NULL!	#NULL!	#NULL!
2,00	2,00	#NULL!	#NULL!	#NULL!	#NULL!	#NULL!
2,00	2,00	#NULL!	#NULL!	#NULL!	#NULL!	#NULL!
2,00	2,00	#NULL!	#NULL!	#NULL!	#NULL!	#NULL!
2,00	2,00	#NULL!	#NULL!	#NULL!	#NULL!	#NULL!
2,00	2,00	#NULL!	#NULL!	#NULL!	#NULL!	#NULL!
2,00	2,00	#NULL!	#NULL!	#NULL!	#NULL!	#NULL!
2,00	2,00	#NULL!	#NULL!	#NULL!	#NULL!	#NULL!
2,00	2,00	#NULL!	#NULL!	#NULL!	#NULL!	#NULL!
2,00	2,00	#NULL!	#NULL!	#NULL!	#NULL!	#NULL!
2,00	2,00	#NULL!	#NULL!	#NULL!	#NULL!	#NULL!
2,00	2,00	#NULL!	#NULL!	#NULL!	#NULL!	#NULL!
2,00	2,00	#NULL!	#NULL!	#NULL!	#NULL!	#NULL!
2,00	2,00	#NULL!	#NULL!	#NULL!	#NULL!	#NULL!
2,00	2,00	#NULL!	#NULL!	#NULL!	#NULL!	#NULL!
2,00	2,00	#NULL!	#NULL!	#NULL!	#NULL!	#NULL!
2,00	2,00	#NULL!	#NULL!	#NULL!	#NULL!	#NULL!
2,00	2,00	#NULL!	#NULL!	#NULL!	#NULL!	#NULL!
2,00	2,00	#NULL!	#NULL!	#NULL!	#NULL!	#NULL!
2,00	2,00	#NULL!	#NULL!	#NULL!	#NULL!	#NULL!
2,00	2,00	#NULL!	#NULL!	#NULL!	#NULL!	#NULL!
2,00	2,00	#NULL!	#NULL!	#NULL!	#NULL!	#NULL!
2,00	2,00	#NULL!	#NULL!	#NULL!	#NULL!	#NULL!
2,00	2,00	#NULL!	#NULL!	#NULL!	#NULL!	#NULL!
2,00	2,00	#NULL!	#NULL!	#NULL!	#NULL!	#NULL!
1,00	2,00	#NULL!	#NULL!	#NULL!	#NULL!	#NULL!
2,00	2,00	#NULL!	#NULL!	#NULL!	#NULL!	#NULL!

2,00	2,00	#NULL!	#NULL!	#NULL!	#NULL!	#NULL!
2,00	2,00	#NULL!	#NULL!	#NULL!	#NULL!	#NULL!
2,00	2,00	#NULL!	#NULL!	#NULL!	#NULL!	#NULL!
2,00	2,00	#NULL!	#NULL!	#NULL!	#NULL!	#NULL!
2,00	2,00	#NULL!	#NULL!	#NULL!	#NULL!	#NULL!
2,00	2,00	#NULL!	#NULL!	#NULL!	#NULL!	#NULL!
2,00	2,00	#NULL!	#NULL!	#NULL!	#NULL!	#NULL!
2,00	2,00	#NULL!	#NULL!	#NULL!	#NULL!	#NULL!
2,00	2,00	#NULL!	#NULL!	#NULL!	#NULL!	#NULL!
2,00	2,00	#NULL!	#NULL!	#NULL!	#NULL!	#NULL!
2,00	2,00	#NULL!	#NULL!	#NULL!	#NULL!	#NULL!
2,00	2,00	#NULL!	#NULL!	#NULL!	#NULL!	#NULL!
2,00	2,00	#NULL!	#NULL!	#NULL!	#NULL!	#NULL!
2,00	1,00	1,00	2,00	1,00	2,00	#NULL!
2,00	2,00	#NULL!	#NULL!	#NULL!	#NULL!	#NULL!
2,00	2,00	#NULL!	#NULL!	#NULL!	#NULL!	#NULL!
2,00	2,00	#NULL!	#NULL!	#NULL!	#NULL!	#NULL!
2,00	1,00	1,00	1,00	2,00	2,00	#NULL!
2,00	2,00	#NULL!	#NULL!	#NULL!	#NULL!	#NULL!
2,00	2,00	#NULL!	#NULL!	#NULL!	#NULL!	#NULL!
2,00	2,00	#NULL!	#NULL!	#NULL!	#NULL!	#NULL!
2,00	2,00	#NULL!	#NULL!	#NULL!	#NULL!	#NULL!
2,00	2,00	#NULL!	#NULL!	#NULL!	#NULL!	#NULL!
2,00	2,00	#NULL!	#NULL!	#NULL!	#NULL!	#NULL!
2,00	2,00	#NULL!	#NULL!	#NULL!	#NULL!	#NULL!
2,00	1,00	1,00	2,00	1,00	2,00	#NULL!
2,00	2,00	#NULL!	#NULL!	#NULL!	#NULL!	#NULL!
2,00	1,00	1,00	1,00	2,00	2,00	#NULL!
2,00	2,00	#NULL!	#NULL!	#NULL!	#NULL!	#NULL!
2,00	2,00	#NULL!	#NULL!	#NULL!	#NULL!	#NULL!
2,00	2,00	#NULL!	#NULL!	#NULL!	#NULL!	#NULL!
2,00	2,00	#NULL!	#NULL!	#NULL!	#NULL!	#NULL!
2,00	2,00	#NULL!	#NULL!	#NULL!	#NULL!	#NULL!
2,00	2,00	#NULL!	#NULL!	#NULL!	#NULL!	#NULL!
2,00	2,00	#NULL!	#NULL!	#NULL!	#NULL!	#NULL!
2,00	2,00	#NULL!	#NULL!	#NULL!	#NULL!	#NULL!
2,00	2,00	#NULL!	#NULL!	#NULL!	#NULL!	#NULL!
2,00	2,00	#NULL!	#NULL!	#NULL!	#NULL!	#NULL!
2,00	2,00	#NULL!	#NULL!	#NULL!	#NULL!	#NULL!
2,00	2,00	#NULL!	#NULL!	#NULL!	#NULL!	#NULL!
2,00	2,00	#NULL!	#NULL!	#NULL!	#NULL!	#NULL!
2,00	2,00	#NULL!	#NULL!	#NULL!	#NULL!	#NULL!
2,00	2,00	#NULL!	#NULL!	#NULL!	#NULL!	#NULL!
2,00	2,00	#NULL!	#NULL!	#NULL!	#NULL!	#NULL!
2,00	2,00	#NULL!	#NULL!	#NULL!	#NULL!	#NULL!
2,00	2,00	#NULL!	#NULL!	#NULL!	#NULL!	#NULL!
2,00	2,00	#NULL!	#NULL!	#NULL!	#NULL!	#NULL!
2,00	2,00	#NULL!	#NULL!	#NULL!	#NULL!	#NULL!
2,00	2,00	#NULL!	#NULL!	#NULL!	#NULL!	#NULL!
2,00	2,00	#NULL!	#NULL!	#NULL!	#NULL!	#NULL!

2,00	2,00	#NULL!	#NULL!	#NULL!	#NULL!	#NULL!
2,00	2,00	#NULL!	#NULL!	#NULL!	#NULL!	#NULL!
2,00	2,00	#NULL!	#NULL!	#NULL!	#NULL!	#NULL!
2,00	2,00	#NULL!	#NULL!	#NULL!	#NULL!	#NULL!
2,00	2,00	#NULL!	#NULL!	#NULL!	#NULL!	#NULL!
2,00	2,00	#NULL!	#NULL!	#NULL!	#NULL!	#NULL!
2,00	2,00	#NULL!	#NULL!	#NULL!	#NULL!	#NULL!
2,00	2,00	#NULL!	#NULL!	#NULL!	#NULL!	#NULL!
2,00	2,00	#NULL!	#NULL!	#NULL!	#NULL!	#NULL!
2,00	2,00	#NULL!	#NULL!	#NULL!	#NULL!	#NULL!
2,00	2,00	#NULL!	#NULL!	#NULL!	#NULL!	#NULL!
2,00	2,00	#NULL!	#NULL!	#NULL!	#NULL!	#NULL!
2,00	2,00	#NULL!	#NULL!	#NULL!	#NULL!	#NULL!
2,00	2,00	#NULL!	#NULL!	#NULL!	#NULL!	#NULL!
2,00	2,00	#NULL!	#NULL!	#NULL!	#NULL!	#NULL!
2,00	2,00	#NULL!	#NULL!	#NULL!	#NULL!	#NULL!
2,00	2,00	#NULL!	#NULL!	#NULL!	#NULL!	#NULL!
2,00	2,00	#NULL!	#NULL!	#NULL!	#NULL!	#NULL!
2,00	2,00	#NULL!	#NULL!	#NULL!	#NULL!	#NULL!
2,00	2,00	#NULL!	#NULL!	#NULL!	#NULL!	#NULL!
2,00	2,00	#NULL!	#NULL!	#NULL!	#NULL!	#NULL!
2,00	2,00	#NULL!	#NULL!	#NULL!	#NULL!	#NULL!
2,00	2,00	#NULL!	#NULL!	#NULL!	#NULL!	#NULL!
2,00	2,00	#NULL!	#NULL!	#NULL!	#NULL!	#NULL!
2,00	2,00	#NULL!	#NULL!	#NULL!	#NULL!	#NULL!
2,00	2,00	#NULL!	#NULL!	#NULL!	#NULL!	#NULL!
2,00	2,00	#NULL!	#NULL!	#NULL!	#NULL!	#NULL!
2,00	2,00	#NULL!	#NULL!	#NULL!	#NULL!	#NULL!
2,00	2,00	#NULL!	#NULL!	#NULL!	#NULL!	#NULL!
2,00	2,00	#NULL!	#NULL!	#NULL!	#NULL!	#NULL!
2,00	2,00	#NULL!	#NULL!	#NULL!	#NULL!	#NULL!
2,00	2,00	#NULL!	#NULL!	#NULL!	#NULL!	#NULL!
2,00	2,00	#NULL!	#NULL!	#NULL!	#NULL!	#NULL!
2,00	2,00	#NULL!	#NULL!	#NULL!	#NULL!	#NULL!
2,00	2,00	#NULL!	#NULL!	#NULL!	#NULL!	#NULL!
2,00	2,00	#NULL!	#NULL!	#NULL!	#NULL!	#NULL!
2,00	2,00	#NULL!	#NULL!	#NULL!	#NULL!	#NULL!
2,00	2,00	#NULL!	#NULL!	#NULL!	#NULL!	#NULL!
2,00	2,00	#NULL!	#NULL!	#NULL!	#NULL!	#NULL!
2,00	2,00	#NULL!	#NULL!	#NULL!	#NULL!	#NULL!
1,00	2,00	#NULL!	#NULL!	#NULL!	#NULL!	#NULL!
2,00	2,00	#NULL!	#NULL!	#NULL!	#NULL!	#NULL!
1,00	2,00	#NULL!	#NULL!	#NULL!	#NULL!	#NULL!
2,00	2,00	#NULL!	#NULL!	#NULL!	#NULL!	#NULL!
2,00	2,00	#NULL!	#NULL!	#NULL!	#NULL!	#NULL!
2,00	2,00	#NULL!	#NULL!	#NULL!	#NULL!	#NULL!
2,00	2,00	#NULL!	#NULL!	#NULL!	#NULL!	#NULL!
2,00	2,00	#NULL!	#NULL!	#NULL!	#NULL!	#NULL!
2,00	2,00	#NULL!	#NULL!	#NULL!	#NULL!	#NULL!
2,00	2,00	#NULL!	#NULL!	#NULL!	#NULL!	#NULL!

2,00	2,00	#NULL!	#NULL!	#NULL!	#NULL!	#NULL!
2,00	2,00	#NULL!	#NULL!	#NULL!	#NULL!	#NULL!
1,00	2,00	#NULL!	#NULL!	#NULL!	#NULL!	#NULL!
2,00	2,00	#NULL!	#NULL!	#NULL!	#NULL!	#NULL!
2,00	2,00	#NULL!	#NULL!	#NULL!	#NULL!	#NULL!
2,00	2,00	#NULL!	#NULL!	#NULL!	#NULL!	#NULL!
2,00	2,00	#NULL!	#NULL!	#NULL!	#NULL!	#NULL!
2,00	2,00	#NULL!	#NULL!	#NULL!	#NULL!	#NULL!
2,00	2,00	#NULL!	#NULL!	#NULL!	#NULL!	#NULL!
2,00	2,00	#NULL!	#NULL!	#NULL!	#NULL!	#NULL!
2,00	2,00	#NULL!	#NULL!	#NULL!	#NULL!	#NULL!
2,00	2,00	#NULL!	#NULL!	#NULL!	#NULL!	#NULL!
2,00	2,00	#NULL!	#NULL!	#NULL!	#NULL!	#NULL!
2,00	2,00	#NULL!	#NULL!	#NULL!	#NULL!	#NULL!
2,00	2,00	#NULL!	#NULL!	#NULL!	#NULL!	#NULL!
2,00	2,00	#NULL!	#NULL!	#NULL!	#NULL!	#NULL!
1,00	2,00	#NULL!	#NULL!	#NULL!	#NULL!	#NULL!
1,00	2,00	#NULL!	#NULL!	#NULL!	#NULL!	#NULL!
2,00	2,00	#NULL!	#NULL!	#NULL!	#NULL!	#NULL!
2,00	2,00	#NULL!	#NULL!	#NULL!	#NULL!	#NULL!
2,00	2,00	#NULL!	#NULL!	#NULL!	#NULL!	#NULL!
2,00	2,00	#NULL!	#NULL!	#NULL!	#NULL!	#NULL!
2,00	2,00	#NULL!	#NULL!	#NULL!	#NULL!	#NULL!
2,00	2,00	#NULL!	#NULL!	#NULL!	#NULL!	#NULL!
2,00	2,00	#NULL!	#NULL!	#NULL!	#NULL!	#NULL!
1,00	2,00	#NULL!	#NULL!	#NULL!	#NULL!	#NULL!
2,00	2,00	#NULL!	#NULL!	#NULL!	#NULL!	#NULL!
1,00	2,00	#NULL!	#NULL!	#NULL!	#NULL!	#NULL!
1,00	2,00	#NULL!	#NULL!	#NULL!	#NULL!	#NULL!
2,00	2,00	#NULL!	#NULL!	#NULL!	#NULL!	#NULL!
2,00	2,00	#NULL!	#NULL!	#NULL!	#NULL!	#NULL!
2,00	2,00	#NULL!	#NULL!	#NULL!	#NULL!	#NULL!
2,00	2,00	#NULL!	#NULL!	#NULL!	#NULL!	#NULL!
2,00	2,00	#NULL!	#NULL!	#NULL!	#NULL!	#NULL!
2,00	2,00	#NULL!	#NULL!	#NULL!	#NULL!	#NULL!
2,00	2,00	#NULL!	#NULL!	#NULL!	#NULL!	#NULL!
2,00	2,00	#NULL!	#NULL!	#NULL!	#NULL!	#NULL!
2,00	2,00	#NULL!	#NULL!	#NULL!	#NULL!	#NULL!
2,00	2,00	#NULL!	#NULL!	#NULL!	#NULL!	#NULL!
2,00	2,00	#NULL!	#NULL!	#NULL!	#NULL!	#NULL!
2,00	2,00	#NULL!	#NULL!	#NULL!	#NULL!	#NULL!
2,00	2,00	#NULL!	#NULL!	#NULL!	#NULL!	#NULL!
2,00	2,00	#NULL!	#NULL!	#NULL!	#NULL!	#NULL!
2,00	2,00	#NULL!	#NULL!	#NULL!	#NULL!	#NULL!
2,00	2,00	#NULL!	#NULL!	#NULL!	#NULL!	#NULL!
2,00	2,00	#NULL!	#NULL!	#NULL!	#NULL!	#NULL!
2,00	2,00	#NULL!	#NULL!	#NULL!	#NULL!	#NULL!
2,00	2,00	#NULL!	#NULL!	#NULL!	#NULL!	#NULL!
2,00	2,00	#NULL!	#NULL!	#NULL!	#NULL!	#NULL!
2,00	2,00	#NULL!	#NULL!	#NULL!	#NULL!	#NULL!

2,00	2,00	#NULL!	#NULL!	#NULL!	#NULL!	#NULL!
2,00	2,00	#NULL!	#NULL!	#NULL!	#NULL!	#NULL!
2,00	2,00	#NULL!	#NULL!	#NULL!	#NULL!	#NULL!
1,00	2,00	#NULL!	#NULL!	#NULL!	#NULL!	#NULL!
2,00	2,00	#NULL!	#NULL!	#NULL!	#NULL!	#NULL!
2,00	2,00	#NULL!	#NULL!	#NULL!	#NULL!	#NULL!
2,00	2,00	#NULL!	#NULL!	#NULL!	#NULL!	#NULL!
1,00	2,00	#NULL!	#NULL!	#NULL!	#NULL!	#NULL!
1,00	2,00	#NULL!	#NULL!	#NULL!	#NULL!	#NULL!
2,00	2,00	#NULL!	#NULL!	#NULL!	#NULL!	#NULL!
1,00	2,00	#NULL!	#NULL!	#NULL!	#NULL!	#NULL!
2,00	2,00	#NULL!	#NULL!	#NULL!	#NULL!	#NULL!
2,00	2,00	#NULL!	#NULL!	#NULL!	#NULL!	#NULL!
2,00	2,00	#NULL!	#NULL!	#NULL!	#NULL!	#NULL!
2,00	2,00	#NULL!	#NULL!	#NULL!	#NULL!	#NULL!
2,00	2,00	#NULL!	#NULL!	#NULL!	#NULL!	#NULL!
1,00	2,00	#NULL!	#NULL!	#NULL!	#NULL!	#NULL!
1,00	2,00	#NULL!	#NULL!	#NULL!	#NULL!	#NULL!
1,00	2,00	#NULL!	#NULL!	#NULL!	#NULL!	#NULL!
2,00	2,00	#NULL!	#NULL!	#NULL!	#NULL!	#NULL!
2,00	2,00	#NULL!	#NULL!	#NULL!	#NULL!	#NULL!
2,00	2,00	#NULL!	#NULL!	#NULL!	#NULL!	#NULL!
2,00	2,00	#NULL!	#NULL!	#NULL!	#NULL!	#NULL!
2,00	2,00	#NULL!	#NULL!	#NULL!	#NULL!	#NULL!
2,00	2,00	#NULL!	#NULL!	#NULL!	#NULL!	#NULL!
2,00	2,00	#NULL!	#NULL!	#NULL!	#NULL!	#NULL!
2,00	2,00	#NULL!	#NULL!	#NULL!	#NULL!	#NULL!
2,00	2,00	#NULL!	#NULL!	#NULL!	#NULL!	#NULL!
2,00	2,00	#NULL!	#NULL!	#NULL!	#NULL!	#NULL!
2,00	2,00	#NULL!	#NULL!	#NULL!	#NULL!	#NULL!
2,00	2,00	#NULL!	#NULL!	#NULL!	#NULL!	#NULL!
2,00	2,00	#NULL!	#NULL!	#NULL!	#NULL!	#NULL!
2,00	2,00	#NULL!	#NULL!	#NULL!	#NULL!	#NULL!
1,00	2,00	#NULL!	#NULL!	#NULL!	#NULL!	#NULL!
1,00	2,00	#NULL!	#NULL!	#NULL!	#NULL!	#NULL!
2,00	2,00	#NULL!	#NULL!	#NULL!	#NULL!	#NULL!
2,00	2,00	#NULL!	#NULL!	#NULL!	#NULL!	#NULL!
2,00	2,00	#NULL!	#NULL!	#NULL!	#NULL!	#NULL!
2,00	2,00	#NULL!	#NULL!	#NULL!	#NULL!	#NULL!
1,00	2,00	#NULL!	#NULL!	#NULL!	#NULL!	#NULL!
2,00	2,00	#NULL!	#NULL!	#NULL!	#NULL!	#NULL!
2,00	2,00	#NULL!	#NULL!	#NULL!	#NULL!	#NULL!
2,00	2,00	#NULL!	#NULL!	#NULL!	#NULL!	#NULL!
2,00	2,00	#NULL!	#NULL!	#NULL!	#NULL!	#NULL!
2,00	2,00	#NULL!	#NULL!	#NULL!	#NULL!	#NULL!
1,00	2,00	#NULL!	#NULL!	#NULL!	#NULL!	#NULL!
2,00	2,00	#NULL!	#NULL!	#NULL!	#NULL!	#NULL!
2,00	2,00	#NULL!	#NULL!	#NULL!	#NULL!	#NULL!
2,00	2,00	#NULL!	#NULL!	#NULL!	#NULL!	#NULL!
2,00	1,00	1,00	1,00	2,00	2,00	#NULL!

2,00	2,00	#NULL!	#NULL!	#NULL!	#NULL!	#NULL!
2,00	2,00	#NULL!	#NULL!	#NULL!	#NULL!	#NULL!
2,00	2,00	#NULL!	#NULL!	#NULL!	#NULL!	#NULL!
2,00	1,00	1,00	1,00	2,00	2,00	#NULL!
2,00	1,00	1,00	1,00	2,00	2,00	#NULL!
2,00	2,00	#NULL!	#NULL!	#NULL!	#NULL!	#NULL!
2,00	2,00	#NULL!	#NULL!	#NULL!	#NULL!	#NULL!
2,00	1,00	1,00	1,00	2,00	2,00	#NULL!
2,00	2,00	#NULL!	#NULL!	#NULL!	#NULL!	#NULL!
2,00	2,00	#NULL!	#NULL!	#NULL!	#NULL!	#NULL!
2,00	2,00	#NULL!	#NULL!	#NULL!	#NULL!	#NULL!
2,00	2,00	#NULL!	#NULL!	#NULL!	#NULL!	#NULL!
2,00	2,00	#NULL!	#NULL!	#NULL!	#NULL!	#NULL!
1,00	2,00	#NULL!	#NULL!	#NULL!	#NULL!	#NULL!
2,00	2,00	#NULL!	#NULL!	#NULL!	#NULL!	#NULL!
2,00	2,00	#NULL!	#NULL!	#NULL!	#NULL!	#NULL!
2,00	2,00	#NULL!	#NULL!	#NULL!	#NULL!	#NULL!
2,00	2,00	#NULL!	#NULL!	#NULL!	#NULL!	#NULL!
2,00	2,00	#NULL!	#NULL!	#NULL!	#NULL!	#NULL!
2,00	2,00	#NULL!	#NULL!	#NULL!	#NULL!	#NULL!
2,00	2,00	#NULL!	#NULL!	#NULL!	#NULL!	#NULL!
2,00	2,00	#NULL!	#NULL!	#NULL!	#NULL!	#NULL!
2,00	2,00	#NULL!	#NULL!	#NULL!	#NULL!	#NULL!
2,00	2,00	#NULL!	#NULL!	#NULL!	#NULL!	#NULL!
2,00	2,00	#NULL!	#NULL!	#NULL!	#NULL!	#NULL!
2,00	2,00	#NULL!	#NULL!	#NULL!	#NULL!	#NULL!
2,00	2,00	#NULL!	#NULL!	#NULL!	#NULL!	#NULL!
2,00	2,00	#NULL!	#NULL!	#NULL!	#NULL!	#NULL!
2,00	2,00	#NULL!	#NULL!	#NULL!	#NULL!	#NULL!
2,00	2,00	#NULL!	#NULL!	#NULL!	#NULL!	#NULL!
2,00	2,00	#NULL!	#NULL!	#NULL!	#NULL!	#NULL!
2,00	2,00	#NULL!	#NULL!	#NULL!	#NULL!	#NULL!
2,00	2,00	#NULL!	#NULL!	#NULL!	#NULL!	#NULL!
1,00	2,00	#NULL!	#NULL!	#NULL!	#NULL!	#NULL!
2,00	2,00	#NULL!	#NULL!	#NULL!	#NULL!	#NULL!
2,00	2,00	#NULL!	#NULL!	#NULL!	#NULL!	#NULL!
2,00	2,00	#NULL!	#NULL!	#NULL!	#NULL!	#NULL!
2,00	2,00	#NULL!	#NULL!	#NULL!	#NULL!	#NULL!
2,00	2,00	#NULL!	#NULL!	#NULL!	#NULL!	#NULL!
2,00	2,00	#NULL!	#NULL!	#NULL!	#NULL!	#NULL!
2,00	2,00	#NULL!	#NULL!	#NULL!	#NULL!	#NULL!
2,00	2,00	#NULL!	#NULL!	#NULL!	#NULL!	#NULL!
2,00	2,00	#NULL!	#NULL!	#NULL!	#NULL!	#NULL!
2,00	2,00	#NULL!	#NULL!	#NULL!	#NULL!	#NULL!
2,00	2,00	#NULL!	#NULL!	#NULL!	#NULL!	#NULL!
2,00	2,00	#NULL!	#NULL!	#NULL!	#NULL!	#NULL!
2,00	2,00	#NULL!	#NULL!	#NULL!	#NULL!	#NULL!
2,00	2,00	#NULL!	#NULL!	#NULL!	#NULL!	#NULL!
2,00	2,00	#NULL!	#NULL!	#NULL!	#NULL!	#NULL!
2,00	2,00	#NULL!	#NULL!	#NULL!	#NULL!	#NULL!

2,00	2,00	#NULL!	#NULL!	#NULL!	#NULL!	#NULL!
2,00	2,00	#NULL!	#NULL!	#NULL!	#NULL!	#NULL!
2,00	2,00	#NULL!	#NULL!	#NULL!	#NULL!	#NULL!
2,00	2,00	#NULL!	#NULL!	#NULL!	#NULL!	#NULL!
2,00	2,00	#NULL!	#NULL!	#NULL!	#NULL!	#NULL!
2,00	2,00	#NULL!	#NULL!	#NULL!	#NULL!	#NULL!
2,00	2,00	#NULL!	#NULL!	#NULL!	#NULL!	#NULL!
2,00	2,00	#NULL!	#NULL!	#NULL!	#NULL!	#NULL!
2,00	2,00	#NULL!	#NULL!	#NULL!	#NULL!	#NULL!
2,00	2,00	#NULL!	#NULL!	#NULL!	#NULL!	#NULL!
2,00	2,00	#NULL!	#NULL!	#NULL!	#NULL!	#NULL!
2,00	2,00	#NULL!	#NULL!	#NULL!	#NULL!	#NULL!
2,00	2,00	#NULL!	#NULL!	#NULL!	#NULL!	#NULL!
2,00	2,00	#NULL!	#NULL!	#NULL!	#NULL!	#NULL!
2,00	2,00	#NULL!	#NULL!	#NULL!	#NULL!	#NULL!
2,00	2,00	#NULL!	#NULL!	#NULL!	#NULL!	#NULL!
2,00	2,00	#NULL!	#NULL!	#NULL!	#NULL!	#NULL!
2,00	2,00	#NULL!	#NULL!	#NULL!	#NULL!	#NULL!
2,00	2,00	#NULL!	#NULL!	#NULL!	#NULL!	#NULL!
2,00	2,00	#NULL!	#NULL!	#NULL!	#NULL!	#NULL!
2,00	2,00	#NULL!	#NULL!	#NULL!	#NULL!	#NULL!
2,00	2,00	#NULL!	#NULL!	#NULL!	#NULL!	#NULL!
2,00	2,00	#NULL!	#NULL!	#NULL!	#NULL!	#NULL!
2,00	2,00	#NULL!	#NULL!	#NULL!	#NULL!	#NULL!
1,00	2,00	#NULL!	#NULL!	#NULL!	#NULL!	#NULL!
2,00	2,00	#NULL!	#NULL!	#NULL!	#NULL!	#NULL!
2,00	2,00	#NULL!	#NULL!	#NULL!	#NULL!	#NULL!
2,00	2,00	#NULL!	#NULL!	#NULL!	#NULL!	#NULL!
2,00	2,00	#NULL!	#NULL!	#NULL!	#NULL!	#NULL!
2,00	2,00	#NULL!	#NULL!	#NULL!	#NULL!	#NULL!
2,00	2,00	#NULL!	#NULL!	#NULL!	#NULL!	#NULL!
2,00	2,00	#NULL!	#NULL!	#NULL!	#NULL!	#NULL!
2,00	2,00	#NULL!	#NULL!	#NULL!	#NULL!	#NULL!
2,00	2,00	#NULL!	#NULL!	#NULL!	#NULL!	#NULL!
2,00	2,00	#NULL!	#NULL!	#NULL!	#NULL!	#NULL!
2,00	2,00	#NULL!	#NULL!	#NULL!	#NULL!	#NULL!
2,00	2,00	#NULL!	#NULL!	#NULL!	#NULL!	#NULL!
2,00	2,00	#NULL!	#NULL!	#NULL!	#NULL!	#NULL!
2,00	2,00	#NULL!	#NULL!	#NULL!	#NULL!	#NULL!
2,00	2,00	#NULL!	#NULL!	#NULL!	#NULL!	#NULL!
2,00	2,00	#NULL!	#NULL!	#NULL!	#NULL!	#NULL!
2,00	2,00	#NULL!	#NULL!	#NULL!	#NULL!	#NULL!
2,00	2,00	#NULL!	#NULL!	#NULL!	#NULL!	#NULL!
2,00	2,00	#NULL!	#NULL!	#NULL!	#NULL!	#NULL!
2,00	2,00	#NULL!	#NULL!	#NULL!	#NULL!	#NULL!
2,00	2,00	#NULL!	#NULL!	#NULL!	#NULL!	#NULL!
2,00	2,00	#NULL!	#NULL!	#NULL!	#NULL!	#NULL!
2,00	2,00	#NULL!	#NULL!	#NULL!	#NULL!	#NULL!
2,00	2,00	#NULL!	#NULL!	#NULL!	#NULL!	#NULL!
2,00	2,00	#NULL!	#NULL!	#NULL!	#NULL!	#NULL!

2,00	2,00	#NULL!	#NULL!	#NULL!	#NULL!	#NULL!
2,00	2,00	#NULL!	#NULL!	#NULL!	#NULL!	#NULL!
2,00	2,00	#NULL!	#NULL!	#NULL!	#NULL!	#NULL!
2,00	2,00	#NULL!	#NULL!	#NULL!	#NULL!	#NULL!
2,00	1,00	1,00	1,00	2,00	2,00	#NULL!
2,00	2,00	#NULL!	#NULL!	#NULL!	#NULL!	#NULL!
2,00	2,00	#NULL!	#NULL!	#NULL!	#NULL!	#NULL!
2,00	2,00	#NULL!	#NULL!	#NULL!	#NULL!	#NULL!
2,00	2,00	#NULL!	#NULL!	#NULL!	#NULL!	#NULL!
2,00	2,00	#NULL!	#NULL!	#NULL!	#NULL!	#NULL!
2,00	2,00	#NULL!	#NULL!	#NULL!	#NULL!	#NULL!
2,00	2,00	#NULL!	#NULL!	#NULL!	#NULL!	#NULL!
2,00	2,00	#NULL!	#NULL!	#NULL!	#NULL!	#NULL!
2,00	2,00	#NULL!	#NULL!	#NULL!	#NULL!	#NULL!
2,00	2,00	#NULL!	#NULL!	#NULL!	#NULL!	#NULL!
2,00	2,00	#NULL!	#NULL!	#NULL!	#NULL!	#NULL!
2,00	2,00	#NULL!	#NULL!	#NULL!	#NULL!	#NULL!
2,00	2,00	#NULL!	#NULL!	#NULL!	#NULL!	#NULL!
2,00	2,00	#NULL!	#NULL!	#NULL!	#NULL!	#NULL!
2,00	2,00	#NULL!	#NULL!	#NULL!	#NULL!	#NULL!
2,00	2,00	#NULL!	#NULL!	#NULL!	#NULL!	#NULL!
2,00	2,00	#NULL!	#NULL!	#NULL!	#NULL!	#NULL!
2,00	2,00	#NULL!	#NULL!	#NULL!	#NULL!	#NULL!
2,00	2,00	#NULL!	#NULL!	#NULL!	#NULL!	#NULL!
2,00	2,00	#NULL!	#NULL!	#NULL!	#NULL!	#NULL!
1,00	2,00	#NULL!	#NULL!	#NULL!	#NULL!	#NULL!
2,00	2,00	#NULL!	#NULL!	#NULL!	#NULL!	#NULL!
2,00	2,00	#NULL!	#NULL!	#NULL!	#NULL!	#NULL!
2,00	2,00	#NULL!	#NULL!	#NULL!	#NULL!	#NULL!
2,00	2,00	#NULL!	#NULL!	#NULL!	#NULL!	#NULL!
2,00	2,00	#NULL!	#NULL!	#NULL!	#NULL!	#NULL!
2,00	2,00	#NULL!	#NULL!	#NULL!	#NULL!	#NULL!
2,00	2,00	#NULL!	#NULL!	#NULL!	#NULL!	#NULL!
2,00	2,00	#NULL!	#NULL!	#NULL!	#NULL!	#NULL!
2,00	2,00	#NULL!	#NULL!	#NULL!	#NULL!	#NULL!
1,00	2,00	#NULL!	#NULL!	#NULL!	#NULL!	#NULL!
2,00	2,00	#NULL!	#NULL!	#NULL!	#NULL!	#NULL!
2,00	2,00	#NULL!	#NULL!	#NULL!	#NULL!	#NULL!
2,00	2,00	#NULL!	#NULL!	#NULL!	#NULL!	#NULL!
2,00	2,00	#NULL!	#NULL!	#NULL!	#NULL!	#NULL!
2,00	2,00	#NULL!	#NULL!	#NULL!	#NULL!	#NULL!
2,00	2,00	#NULL!	#NULL!	#NULL!	#NULL!	#NULL!
2,00	2,00	#NULL!	#NULL!	#NULL!	#NULL!	#NULL!
2,00	2,00	#NULL!	#NULL!	#NULL!	#NULL!	#NULL!
2,00	2,00	#NULL!	#NULL!	#NULL!	#NULL!	#NULL!
2,00	2,00	#NULL!	#NULL!	#NULL!	#NULL!	#NULL!
2,00	2,00	#NULL!	#NULL!	#NULL!	#NULL!	#NULL!
2,00	2,00	#NULL!	#NULL!	#NULL!	#NULL!	#NULL!
2,00	2,00	#NULL!	#NULL!	#NULL!	#NULL!	#NULL!
2,00	2,00	#NULL!	#NULL!	#NULL!	#NULL!	#NULL!

2,00	2,00	#NULL!	#NULL!	#NULL!	#NULL!	#NULL!
2,00	2,00	#NULL!	#NULL!	#NULL!	#NULL!	#NULL!
1,00	2,00	#NULL!	#NULL!	#NULL!	#NULL!	#NULL!
2,00	2,00	#NULL!	#NULL!	#NULL!	#NULL!	#NULL!
2,00	2,00	#NULL!	#NULL!	#NULL!	#NULL!	#NULL!
2,00	2,00	#NULL!	#NULL!	#NULL!	#NULL!	#NULL!
2,00	2,00	#NULL!	#NULL!	#NULL!	#NULL!	#NULL!
2,00	2,00	#NULL!	#NULL!	#NULL!	#NULL!	#NULL!
2,00	2,00	#NULL!	#NULL!	#NULL!	#NULL!	#NULL!
2,00	2,00	#NULL!	#NULL!	#NULL!	#NULL!	#NULL!
2,00	2,00	#NULL!	#NULL!	#NULL!	#NULL!	#NULL!
2,00	2,00	#NULL!	#NULL!	#NULL!	#NULL!	#NULL!
2,00	2,00	#NULL!	#NULL!	#NULL!	#NULL!	#NULL!
1,00	2,00	#NULL!	#NULL!	#NULL!	#NULL!	#NULL!
2,00	2,00	#NULL!	#NULL!	#NULL!	#NULL!	#NULL!
2,00	2,00	#NULL!	#NULL!	#NULL!	#NULL!	#NULL!
2,00	2,00	#NULL!	#NULL!	#NULL!	#NULL!	#NULL!
2,00	2,00	#NULL!	#NULL!	#NULL!	#NULL!	#NULL!
2,00	2,00	#NULL!	#NULL!	#NULL!	#NULL!	#NULL!
2,00	2,00	#NULL!	#NULL!	#NULL!	#NULL!	#NULL!
2,00	2,00	#NULL!	#NULL!	#NULL!	#NULL!	#NULL!
2,00	2,00	#NULL!	#NULL!	#NULL!	#NULL!	#NULL!
2,00	2,00	#NULL!	#NULL!	#NULL!	#NULL!	#NULL!
2,00	2,00	#NULL!	#NULL!	#NULL!	#NULL!	#NULL!
2,00	2,00	#NULL!	#NULL!	#NULL!	#NULL!	#NULL!
2,00	2,00	#NULL!	#NULL!	#NULL!	#NULL!	#NULL!
2,00	2,00	#NULL!	#NULL!	#NULL!	#NULL!	#NULL!
2,00	2,00	#NULL!	#NULL!	#NULL!	#NULL!	#NULL!
2,00	2,00	#NULL!	#NULL!	#NULL!	#NULL!	#NULL!
2,00	2,00	#NULL!	#NULL!	#NULL!	#NULL!	#NULL!
2,00	2,00	#NULL!	#NULL!	#NULL!	#NULL!	#NULL!
2,00	2,00	#NULL!	#NULL!	#NULL!	#NULL!	#NULL!
2,00	2,00	#NULL!	#NULL!	#NULL!	#NULL!	#NULL!
2,00	2,00	#NULL!	#NULL!	#NULL!	#NULL!	#NULL!
2,00	2,00	#NULL!	#NULL!	#NULL!	#NULL!	#NULL!
2,00	2,00	#NULL!	#NULL!	#NULL!	#NULL!	#NULL!
2,00	2,00	#NULL!	#NULL!	#NULL!	#NULL!	#NULL!
2,00	2,00	#NULL!	#NULL!	#NULL!	#NULL!	#NULL!
2,00	2,00	#NULL!	#NULL!	#NULL!	#NULL!	#NULL!
2,00	2,00	#NULL!	#NULL!	#NULL!	#NULL!	#NULL!
2,00	2,00	#NULL!	#NULL!	#NULL!	#NULL!	#NULL!
2,00	2,00	#NULL!	#NULL!	#NULL!	#NULL!	#NULL!
2,00	2,00	#NULL!	#NULL!	#NULL!	#NULL!	#NULL!
2,00	2,00	#NULL!	#NULL!	#NULL!	#NULL!	#NULL!
2,00	2,00	#NULL!	#NULL!	#NULL!	#NULL!	#NULL!
2,00	2,00	#NULL!	#NULL!	#NULL!	#NULL!	#NULL!
2,00	2,00	#NULL!	#NULL!	#NULL!	#NULL!	#NULL!
2,00	2,00	#NULL!	#NULL!	#NULL!	#NULL!	#NULL!
2,00	2,00	#NULL!	#NULL!	#NULL!	#NULL!	#NULL!
2,00	2,00	#NULL!	#NULL!	#NULL!	#NULL!	#NULL!

2,00	2,00	#NULL!	#NULL!	#NULL!	#NULL!	#NULL!
2,00	2,00	#NULL!	#NULL!	#NULL!	#NULL!	#NULL!
2,00	2,00	#NULL!	#NULL!	#NULL!	#NULL!	#NULL!
2,00	2,00	#NULL!	#NULL!	#NULL!	#NULL!	#NULL!
2,00	2,00	#NULL!	#NULL!	#NULL!	#NULL!	#NULL!
2,00	2,00	#NULL!	#NULL!	#NULL!	#NULL!	#NULL!
2,00	2,00	#NULL!	#NULL!	#NULL!	#NULL!	#NULL!
1,00	2,00	#NULL!	#NULL!	#NULL!	#NULL!	#NULL!
2,00	2,00	#NULL!	#NULL!	#NULL!	#NULL!	#NULL!
2,00	2,00	#NULL!	#NULL!	#NULL!	#NULL!	#NULL!
2,00	2,00	#NULL!	#NULL!	#NULL!	#NULL!	#NULL!
2,00	2,00	#NULL!	#NULL!	#NULL!	#NULL!	#NULL!
2,00	2,00	#NULL!	#NULL!	#NULL!	#NULL!	#NULL!
2,00	2,00	#NULL!	#NULL!	#NULL!	#NULL!	#NULL!
2,00	2,00	#NULL!	#NULL!	#NULL!	#NULL!	#NULL!
1,00	2,00	#NULL!	#NULL!	#NULL!	#NULL!	#NULL!
2,00	2,00	#NULL!	#NULL!	#NULL!	#NULL!	#NULL!
2,00	2,00	#NULL!	#NULL!	#NULL!	#NULL!	#NULL!
2,00	2,00	#NULL!	#NULL!	#NULL!	#NULL!	#NULL!
2,00	2,00	#NULL!	#NULL!	#NULL!	#NULL!	#NULL!
2,00	2,00	#NULL!	#NULL!	#NULL!	#NULL!	#NULL!
2,00	2,00	#NULL!	#NULL!	#NULL!	#NULL!	#NULL!
2,00	2,00	#NULL!	#NULL!	#NULL!	#NULL!	#NULL!
2,00	2,00	#NULL!	#NULL!	#NULL!	#NULL!	#NULL!
2,00	2,00	#NULL!	#NULL!	#NULL!	#NULL!	#NULL!
2,00	2,00	#NULL!	#NULL!	#NULL!	#NULL!	#NULL!
2,00	2,00	#NULL!	#NULL!	#NULL!	#NULL!	#NULL!
2,00	2,00	#NULL!	#NULL!	#NULL!	#NULL!	#NULL!
2,00	2,00	#NULL!	#NULL!	#NULL!	#NULL!	#NULL!
2,00	2,00	#NULL!	#NULL!	#NULL!	#NULL!	#NULL!
1,00	2,00	#NULL!	#NULL!	#NULL!	#NULL!	#NULL!
2,00	2,00	#NULL!	#NULL!	#NULL!	#NULL!	#NULL!
2,00	2,00	#NULL!	#NULL!	#NULL!	#NULL!	#NULL!
2,00	2,00	#NULL!	#NULL!	#NULL!	#NULL!	#NULL!
2,00	2,00	#NULL!	#NULL!	#NULL!	#NULL!	#NULL!
1,00	2,00	#NULL!	#NULL!	#NULL!	#NULL!	#NULL!
2,00	2,00	#NULL!	#NULL!	#NULL!	#NULL!	#NULL!
2,00	2,00	#NULL!	#NULL!	#NULL!	#NULL!	#NULL!
2,00	2,00	#NULL!	#NULL!	#NULL!	#NULL!	#NULL!
1,00	2,00	#NULL!	#NULL!	#NULL!	#NULL!	#NULL!
2,00	2,00	#NULL!	#NULL!	#NULL!	#NULL!	#NULL!
2,00	2,00	#NULL!	#NULL!	#NULL!	#NULL!	#NULL!
2,00	2,00	#NULL!	#NULL!	#NULL!	#NULL!	#NULL!
2,00	2,00	#NULL!	#NULL!	#NULL!	#NULL!	#NULL!
2,00	2,00	#NULL!	#NULL!	#NULL!	#NULL!	#NULL!
2,00	2,00	#NULL!	#NULL!	#NULL!	#NULL!	#NULL!
2,00	2,00	#NULL!	#NULL!	#NULL!	#NULL!	#NULL!
2,00	2,00	#NULL!	#NULL!	#NULL!	#NULL!	#NULL!
2,00	2,00	#NULL!	#NULL!	#NULL!	#NULL!	#NULL!
2,00	2,00	#NULL!	#NULL!	#NULL!	#NULL!	#NULL!

2,00	2,00	#NULL!	#NULL!	#NULL!	#NULL!	#NULL!
2,00	2,00	#NULL!	#NULL!	#NULL!	#NULL!	#NULL!
1,00	2,00	#NULL!	#NULL!	#NULL!	#NULL!	#NULL!
2,00	1,00	1,00	1,00	2,00	2,00	#NULL!
2,00	2,00	#NULL!	#NULL!	#NULL!	#NULL!	#NULL!
2,00	2,00	#NULL!	#NULL!	#NULL!	#NULL!	#NULL!
2,00	2,00	#NULL!	#NULL!	#NULL!	#NULL!	#NULL!
2,00	2,00	#NULL!	#NULL!	#NULL!	#NULL!	#NULL!
2,00	2,00	#NULL!	#NULL!	#NULL!	#NULL!	#NULL!
2,00	2,00	#NULL!	#NULL!	#NULL!	#NULL!	#NULL!
2,00	2,00	#NULL!	#NULL!	#NULL!	#NULL!	#NULL!
2,00	2,00	#NULL!	#NULL!	#NULL!	#NULL!	#NULL!
2,00	2,00	#NULL!	#NULL!	#NULL!	#NULL!	#NULL!
2,00	2,00	#NULL!	#NULL!	#NULL!	#NULL!	#NULL!
2,00	2,00	#NULL!	#NULL!	#NULL!	#NULL!	#NULL!
2,00	2,00	#NULL!	#NULL!	#NULL!	#NULL!	#NULL!
2,00	2,00	#NULL!	#NULL!	#NULL!	#NULL!	#NULL!
2,00	2,00	#NULL!	#NULL!	#NULL!	#NULL!	#NULL!
2,00	2,00	#NULL!	#NULL!	#NULL!	#NULL!	#NULL!
2,00	2,00	#NULL!	#NULL!	#NULL!	#NULL!	#NULL!
2,00	2,00	#NULL!	#NULL!	#NULL!	#NULL!	#NULL!
2,00	2,00	#NULL!	#NULL!	#NULL!	#NULL!	#NULL!
2,00	2,00	#NULL!	#NULL!	#NULL!	#NULL!	#NULL!
2,00	2,00	#NULL!	#NULL!	#NULL!	#NULL!	#NULL!
2,00	2,00	#NULL!	#NULL!	#NULL!	#NULL!	#NULL!
2,00	2,00	#NULL!	#NULL!	#NULL!	#NULL!	#NULL!
2,00	2,00	#NULL!	#NULL!	#NULL!	#NULL!	#NULL!
2,00	2,00	#NULL!	#NULL!	#NULL!	#NULL!	#NULL!
2,00	2,00	#NULL!	#NULL!	#NULL!	#NULL!	#NULL!
2,00	2,00	#NULL!	#NULL!	#NULL!	#NULL!	#NULL!
2,00	2,00	#NULL!	#NULL!	#NULL!	#NULL!	#NULL!
2,00	2,00	#NULL!	#NULL!	#NULL!	#NULL!	#NULL!
2,00	2,00	#NULL!	#NULL!	#NULL!	#NULL!	#NULL!
2,00	2,00	#NULL!	#NULL!	#NULL!	#NULL!	#NULL!
2,00	2,00	#NULL!	#NULL!	#NULL!	#NULL!	#NULL!
2,00	2,00	#NULL!	#NULL!	#NULL!	#NULL!	#NULL!
2,00	2,00	#NULL!	#NULL!	#NULL!	#NULL!	#NULL!
2,00	2,00	#NULL!	#NULL!	#NULL!	#NULL!	#NULL!
2,00	2,00	#NULL!	#NULL!	#NULL!	#NULL!	#NULL!
2,00	2,00	#NULL!	#NULL!	#NULL!	#NULL!	#NULL!
2,00	2,00	#NULL!	#NULL!	#NULL!	#NULL!	#NULL!
2,00	2,00	#NULL!	#NULL!	#NULL!	#NULL!	#NULL!
2,00	2,00	#NULL!	#NULL!	#NULL!	#NULL!	#NULL!
2,00	2,00	#NULL!	#NULL!	#NULL!	#NULL!	#NULL!
2,00	2,00	#NULL!	#NULL!	#NULL!	#NULL!	#NULL!
2,00	2,00	#NULL!	#NULL!	#NULL!	#NULL!	#NULL!
2,00	2,00	#NULL!	#NULL!	#NULL!	#NULL!	#NULL!
2,00	2,00	#NULL!	#NULL!	#NULL!	#NULL!	#NULL!
2,00	2,00	#NULL!	#NULL!	#NULL!	#NULL!	#NULL!
2,00	2,00	#NULL!	#NULL!	#NULL!	#NULL!	#NULL!

2,00	2,00	#NULL!	#NULL!	#NULL!	#NULL!	#NULL!
2,00	2,00	#NULL!	#NULL!	#NULL!	#NULL!	#NULL!
2,00	2,00	#NULL!	#NULL!	#NULL!	#NULL!	#NULL!
2,00	2,00	#NULL!	#NULL!	#NULL!	#NULL!	#NULL!
2,00	2,00	#NULL!	#NULL!	#NULL!	#NULL!	#NULL!
2,00	2,00	#NULL!	#NULL!	#NULL!	#NULL!	#NULL!
2,00	2,00	#NULL!	#NULL!	#NULL!	#NULL!	#NULL!
2,00	2,00	#NULL!	#NULL!	#NULL!	#NULL!	#NULL!
2,00	2,00	#NULL!	#NULL!	#NULL!	#NULL!	#NULL!
2,00	2,00	#NULL!	#NULL!	#NULL!	#NULL!	#NULL!
2,00	2,00	#NULL!	#NULL!	#NULL!	#NULL!	#NULL!
2,00	2,00	#NULL!	#NULL!	#NULL!	#NULL!	#NULL!
2,00	2,00	#NULL!	#NULL!	#NULL!	#NULL!	#NULL!
2,00	2,00	#NULL!	#NULL!	#NULL!	#NULL!	#NULL!
2,00	2,00	#NULL!	#NULL!	#NULL!	#NULL!	#NULL!
2,00	2,00	#NULL!	#NULL!	#NULL!	#NULL!	#NULL!
2,00	2,00	#NULL!	#NULL!	#NULL!	#NULL!	#NULL!
2,00	2,00	#NULL!	#NULL!	#NULL!	#NULL!	#NULL!
2,00	2,00	#NULL!	#NULL!	#NULL!	#NULL!	#NULL!
2,00	2,00	#NULL!	#NULL!	#NULL!	#NULL!	#NULL!
2,00	2,00	#NULL!	#NULL!	#NULL!	#NULL!	#NULL!
2,00	2,00	#NULL!	#NULL!	#NULL!	#NULL!	#NULL!
2,00	2,00	#NULL!	#NULL!	#NULL!	#NULL!	#NULL!
2,00	2,00	#NULL!	#NULL!	#NULL!	#NULL!	#NULL!
2,00	2,00	#NULL!	#NULL!	#NULL!	#NULL!	#NULL!
2,00	2,00	#NULL!	#NULL!	#NULL!	#NULL!	#NULL!
2,00	2,00	#NULL!	#NULL!	#NULL!	#NULL!	#NULL!
2,00	2,00	#NULL!	#NULL!	#NULL!	#NULL!	#NULL!
2,00	2,00	#NULL!	#NULL!	#NULL!	#NULL!	#NULL!
2,00	2,00	#NULL!	#NULL!	#NULL!	#NULL!	#NULL!
2,00	2,00	#NULL!	#NULL!	#NULL!	#NULL!	#NULL!
2,00	2,00	#NULL!	#NULL!	#NULL!	#NULL!	#NULL!
2,00	2,00	#NULL!	#NULL!	#NULL!	#NULL!	#NULL!
2,00	2,00	#NULL!	#NULL!	#NULL!	#NULL!	#NULL!
2,00	2,00	#NULL!	#NULL!	#NULL!	#NULL!	#NULL!
2,00	2,00	#NULL!	#NULL!	#NULL!	#NULL!	#NULL!
2,00	2,00	#NULL!	#NULL!	#NULL!	#NULL!	#NULL!
2,00	2,00	#NULL!	#NULL!	#NULL!	#NULL!	#NULL!
1,00	2,00	#NULL!	#NULL!	#NULL!	#NULL!	#NULL!
2,00	2,00	#NULL!	#NULL!	#NULL!	#NULL!	#NULL!
2,00	2,00	#NULL!	#NULL!	#NULL!	#NULL!	#NULL!
2,00	2,00	#NULL!	#NULL!	#NULL!	#NULL!	#NULL!
2,00	2,00	#NULL!	#NULL!	#NULL!	#NULL!	#NULL!
2,00	2,00	#NULL!	#NULL!	#NULL!	#NULL!	#NULL!
2,00	2,00	#NULL!	#NULL!	#NULL!	#NULL!	#NULL!
2,00	2,00	#NULL!	#NULL!	#NULL!	#NULL!	#NULL!
2,00	2,00	#NULL!	#NULL!	#NULL!	#NULL!	#NULL!
2,00	2,00	#NULL!	#NULL!	#NULL!	#NULL!	#NULL!
2,00	2,00	#NULL!	#NULL!	#NULL!	#NULL!	#NULL!
2,00	2,00	#NULL!	#NULL!	#NULL!	#NULL!	#NULL!

2,00	2,00	#NULL!	#NULL!	#NULL!	#NULL!	#NULL!
2,00	2,00	#NULL!	#NULL!	#NULL!	#NULL!	#NULL!
2,00	2,00	#NULL!	#NULL!	#NULL!	#NULL!	#NULL!
2,00	2,00	#NULL!	#NULL!	#NULL!	#NULL!	#NULL!
2,00	2,00	#NULL!	#NULL!	#NULL!	#NULL!	#NULL!
2,00	2,00	#NULL!	#NULL!	#NULL!	#NULL!	#NULL!
2,00	2,00	#NULL!	#NULL!	#NULL!	#NULL!	#NULL!
2,00	2,00	#NULL!	#NULL!	#NULL!	#NULL!	#NULL!
2,00	2,00	#NULL!	#NULL!	#NULL!	#NULL!	#NULL!
2,00	2,00	#NULL!	#NULL!	#NULL!	#NULL!	#NULL!
2,00	2,00	#NULL!	#NULL!	#NULL!	#NULL!	#NULL!
2,00	2,00	#NULL!	#NULL!	#NULL!	#NULL!	#NULL!
2,00	2,00	#NULL!	#NULL!	#NULL!	#NULL!	#NULL!
1,00	2,00	#NULL!	#NULL!	#NULL!	#NULL!	#NULL!
2,00	2,00	#NULL!	#NULL!	#NULL!	#NULL!	#NULL!
2,00	2,00	#NULL!	#NULL!	#NULL!	#NULL!	#NULL!
2,00	2,00	#NULL!	#NULL!	#NULL!	#NULL!	#NULL!
2,00	2,00	#NULL!	#NULL!	#NULL!	#NULL!	#NULL!
2,00	2,00	#NULL!	#NULL!	#NULL!	#NULL!	#NULL!
2,00	2,00	#NULL!	#NULL!	#NULL!	#NULL!	#NULL!
2,00	2,00	#NULL!	#NULL!	#NULL!	#NULL!	#NULL!
2,00	2,00	#NULL!	#NULL!	#NULL!	#NULL!	#NULL!
2,00	2,00	#NULL!	#NULL!	#NULL!	#NULL!	#NULL!
2,00	2,00	#NULL!	#NULL!	#NULL!	#NULL!	#NULL!
2,00	2,00	#NULL!	#NULL!	#NULL!	#NULL!	#NULL!
2,00	2,00	#NULL!	#NULL!	#NULL!	#NULL!	#NULL!
2,00	2,00	#NULL!	#NULL!	#NULL!	#NULL!	#NULL!
2,00	2,00	#NULL!	#NULL!	#NULL!	#NULL!	#NULL!
2,00	2,00	#NULL!	#NULL!	#NULL!	#NULL!	#NULL!
2,00	2,00	#NULL!	#NULL!	#NULL!	#NULL!	#NULL!
2,00	2,00	#NULL!	#NULL!	#NULL!	#NULL!	#NULL!
2,00	2,00	#NULL!	#NULL!	#NULL!	#NULL!	#NULL!
2,00	2,00	#NULL!	#NULL!	#NULL!	#NULL!	#NULL!
2,00	2,00	#NULL!	#NULL!	#NULL!	#NULL!	#NULL!
2,00	2,00	#NULL!	#NULL!	#NULL!	#NULL!	#NULL!
2,00	2,00	#NULL!	#NULL!	#NULL!	#NULL!	#NULL!
2,00	2,00	#NULL!	#NULL!	#NULL!	#NULL!	#NULL!
2,00	2,00	#NULL!	#NULL!	#NULL!	#NULL!	#NULL!
2,00	2,00	#NULL!	#NULL!	#NULL!	#NULL!	#NULL!
2,00	2,00	#NULL!	#NULL!	#NULL!	#NULL!	#NULL!
2,00	2,00	#NULL!	#NULL!	#NULL!	#NULL!	#NULL!
2,00	2,00	#NULL!	#NULL!	#NULL!	#NULL!	#NULL!
2,00	2,00	#NULL!	#NULL!	#NULL!	#NULL!	#NULL!
2,00	2,00	#NULL!	#NULL!	#NULL!	#NULL!	#NULL!
2,00	2,00	#NULL!	#NULL!	#NULL!	#NULL!	#NULL!
2,00	2,00	#NULL!	#NULL!	#NULL!	#NULL!	#NULL!
2,00	2,00	#NULL!	#NULL!	#NULL!	#NULL!	#NULL!
2,00	2,00	#NULL!	#NULL!	#NULL!	#NULL!	#NULL!
2,00	2,00	#NULL!	#NULL!	#NULL!	#NULL!	#NULL!
2,00	2,00	#NULL!	#NULL!	#NULL!	#NULL!	#NULL!

2,00	2,00	#NULL!	#NULL!	#NULL!	#NULL!	#NULL!
2,00	2,00	#NULL!	#NULL!	#NULL!	#NULL!	#NULL!
2,00	2,00	#NULL!	#NULL!	#NULL!	#NULL!	#NULL!
2,00	2,00	#NULL!	#NULL!	#NULL!	#NULL!	#NULL!
2,00	2,00	#NULL!	#NULL!	#NULL!	#NULL!	#NULL!
2,00	2,00	#NULL!	#NULL!	#NULL!	#NULL!	#NULL!
2,00	2,00	#NULL!	#NULL!	#NULL!	#NULL!	#NULL!
2,00	2,00	#NULL!	#NULL!	#NULL!	#NULL!	#NULL!
2,00	2,00	#NULL!	#NULL!	#NULL!	#NULL!	#NULL!
2,00	2,00	#NULL!	#NULL!	#NULL!	#NULL!	#NULL!
2,00	2,00	#NULL!	#NULL!	#NULL!	#NULL!	#NULL!
2,00	2,00	#NULL!	#NULL!	#NULL!	#NULL!	#NULL!
2,00	2,00	#NULL!	#NULL!	#NULL!	#NULL!	#NULL!
2,00	2,00	#NULL!	#NULL!	#NULL!	#NULL!	#NULL!
2,00	2,00	#NULL!	#NULL!	#NULL!	#NULL!	#NULL!
2,00	2,00	#NULL!	#NULL!	#NULL!	#NULL!	#NULL!
2,00	2,00	#NULL!	#NULL!	#NULL!	#NULL!	#NULL!
2,00	2,00	#NULL!	#NULL!	#NULL!	#NULL!	#NULL!
2,00	2,00	#NULL!	#NULL!	#NULL!	#NULL!	#NULL!
2,00	2,00	#NULL!	#NULL!	#NULL!	#NULL!	#NULL!
2,00	2,00	#NULL!	#NULL!	#NULL!	#NULL!	#NULL!
2,00	2,00	#NULL!	#NULL!	#NULL!	#NULL!	#NULL!
2,00	2,00	#NULL!	#NULL!	#NULL!	#NULL!	#NULL!
2,00	2,00	#NULL!	#NULL!	#NULL!	#NULL!	#NULL!
2,00	2,00	#NULL!	#NULL!	#NULL!	#NULL!	#NULL!
1,00	2,00	#NULL!	#NULL!	#NULL!	#NULL!	#NULL!
2,00	2,00	#NULL!	#NULL!	#NULL!	#NULL!	#NULL!
2,00	2,00	#NULL!	#NULL!	#NULL!	#NULL!	#NULL!
2,00	2,00	#NULL!	#NULL!	#NULL!	#NULL!	#NULL!
2,00	2,00	#NULL!	#NULL!	#NULL!	#NULL!	#NULL!
2,00	2,00	#NULL!	#NULL!	#NULL!	#NULL!	#NULL!
2,00	2,00	#NULL!	#NULL!	#NULL!	#NULL!	#NULL!
2,00	2,00	#NULL!	#NULL!	#NULL!	#NULL!	#NULL!
2,00	2,00	#NULL!	#NULL!	#NULL!	#NULL!	#NULL!
2,00	2,00	#NULL!	#NULL!	#NULL!	#NULL!	#NULL!
2,00	2,00	#NULL!	#NULL!	#NULL!	#NULL!	#NULL!
2,00	2,00	#NULL!	#NULL!	#NULL!	#NULL!	#NULL!
2,00	2,00	#NULL!	#NULL!	#NULL!	#NULL!	#NULL!
2,00	2,00	#NULL!	#NULL!	#NULL!	#NULL!	#NULL!
2,00	2,00	#NULL!	#NULL!	#NULL!	#NULL!	#NULL!
2,00	2,00	#NULL!	#NULL!	#NULL!	#NULL!	#NULL!
2,00	2,00	#NULL!	#NULL!	#NULL!	#NULL!	#NULL!
2,00	2,00	#NULL!	#NULL!	#NULL!	#NULL!	#NULL!
2,00	2,00	#NULL!	#NULL!	#NULL!	#NULL!	#NULL!
2,00	2,00	#NULL!	#NULL!	#NULL!	#NULL!	#NULL!
2,00	2,00	#NULL!	#NULL!	#NULL!	#NULL!	#NULL!
2,00	2,00	#NULL!	#NULL!	#NULL!	#NULL!	#NULL!
2,00	2,00	#NULL!	#NULL!	#NULL!	#NULL!	#NULL!
2,00	2,00	#NULL!	#NULL!	#NULL!	#NULL!	#NULL!
2,00	2,00	#NULL!	#NULL!	#NULL!	#NULL!	#NULL!

2,00	2,00	#NULL!	#NULL!	#NULL!	#NULL!	#NULL!
2,00	2,00	#NULL!	#NULL!	#NULL!	#NULL!	#NULL!
2,00	2,00	#NULL!	#NULL!	#NULL!	#NULL!	#NULL!
2,00	2,00	#NULL!	#NULL!	#NULL!	#NULL!	#NULL!
2,00	2,00	#NULL!	#NULL!	#NULL!	#NULL!	#NULL!
2,00	2,00	#NULL!	#NULL!	#NULL!	#NULL!	#NULL!
2,00	2,00	#NULL!	#NULL!	#NULL!	#NULL!	#NULL!
2,00	2,00	#NULL!	#NULL!	#NULL!	#NULL!	#NULL!
2,00	2,00	#NULL!	#NULL!	#NULL!	#NULL!	#NULL!
2,00	2,00	#NULL!	#NULL!	#NULL!	#NULL!	#NULL!
2,00	2,00	#NULL!	#NULL!	#NULL!	#NULL!	#NULL!
2,00	2,00	#NULL!	#NULL!	#NULL!	#NULL!	#NULL!
2,00	2,00	#NULL!	#NULL!	#NULL!	#NULL!	#NULL!
2,00	2,00	#NULL!	#NULL!	#NULL!	#NULL!	#NULL!
2,00	2,00	#NULL!	#NULL!	#NULL!	#NULL!	#NULL!
2,00	2,00	#NULL!	#NULL!	#NULL!	#NULL!	#NULL!
2,00	2,00	#NULL!	#NULL!	#NULL!	#NULL!	#NULL!
2,00	2,00	#NULL!	#NULL!	#NULL!	#NULL!	#NULL!
2,00	1,00	1,00	1,00	2,00	2,00	#NULL!
2,00	2,00	#NULL!	#NULL!	#NULL!	#NULL!	#NULL!
2,00	2,00	#NULL!	#NULL!	#NULL!	#NULL!	#NULL!
2,00	2,00	#NULL!	#NULL!	#NULL!	#NULL!	#NULL!
2,00	1,00	1,00	1,00	2,00	2,00	#NULL!
2,00	2,00	#NULL!	#NULL!	#NULL!	#NULL!	#NULL!
2,00	2,00	#NULL!	#NULL!	#NULL!	#NULL!	#NULL!
2,00	2,00	#NULL!	#NULL!	#NULL!	#NULL!	#NULL!
2,00	1,00	1,00	1,00	2,00	2,00	#NULL!
2,00	2,00	#NULL!	#NULL!	#NULL!	#NULL!	#NULL!
2,00	2,00	#NULL!	#NULL!	#NULL!	#NULL!	#NULL!
1,00	1,00	1,00	1,00	2,00	2,00	#NULL!
2,00	2,00	#NULL!	#NULL!	#NULL!	#NULL!	#NULL!
2,00	2,00	#NULL!	#NULL!	#NULL!	#NULL!	#NULL!
2,00	2,00	#NULL!	#NULL!	#NULL!	#NULL!	#NULL!
2,00	2,00	#NULL!	#NULL!	#NULL!	#NULL!	#NULL!
2,00	1,00	1,00	1,00	2,00	2,00	#NULL!
2,00	2,00	#NULL!	#NULL!	#NULL!	#NULL!	#NULL!
2,00	2,00	#NULL!	#NULL!	#NULL!	#NULL!	#NULL!
2,00	2,00	#NULL!	#NULL!	#NULL!	#NULL!	#NULL!
2,00	1,00	1,00	1,00	2,00	2,00	#NULL!
2,00	1,00	1,00	2,00	1,00	2,00	#NULL!
2,00	2,00	#NULL!	#NULL!	#NULL!	#NULL!	#NULL!
2,00	2,00	#NULL!	#NULL!	#NULL!	#NULL!	#NULL!
2,00	1,00	1,00	2,00	1,00	2,00	#NULL!
2,00	2,00	#NULL!	#NULL!	#NULL!	#NULL!	#NULL!
2,00	2,00	#NULL!	#NULL!	#NULL!	#NULL!	#NULL!
2,00	2,00	#NULL!	#NULL!	#NULL!	#NULL!	#NULL!
2,00	2,00	#NULL!	#NULL!	#NULL!	#NULL!	#NULL!
2,00	1,00	2,00	1,00	2,00	2,00	#NULL!
2,00	2,00	#NULL!	#NULL!	#NULL!	#NULL!	#NULL!
2,00	2,00	#NULL!	#NULL!	#NULL!	#NULL!	#NULL!

2,00	2,00	#NULL!	#NULL!	#NULL!	#NULL!	#NULL!
